# Supplementary material for: VISTA checkpoint inhibition by pH-selective antibody SNS-101 with optimized safety and pharmacokinetic profiles enhances PD-1 response
Source: Nat Commun. 2024 Apr 4;15:2917. doi: 10.1038/s41467-024-47256-x (PMC10995192; doi:10.1038/s41467-024-47256-x)
Supplement: Supplementary file 1 — Supplementary Information [file 41467_2024_47256_MOESM1_ESM.pdf]

**Supplementary Information for the manuscript:**

# VISTA checkpoint inhibition by pH-selective antibody SNS-101 with optimized safety and pharmacokinetic profiles enhances PD-1 therapeutic response

Thomas Thisted[1], F. Donelson Smith[1], Arnab Mukherjee[1], Yuliya Kleschenko[1], Feng Feng[1], Zhi-Gang Jiang[1], Timothy Eitas[1], Kanam Malhotra[1], Zuzana Biesova[1], Adejumoke Onumajuru[1], Faith Finley[1], Anokhi Cifuentes[1], Guolin Zhang[1], Gaëlle H. Martin[2], Yoshiko Takeuchi[3], Kader Thiam[2], Robert D. Schreiber[3] & Edward H. van der Horst[1*]

[1]Sensei Biotherapeutics, Inc., 1405 Research Blvd, Suite 125, Rockville, MD 20850
[2]genOway, Technopark Gerland, 69007 Lyon, France
[3]Washington Univ. School Medicine, Department of Pathology and Immunology, 425 South Euclid Ave, St. Louis, MO 63110

## Description of Supplementary Files

Supplementary Information:

Supplementary Figures 1 – 13
Supplementary Tables 1 – 4

Supplementary Data File:

“Supplemental - General Toxicology Report.pdf”

Toxicology report and comprehensive clinical endpoints from NHP studies.

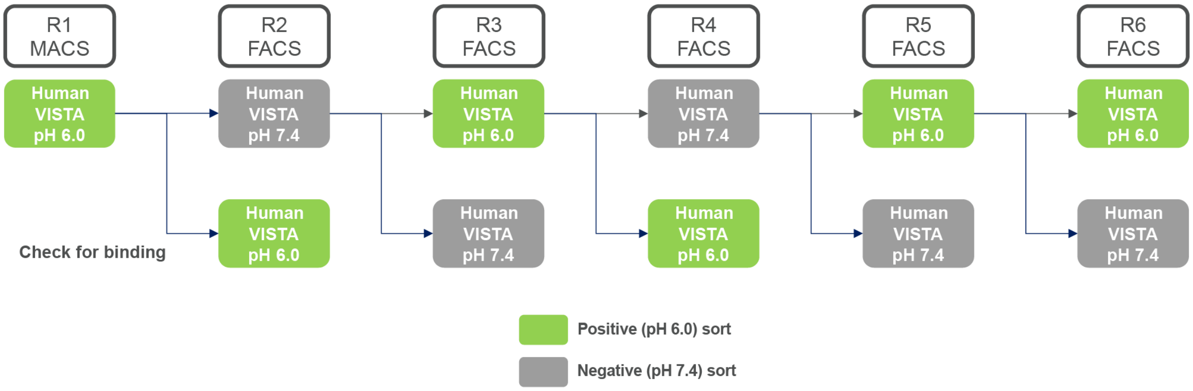


**Supplementary Figure 1: Selection scheme for SNS-101 antibody discovery.** pH-dependent human IgGs that bind human VISTA were selected from yeast surface display libraries through iterative cycles alternating between positive enrichment rounds at pH 6.0 and negative selection rounds at pH 7.4 (Adimab LLC). The antigen and antibody-expressing yeast populations were incubated at the described pH within the round and that pH was maintained during secondary labeling and sorting. Round of selection is indicated at top. MACS = Magnetic Activated Cell Sorting. FACS = Fluorescence Activated Cell Sorting.

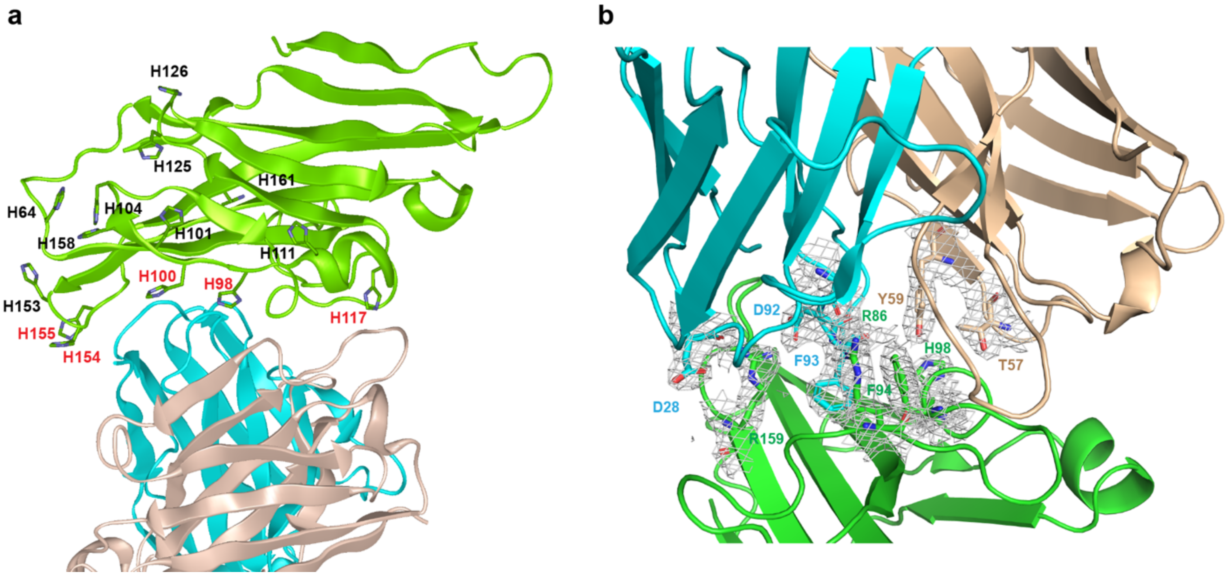


**Supplementary Figure 2: Crystal structure of VISTA:SNS-101 Fab complex. a,** All surface histidines of VISTA-ECD highlighted. Histidine residues located at the binding interface and non-interface residues are labeled in red and black, respectively. VISTA–green; SNS-101-Fab LC–Cyan; SNS-101-Fab HC–Irish cream. **b,** Image of the electron density map (2Fo-Fc map contoured at 1 sigma) includes the key contacting residues (sticks) at the VISTA:SNS-101 Fab interface. The orientation is maintained from Figure 1d. The electron density map is displayed in gray and otherwise the color scheme used as in (a).

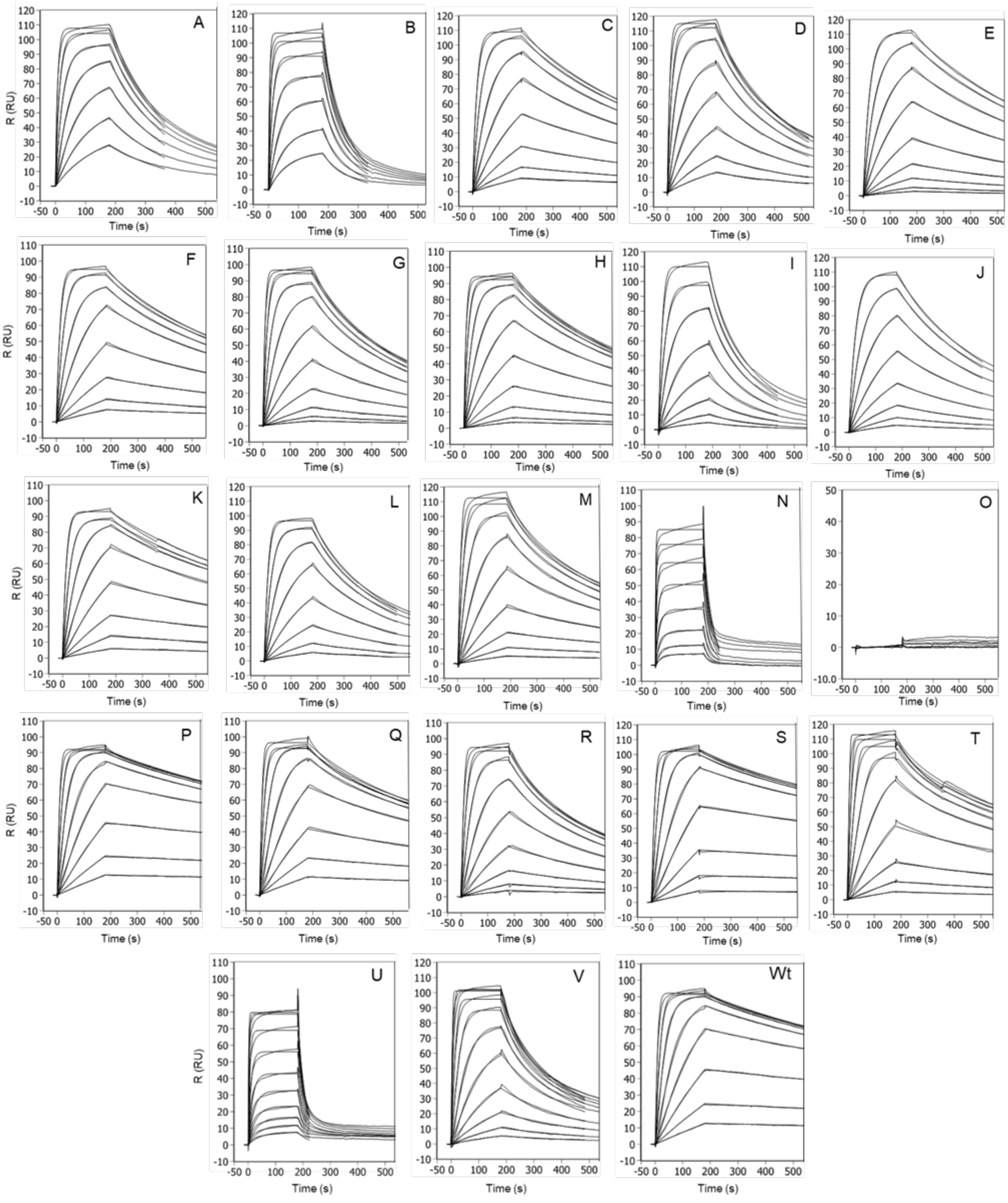


**Supplementary Figure 3:** SPR sensorgrams representing the interaction between VISTA variants and SNS-101. The binding affinities between SNS-101 and VISTA variants A) H153A/H154A/H155A; B) H98A/H100A; C) H101A/H104A; D) H158A/H161A;

E) H125A/H126A; F) H153A; G) H154A; H) H155A; I) H98A; J) H100A; K) H64A; L) H117A; M) H111A; N) R159A; O) R86A; P) S84A; Q) D96A; R) R116A; S) S110A; T) E157A; U) F94A; V) Q95A and wild-type VISTA (Wt) were measured at pH 5.8 (PBS, 0.05% Tween-20) and 25ºC. See associated Supplementary Table 3.

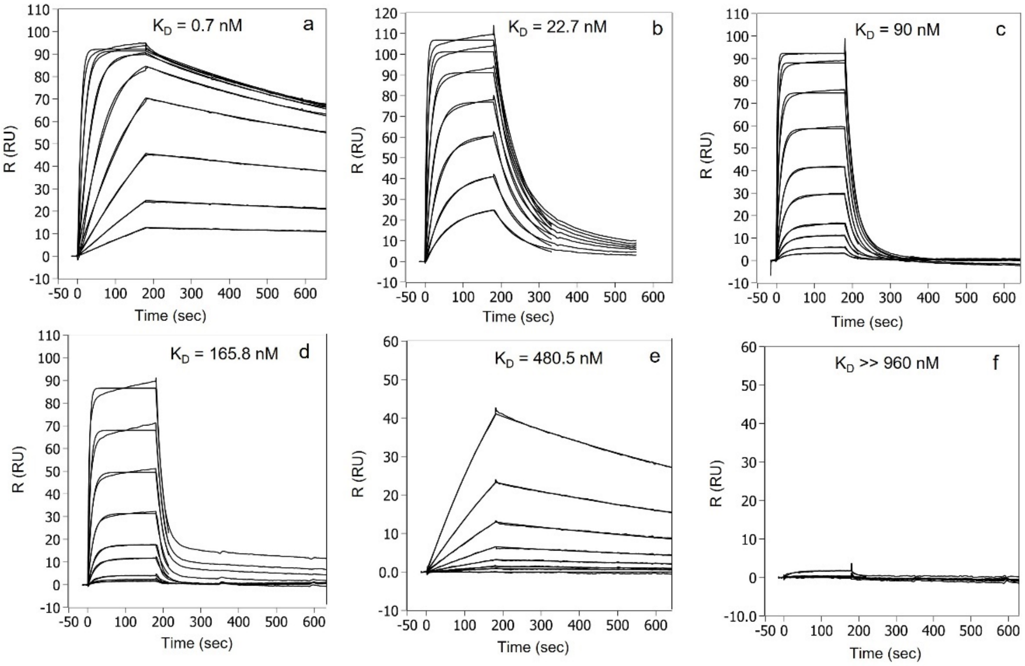


**Supplementary Figure 4:** Interaction of SNS-101 with VISTA variants containing combination of critical interface mutations. The interaction between SNS-101 and VISTA WT **a**, or VISTA variants **b**, H98A/H100A; **c**, H98A/H100A/H117A; **d**, H98A/H100A/H154A/H155A; **e**, H98A/H100A/H117A/R159A; **f**, H98A/H100A/H154A/H155A/R159A were measured at pH 5.8 (PBS, 0.05% Tween-20) and 25ºC. The measured dissociation constants are indicated within respective sensorgram. See associated Supplementary Table 3.

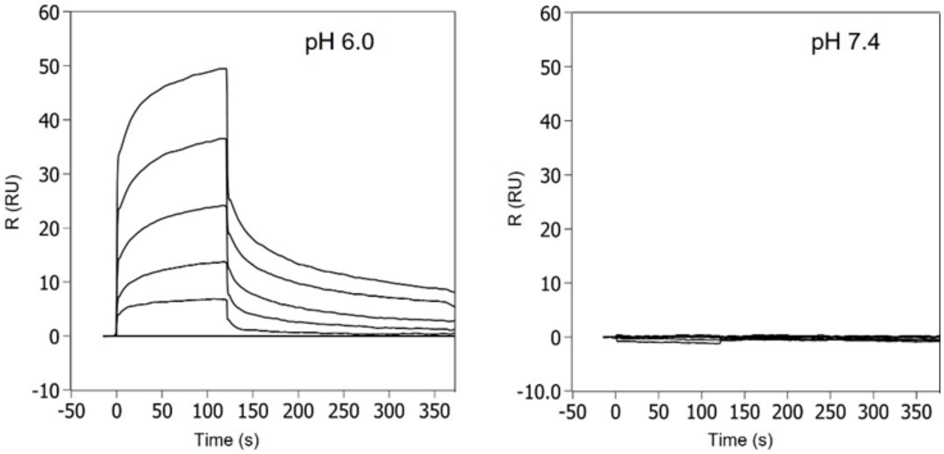


**Supplementary Figure 5:** The interaction between PSGL1-19mer-Fc and VISTA-HIS. The binding affinities between SNS-101 and VISTA-His were measured at pH 6.0 and pH 7.4 using PBS + 0.05% Tween-20 as running buffer. The dissociation constant ($K_D$ ~ 600 nM) at pH 6.0 was calculated from equilibrium analysis.

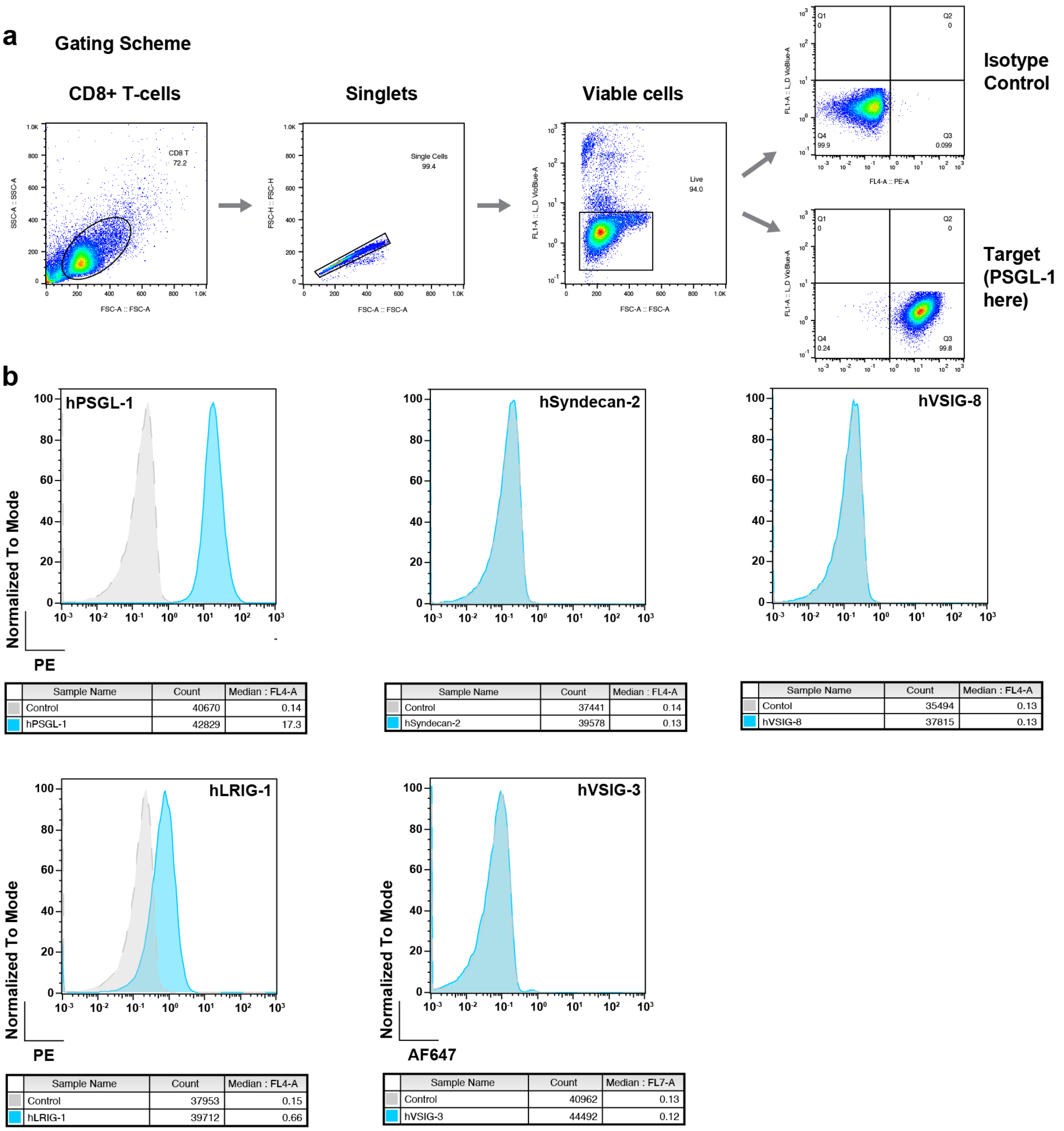


| Sample Name | Count | Median : FL4-A |
|---|---|---|
| Control | 40670 | 0.14 |
| hPSGL-1 | 42829 | 17.3 |

| Sample Name | Count | Median : FL4-A |
|---|---|---|
| Control | 37441 | 0.14 |
| hSyndecan-2 | 39578 | 0.13 |

| Sample Name | Count | Median : FL4-A |
|---|---|---|
| Contol | 35494 | 0.13 |
| hVSIG-8 | 37815 | 0.13 |

| Sample Name | Count | Median : FL4-A |
|---|---|---|
| Control | 37953 | 0.15 |
| hLRIG-1 | 39712 | 0.66 |

| Sample Name | Count | Median : FL7-A |
|---|---|---|
| Control | 40962 | 0.13 |
| hVSIG-3 | 44492 | 0.12 |

**Supplementary Figure 6:** Expression analysis of PSGL-1, Syndecan-2, LRIG-1, VSIG-3 and VSIG-8 on primary T-cells by flow cytometry. **a,** Gating scheme for experiment. **b,** CD8$^{+}$ T-cells (activated and cultured as in Fig. 2g & h and as described in Methods) were analyzed by flow cytometry using Phycoerythrin or AF647-labelled anti-PSGL-1 (BioLegend 328805), Syndecan-2 (R&D Systems FAP2965P), LRIG-1 (R&D Systems FAB7498P), VSIG-3 (R&D Systems FAB92292R) and VSIG-8 (R&D Systems FAB9418P) (blue), and corresponding isotype controls (gray). Expression of PSGL-1 and to a lesser extent LRIG-1 was detected. Syndecan-2, VSIG-3 and VSIG-8 were not detected.

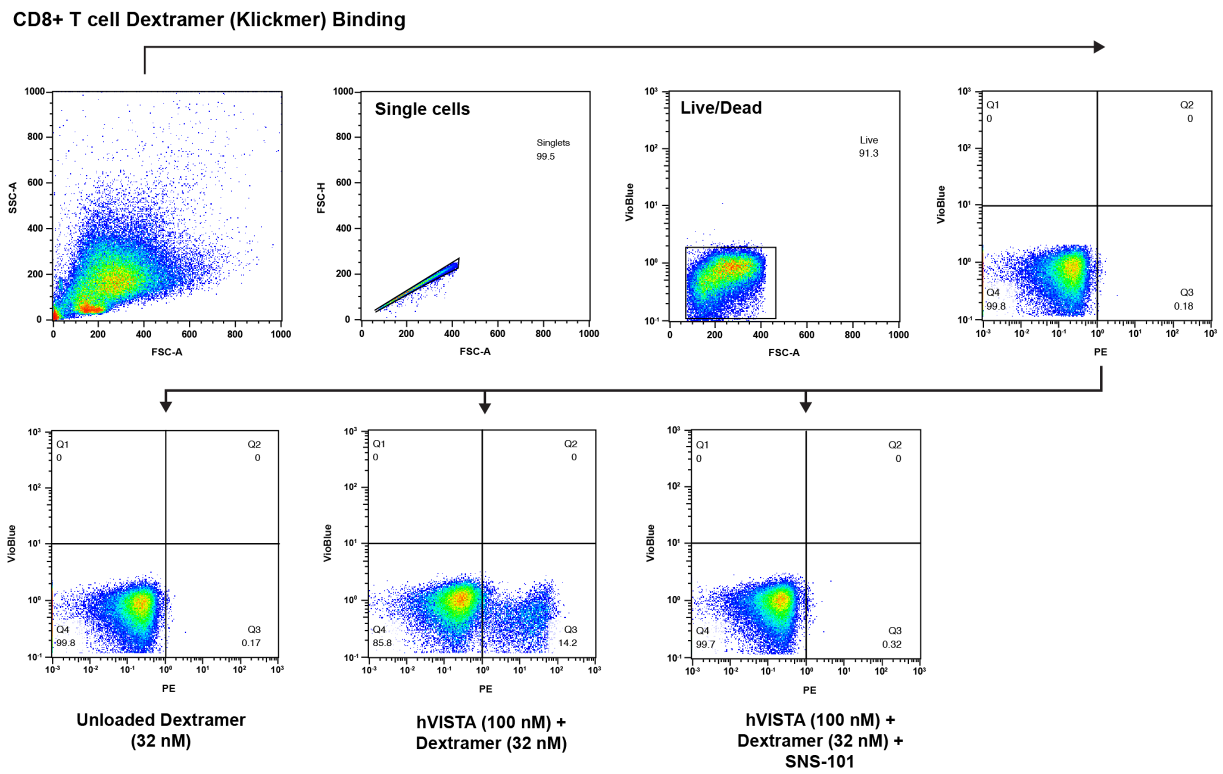


**Supplementary Figure 7:** Gating strategy and exemplary flow cytometry data showing CD8$^{+}$ T cell staining using the Dextramer reagent. Bottom left panel: Control staining by unloaded Dextramer. Bottom middle panel: Staining using 32nM Dextramer complexed with 100 nM hVISTA. Bottom right panel: As middle panel, but pre-incubated with 1 µM SNS-101. All experiments conducted at pH 6.0 as described in Methods.

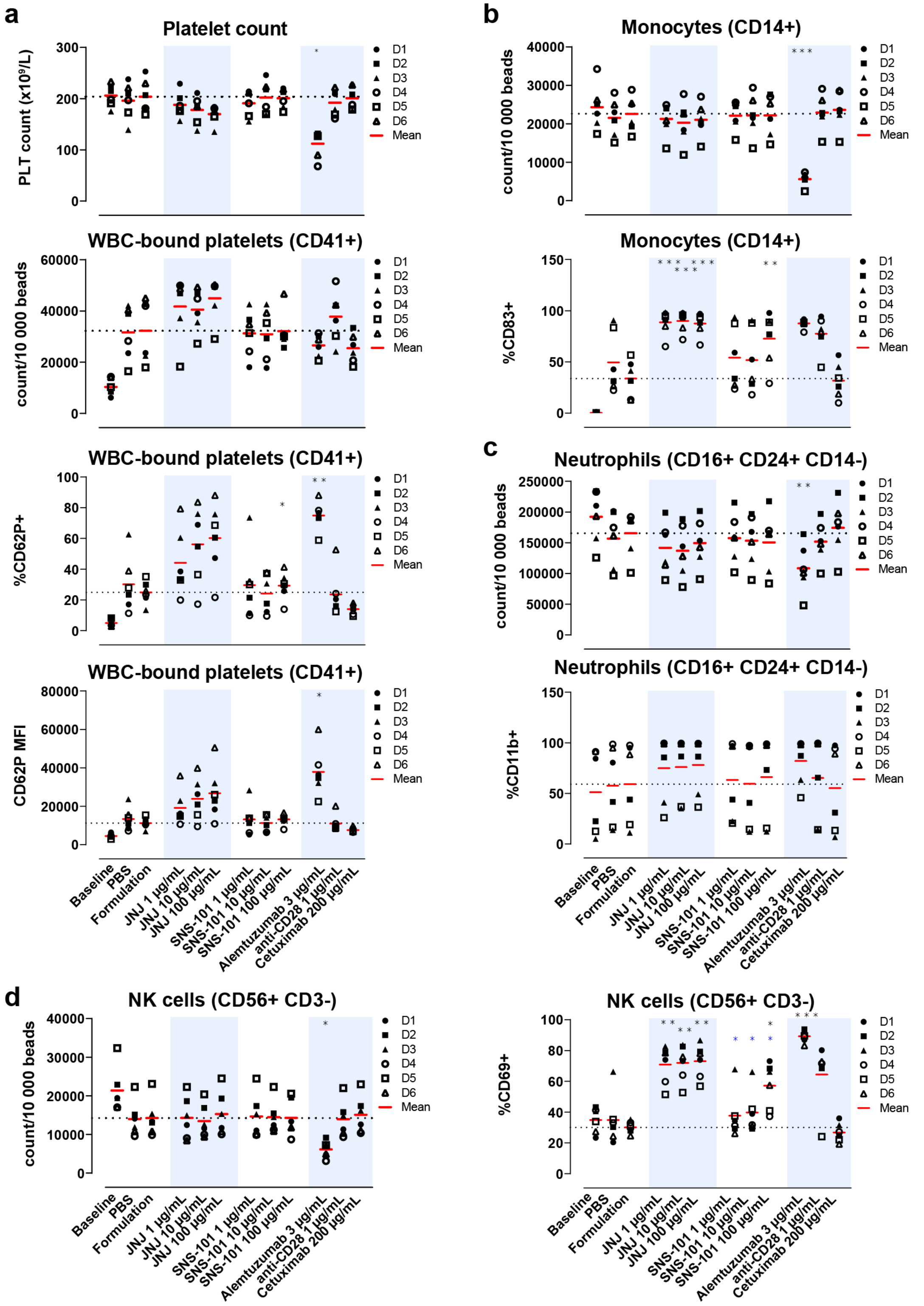


**Supplementary Figure 8:** ***Ex vivo*** **loop assay of human whole blood (ID.Flow) for assessment of CRS.** Fresh whole blood was taken from healthy volunteers (n=6) and a low amount of soluble heparin (allowing for analysis of drug-related effects on

complement or coagulation cascade systems) was added. Blood was immediately transferred to the ID.Flow system, followed by administration of the test items, and set to circulate at 37°C to prevent clotting. Blood was extracted at baseline and at 4 hours, and automatically counted using a Sysmex XN-L350 Hematology Analyzer. **a,** Platelet count (PLTs), white blood cell (WBC)-bound platelet counts per $10^4$ counting beads**,** proportions of leukocyte-bound ($CD62P^+$) platelets ($CD41^+$) and $CD62P^+$ WBC-bound platelet median fluorescence intensity (MFI). **b,** Monocyte counts per $10^4$ counting beads, and proportions of monocytes expressing CD83. **c,** Neutrophil cell counts per $10^4$ counting beads and proportions of neutrophils expressing CD11b. **d,** NK cell counts per $10^4$ counting beads and proportions of NK cells expressing CD69. The mean value for each group is indicated with a horizontal red line. The dotted line represents the average of the formulation buffer group. Paired Student's t-test was performed on the values followed by Holm-Sidak correction for multiple comparisons; control substances alemtuzumab, anti-CD28, and cetuximab were compared to PBS, while SNS-101- and JNJ-treated samples were compared to formulation buffer, and samples treated with the same concentration of SNS-101 and JNJ were compared to each other (indicated with blue stars); * P<0.05; ** P<0.01; *** P<0.001; **** P<0.0001; comparisons that did not reach statistical significance are not indicated. Exact P-values were as follows (non-significant values (P>0.05) not reported): Platelet count: PBS vs. Alemtuzumab, 3 µg/ml, P= 0.02380. WBC-bound platelets, %CD62P+: PBS vs. Alemtuzumab, 3 µg/ml, P= 0.0087; JNJ, 100 µg/ml vs. SNS-101, 100 µg/ml, P= 0.0261. WBC-bound platelets, CD62P MFI: PBS vs. Alemtuzumab, 3 µg/ml, P= 0.0247. Monocyte count: PBS vs. Alemtuzumab, 3 µg/ml, P= 0.000286. Monocytes, %CD83+: Formulation Buffer (FB) vs. JNJ, 1 µg/ml, P= 0.000320; FB vs. JNJ, 10 µg/ml, P= 0.000311; FB vs. JNJ, 100 µg/ml, P= 0.000320; FB vs. SNS-101, 100 µg/ml, P= 0.00210. Neutrophil count: PBS vs. Alemtuzumab, 3 µg/ml, p= 0.00750. NK cell count: PBS vs. Alemtuzumab, 3 µg/ml, P= 0.0132. NK cells, %CD69+: PBS vs. Alemtuzumab, 3 µg/ml, P= 0.000646; FB vs. JNJ, 1 µg/ml, P= 0.00314; FB vs. JNJ, 10 µg/ml, P= 0.00251; FB vs. JNJ, 100 µg/ml, P= 0.00191; FB vs. SNS-101, 100 µg/ml, P= 0.0297. Source data are provided as a Source Data file.

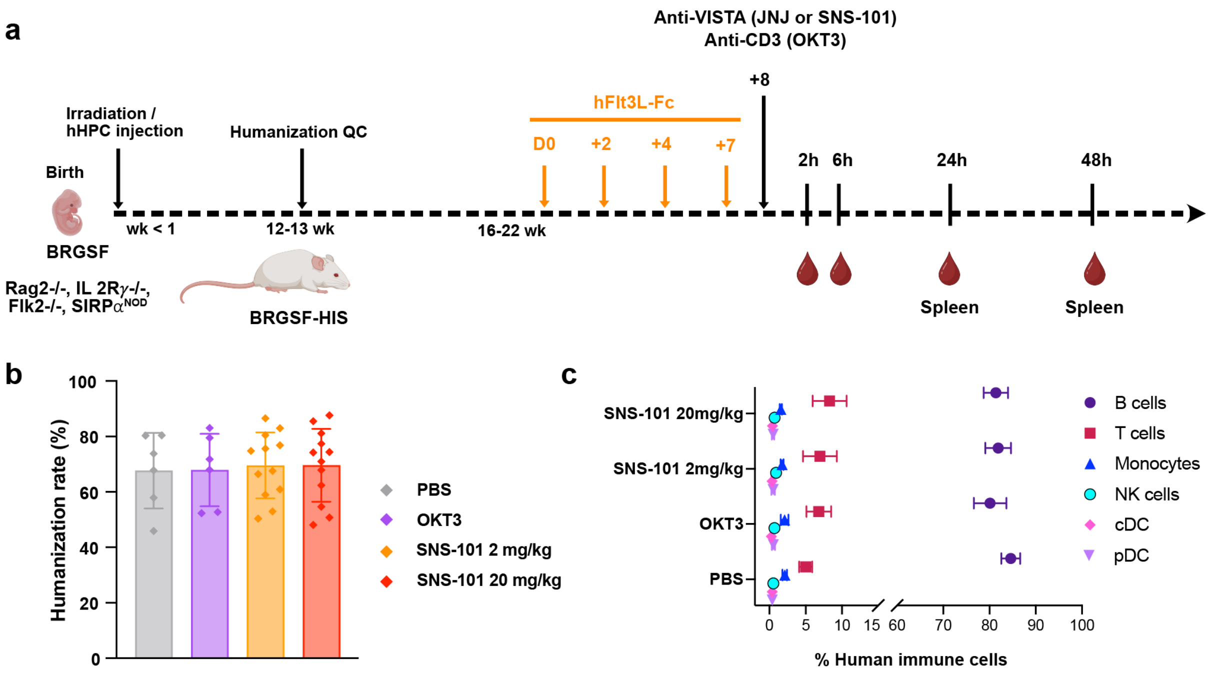


**Supplementary Figure 9: Characteristics of BRGSF-HIS humanized mouse model.** **a,** Schematic showing experimental time course. At birth, BRGSF mice (BALB/c $Rag^{2tm1Fwa}Il2r\gamma^{tm1Cgn}Sirp\alpha^{NOD}Flt3^{tm1Irl}$) are irradiated and after 24 hours undergo intra-hepatic transplantation with ~$0.9 \times 10^5$ human hematopoietic progenitor cells (hHPC; $CD34^+$ cord blood cells). Approximately 1 week prior to the start of experiment, mice (4 groups, n=10/group) are injected IP with 4 doses (10 µg/dose) of recombinant human Flt3L (BioXCell Cat BE0098-777120A1) to expand the myeloid compartment. Blood and spleen samples are taken at the indicated times post-antibody treatment (SNS-101, as indicated, or OKT3, 2 mg/kg; BioXCell Cat BE0001-2-730321A1). **b,** Humanization rate in the randomization groups evaluated in blood 12 weeks post-HPC injection. Data is expressed as mean ± SD. **c**, Percentage of main immune cell proportions. Data is expressed as mean ± SEM. Source data are provided as a Source Data file. Figure created in part using artwork from Biorender.com.

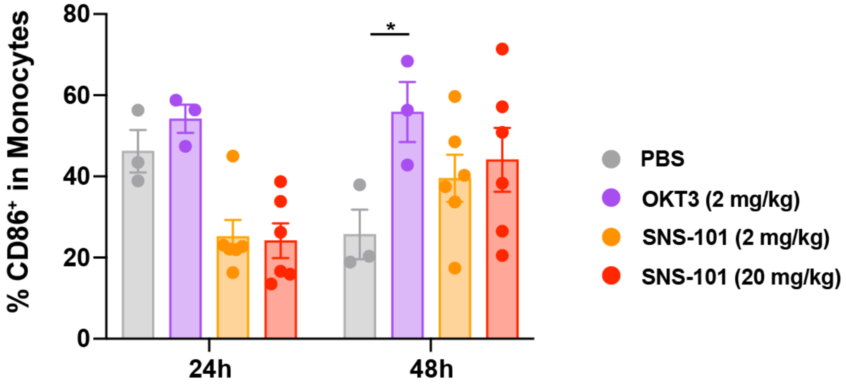


**Supplementary Figure 10: *In vivo* effect of SNS-101 on monocyte activation.** BRGSF-HIS mice (n=6 per time point) were dosed with indicated antibodies by IV injection and blood was collected at 24- and 48-hours post injection. Percentage of activated $CD3^{-}CD19^{-}CD14^{+}CD86^{+}$ monocytes was analyzed by flow cytometry analysis of isolated splenocytes. Statistical analysis was performed using 2-way ANOVA Multiple comparison; *P= 0.0388. Source data are provided as a Source Data file.

E.G7 tumors in C57BL/6-hVISTA-KI mice

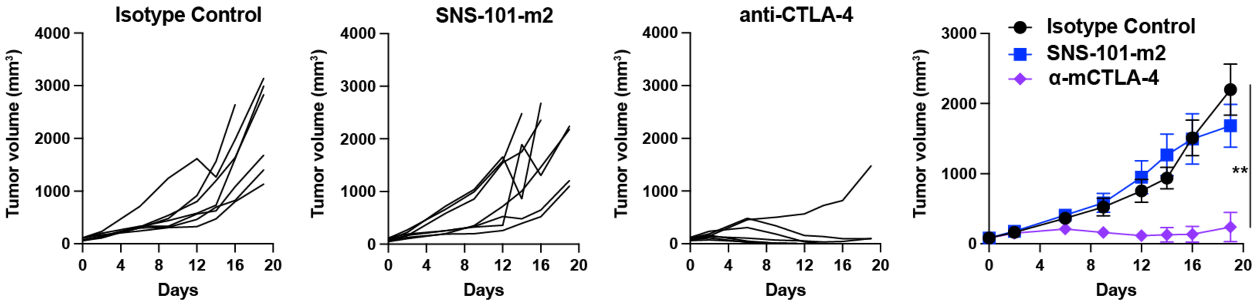


**Supplementary Figure 11: Comparison of anti-tumor activities of SNS-101-m2 and anti-CTLA-4 in EG.7 syngeneic tumors.** Tumors were established by subcutaneous injection of EG.7-OVA cells (1 x $10^6$ cells per animal, n=8 mice per group; ATCC CRL-2113) into female hVISTA-KI mice. When tumors reached ~ 80 $mm^3$, animals were randomized into treatment cohorts and treated with the indicated doses of isotype control IgGs, SNS-101-m2, or α-mouse CTLA-4 (clone 9H10, Bio X Cell BE0131) 3x per week for 3 weeks. Data shows spider plots of EG.7 tumor volume measurements for each mouse in a cohort and summary plot of mean tumor volume ± SEM. SNS-101-m2 had no significant effect on tumor volume, while anti-mCTLA-4 resulted in significant decreases in tumor volume relative to control (89%, P=0.0148). Significance was evaluated using Mann-Whitney unpaired two-sided t-test with P<0.05 considered to be statistically significant. Source data are provided as a Source Data file.

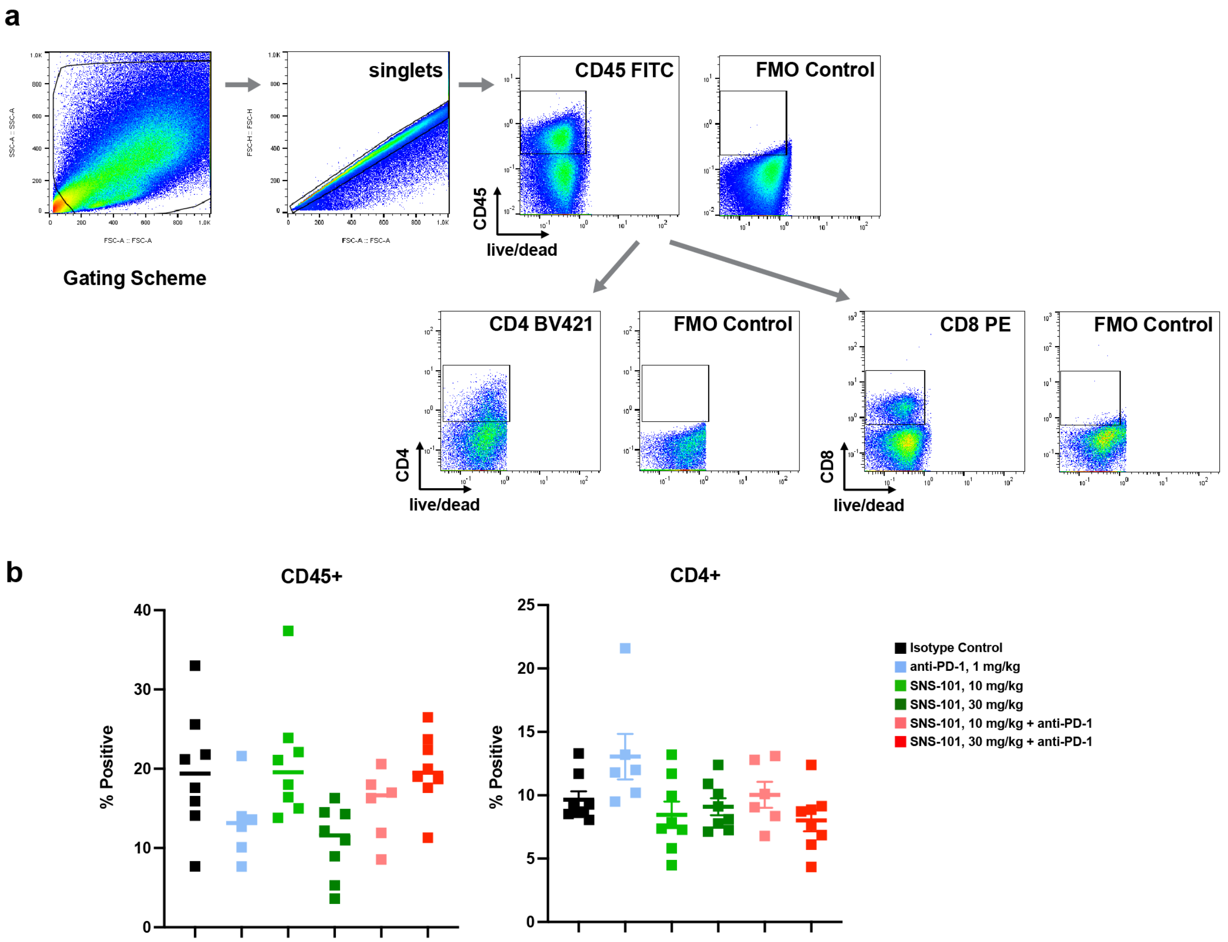


**Supplementary Figure 12: Analysis of leukocytes in MC38 tumors. a,** Gating scheme for analysis of tumor-infiltrating $CD4^+$ and $CD8^+$ T-cells (related to Fig. 6g). MC38 tumors were established and when tumors reached ~ 100 $mm^3$ (~ 1 week), animals were randomized into treatment cohorts and treated with the indicated doses of isotype control IgGs, SNS-101-m2, anti-mPD-1 (clone RMP1-14, Bio X Cell) or combinations, 2-3x/week for 3 weeks. Tumors were excised, dissociated and proportions of lymphocytes were measured by flow cytometry. **b,** Proportions of $CD45^+$ cells and $CD4^+$ T-cells (n=8 isotype control; n=6 anti-PD-1; n=8 SNS-101, 10 mg/kg; n=8 SNS-101, 30 mg/kg; n=6 SNS-101, 10 mg/kg + anti-PD-1, 1 mg/kg; n=8 SNS-101, 30 mg/kg + anti-PD-1, 1 mg/kg). One-way ANOVA, with Tukey Post-*Hoc* testing was used for statistical analysis (*$P < 0.05$). Source data are provided as a Source Data file.

**a** Gating Scheme for sorting from whole blood

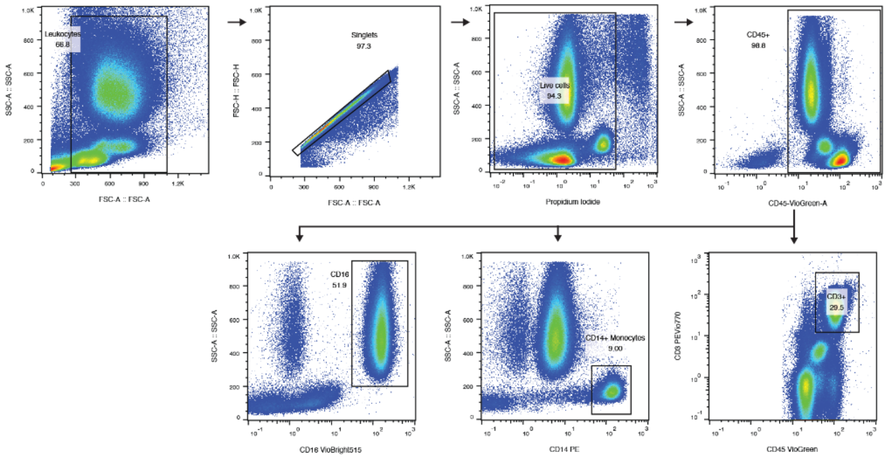


**b** Gating Scheme for sorting from isolated NK cells

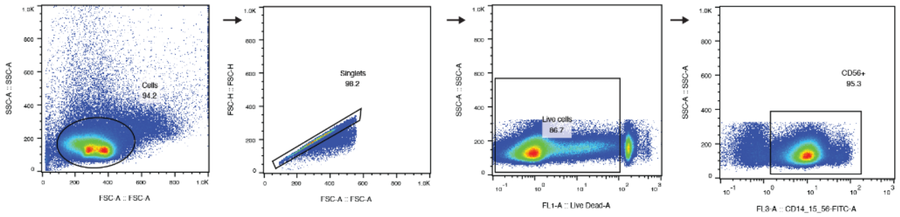


**c**

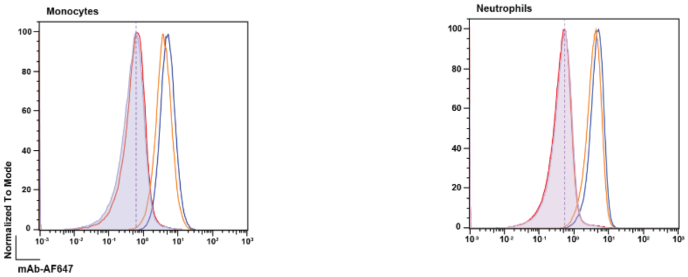


| Sample Name | Subset Name | Count | Median : Comp-FL7-A |
|---|---|---|---|
| Control IgG | CD14 | 42961 | 0.49 |
| h26A | CD14 | 44539 | 3.89 |
| JNJ | CD14 | 45719 | 5.10 |
| SNS-101 | CD14 | 44179 | 0.57 |

| Sample Name | Subset Name | Count | Median : Comp-FL7-A |
|---|---|---|---|
| Control IgG | CD16 | 270750 | 0.46 |
| h26A | CD16 | 266734 | 3.80 |
| JNJ | CD16 | 267438 | 4.59 |
| SNS-101 | CD16 | 318374 | 0.43 |

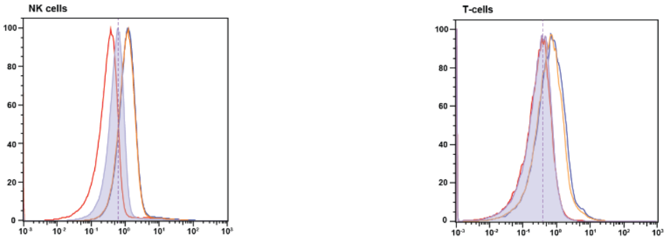


| Sample Name | Subset Name | Count | Median : Comp-FL7-A |
|---|---|---|---|
| Control IgG | CD56 | 248400 | 0.55 |
| h26A | CD56 | 214041 | 1.12 |
| JNJ | CD56 | 209286 | 1.16 |
| SNS-101 | CD56 | 237053 | 0.31 |

| Sample Name | Subset Name | Count | Median : Comp-FL7-A |
|---|---|---|---|
| Control IgG | CD3 | 29081 | 0.26 |
| h26A | CD3 | 31796 | 0.58 |
| JNJ | CD3 | 29855 | 0.64 |
| SNS-101 | CD3 | 32737 | 0.25 |

Control IgG
h26A
JNJ
SNS-101
(h26A, JNJ, SNS-101: anti-VISTA mAbs)

Fold Over Control

| | Monocytes | Neutrophils | NK cells | T-cells |
|---|---|---|---|---|
| Control | – | – | – | – |
| h26A | 7.9 | 8.3 | 2 | 2.2 |
| JNJ | 10.4 | 10 | 2.1 | 2.4 |
| SNS-101 | 1.2 | 0.9 | 0.5 | 1 |

**Supplementary Figure 13: Binding profiles of SNS-101, JNJ, h26A and hIgG isotype control to human monocytes, neutrophils, NK cells and T-cells. a,** Gating scheme for flow cytometry analysis from human whole blood. **b,** Gating scheme for flow cytometry analysis from commercially isolated NK cells. **c,** Binding profiles. Alexa Fluor 647-labeled anti-VISTA antibodies to the various human cell types was analyzed at pH 7.4. Fold over control values were calculated using MFI values of isotype control relative to the anti-VISTA antibodies.

**Supplementary Table 1 (related to Figure 1):**

| pH | $k_a$ ($M^{-1}s^{-1}$) | $k_d$ ($s^{-1}$) | $K_D$ (M) |
|---|---|---|---|
| **5.8** | 3.68E+06 | 1.29E-03 | 3.49E-10 |
| **6.0** | 2.74E+06 | 2.13E-03 | 7.79E-10 |
| **6.2** | 1.23E+06 | 2.19E-03 | 1.78E-09 |
| **6.4** | 5.39E+05 | 3.21E-03 | 5.96E-09 |
| **6.6** | 3.06E+05 | 5.66E-03 | 1.85E-08 |
| **6.8** | 2.10E+05 | 8.42E-03 | 4.01E-08 |
| **7.0** | 1.31E+05 | 1.01E-02 | 7.70E-08 |
| **7.2** | 1.10E+05 | 1.47E-02 | 1.34E-07 |
| **7.4** | 8.72E+04 | 3.08E-02 | 3.53E-07 |

**Supplementary Table 2: X-ray crystallography refinement statistics**

| | |
|---|---|
| Resolution [Å] | 158.41-2.59 |
| Number of reflections (working /test) | 24894 / 1291 |
| $R_{cryst}$ [%] | 23.8 |
| $R_{free}$[%][2] | 29.3 |
| Total number of atoms: | |
| Protein | 8708 |
| Water | 47 |
| N-acetyl-D-glucosamine | 42 |
| 1,2-Ethanediol | 8 |
| Average B-factors | |
| Protein | 57.2 |
| Water | 25.6 |
| Other atoms | 64.1 |
| Deviation from ideal geometry: [3] | |
| Bond lengths [Å] | 0.003 |
| Bond angles [°] | 0.94 |
| Bonded B's [Å$^2$] [4] | 1.7 |
| Ramachandran plot: [5] | |
| Favoured [%] | 94.57 |
| Allowed [%] | 5.34 |
| Outliers [%] | 0.09 |
| Molprobity score[5] | 1.44 |
| Molprobity clashscore[5] | 2.75 |

[1] Values as defined in REFMAC5, without sigma cut-off

[2] Test-set contains 4.9% of measured reflections

[3] Root mean square deviations from geometric target values

[4] Calculated with MOLEMAN

[5] Calculated with Molprobity

**Supplementary Table 3. SPR binding data for interaction between VISTA mutants and SNS-101.**

Binding affinities between SNS-101 and VISTA variants were measured at pH 5.8 (PBS, 0.05% Tween-20) and 25ºC. Table values correspond to sensorgram results in Supplementary Fig. 3 (top section) and Supplementary Fig. 4 (bottom section).

| VARIANT | $k_a$ ($M^{-1}s^{-1}$) | $k_d$ ($s^{-1}$) | $K_D$ (nM) |
|---|---|---|---|
| H153A/H154A/H155A | 2.8E+05 | 4.8E-03 | 17.1 |
| H98A/H100A | 6.2E+05 | 1.4E-02 | 22.7 |
| H101A/H104A | 6.6E+05 | 1.7E-03 | 2.6 |
| H158A/H161A | 6.5E+05 | 4.1E-03 | 6.3 |
| H125A/H126A | 1.8E+05 | 1.6E-03 | 8.9 |
| H153A | 8.9E+05 | 1.8E-03 | 2.1 |
| H154A | 8.0E+05 | 3.3E-03 | 4.1 |
| H155A | 6.7E+05 | 2.0E-03 | 3.0 |
| H98A | 1.0E+06 | 9.6E-03 | 9.5 |
| H100A | 3.7E+05 | 2.8E-03 | 7.5 |
| H64A | 7.8E+05 | 1.2E-03 | 1.6 |
| H117A | 8.6E+05 | 4.1E-03 | 4.7 |
| H111A | 1.9E+06 | 3.5E-03 | 1.9 |
| R159A | 2.7E+05 | 3.3E-02 | 122.4 |
| R86A | N/A | N/A | N.B. |
| S84A | 1.5E+06 | 7.7E-04 | 0.5 |
| D96A | 1.9E+06 | 1.7E-03 | 0.9 |
| R116A | 1.8E+06 | 4.1E-03 | 2.2 |
| S110A | 3.1E+06 | 9.0E-04 | 0.3 |
| E157A | 5.9E+06 | 3.7E-03 | 0.6 |
| F94A | 6.9E+05 | 4.1E-02 | 58.9 |
| Q95A | 2.7E+06 | 8.4E-03 | 3.1 |
| WT | 3.2E+06 | 2.2E-03 | 0.7 |
| VARIANT | $k_a$ ($M^{-1}s^{-1}$) | $k_d$ ($s^{-1}$) | $K_D$ (nM) |
| H98A/H100A/H117A | 4.3E+05 | 3.9E-02 | 90.0 |
| H98A/H100A/H154A/H155A | 2.4E+05 | 3.9E-02 | 165.8 |
| H98A/H100A/H117A/R159A | 1.9E+03 | 8.9E-04 | 480.6 |
| H98A/H100A/H154A/H155A/R159A | N/A | N/A | N.B. |

**Supplementary Table 4. Antibodies and fluorescent reagents used for BRGSF-HIS mouse experiments.**

| mAb and reagent | Clone | Supplier | Reference | Dilution |
|---|---|---|---|---|
| InvivoMab anti-human CD3 | N/A | BioXCell | BX-BE0001-2 | |
| InvivoMab recombinant Flt-3L-Ig | N/A | BioXCell | BE0098 | |
| FcR Blocking Reagent, mouse | | Miltenyi Biotec | 130-092-575 | 1/100 |
| Human BD Fc Block | Fc1 (RUO) | BD Biosciences | 564220 | 1/100 |
| PerCP-Vio700 anti-mouse CD45 | REA737 | Miltenyi Biotec | 130-110-801 | 1/100 |
| APC/Fire750 anti-human CD45 | HI30 | BioLegend | 304062 | 1/100 |
| Vio Bright R720 anti-human CD3 | REA613 | Miltenyi Biotec | 130-127-377 | 1/100 |
| VioBlue anti-human CD4 | REA623 | Miltenyi Biotec | 130-114-725 | 1/100 |
| PE-Vio 615 anti-human CD8 | REA734 | Miltenyi Biotec | 130-110-823 | 1/100 |
| PE-Vio 770 anti-human CD56 | REA196 | Miltenyi Biotec | 130-113-313 | 1/50 |
| Brilliant Violet 711 anti-human CD45RA | HI100 | BioLegend | 304138 | 1/40 |
| APC anti-human CD197 (CCR7) | G043H7 | BioLegend | 353214 | 1/40 |
| Brilliant Violet 605 anti-human CD127 (IL-7R$\alpha$) | A019D5 | BioLegend | 351334 | 1/40 |
| PE anti-human CD25 | REA570 | Miltenyi Biotec | 130-113-286 | 1/100 |
| Brilliant Violet 711 anti-human CD19 | HIB19 | BioLegend | 302246 | 1/100 |
| PE/Cyanine7 anti-human CD11c | Bu15 | BioLegend | 337216 | 1/100 |
| APC anti-human HLA-DR | LN3 | BioLegend | 327022 | 1/100 |
| FITC Mouse Anti-Human CD14 | M5E2 (RUO) | BD Biosciences | 555397 | 1/20 |
| eFluor450 anti-human CD16 | eBIOBC16 | ThermoFisher | 48-0168-42 | 1/50 |
| Brilliant Violet 605 anti-human CD123 | 6H6 | BioLegend | 306026 | 1/50 |
| PE anti-human CD86 Antibody | REA968 | Miltenyi Biotec | 130-116-160 | 1/100 |
| LIVE/DEAD™ Fixable Aqua | | ThermoFisher | L34957 | 1/1000 |

## Supplemental References


1 Findlay, L. *et al.* Endothelial cells co-stimulate peripheral blood mononuclear cell responses to monoclonal antibody TGN1412 in culture. *Cytokine* **55**, 141-151 (2011). https://doi.org:10.1016/j.cyto.2011.03.019

2 Tsukihara, H. *et al.* Efficacy of combination chemotherapy using a novel oral chemotherapeutic agent, TAS-102, together with bevacizumab, cetuximab, or panitumumab on human colorectal cancer xenografts. *Oncol Rep* **33**, 2135-2142 (2015). https://doi.org:10.3892/or.2015.3876

3 Demichev, V., Messner, C. B., Vernardis, S. I., Lilley, K. S. & Ralser, M. DIA-NN: neural networks and interference correction enable deep proteome coverage in high throughput. *Nat Methods* **17**, 41-44 (2020). https://doi.org:10.1038/s41592-019-0638-x

4 Cuklina, J. *et al.* Diagnostics and correction of batch effects in large-scale proteomic studies: a tutorial. *Mol Syst Biol* **17**, e10240 (2021). https://doi.org:10.15252/msb.202110240

5 Breuer, K. *et al.* InnateDB: systems biology of innate immunity and beyond--recent updates and continuing curation. *Nucleic Acids Res* **41**, D1228-1233 (2013). https://doi.org:10.1093/nar/gks1147

6 Shannon, P. *et al.* Cytoscape: a software environment for integrated models of biomolecular interaction networks. *Genome Res* **13**, 2498-2504 (2003). https://doi.org:10.1101/gr.1239303
