# Supplementary material for: VISTA checkpoint inhibition by pH-selective antibody SNS-101 with optimized safety and pharmacokinetic profiles enhances PD-1 response
Source: Nat Commun. 2024 Apr 4;15:2917. doi: 10.1038/s41467-024-47256-x (PMC10995192; doi:10.1038/s41467-024-47256-x)
Supplement: Supplementary file 3 — Description of Additional Supplementary Files [file 41467_2024_47256_MOESM3_ESM.pdf]

Description of Additional Supplementary Files

File Name: Supplementary Data 1
Description: Toxicology report and comprehensive clinical endpoints from NHP studies.
