# Supplementary material for: VISTA checkpoint inhibition by pH-selective antibody SNS-101 with optimized safety and pharmacokinetic profiles enhances PD-1 response
Source: Nat Commun. 2024 Apr 4;15:2917. doi: 10.1038/s41467-024-47256-x (PMC10995192; doi:10.1038/s41467-024-47256-x)
Supplement: Supplementary file 4 — Supplementary Data 1 [file 41467_2024_47256_MOESM4_ESM.pdf]

Appendix 6

Individual Clinical Observations

0 mg/kg /dose Group 1 Sex: Male	Observation Type: Toxicology Observations	Day(s) Relative to Start Date						
		-26 DE	-13 DE	-6 DE	-1 DE	7 DE	14 DE	21 DE
1004	Fur, Thin Cover, Hindlimb, Right	.	.	.	.	X	X	X

X=Present

Appendix 6

Individual Clinical Observations

0<br>mg/kg<br>/dose<br>Group 1<br>Sex: Male	Observation Type: Toxicology Observations	Day(s) Relative to Start Date						
		28<br>DE	30<br>DE	31<br>DE	35<br>DE	42<br>DE	49<br>DE	56<br>DE
1004	Fur, Thin Cover, Hindlimb, Right	X	.	.	X	X	X	X

X=Present

Appendix 6

Individual Clinical Observations

0 mg/kg /dose Group 1 Sex: Male	Observation Type: Toxicology Observations	Day(s) Relative to Start Date						
		63 DE	70 DE	71 DE				
1004	Fur, Thin Cover, Hindlimb, Right	X	X	X				

X=Present

Appendix 6

Individual Clinical Observations

100 mg/kg /dose Group 4 Sex: Male	Observation Type: Toxicology Observations	Day(s) Relative to Start Date						
		-26 DE	-13 DE	-6 DE	-1 DE	7 DE	14 DE	21 DE
4001	Skin, Scab, Forelimb, Right	X	.	.	.	.	.	.
4004	Fur, Thin Cover, Cranium	.	.	.	.	X	X	X

X=Present

Appendix 6

Individual Clinical Observations

100 mg/kg /dose Group 4 Sex: Male	Observation Type: Toxicology Observations	Day(s) Relative to Start Date						
		28 DE	30 DE	31 DE	35 DE	42 DE	49 DE	56 DE
4001	Skin, Scab, Forelimb, Right	.	.	.	.	.	.	.
4004	Fur, Thin Cover, Cranium	.	.	.	.	.	.	.

Appendix 6

Individual Clinical Observations

100 mg/kg /dose Group 4 Sex: Male	Observation Type: Toxicology Observations	Day(s) Relative to Start Date						
		63 DE	70 DE	71 DE				
4001	Skin, Scab, Forelimb, Right	.	.	.				
4004	Fur, Thin Cover, Cranium	.	.	.				

Appendix 6

Individual Clinical Observations

0 mg/kg /dose Group 1 Sex: Female	Observation Type: Toxicology Observations	Day(s) Relative to Start Date						
		-26 DE	-13 DE	-6 DE	-1 DE	7 DE	14 DE	21 DE
1504	Fur, Thin Cover, Hindlimb, Left	.	.	.	.	X	X	X
	Fur, Thin Cover, Hindlimb, Right	.	.	.	.	X	X	X

X=Present

Appendix 6

Individual Clinical Observations

0 mg/kg /dose Group 1 Sex: Female	Observation Type: Toxicology Observations	Day(s) Relative to Start Date						
		28 DE	30 DE	31 DE	35 DE	42 DE	49 DE	56 DE
1504	Fur, Thin Cover, Hindlimb, Left	X	.	.	X	X	X	X
	Fur, Thin Cover, Hindlimb, Right	X	.	.	X	X	X	X

X=Present

Appendix 6

Individual Clinical Observations

0 mg/kg /dose Group 1 Sex: Female	Observation Type: Toxicology Observations	Day(s) Relative to Start Date						
		63 DE	70 DE	71 DE				
1504	Fur, Thin Cover, Hindlimb, Left	X	X	X				
	Fur, Thin Cover, Hindlimb, Right	X	X	X				

X=Present

Appendix 6

Individual Clinical Observations

3<br>mg/kg<br>/dose<br>Group 2<br>Sex: Female	Observation Type: Toxicology Observations	Day(s) Relative to Start Date						
		-26<br>DE	-13<br>DE	-6<br>DE	-1<br>DE	7<br>DE	14<br>DE	21<br>DE
2502	Fur, Thin Cover, Cranium	X	X	X	X	X	X	X
	Fur, Thin Cover, Dorsal Thoracic	.	.	.	.	X	X	X

X=Present

Appendix 6

Individual Clinical Observations

3 mg/kg /dose Group 2 Sex: Female	Observation Type: Toxicology Observations	Day(s) Relative to Start Date						
		28 DE	30 DE	31 DE	35 DE	42 DE	49 DE	56 DE
2502	Fur, Thin Cover, Cranium	.	.	.	.	.	.	.
	Fur, Thin Cover, Dorsal Thoracic	X	X	X	.	.	.	.

X=Present

Appendix 6

Individual Clinical Observations

3<br>mg/kg<br>/dose<br>Group 2<br>Sex: Female	Observation Type: Toxicology Observations	Day(s) Relative to Start Date						
		63<br>DE	70<br>DE	71<br>DE				
2502	Fur, Thin Cover, Cranium	.	.	.				
	Fur, Thin Cover, Dorsal Thoracic	.	.	.				

Appendix 6

Individual Clinical Observations

100 mg/kg /dose Group 4 Sex: Female	Observation Type: Toxicology Observations	Day(s) Relative to Start Date						
		-26 DE	-13 DE	-6 DE	-1 DE	7 DE	14 DE	21 DE
4504	Fur, Thin Cover, Hindlimb, Left	.	.	.	.	X	X	X
	Fur, Thin Cover, Hindlimb, Right	.	.	.	.	X	X	X
	Material Present, Dry, Red, Slight Group Housed	.	.	.	.	.	.	.
4505	Material Present, Dry, Red, Slight Group Housed	.	.	.	.	.	.	.

X=Present

Appendix 6

Individual Clinical Observations

100 mg/kg /dose Group 4 Sex: Female	Observation Type: Toxicology Observations	Day(s) Relative to Start Date						
		28 DE	30 DE	31 DE	35 DE	42 DE	49 DE	56 DE
4504	Fur, Thin Cover, Hindlimb, Left	X	.	.	X	.	.	.
	Fur, Thin Cover, Hindlimb, Right	X	.	.	X	.	.	.
	Material Present, Dry, Red, Slight Group Housed	.	.	.	X	.	.	.
4505	Material Present, Dry, Red, Slight Group Housed	.	.	.	X	.	.	.

X=Present

Appendix 6

Individual Clinical Observations

100 mg/kg /dose Group 4 Sex: Female	Observation Type: Toxicology Observations	Day(s) Relative to Start Date						
		63 DE	70 DE	71 DE				
4504	Fur, Thin Cover, Hindlimb, Left	.	.	.				
	Fur, Thin Cover, Hindlimb, Right	.	.	.				
	Material Present, Dry, Red, Slight Group Housed	.	.	.				
4505	Material Present, Dry, Red, Slight Group Housed	.	.	.				

Appendix 6

Individual Clinical Observations

0 mg/kg /dose Group 1 Sex: Male	Observation Type: SIRT/Cage Side Observations	Day(s) Relative to Start Date						
		-21 Un1	1 UDu1	3 CSO	15 AM_S	50 CSO	65 CSO	
1004	Feces, Abnormal Consistency, Liquid, Slight Group Housed	.	.	X	.	.	.	
1005	Feces, Abnormal Consistency, Liquid, Slight Group Housed	.	.	X	.	.	.	

X=Present

Appendix 6

Individual Clinical Observations

0<br>mg/kg<br>/dose<br>Group 1<br>Sex: Female	Observation Type: SIRT/Cage Side Observations	Day(s) Relative to Start Date						
		-21<br>Un1	1<br>UDu1	3<br>CSO	15<br>AM_S	50<br>CSO	65<br>CSO	
1504	Reduced Appetite, Group Housed	.	.	.	.	.	X	
1505	Reduced Appetite, Group Housed	.	.	.	.	.	X	

X=Present

Appendix 6

Individual Clinical Observations

3 mg/kg /dose Group 2 Sex: Female	Observation Type: SIRT/Cage Side Observations	Day(s) Relative to Start Date						
		-21 Un1	1 UDu1	3 CSO	15 AM_S	50 CSO	65 CSO	
2501	Vomitus, Foamy, White, Slight	.	X	.	.	.	.	

X=Present

Appendix 6

Individual Clinical Observations

100 mg/kg /dose Group 4 Sex: Female	Observation Type: SIRT/Cage Side Observations	Day(s) Relative to Start Date						
		-21 Un1	1 UDu1	3 CSO	15 AM_S	50 CSO	65 CSO	
4502	Fur, Staining, Urogenital, Red	X	.	.	.	.	.	
	Material Present, Dry, Red, Moderate	X	.	.	.	.	.	
4504	Reduced Appetite, Group Housed	.	.	.	X	X	.	
4505	Reduced Appetite, Group Housed	.	.	.	X	X	.	

X=Present

Appendix 6

Individual Clinical Observations

0 mg/kg /dose Group 1 Sex: Male	Observation Type: Physical Exam / Vet	Day(s) Relative to Start Date						
		-25 DE						
1004	Fur, Thin Cover, Hindlimb, Left	X						
	Fur, Thin Cover, Hindlimb, Right	X						

X=Present

Appendix 6

Individual Clinical Observations

10 mg/kg /dose Group 3 Sex: Male	Observation Type: Physical Exam / Vet	Day(s) Relative to Start Date						
		-25 DE						
3002	Fur, Thin Cover, Hindlimb, Left	X						
	Fur, Thin Cover, Hindlimb, Right	X						

X=Present

Appendix 6

Individual Clinical Observations

100 mg/kg /dose Group 4 Sex: Male	Observation Type: Physical Exam / Vet	Day(s) Relative to Start Date						
		-25 DE						
4003	Fur, Thin Cover, Hindlimb, Left	X						
	Fur, Thin Cover, Hindlimb, Right	X						
4004	Skin - Laceration, Forelimb, Right, Slight	X						

X=Present

Appendix 6

Individual Clinical Observations

0<br>mg/kg<br>/dose<br>Group 1<br>Sex: Female	Observation Type: Physical Exam / Vet	Day(s) Relative to Start Date<br>-25<br>DE						
1502	Fur, Thin Cover, Dorsal Aspect Generalized	X						
	Fur, Thin Cover, Hindlimb, Left	X						
	Fur, Thin Cover, Hindlimb, Right	X						
1504	Fur, Thin Cover, Dorsal Aspect Generalized	X						
	Material Present, Liquid, Red, Moderate	X						
1505	Fur, Thin Cover, Hindlimb, Left	X						
	Fur, Thin Cover, Hindlimb, Right	X						

X=Present

Appendix 6

Individual Clinical Observations

3 mg/kg /dose Group 2 Sex: Female	Observation Type: Physical Exam / Vet	Day(s) Relative to Start Date						
		-25 DE						
2502	Fur, Thin Cover, Cranium	X						
	Fur, Thin Cover, Dorsal Aspect Generalized	X						
	Fur, Thin Cover, Hindlimb, Left	X						
	Fur, Thin Cover, Hindlimb, Right	X						

X=Present

Appendix 6

Individual Clinical Observations

10 mg/kg /dose Group 3 Sex: Female	Observation Type: Physical Exam / Vet	Day(s) Relative to Start Date						
		-25 DE						
3501	Fur, Thin Cover, Dorsal Aspect Generalized	X						
	Fur, Thin Cover, Hindlimb, Left	X						
	Fur, Thin Cover, Hindlimb, Right	X						

X=Present

Appendix 6

Individual Clinical Observations

100 mg/kg /dose Group 4 Sex: Female	Observation Type: Physical Exam / Vet	Day(s) Relative to Start Date						
		-25 DE						
4502	Fur, Thin Cover, Dorsal Aspect Generalized	X						

X=Present

Appendix 7

Individual Body Weights

Sex: Male   Bodyweight (kg)

0 mg/kg /dose Group 1	Day(s) Relative to Start Date						
	-25	-13	-6	-1	7	14	21
1001	2.2	2.4	2.4	2.3	2.4	2.5	2.6
1002	4.0	4.1	4.1	4.0	4.1	4.3	4.4
1003	2.8	3.0	3.0	3.0	3.1	3.1	3.2
1004	2.7	2.8	2.8	2.8	2.9	3.1	3.1
1005	3.0	3.2	3.2	3.2	3.3	3.4	3.6
Mean	2.94	3.10	3.10	3.06	3.16	3.28	3.38
SD	0.66	0.63	0.63	0.62	0.62	0.66	0.67
N	5	5	5	5	5	5	5

Appendix 7

Individual Body Weights

Sex: Male   Bodyweight (kg)

0 mg/kg /dose Group 1	Day(s) Relative to Start Date						
	28	30	31	35	42	49	56
1001	2.4	2.4	2.4	-	-	-	-
1002	4.3	4.4	4.3	-	-	-	-
1003	3.0	3.1	3.0	-	-	-	-
1004	3.0	3.0	-	3.1	3.0	3.2	3.0
1005	3.4	3.4	-	3.5	3.6	3.8	3.6
Mean	3.22	3.26	3.23	3.30	3.30	3.50	3.30
SD	0.70	0.73	0.97	0.28	0.42	0.42	0.42
N	5	5	3	2	2	2	2

Appendix 7

Individual Body Weights

Sex: Male   Bodyweight (kg)

0 mg/kg /dose Group 1	Day(s) Relative to Start Date		
	63	70	71
1001	-	-	-
1002	-	-	-
1003	-	-	-
1004	3.1	3.2	3.1
1005	3.6	3.7	3.6
Mean	3.35	3.45	3.35
SD	0.35	0.35	0.35
N	2	2	2

Appendix 7

Individual Body Weights

Sex: Male   Bodyweight (kg)

3 mg/kg /dose Group 2	Day(s) Relative to Start Date						
	-25	-13	-6	-1	7	14	21
2001	3.2	3.3	3.3	3.3	3.4	3.5	3.6
2002	2.7	2.8	2.9	2.8	2.8	2.9	3.1
2003	2.7	2.8	2.9	2.8	2.9	3.0	3.1
Mean	2.87	2.97	3.03	2.97	3.03	3.13	3.27
SD	0.29	0.29	0.23	0.29	0.32	0.32	0.29
N	3	3	3	3	3	3	3
%Diff	-2.49	-4.30	-2.15	-3.05	-4.01	-4.47	-3.35

Appendix 7

Individual Body Weights

Sex: Male   Bodyweight (kg)

3 mg/kg /dose Group 2	Day(s) Relative to Start Date		
	28	30	31
2001	3.4	3.4	3.4
2002	2.9	2.9	2.7
2003	2.9	2.9	2.9
Mean	3.07	3.07	3.00
SD	0.29	0.29	0.36
N	3	3	3
%Diff	-4.76	-5.93	-7.22

Appendix 7

Individual Body Weights

Sex: Male   Bodyweight (kg)

10 mg/kg /dose Group 3	Day(s) Relative to Start Date						
	-25	-13	-6	-1	7	14	21
3001	2.9	3.0	3.1	3.0	3.0	3.2	3.2
3002	2.6	2.8	2.8	2.8	2.9	3.0	3.1
3003	3.1	3.3	3.3	3.1	3.1	3.3	3.3
Mean	2.87	3.03	3.07	2.97	3.00	3.17	3.20
SD	0.25	0.25	0.25	0.15	0.10	0.15	0.10
N	3	3	3	3	3	3	3
%Diff	-2.49	-2.15	-1.08	-3.05	-5.06	-3.46	-5.33

Appendix 7

Individual Body Weights

Sex: Male   Bodyweight (kg)

10 mg/kg /dose Group 3	Day(s) Relative to Start Date		
	28	30	31
3001	3.0	3.1	3.0
3002	3.0	3.0	2.9
3003	3.2	3.2	3.1
Mean	3.07	3.10	3.00
SD	0.12	0.10	0.10
N	3	3	3
%Diff	-4.76	-4.91	-7.22

Appendix 7

Individual Body Weights

Sex: Male Bodyweight (kg)

100 mg/kg /dose Group 4	Day(s) Relative to Start Date						
	-25	-13	-6	-1	7	14	21
4001	2.9	3.0	3.0	3.0	3.0	3.1	3.2
4002	2.9	3.1	3.3	3.2	3.3	3.4	3.5
4003	2.3	2.4	2.4	2.4	2.5	2.6	2.7
4004	2.2	2.4	2.4	2.3	2.4	2.5	2.5
4005	3.2	3.5	3.5	3.4	3.5	3.6	3.8
Mean	2.70	2.88	2.92	2.86	2.94	3.04	3.14
SD	0.43	0.48	0.51	0.49	0.48	0.48	0.54
N	5	5	5	5	5	5	5
%Diff	-8.16	-7.10	-5.81	-6.54	-6.96	-7.32	-7.10

Appendix 7

Individual Body Weights

Sex: Male   Bodyweight (kg)

100 mg/kg /dose Group 4	Day(s) Relative to Start Date						
	28	30	31	35	42	49	56
4001	3.0	3.1	3.0	-	-	-	-
4002	3.3	3.4	3.2	-	-	-	-
4003	2.5	2.5	2.5	-	-	-	-
4004	2.4	2.4	-	2.4	2.5	2.4	2.5
4005	3.6	3.6	-	3.7	3.6	3.7	3.6
Mean	2.96	3.00	2.90	3.05	3.05	3.05	3.05
SD	0.51	0.53	0.36	0.92	0.78	0.92	0.78
N	5	5	3	2	2	2	2
%Diff	-8.07	-7.98	-10.31	-7.58	-7.58	-12.86	-7.58

Appendix 7

Individual Body Weights

Sex: Male   Bodyweight (kg)

100 mg/kg /dose Group 4	Day(s) Relative to Start Date		
	63	70	71
4001	-	-	-
4002	-	-	-
4003	-	-	-
4004	2.4	2.5	2.4
4005	3.7	3.7	3.6
Mean	3.05	3.10	3.00
SD	0.92	0.85	0.85
N	2	2	2
%Diff	-8.96	-10.14	-10.45

Appendix 7

Individual Body Weights

Sex: Female   Bodyweight (kg)

0 mg/kg /dose Group 1	Day(s) Relative to Start Date						
	-25	-13	-6	-1	7	14	21
1501	2.8	3.0	3.1	2.9	3.0	3.2	3.3
1502	3.2	3.3	3.5	3.3	3.4	3.5	3.5
1503	2.4	2.5	2.6	2.5	2.7	2.8	2.9
1504	2.3	2.6	2.7	2.6	2.6	2.8	3.0
1505	2.5	2.7	2.7	2.6	2.7	2.8	3.0
Mean	2.64	2.82	2.92	2.78	2.88	3.02	3.14
SD	0.36	0.33	0.38	0.33	0.33	0.32	0.25
N	5	5	5	5	5	5	5

Appendix 7

Individual Body Weights

Sex: Female Bodyweight (kg)

0 mg/kg /dose Group 1	Day(s) Relative to Start Date						
	28	30	31	35	42	49	56
1501	3.1	3.1	3.0	-	-	-	-
1502	3.3	3.3	3.3	-	-	-	-
1503	2.6	2.7	2.6	-	-	-	-
1504	2.8	2.8	-	2.8	2.7	2.7	2.7
1505	2.8	2.8	-	2.9	2.9	2.9	2.8
Mean	2.92	2.94	2.97	2.85	2.80	2.80	2.75
SD	0.28	0.25	0.35	0.07	0.14	0.14	0.07
N	5	5	3	2	2	2	2

Appendix 7

Individual Body Weights

Sex: Female   Bodyweight (kg)

0 mg/kg /dose Group 1	Day(s) Relative to Start Date		
	63	70	71
1501	-	-	-
1502	-	-	-
1503	-	-	-
1504	2.7	2.8	2.7
1505	2.7	2.8	2.7
Mean	2.70	2.80	2.70
SD	0.00	0.00	0.00
N	2	2	2

Appendix 7

Individual Body Weights

Sex: Female   Bodyweight (kg)

3 mg/kg /dose Group 2	Day(s) Relative to Start Date						
	-25	-13	-6	-1	7	14	21
2501	2.6	2.7	2.8	2.7	2.9	2.9	2.9
2502	2.5	2.5	2.6	2.5	2.7	2.7	2.9
2503	2.7	3.0	2.9	2.9	2.9	3.0	3.2
Mean	2.60	2.73	2.77	2.70	2.83	2.87	3.00
SD	0.10	0.25	0.15	0.20	0.12	0.15	0.17
N	3	3	3	3	3	3	3
%Diff	-1.52	-3.07	-5.25	-2.88	-1.62	-5.08	-4.46

Appendix 7

Individual Body Weights

Sex: Female   Bodyweight (kg)

3 mg/kg /dose Group 2	Day(s) Relative to Start Date		
	28	30	31
2501	2.8	2.9	2.8
2502	2.7	2.7	2.6
2503	3.0	3.0	2.9
Mean	2.83	2.87	2.77
SD	0.15	0.15	0.15
N	3	3	3
%Diff	-2.97	-2.49	-6.74

Appendix 7

Individual Body Weights

Sex: Female   Bodyweight (kg)

10 mg/kg /dose Group 3	Day(s) Relative to Start Date						
	-25	-13	-6	-1	7	14	21
3501	2.5	2.6	2.7	2.6	2.7	2.7	2.8
3502	2.7	3.0	3.1	3.0	3.0	3.0	3.0
3503	2.5	2.6	2.6	2.6	2.7	2.9	2.9
Mean	2.57	2.73	2.80	2.73	2.80	2.87	2.90
SD	0.12	0.23	0.26	0.23	0.17	0.15	0.10
N	3	3	3	3	3	3	3
%Diff	-2.78	-3.07	-4.11	-1.68	-2.78	-5.08	-7.64

Appendix 7

Individual Body Weights

Sex: Female Bodyweight (kg)

10 mg/kg /dose Group 3	Day(s) Relative to Start Date		
	28	30	31
3501	2.7	2.6	2.6
3502	2.9	2.9	2.9
3503	2.9	2.8	2.7
Mean	2.83	2.77	2.73
SD	0.12	0.15	0.15
N	3	3	3
%Diff	-2.97	-5.90	-7.87

Appendix 7

Individual Body Weights

Sex: Female   Bodyweight (kg)

100 mg/kg /dose Group 4	Day(s) Relative to Start Date						
	-25	-13	-6	-1	7	14	21
4501	2.5	2.7	2.6	2.5	2.6	2.7	2.7
4502	3.0	3.3	3.4	3.4	3.4	3.5	3.7
4503	2.5	2.7	2.7	2.7	2.7	2.8	2.9
4504	2.6	2.7	2.7	2.7	2.8	2.8	2.8
4505	2.7	2.9	2.9	2.9	3.0	3.0	3.0
Mean	2.66	2.86	2.86	2.84	2.90	2.96	3.02
SD	0.21	0.26	0.32	0.34	0.32	0.32	0.40
N	5	5	5	5	5	5	5
%Diff	0.76	1.42	-2.05	2.16	0.69	-1.99	-3.82

Appendix 7

Individual Body Weights

Sex: Female   Bodyweight (kg)

100 mg/kg /dose Group 4	Day(s) Relative to Start Date						
	28	30	31	35	42	49	56
4501	2.6	2.6	2.5	-	-	-	-
4502	3.5	3.5	3.5	-	-	-	-
4503	2.8	2.8	2.7	-	-	-	-
4504	2.7	2.7	-	2.8	2.7	2.7	2.6
4505	2.9	2.8	-	3.0	2.9	2.8	2.9
Mean	2.90	2.88	2.90	2.90	2.80	2.75	2.75
SD	0.35	0.36	0.53	0.14	0.14	0.07	0.21
N	5	5	3	2	2	2	2
%Diff	-0.68	-2.04	-2.25	1.75	0.00	-1.79	0.00

Appendix 7

Individual Body Weights

Sex: Female   Bodyweight (kg)

100 mg/kg /dose Group 4	Day(s) Relative to Start Date		
	63	70	71
4501	-	-	-
4502	-	-	-
4503	-	-	-
4504	2.7	2.7	2.7
4505	2.9	2.9	2.9
Mean	2.80	2.80	2.80
SD	0.14	0.14	0.14
N	2	2	2
%Diff	3.70	0.00	3.70

Appendix 8

Individual Body Weight Gains (kg)

Sex: Female   Bodyweight Gain (Interval)

0 mg/kg /dose Group 1	Day(s) Relative to Start Date						
	-25 → -13	-13 → -6	-6 → -1	-1 → 7	7 → 14	14 → 21	21 → 28
1501	0.2	0.1	-0.2	0.1	0.2	0.1	-0.2
1502	0.1	0.2	-0.2	0.1	0.1	0.0	-0.2
1503	0.1	0.1	-0.1	0.2	0.1	0.1	-0.3
1504	0.3	0.1	-0.1	0.0	0.2	0.2	-0.2
1505	0.2	0.0	-0.1	0.1	0.1	0.2	-0.2
Mean	0.18	0.10	-0.14	0.10	0.14	0.12	-0.22
SD	0.08	0.07	0.05	0.07	0.05	0.08	0.04
N	5	5	5	5	5	5	5

Appendix 8

Individual Body Weight Gains (kg)

Sex: Female   Bodyweight Gain (Interval)

0 mg/kg /dose Group 1	Day(s) Relative to Start Date						
	28 → 30	30 → 31	30 → 35	35 → 42	42 → 49	49 → 56	56 → 63
1501	0.0	-0.1	-	-	-	-	-
1502	0.0	0.0	-	-	-	-	-
1503	0.1	-0.1	-	-	-	-	-
1504	0.0	-	0.0	-0.1	0.0	0.0	0.0
1505	0.0	-	0.1	0.0	0.0	-0.1	-0.1
Mean	0.02	-0.07	0.05	-0.05	0.00	-0.05	-0.05
SD	0.04	0.06	0.07	0.07	0.00	0.07	0.07
N	5	3	2	2	2	2	2

Appendix 8

Individual Body Weight Gains (kg)

Sex: Female   Bodyweight Gain (Interval)

0 mg/kg /dose Group 1	Day(s) Relative to Start Date	
	63 → 70	70 → 71
1501	-	-
1502	-	-
1503	-	-
1504	0.1	-0.1
1505	0.1	-0.1
Mean	0.10	-0.10
SD	0.00	0.00
N	2	2

Appendix 8

Individual Body Weight Gains (kg)

Sex: Female   Bodyweight Gain (Interval)

3 mg/kg /dose Group 2	Day(s) Relative to Start Date						
	-25 → -13	-13 → -6	-6 → -1	-1 → 7	7 → 14	14 → 21	21 → 28
2501	0.1	0.1	-0.1	0.2	0.0	0.0	-0.1
2502	0.0	0.1	-0.1	0.2	0.0	0.2	-0.2
2503	0.3	-0.1	0.0	0.0	0.1	0.2	-0.2
Mean	0.13	0.03	-0.07	0.13	0.03	0.13	-0.17
SD	0.15	0.12	0.06	0.12	0.06	0.12	0.06
N	3	3	3	3	3	3	3

Appendix 8

Individual Body Weight Gains (kg)

Sex: Female   Bodyweight Gain (Interval)

3 mg/kg /dose Group 2	Day(s) Relative to Start Date	
	28 → 30	30 → 31
2501	0.1	-0.1
2502	0.0	-0.1
2503	0.0	-0.1
Mean	0.03	-0.10
SD	0.06	0.00
N	3	3

Appendix 8

Individual Body Weight Gains (kg)

Sex: Female Bodyweight Gain (Interval)

10 mg/kg /dose Group 3	Day(s) Relative to Start Date						
	-25 → -13	-13 → -6	-6 → -1	-1 → 7	7 → 14	14 → 21	21 → 28
3501	0.1	0.1	-0.1	0.1	0.0	0.1	-0.1
3502	0.3	0.1	-0.1	0.0	0.0	0.0	-0.1
3503	0.1	0.0	0.0	0.1	0.2	0.0	0.0
Mean	0.17	0.07	-0.07	0.07	0.07	0.03	-0.07
SD	0.12	0.06	0.06	0.06	0.12	0.06	0.06
N	3	3	3	3	3	3	3

Appendix 8

Individual Body Weight Gains (kg)

Sex: Female Bodyweight Gain (Interval)

10 mg/kg /dose Group 3	Day(s) Relative to Start Date	
	28 → 30	30 → 31
3501	-0.1	0.0
3502	0.0	0.0
3503	-0.1	-0.1
Mean	-0.07	-0.03
SD	0.06	0.06
N	3	3

Appendix 8

Individual Body Weight Gains (kg)

Sex: Female   Bodyweight Gain (Interval)

100 mg/kg /dose Group 4	Day(s) Relative to Start Date						
	-25 → -13	-13 → -6	-6 → -1	-1 → 7	7 → 14	14 → 21	21 → 28
4501	0.2	-0.1	-0.1	0.1	0.1	0.0	-0.1
4502	0.3	0.1	0.0	0.0	0.1	0.2	-0.2
4503	0.2	0.0	0.0	0.0	0.1	0.1	-0.1
4504	0.1	0.0	0.0	0.1	0.0	0.0	-0.1
4505	0.2	0.0	0.0	0.1	0.0	0.0	-0.1
Mean	0.20	0.00	-0.02	0.06	0.06	0.06	-0.12
SD	0.07	0.07	0.04	0.05	0.05	0.09	0.04
N	5	5	5	5	5	5	5

Appendix 8

Individual Body Weight Gains (kg)

Sex: Female Bodyweight Gain (Interval)

100 mg/kg /dose Group 4	Day(s) Relative to Start Date						
	28 → 30	30 → 31	30 → 35	35 → 42	42 → 49	49 → 56	56 → 63
4501	0.0	-0.1	-	-	-	-	-
4502	0.0	0.0	-	-	-	-	-
4503	0.0	-0.1	-	-	-	-	-
4504	0.0	-	0.1	-0.1	0.0	-0.1	0.1
4505	-0.1	-	0.2	-0.1	-0.1	0.1	0.0
Mean	-0.02	-0.07	0.15	-0.10	-0.05	0.00	0.05
SD	0.04	0.06	0.07	0.00	0.07	0.14	0.07
N	5	3	2	2	2	2	2

Appendix 8

Individual Body Weight Gains (kg)

Sex: Female   Bodyweight Gain (Interval)

100 mg/kg /dose Group 4	Day(s) Relative to Start Date	
	63 → 70	70 → 71
4501	-	-
4502	-	-
4503	-	-
4504	0.0	0.0
4505	0.0	0.0
Mean	0.00	0.00
SD	0.00	0.00
N	2	2

Appendix 9

Individual Vital Signs

Sex: Male   Respiratory Rate (breaths/min)

0 mg/kg /dose Group 1	Day(s) Relative to Start Date	
	-13	24
1001	42	42
1002	48	48
1003	36	30
1004	36	42
1005	42	36
Mean	40.8	39.6
SD	5.0	6.8
N	5	5

Appendix 9

Individual Vital Signs

Sex: Male   Respiratory Rate (breaths/min)

3 mg/kg /dose Group 2	Day(s) Relative to Start Date	
	-13	24
2001	48	54
2002	54	48
2003	42	48
Mean	48.0	50.0
SD	6.0	3.5
N	3	3
%Diff	17.6	26.3

Appendix 9

Individual Vital Signs

Sex: Male   Respiratory Rate (breaths/min)

10 mg/kg /dose Group 3	Day(s) Relative to Start Date	
	-13	24
3001	54	42
3002	42	36
3003	48	48
Mean	48.0	42.0
SD	6.0	6.0
N	3	3
%Diff	17.6	6.1

Appendix 9

Individual Vital Signs

Sex: Male Respiratory Rate (breaths/min)

100 mg/kg /dose Group 4	Day(s) Relative to Start Date	
	-13	24
4001	48	48
4002	48	42
4003	42	36
4004	36	60
4005	48	36
Mean	44.4	44.4
SD	5.4	10.0
N	5	5
%Diff	8.8	12.1

Appendix 9

Individual Vital Signs

Sex: Female Respiratory Rate (breaths/min)

0 mg/kg /dose Group 1	Day(s) Relative to Start Date	
	-13	24
1501	42	54
1502	36	42
1503	42	48
1504	42	42
1505	48	42
Mean	42.0	45.6
SD	4.2	5.4
N	5	5

Appendix 9

Individual Vital Signs

Sex: Female   Respiratory Rate (breaths/min)

3 mg/kg /dose Group 2	Day(s) Relative to Start Date	
	-13	24
2501	48	42
2502	48	48
2503	42	48
Mean	46.0	46.0
SD	3.5	3.5
N	3	3
%Diff	9.5	0.9

Appendix 9

Individual Vital Signs

Sex: Female Respiratory Rate (breaths/min)

10 mg/kg /dose Group 3	Day(s) Relative to Start Date	
	-13	24
3501	36	48
3502	42	48
3503	42	42
Mean	40.0	46.0
SD	3.5	3.5
N	3	3
%Diff	-4.8	0.9

Appendix 9

Individual Vital Signs

Sex: Female   Respiratory Rate (breaths/min)

100 mg/kg /dose Group 4	Day(s) Relative to Start Date	
	-13	24
4501	36	42
4502	36	48
4503	36	42
4504	48	36
4505	42	36
Mean	39.6	40.8
SD	5.4	5.0
N	5	5
%Diff	-5.7	-10.5

Appendix 9

Individual Vital Signs

Sex: Male   Systolic Pressure (mmHg)

0 mg/kg /dose Group 1	Day(s) Relative to Start Date	
	-13	24
1001	158	131
1002	189 [a]	140
1003	160	134
1004	128	160
1005	134	158
Mean	153.8	144.6
SD	24.3	13.6
N	5	5

[a] [RC:value out of range recollect 1 time]

Appendix 9

Individual Vital Signs

Sex: Male   Systolic Pressure (mmHg)

3 mg/kg /dose Group 2	Day(s) Relative to Start Date	
	-13	24
2001	123	160
2002	153	160
2003	128	137
Mean	134.7	152.3
SD	16.1	13.3
N	3	3
%Diff	-12.4	5.3

Appendix 9

Individual Vital Signs

Sex: Male   Systolic Pressure (mmHg)

10 mg/kg /dose Group 3	Day(s) Relative to Start Date	
	-13	24
3001	152	152
3002	145	104
3003	155	131
Mean	150.7	129.0
SD	5.1	24.1
N	3	3
%Diff	-2.0	-10.8

Appendix 9

Individual Vital Signs

Sex: Male Systolic Pressure (mmHg)

100 mg/kg /dose Group 4	Day(s) Relative to Start Date	
	-13	24
4001	136	120
4002	148	160
4003	135	155
4004	140	160
4005	149	118
Mean	141.6	142.6
SD	6.6	21.7
N	5	5
%Diff	-7.9	-1.4

Appendix 9

Individual Vital Signs

Sex: Female   Systolic Pressure (mmHg)

0 mg/kg /dose Group 1	Day(s) Relative to Start Date	
	-13	24
1501	135	143
1502	116	138
1503	126	142
1504	122	148
1505	157	147
Mean	131.2	143.6
SD	16.0	4.0
N	5	5

Appendix 9

Individual Vital Signs

Sex: Female   Systolic Pressure (mmHg)

3 mg/kg /dose Group 2	Day(s) Relative to Start Date	
	-13	24
2501	143	113
2502	160	130
2503	130	62
Mean	144.3	101.7
SD	15.0	35.4
N	3	3
%Diff	10.0	-29.2

Appendix 9

Individual Vital Signs

Sex: Female   Systolic Pressure (mmHg)

10 mg/kg /dose Group 3	Day(s) Relative to Start Date	
	-13	24
3501	149	129
3502	129	142
3503	139	140
Mean	139.0	137.0
SD	10.0	7.0
N	3	3
%Diff	5.9	-4.6

Appendix 9

Individual Vital Signs

Sex: Female   Systolic Pressure (mmHg)

100 mg/kg /dose Group 4	Day(s) Relative to Start Date	
	-13	24
4501	146	131
4502	134	126
4503	139	123
4504	145	138
4505	138	134
Mean	140.4	130.4
SD	5.0	6.0
N	5	5
%Diff	7.0	-9.2

Appendix 9

Individual Vital Signs

Sex: Male   Diastolic Pressure (mmHg)

0 mg/kg /dose Group 1	Day(s) Relative to Start Date	
	-13	24
1001	91	94
1002	99	107
1003	110	104
1004	84	100
1005	100	110
Mean	96.8	103.0
SD	9.8	6.2
N	5	5

Appendix 9

Individual Vital Signs

Sex: Male   Diastolic Pressure (mmHg)

3 mg/kg /dose Group 2	Day(s) Relative to Start Date	
	-13	24
2001	83	110
2002	110	90
2003	74	95
Mean	89.0	98.3
SD	18.7	10.4
N	3	3
%Diff	-8.1	-4.5

Appendix 9

Individual Vital Signs

Sex: Male Diastolic Pressure (mmHg)

10 mg/kg /dose Group 3	Day(s) Relative to Start Date	
	-13	24
3001	82	91
3002	58	58
3003	88	73
Mean	76.0	74.0
SD	15.9	16.5
N	3	3
%Diff	-21.5	-28.2

Appendix 9

Individual Vital Signs

Sex: Male   Diastolic Pressure (mmHg)

100 mg/kg /dose Group 4	Day(s) Relative to Start Date	
	-13	24
4001	100	73
4002	102	110
4003	69	85
4004	96	110
4005	102	64
Mean	93.8	88.4
SD	14.1	21.1
N	5	5
%Diff	-3.1	-14.2

Appendix 9

Individual Vital Signs

Sex: Female   Diastolic Pressure (mmHg)

0 mg/kg /dose Group 1	Day(s) Relative to Start Date	
	-13	24
1501	96	102
1502	71	110
1503	70	71
1504	74	110
1505	101	106
Mean	82.4	99.8
SD	14.9	16.4
N	5	5

Appendix 9

Individual Vital Signs

Sex: Female Diastolic Pressure (mmHg)

3 mg/kg /dose Group 2	Day(s) Relative to Start Date	
	-13	24
2501	110	86
2502	61	77
2503	63	35
Mean	78.0	66.0
SD	27.7	27.2
N	3	3
%Diff	-5.3	-33.9

Appendix 9

Individual Vital Signs

Sex: Female   Diastolic Pressure (mmHg)

10 mg/kg /dose Group 3	Day(s) Relative to Start Date	
	-13	24
3501	86	92
3502	77	99
3503	58	92
Mean	73.7	94.3
SD	14.3	4.0
N	3	3
%Diff	-10.6	-5.5

Appendix 9

Individual Vital Signs

Sex: Female   Diastolic Pressure (mmHg)

100 mg/kg /dose Group 4	Day(s) Relative to Start Date	
	-13	24
4501	81	71
4502	92	81
4503	70	68
4504	102	96
4505	86	74
Mean	86.2	78.0
SD	12.0	11.2
N	5	5
%Diff	4.6	-21.8

Appendix 9

Individual Vital Signs

Sex: Male   Mean Arterial Pressure (mmHg)

0 mg/kg /dose Group 1	Day(s) Relative to Start Date	
	-13	24
1001	113	110
1002	131 [a]	118
1003	125	116
1004	103	125
1005	107	125
Mean	115.8	118.8
SD	11.9	6.4
N	5	5

[a] [RC:Value out of range recollected 1 time]

Appendix 9

Individual Vital Signs

Sex: Male   Mean Arterial Pressure (mmHg)

3 mg/kg /dose Group 2	Day(s) Relative to Start Date	
	-13	24
2001	99	125
2002	125	109
2003	94	114
Mean	106.0	116.0
SD	16.6	8.2
N	3	3
%Diff	-8.5	-2.4

Appendix 9

Individual Vital Signs

Sex: Male   Mean Arterial Pressure (mmHg)

10 mg/kg /dose Group 3	Day(s) Relative to Start Date	
	-13	24
3001	112	114
3002	97	75
3003	113	99
Mean	107.3	96.0
SD	9.0	19.7
N	3	3
%Diff	-7.3	-19.2

Appendix 9

Individual Vital Signs

Sex: Male   Mean Arterial Pressure (mmHg)

100 mg/kg /dose Group 4	Day(s) Relative to Start Date	
	-13	24
4001	105	90
4002	122	125
4003	96	106
4004	109	125
4005	112	86
Mean	108.8	106.4
SD	9.5	18.6
N	5	5
%Diff	-6.0	-10.4

Appendix 9

Individual Vital Signs

Sex: Female Mean Arterial Pressure (mmHg)

0 mg/kg /dose Group 1	Day(s) Relative to Start Date	
	-13	24
1501	109	117
1502	90	121
1503	87	97
1504	93	125
1505	123	119
Mean	100.4	115.8
SD	15.2	10.9
N	5	5

Appendix 9

Individual Vital Signs

Sex: Female Mean Arterial Pressure (mmHg)

3 mg/kg /dose Group 2	Day(s) Relative to Start Date	
	-13	24
2501	125	96
2502	104	101
2503	90	43
Mean	106.3	80.0
SD	17.6	32.1
N	3	3
%Diff	5.9	-30.9

Appendix 9

Individual Vital Signs

Sex: Female   Mean Arterial Pressure (mmHg)

10 mg/kg /dose Group 3	Day(s) Relative to Start Date	
	-13	24
3501	106	105
3502	97	115
3503	93	112
Mean	98.7	110.7
SD	6.7	5.1
N	3	3
%Diff	-1.7	-4.4

Appendix 9

Individual Vital Signs

Sex: Female   Mean Arterial Pressure (mmHg)

100 mg/kg /dose Group 4	Day(s) Relative to Start Date	
	-13	24
4501	103	89
4502	104	100
4503	95	88
4504	118	106
4505	106	99
Mean	105.2	96.4
SD	8.3	7.7
N	5	5
%Diff	4.8	-16.8

Appendix 10

Individual Hematology Values

Sex: Male

0 mg/kg /dose Group 1	Day(s) Relative to Start Date	Reporting Hematology				
		WBC (10^3/uL)	NEUT (10^3/uL)	LYMPH (10^3/uL)	MONO (10^3/uL)	EOS (10^3/uL)
1001	-14	5.84	2.34	3.25	0.16	0.02
	31	6.97	3.42	3.25	0.18	0.02
1002	-14	12.61	5.81	6.08	0.41	0.18
	31	15.81	7.94	7.09	0.37	0.20
1003	-14	10.07	4.98	4.65	0.26	0.07
	31	13.87	7.81	5.38	0.32	0.19
1004	-14	10.71	5.92	3.98	0.40	0.23
	31	12.19	5.36	5.70	0.49	0.36
	71	10.18	3.70	5.42	0.50	0.40
1005	-14	7.92	2.39	4.96	0.39	0.09
	31	9.89	4.82	4.40	0.38	0.11
	71	7.31	1.29	5.46	0.35	0.08

Appendix 10

Individual Hematology Values

Sex: Male

0 mg/kg /dose Group 1	Day(s) Relative to Start Date	Reporting Hematology BASO (10^3/uL)	LUC (10^3/uL)	RBC (10^6/uL)	HGB (g/dL)	HCT (%)
1001	-14	0.03	0.05	7.17	14.1	48.2
	31	0.05	0.05	6.37	12.8	44.0
1002	-14	0.05	0.09	7.56	15.7	51.3
	31	0.08	0.13	6.96	14.7	46.8
1003	-14	0.02	0.08	6.83	13.1	44.4
	31	0.07	0.10	6.49	12.4	42.4
1004	-14	0.05	0.13	6.38	12.8	42.9
	31	0.08	0.20	5.83	11.9	40.5
	71	0.04	0.12	6.51	13.2	46.0
1005	-14	0.03	0.06	7.12	15.1	48.2
	31	0.07	0.10	6.25	13.3	43.4
	71	0.03	0.09	6.94	14.6	48.8

Appendix 10

Individual Hematology Values

Sex: Male

0 mg/kg /dose Group 1	Day(s) Relative to Start Date	Reporting Hematology MCV (fL)	MCH (pg)	MCHC (g/dL)	RDW (%)	PLT (10^3/uL)
1001	-14	67.3	19.7	29.2	13.0	356
	31	69.0	20.1	29.1	13.9	322
1002	-14	67.9	20.8	30.6	13.9	394
	31	67.2	21.2	31.5	14.1	395
1003	-14	65.0	19.1	29.4	14.2	411
	31	65.4	19.1	29.3	13.7	369
1004	-14	67.2	20.1	29.9	14.9	712
	31	69.5	20.5	29.5	15.3	586
	71	70.7	20.3	28.7	14.1	508
1005	-14	67.7	21.2	31.3	12.7	449
	31	69.5	21.3	30.7	12.5	436
	71	70.3	21.1	30.0	11.9	377

Appendix 10

Individual Hematology Values

Sex: Male

0 mg/kg /dose Group 1	Day(s) Relative to Start Date	Reporting Hematology RETIC (10^9/L)	ECHINO	LARGE PLATELETS	WBC MORPH
1001	-14	16.8	-	-	-
	31	19.2	-	-	-
1002	-14	34.0	-	-	-
	31	38.0	-	-	-
1003	-14	22.4	-	-	-
	31	26.0	-	-	-
1004	-14	26.2	-	-	-
	31	26.0	-	-	-
	71	36.8	-	-	-
1005	-14	69.3	-	-	-
	31	49.8	-	-	-
	71	48.2	-	-	-

Appendix 10

Individual Hematology Values

Sex: Male

3 mg/kg /dose Group 2	Day(s) Relative to Start Date	Reporting Hematology WBC (10^3/uL)	NEUT (10^3/uL)	LYMPH (10^3/uL)	MONO (10^3/uL)	EOS (10^3/uL)
2001	-14	10.95	6.26	3.82	0.66	0.02
	31	12.89	6.76	5.36	0.36	0.22
2002	-14	9.52	4.54	4.27	0.45	0.07
	31	12.38	5.15	6.58	0.31	0.12
2003	-14	8.16	3.25	4.32	0.40	0.06
	31	9.48	4.20	4.78	0.35	0.04

Appendix 10

Individual Hematology Values

Sex: Male

3 mg/kg /dose Group 2	Day(s) Relative to Start Date	Reporting Hematology				
		BASO (10^3/uL)	LUC (10^3/uL)	RBC (10^6/uL)	HGB (g/dL)	HCT (%)
2001	-14	0.05	0.15	7.30	14.6	46.6
	31	0.05	0.14	6.69	13.1	44.1
2002	-14	0.05	0.14	6.83	13.2	47.0
	31	0.07	0.15	6.00	11.6	40.4
2003	-14	0.04	0.09	7.26	13.6	48.1
	31	0.04	0.07	6.63	12.6	44.0

Appendix 10

Individual Hematology Values

Sex: Male

3 mg/kg /dose Group 2	Day(s) Relative to Start Date	Reporting Hematology MCV (fL)	MCH (pg)	MCHC (g/dL)	RDW (%)	PLT (10^3/uL)
2001	-14	63.8	20.0	31.3	13.2	392
	31	65.9	19.7	29.8	13.2	356
2002	-14	68.9	19.3	28.1	14.9	466
	31	67.3	19.3	28.7	14.5	422
2003	-14	66.2	18.7	28.2	15.4	421
	31	66.3	19.0	28.7	15.1	362

Appendix 10

Individual Hematology Values

Sex: Male

3 mg/kg /dose Group 2	Day(s) Relative to Start Date	Reporting Hematology			
		RETIC (10^9/L)	ECHINO	LARGE PLATELETS	WBC MORPH
2001	-14	40.4	-	-	-
	31	47.1	-	-	-
2002	-14	49.3	-	-	-
	31	25.6	-	-	-
2003	-14	66.1	-	-	-
	31	57.7	-	-	-

Appendix 10

Individual Hematology Values

Sex: Male

10 mg/kg /dose Group 3	Day(s) Relative to Start Date	Reporting Hematology WBC (10^3/uL)	NEUT (10^3/uL)	LYMPH (10^3/uL)	MONO (10^3/uL)	EOS (10^3/uL)
3001	-14	8.08	2.32	5.12	0.36	0.13
	31	13.91	6.24	6.51	0.33	0.60
3002	-14	13.86	7.01	5.94	0.67	0.03
	31	9.98	4.67	4.66	0.47	0.02
3003	-14	10.23	3.91	5.70	0.31	0.16
	31	12.30	6.03	5.63	0.21	0.23

Appendix 10

Individual Hematology Values

Sex: Male

10 mg/kg /dose Group 3	Day(s) Relative to Start Date	Reporting Hematology BASO (10^3/uL)	LUC (10^3/uL)	RBC (10^6/uL)	HGB (g/dL)	HCT (%)
3001	-14	0.04	0.11	7.17	14.2	48.2
	31	0.06	0.15	6.49	13.0	43.8
3002	-14	0.07	0.15	7.06	12.9	46.7
	31	0.04	0.12	6.18	11.3	39.0
3003	-14	0.03	0.11	6.93	13.8	47.3
	31	0.05	0.15	6.31	12.6	42.9

Appendix 10

Individual Hematology Values

Sex: Male

10 mg/kg /dose Group 3	Day(s) Relative to Start Date	Reporting Hematology MCV (fL)	MCH (pg)	MCHC (g/dL)	RDW (%)	PLT (10^3/uL)
3001	-14	67.3	19.7	29.4	13.8	458
	31	67.5	20.0	29.6	13.0	454
3002	-14	66.1	18.2	27.6	14.6	405
	31	63.2	18.3	28.9	14.3	314
3003	-14	68.2	19.9	29.2	14.5	436
	31	68.0	19.9	29.3	13.7	395

Appendix 10

Individual Hematology Values

Sex: Male

10 mg/kg /dose Group 3	Day(s) Relative to Start Date	Reporting Hematology			
		RETIC (10^9/L)	ECHINO	LARGE PLATELETS	WBC MORPH
3001	-14	32.7	-	-	-
	31	29.5	-	-	-
3002	-14	67.3	-	-	-
	31	44.2	-	-	-
3003	-14	62.3	-	-	-
	31	50.8	-	-	-

Appendix 10

Individual Hematology Values

Sex: Male

100 mg/kg /dose Group 4	Day(s) Relative to Start Date	Reporting Hematology WBC (10^3/uL)	NEUT (10^3/uL)	LYMPH (10^3/uL)	MONO (10^3/uL)	EOS (10^3/uL)
4001	-14	11.31	4.78	5.78	0.50	0.07
	31	21.77	13.55	7.03	0.63	0.26
4002	-14	11.25	2.59	8.10	0.45	0.11
	31	13.69	8.39	4.57	0.47	0.06
4003	-14	9.58	4.23	4.66	0.54	0.04
	31	15.79	10.07	5.04	0.42	0.08
4004	-14	10.43	4.64	5.07	0.40	0.19
	31	9.68	3.73	5.29	0.38	0.15
	71	9.96	4.72	4.71	0.38	0.07
4005	-14	7.70	2.11	4.57	0.45	0.36
	31	12.01	6.27	4.79	0.49	0.23
	71	11.51	5.23	5.32	0.57	0.19

Appendix 10

Individual Hematology Values

Sex: Male

100 mg/kg /dose Group 4	Day(s) Relative to Start Date	Reporting Hematology BASO (10^3/uL)	LUC (10^3/uL)	RBC (10^6/uL)	HGB (g/dL)	HCT (%)
4001	-14	0.04	0.14	6.64	13.3	46.3
	31	0.10	0.19	5.69	11.4	39.6
4002	-14	0.00	0.00	5.67	11.3	38.7
	31	0.05	0.14	6.46	13.2	44.2
4003	-14	0.03	0.07	7.03	13.3	44.8
	31	0.07	0.11	6.91	13.1	44.4
4004	-14	0.03	0.10	7.26	13.9	47.2
	31	0.04	0.08	6.56	12.7	43.2
	71	0.02	0.06	7.29	14.1	47.5
4005	-14	0.04	0.17	6.06	12.6	41.8
	31	0.05	0.18	5.77	12.1	40.4
	71	0.05	0.13	6.45	13.7	46.9

Appendix 10

Individual Hematology Values

Sex: Male

100 mg/kg /dose Group 4	Day(s) Relative to Start Date	Reporting Hematology MCV (fL)	MCH (pg)	MCHC (g/dL)	RDW (%)	PLT (10^3/uL)
4001	-14	69.7	20.0	28.7	15.3	328
	31	69.6	20.1	28.9	14.6	334
4002	-14	68.3	20.0	29.3	14.6	390
	31	68.4	20.5	30.0	13.9	245
4003	-14	63.7	18.8	29.6	13.9	313
	31	64.3	18.9	29.5	13.8	294
4004	-14	65.0	19.2	29.5	15.6	355
	31	65.9	19.3	29.3	14.7	375
	71	65.1	19.3	29.6	14.3	305
4005	-14	69.1	20.8	30.1	13.4	465
	31	69.9	20.9	30.0	13.7	439
	71	72.7	21.2	29.1	12.9	393

Appendix 10

Individual Hematology Values

Sex: Male

100 mg/kg /dose Group 4	Day(s) Relative to Start Date	Reporting Hematology			
		RETIC (10^9/L)	ECHINO	LARGE PLATELETS	WBC MORPH
4001	-14	63.9	-	-	-
	31	52.7	-	-	-
4002	-14	191.6	1+	1+	NAF
	31	21.4	-	-	-
4003	-14	15.8	-	-	-
	31	15.2	-	-	-
4004	-14	46.0	-	-	-
	31	28.7	-	-	-
	71	25.8	-	-	-
4005	-14	36.9	-	-	-
	31	22.8	-	-	-
	71	22.3	-	-	-

Appendix 10

Individual Hematology Values

Sex: Female

0 mg/kg /dose Group 1	Day(s) Relative to Start Date	Reporting Hematology				
		WBC (10^3/uL)	NEUT (10^3/uL)	LYMPH (10^3/uL)	MONO (10^3/uL)	EOS (10^3/uL)
1501	-14	7.50	3.96	3.20	0.22	0.05
	31	14.18	9.16	4.16	0.62	0.12
1502	-14	18.96	13.49	4.37	0.42	0.48
	31	17.71	11.44	4.09	0.33	1.66
1503	-14	6.90	2.43	3.93	0.31	0.07
	31	9.05	2.41	5.37	0.48	0.58
1504	-14	11.31	6.72	4.09	0.29	0.05
	31	9.98	5.21	4.21	0.33	0.14
	71	9.14	2.73	5.81	0.33	0.15
1505	-14	11.14	6.62	4.14	0.26	0.02
	31	11.59	4.77	6.20	0.38	0.05
	71	13.32	7.21	5.58	0.34	0.04

Appendix 10

Individual Hematology Values

Sex: Female

0 mg/kg /dose Group 1	Day(s) Relative to Start Date	Reporting Hematology BASO (10^3/uL)	LUC (10^3/uL)	RBC (10^6/uL)	HGB (g/dL)	HCT (%)
1501	-14	0.03	0.05	6.47	12.6	39.3
	31	0.04	0.08	5.19	10.4	32.1
1502	-14	0.10	0.10	7.66	13.1	46.4
	31	0.06	0.12	7.27	12.7	44.3
1503	-14	0.04	0.11	7.18	13.8	47.5
	31	0.05	0.17	6.40	12.4	42.3
1504	-14	0.03	0.12	6.44	12.2	44.3
	31	0.04	0.06	6.09	11.8	40.9
	71	0.03	0.10	6.53	12.8	46.4
1505	-14	0.02	0.08	6.64	13.3	45.8
	31	0.08	0.11	6.16	12.4	43.3
	71	0.06	0.09	6.84	13.9	47.3

Appendix 10

Individual Hematology Values

Sex: Female

0 mg/kg /dose Group 1	Day(s) Relative to Start Date	Reporting Hematology MCV (fL)	MCH (pg)	MCHC (g/dL)	RDW (%)	PLT (10^3/uL)
1501	-14	60.7	19.5	32.1	14.4	517
	31	61.8	20.0	32.4	14.7	354
1502	-14	60.7	17.0	28.1	15.8	661
	31	60.9	17.4	28.6	16.1	599
1503	-14	66.1	19.2	29.1	13.2	436
	31	66.1	19.4	29.3	13.1	392
1504	-14	68.8	19.0	27.6	14.6	443
	31	67.1	19.5	29.0	14.2	381
	71	71.1	19.6	27.6	12.9	417
1505	-14	68.9	20.0	29.1	14.8	383
	31	70.2	20.1	28.6	14.0	303
	71	69.2	20.3	29.4	12.8	262

Appendix 10

Individual Hematology Values

Sex: Female

0 mg/kg /dose Group 1	Day(s) Relative to Start Date	Reporting Hematology RETIC (10^9/L)	ECHINO	LARGE PLATELETS	WBC MORPH
1501	-14	112.8	-	-	-
	31	123.4	-	-	-
1502	-14	44.2	-	-	-
	31	48.4	-	-	-
1503	-14	53.5	-	-	-
	31	22.2	-	-	-
1504	-14	87.7	-	-	-
	31	30.5	-	-	-
	71	25.0	-	-	-
1505	-14	62.3	-	-	-
	31	41.6	-	-	-
	71	30.7	-	-	-

Appendix 10

Individual Hematology Values

Sex: Female

3 mg/kg /dose Group 2	Day(s) Relative to Start Date	Reporting Hematology WBC (10^3/uL)	NEUT (10^3/uL)	LYMPH (10^3/uL)	MONO (10^3/uL)	EOS (10^3/uL)
2501	-14	15.15	11.00	3.50	0.39	0.14
	31	9.18	4.46	3.92	0.41	0.24
2502	-14	10.53	6.81	3.29	0.31	0.03
	31	8.10	3.97	3.63	0.32	0.06
2503	-14	15.30	9.83	4.94	0.28	0.10
	31	10.94	4.66	5.53	0.24	0.31

Appendix 10

Individual Hematology Values

Sex: Female

3 mg/kg /dose Group 2	Day(s) Relative to Start Date	Reporting Hematology BASO (10^3/uL)	LUC (10^3/uL)	RBC (10^6/uL)	HGB (g/dL)	HCT (%)
2501	-14	0.04	0.09	6.31	12.8	42.7
	31	0.04	0.11	5.73	12.0	40.1
2502	-14	0.04	0.04	7.10	12.9	44.5
	31	0.04	0.08	6.49	12.2	41.7
2503	-14	0.05	0.10	6.62	13.2	45.7
	31	0.06	0.15	6.11	12.7	43.7

Appendix 10

Individual Hematology Values

Sex: Female

3 mg/kg /dose Group 2	Day(s) Relative to Start Date	Reporting Hematology				
		MCV (fL)	MCH (pg)	MCHC (g/dL)	RDW (%)	PLT (10^3/uL)
2501	-14	67.7	20.4	30.1	14.4	390
	31	69.9	20.9	29.9	14.2	312
2502	-14	62.6	18.1	28.9	14.0	550
	31	64.3	18.8	29.2	14.0	459
2503	-14	69.1	20.0	29.0	14.0	561
	31	71.5	20.8	29.1	13.6	491

Appendix 10

Individual Hematology Values

Sex: Female

3 mg/kg /dose Group 2	Day(s) Relative to Start Date	Reporting Hematology			
		RETIC (10^9/L)	ECHINO	LARGE PLATELETS	WBC MORPH
2501	-14	53.5	-	-	-
	31	42.6	-	-	-
2502	-14	28.9	-	-	-
	31	20.9	-	-	-
2503	-14	45.2	-	-	-
	31	33.0	-	-	-

Appendix 10

Individual Hematology Values

Sex: Female

10 mg/kg /dose Group 3	Day(s) Relative to Start Date	Reporting Hematology				
		WBC (10^3/uL)	NEUT (10^3/uL)	LYMPH (10^3/uL)	MONO (10^3/uL)	EOS (10^3/uL)
3501	-14	14.64	9.53	4.32	0.52	0.12
	31	9.91	5.07	3.96	0.57	0.11
3502	-14	8.11	4.20	3.35	0.36	0.08
	31	10.75	3.43	6.05	0.61	0.44
3503	-14	11.68	7.17	4.03	0.35	0.03
	31	12.70	5.06	6.88	0.43	0.15

Appendix 10

Individual Hematology Values

Sex: Female

10 mg/kg /dose Group 3	Day(s) Relative to Start Date	Reporting Hematology				
		BASO (10^3/uL)	LUC (10^3/uL)	RBC (10^6/uL)	HGB (g/dL)	HCT (%)
3501	-14	0.05	0.12	7.02	12.5	43.1
	31	0.04	0.16	5.71	10.4	36.2
3502	-14	0.03	0.09	6.48	13.6	46.1
	31	0.08	0.15	6.02	12.6	42.7
3503	-14	0.04	0.07	7.56	13.5	48.8
	31	0.08	0.10	7.42	13.4	47.7

Appendix 10

Individual Hematology Values

Sex: Female

10 mg/kg /dose Group 3	Day(s) Relative to Start Date	Reporting Hematology				
		MCV (fL)	MCH (pg)	MCHC (g/dL)	RDW (%)	PLT (10^3/uL)
3501	-14	61.4	17.8	29.1	17.3	396
	31	63.4	18.2	28.7	16.2	329
3502	-14	71.2	21.0	29.5	14.3	472
	31	70.9	21.0	29.6	13.7	398
3503	-14	64.5	17.8	27.6	15.5	468
	31	64.3	18.0	28.0	15.2	407

Appendix 10

Individual Hematology Values

Sex: Female

10 mg/kg /dose Group 3	Day(s) Relative to Start Date	Reporting Hematology			
		RETIC (10^9/L)	ECHINO	LARGE PLATELETS	WBC MORPH
3501	-14	81.5	-	-	-
	31	57.2	-	-	-
3502	-14	43.6	-	-	-
	31	21.2	-	-	-
3503	-14	46.5	-	-	-
	31	40.6	-	-	-

Appendix 10

Individual Hematology Values

Sex: Female

100 mg/kg /dose Group 4	Day(s) Relative to Start Date	Reporting Hematology				
		WBC (10^3/uL)	NEUT (10^3/uL)	LYMPH (10^3/uL)	MONO (10^3/uL)	EOS (10^3/uL)
4501	-14	13.32	9.87	3.20	0.14	0.01
	31	12.42	7.32	4.68	0.24	0.03
4502	-14	27.86	23.01	4.10	0.53	0.05
	31	16.56	6.30	8.80	0.83	0.35
4503	-14	25.90	21.65	3.22	0.59	0.24
	31	18.40	12.91	4.29	0.57	0.39
4504	-14	17.22	11.45	5.08	0.46	0.09
	31	14.49	6.70	6.99	0.40	0.22
	71	16.27	11.04	4.59	0.49	0.05
4505	-14	8.99	4.08	4.44	0.25	0.11
	31	12.47	7.37	4.25	0.34	0.29
	71	18.63	13.95	3.84	0.57	0.14

Appendix 10

Individual Hematology Values

Sex: Female

100 mg/kg /dose Group 4	Day(s) Relative to Start Date	Reporting Hematology BASO (10^3/uL)	LUC (10^3/uL)	RBC (10^6/uL)	HGB (g/dL)	HCT (%)
4501	-14	0.03	0.07	6.37	13.0	42.9
	31	0.05	0.09	5.62	11.8	39.0
4502	-14	0.10	0.07	6.25	12.4	44.0
	31	0.08	0.20	6.54	13.2	48.3
4503	-14	0.08	0.12	6.70	13.7	45.7
	31	0.08	0.16	5.98	12.4	42.2
4504	-14	0.06	0.09	7.28	14.1	48.9
	31	0.09	0.10	6.78	13.0	46.4
	71	0.04	0.05	7.52	14.7	52.9
4505	-14	0.04	0.08	6.86	13.0	43.1
	31	0.04	0.19	5.93	11.2	37.8
	71	0.04	0.09	6.76	12.7	44.2

Appendix 10

Individual Hematology Values

Sex: Female

100 mg/kg /dose Group 4	Day(s) Relative to Start Date	Reporting Hematology MCV (fL)	MCH (pg)	MCHC (g/dL)	RDW (%)	PLT (10^3/uL)
4501	-14	67.4	20.4	30.3	12.6	417
	31	69.4	20.9	30.2	12.7	244
4502	-14	70.5	19.8	28.1	14.2	627
	31	73.8	20.2	27.4	13.6	503
4503	-14	68.2	20.5	30.1	13.6	380
	31	70.6	20.7	29.3	14.2	365
4504	-14	67.1	19.4	28.9	14.4	511
	31	68.4	19.2	28.1	14.0	413
	71	70.4	19.5	27.7	13.3	373
4505	-14	62.8	18.9	30.1	15.5	320
	31	63.8	18.9	29.6	14.5	211
	71	65.3	18.7	28.7	13.9	249

Appendix 10

Individual Hematology Values

Sex: Female

100 mg/kg /dose Group 4	Day(s) Relative to Start Date	Reporting Hematology			
		RETIC (10^9/L)	ECHINO	LARGE PLATELETS	WBC MORPH
4501	-14	26.9	-	-	-
	31	44.0	-	-	-
4502	-14	115.8	-	-	-
	31	46.2	-	-	-
4503	-14	31.9	-	-	-
	31	55.2	-	-	-
4504	-14	29.2	-	-	-
	31	30.1	-	-	-
	71	25.7	-	-	-
4505	-14	84.4	-	-	-
	31	61.9	-	-	-
	71	77.6	-	-	-

Appendix 12

Individual Clinical Chemistry Values

Sex: Male

0 mg/kg /dose Group 1	Day(s) Relative to Start Date	Reporting Biochemistry AST (U/L)	ALT (U/L)	ALP (U/L)	GGT (U/L)	CK (U/L)	TBIL (mg/dL)
1001	-14	61	70	539	110	274	0.20
	31	55	82	720	138	202	0.18
1002	-14	42	41	1008	230	152	0.29
	31	61	44	913	193	1016	0.40
1003	-14	74	46	842	101	3472	0.31
	31	44	48	894	99	240	0.33
1004	-14	116	46	556	124	6076	0.32
	31	53	63	641	158	449	0.24
	71	41	38	626	172	190	0.11
1005	-14	49	62	917	165	308	0.16
	31	78	85	1225	178	584	0.34
	71	38	52	1231	194	504	0.14

Appendix 12

Individual Clinical Chemistry Values

Sex: Male

0 mg/kg /dose Group 1	Day(s) Relative to Start Date	Reporting Biochemistry UREAN (mg/dL)	CREAT (mg/dL)	GLUC (mg/dL)	CHOL (mg/dL)	TRIG (mg/dL)	TPROT (g/dL)
1001	-14	26	0.6	58	141	39	7.2
	31	27	0.6	68	129	50	7.5
1002	-14	20	0.6	68	93	44	7.7
	31	17	0.7	53	87	58	8.4
1003	-14	19	0.6	84	120	44	7.9
	31	18	0.5	61	115	64	8.3
1004	-14	23	0.6	58	113	64	7.7
	31	19	0.5	71	100	32	7.9
	71	25	0.5	69	92	32	7.2
1005	-14	22	0.5	69	114	43	7.1
	31	19	0.5	59	103	76	7.5
	71	22	0.5	65	92	46	6.9

Appendix 12

Individual Clinical Chemistry Values

Sex: Male

0 mg/kg /dose Group 1	Day(s) Relative to Start Date	Reporting Biochemistry ALB (g/dL)	GLOB (g/dL)	A/G (ratio)	CA (mg/dL)	PHOS (mg/dL)	NA (mEq/L)
1001	-14	4.7	2.5	1.9	10.1	5.8	148
	31	4.4	3.1	1.4	9.6	6.9	148
1002	-14	5.0	2.7	1.9	10.0	5.7	149
	31	4.9	3.5	1.4	10.3	6.1	148
1003	-14	5.2	2.7	1.9	10.2	5.8	146
	31	4.9	3.4	1.4	10.0	5.5	146
1004	-14	5.1	2.6	2.0	9.8	5.8	150
	31	4.5	3.4	1.3	10.1	7.2	149
	71	4.5	2.7	1.7	9.7	5.8	147
1005	-14	4.6	2.5	1.8	9.6	5.5	147
	31	4.3	3.2	1.3	9.3	7.6	146
	71	4.4	2.5	1.8	9.4	6.7	148

Appendix 12

Individual Clinical Chemistry Values

Sex: Male

0 mg/kg /dose Group 1	Day(s) Relative to Start Date	Reporting Biochemistry K (mEq/L)	CL (mEq/L)	SAMQ	CRP3 (mg/dL)
1001	-14	5.2	113	N	0.053
	31	4.0	106	N	0.062
1002	-14	4.1	105	N	0.020
	31	4.7	107	H+	0.028
1003	-14	4.4	107	H+	0.081
	31	4.2	107	N	0.208
1004	-14	6.6	110	H+++	0.042
	31	4.7	107	N	0.115
	71	4.3	103	N	0.057
1005	-14	4.4	112	N	0.099
	31	4.1	108	N	0.432
	71	4.0	107	N	0.091

Appendix 12

Individual Clinical Chemistry Values

Sex: Male

3 mg/kg /dose Group 2	Day(s) Relative to Start Date	Reporting Biochemistry					
		AST (U/L)	ALT (U/L)	ALP (U/L)	GGT (U/L)	CK (U/L)	TBIL (mg/dL)
2001	-14	62	43	698	92	756	0.23
	31	58	55	783	95	159	0.16
2002	-14	133	103	754	141	QNS	QNS
	31	49	68	684	108	116	0.25
2003	-14	60	31	519	103	95	0.21
	31	192	100	703	127	4956	0.33

Appendix 12

Individual Clinical Chemistry Values

Sex: Male

3 mg/kg /dose Group 2	Day(s) Relative to Start Date	Reporting Biochemistry					
		UREAN (mg/dL)	CREAT (mg/dL)	GLUC (mg/dL)	CHOL (mg/dL)	TRIG (mg/dL)	TPROT (g/dL)
2001	-14	17	0.7	73	104	53	7.8
	31	18	0.8	76	105	55	8.0
2002	-14	30	0.5	66	127	QNS	7.3
	31	20	0.6	55	127	57	8.0
2003	-14	26	0.6	84	97	34	7.2
	31	23	0.6	54	102	39	7.7

Appendix 12

Individual Clinical Chemistry Values

Sex: Male

3 mg/kg /dose Group 2	Day(s) Relative to Start Date	Reporting Biochemistry					
		ALB (g/dL)	GLOB (g/dL)	A/G (ratio)	CA (mg/dL)	PHOS (mg/dL)	NA (mEq/L)
2001	-14	5.1	2.7	1.9	10.6	6.0	148
	31	4.4	3.6	1.2	9.5	5.8	147
2002	-14	5.0	2.3	2.2	10.0	6.6	152
	31	4.7	3.3	1.4	10.2	6.8	153
2003	-14	4.8	2.4	2.0	10.2	5.2	149
	31	4.5	3.2	1.4	9.7	7.4	148

Appendix 12

Individual Clinical Chemistry Values

Sex: Male

3 mg/kg /dose Group 2	Day(s) Relative to Start Date	Reporting Biochemistry K (mEq/L)	CL (mEq/L)	SAMQ	CRP3 (mg/dL)
2001	-14	5.3	109	H++	0.333
	31	4.1	106	N	0.094
2002	-14	5.4	115	H+++	0.270
	31	5.0	110	H+	0.136
2003	-14	5.1	108	H++	0.037
	31	5.3	107	N	0.101

Appendix 12

Individual Clinical Chemistry Values

Sex: Male

10 mg/kg /dose Group 3	Day(s) Relative to Start Date	Reporting Biochemistry AST (U/L)	ALT (U/L)	ALP (U/L)	GGT (U/L)	CK (U/L)	TBIL (mg/dL)
3001	-14	61	43	799	186	1285	0.20
	31	55	43	872	183	188	0.34
3002	-14	38	100	512	120	195	0.19
	31	37	80	623	142	143	0.25
3003	-14	89	74	864	201	342	0.29
	31	109	48	710	170	900	0.30

Appendix 12

Individual Clinical Chemistry Values

Sex: Male

10 mg/kg /dose Group 3	Day(s) Relative to Start Date	Reporting Biochemistry					
		UREAN (mg/dL)	CREAT (mg/dL)	GLUC (mg/dL)	CHOL (mg/dL)	TRIG (mg/dL)	TPROT (g/dL)
3001	-14	20	0.7	75	114	34	7.9
	31	21	0.6	50	95	44	8.2
3002	-14	21	0.7	73	119	33	7.9
	31	17	0.5	53	90	41	7.3
3003	-14	20	0.7	59	106	33	8.1
	31	19	0.6	49	97	31	8.3

Appendix 12

Individual Clinical Chemistry Values

Sex: Male

10 mg/kg /dose Group 3	Day(s) Relative to Start Date	Reporting Biochemistry					
		ALB (g/dL)	GLOB (g/dL)	A/G (ratio)	CA (mg/dL)	PHOS (mg/dL)	NA (mEq/L)
3001	-14	5.1	2.8	1.8	10.5	5.7	149
	31	4.7	3.5	1.3	10.8	6.4	149
3002	-14	5.0	2.9	1.7	10.7	6.7	156
	31	4.1	3.2	1.3	9.8	7.0	146
3003	-14	4.8	3.3	1.5	10.1	5.7	150
	31	4.4	3.9	1.1	10.0	5.9	147

Appendix 12

Individual Clinical Chemistry Values

Sex: Male

10 mg/kg /dose Group 3	Day(s) Relative to Start Date	Reporting Biochemistry			
		K (mEq/L)	CL (mEq/L)	SAMQ	CRP3 (mg/dL)
3001	-14	4.5	108	H+	0.101
	31	5.3	111	N	0.198
3002	-14	5.3	113	N	0.162
	31	4.3	111	N	0.171
3003	-14	4.1	110	N	0.188
	31	3.9	109	N	0.212

Appendix 12

Individual Clinical Chemistry Values

Sex: Male

100 mg/kg /dose Group 4	Day(s) Relative to Start Date	Reporting Biochemistry					
		AST (U/L)	ALT (U/L)	ALP (U/L)	GGT (U/L)	CK (U/L)	TBIL (mg/dL)
4001	-14	58	51	434	146	121	0.23
	31	80	61	341	117	1114	0.24
4002	-14	42	52	710	147	118	0.15
	31	46	49	839	213	489	0.20
4003	-14	60	51	607	124	167	0.23
	31	55	60	808	147	152	0.25
4004	-14	89	72	965	129	394	0.28
	31	118	95	901	135	1003	0.29
	71	39	48	798	154	168	0.17
4005	-14	49	51	553	144	583	0.14
	31	68	78	803	173	207	0.29
	71	31	43	775	208	130	0.14

Appendix 12

Individual Clinical Chemistry Values

Sex: Male

100 mg/kg /dose Group 4	Day(s) Relative to Start Date	Reporting Biochemistry					
		UREAN (mg/dL)	CREAT (mg/dL)	GLUC (mg/dL)	CHOL (mg/dL)	TRIG (mg/dL)	TPROT (g/dL)
4001	-14	21	0.7	75	147	23	8.2
	31	23	0.7	59	118	33	8.5
4002	-14	18	0.5	90	97	29	7.6
	31	16	0.5	60	133	50	8.5
4003	-14	24	0.6	63	119	26	7.9
	31	24	0.7	53	117	39	8.7
4004	-14	26	0.6	53	139	46	7.7
	31	22	0.6	61	132	36	8.4
	71	27	0.6	72	139	45	7.2
4005	-14	26	0.8	63	101	32	7.5
	31	23	0.8	48	111	40	8.1
	71	26	0.9	72	119	42	7.6

Appendix 12

Individual Clinical Chemistry Values

Sex: Male

100 mg/kg /dose Group 4	Day(s) Relative to Start Date	Reporting Biochemistry ALB (g/dL)	GLOB (g/dL)	A/G (ratio)	CA (mg/dL)	PHOS (mg/dL)	NA (mEq/L)
4001	-14	5.2	3.0	1.7	10.4	5.8	153
	31	4.6	3.9	1.2	9.5	7.4	147
4002	-14	4.2	3.4	1.2	9.1	6.6	146
	31	4.5	4.0	1.1	9.9	7.4	146
4003	-14	4.9	3.0	1.6	10.0	5.2	148
	31	4.6	4.1	1.1	9.8	5.6	146
4004	-14	4.9	2.8	1.8	10.3	7.5	154
	31	4.7	3.7	1.3	10.4	7.0	151
	71	4.5	2.7	1.7	9.7	6.6	151
4005	-14	4.9	2.6	1.9	10.1	5.3	151
	31	4.6	3.5	1.3	9.9	6.5	147
	71	4.9	2.7	1.8	10.3	6.5	151

Appendix 12

Individual Clinical Chemistry Values

Sex: Male

100 mg/kg /dose Group 4	Day(s) Relative to Start Date	Reporting Biochemistry K (mEq/L)	CL (mEq/L)	SAMQ	CRP3 (mg/dL)
4001	-14	4.4	107	N	0.015
	31	4.1	106	N	0.127
4002	-14	4.0	108	N	0.044
	31	4.3	106	N	1.108
4003	-14	4.6	111	H++	0.006
	31	3.6	106	N	0.008
4004	-14	4.7	109	N	0.357
	31	5.1	108	N	0.445
	71	4.3	110	N	0.145
4005	-14	4.2	110	N	0.069
	31	4.2	107	N	0.079
	71	4.4	106	N	0.025

Appendix 12

Individual Clinical Chemistry Values

Sex: Female

0 mg/kg /dose Group 1	Day(s) Relative to Start Date	Reporting Biochemistry					
		AST (U/L)	ALT (U/L)	ALP (U/L)	GGT (U/L)	CK (U/L)	TBIL (mg/dL)
1501	-14	61	47	536	120	2488	0.53
	31	42	63	453	127	212	0.76
1502	-14	39	45	471	64	219	0.09
	31	52	44	410	49	247	0.14
1503	-14	40	80	620	96	189	0.16
	31	52	65	537	103	461	0.17
1504	-14	50	51	372	89	252	0.12
	31	51	54	405	95	220	0.18
	71	42	54	361	102	194	0.11
1505	-14	35	40	526	93	133	0.33
	31	141	97	476	100	1233	0.55
	71	35	44	561	116	187	0.45

Appendix 12

Individual Clinical Chemistry Values

Sex: Female

0 mg/kg /dose Group 1	Day(s) Relative to Start Date	Reporting Biochemistry UREAN (mg/dL)	CREAT (mg/dL)	GLUC (mg/dL)	CHOL (mg/dL)	TRIG (mg/dL)	TPROT (g/dL)
1501	-14	22	0.6	65	97	44	7.8
	31	21	0.6	57	84	38	7.7
1502	-14	20	0.6	73	88	43	7.9
	31	17	0.5	62	93	59	7.3
1503	-14	23	0.6	50	136	32	7.3
	31	19	0.5	58	135	31	7.7
1504	-14	23	0.6	57	116	38	7.5
	31	19	0.5	63	133	34	7.8
	71	23	0.6	62	127	50	7.7
1505	-14	21	0.6	85	107	35	7.3
	31	21	0.6	65	116	40	7.7
	71	23	0.6	72	114	36	7.3

Appendix 12

Individual Clinical Chemistry Values

Sex: Female

0 mg/kg /dose Group 1	Day(s) Relative to Start Date	Reporting Biochemistry					
		ALB (g/dL)	GLOB (g/dL)	A/G (ratio)	CA (mg/dL)	PHOS (mg/dL)	NA (mEq/L)
1501	-14	5.0	2.8	1.8	9.3	8.6	147
	31	4.3	3.4	1.3	9.5	7.6	148
1502	-14	4.3	3.6	1.2	10.0	5.5	150
	31	3.6	3.7	1.0	9.1	6.3	146
1503	-14	4.6	2.7	1.7	9.9	6.3	148
	31	4.2	3.5	1.2	9.2	7.1	144
1504	-14	4.6	2.9	1.6	10.6	4.6	150
	31	4.1	3.7	1.1	9.4	6.5	146
	71	4.4	3.3	1.3	9.9	5.3	147
1505	-14	4.8	2.5	1.9	10.2	4.5	151
	31	4.5	3.2	1.4	10.0	6.3	150
	71	4.6	2.7	1.7	9.7	6.2	148

Appendix 12

Individual Clinical Chemistry Values

Sex: Female

0 mg/kg /dose Group 1	Day(s) Relative to Start Date	Reporting Biochemistry K (mEq/L)	CL (mEq/L)	SAMQ	CRP3 (mg/dL)
1501	-14	6.0	111	H+++	0.031
	31	5.1	109	H+	0.539
1502	-14	5.2	109	N	0.354
	31	4.3	108	N	0.643
1503	-14	4.2	110	N	0.077
	31	4.7	111	N	0.152
1504	-14	4.4	110	H+	0.044
	31	4.1	110	N	0.045
	71	4.0	106	N	0.004
1505	-14	4.2	109	N	0.125
	31	4.6	108	H+	0.406
	71	3.8	108	N	0.059

Appendix 12

Individual Clinical Chemistry Values

Sex: Female

3 mg/kg /dose Group 2	Day(s) Relative to Start Date	Reporting Biochemistry AST (U/L)	ALT (U/L)	ALP (U/L)	GGT (U/L)	CK (U/L)	TBIL (mg/dL)
2501	-14	43	41	682	123	403	0.31
	31	58	42	735	135	228	0.40
2502	-14	66	64	584	78	757	0.22
	31	50	62	534	72	311	0.20
2503	-14	53	57	575	97	260	0.14
	31	56	80	618	103	209	0.22

Appendix 12

Individual Clinical Chemistry Values

Sex: Female

3 mg/kg /dose Group 2	Day(s) Relative to Start Date	Reporting Biochemistry UREAN (mg/dL)	CREAT (mg/dL)	GLUC (mg/dL)	CHOL (mg/dL)	TRIG (mg/dL)	TPROT (g/dL)
2501	-14	23	0.7	58	134	55	7.5
	31	22	0.6	63	141	64	7.9
2502	-14	20	0.5	50	103	42	7.1
	31	19	0.5	54	104	36	7.6
2503	-14	20	0.5	62	105	50	6.7
	31	17	0.5	60	114	69	7.4

Appendix 12

Individual Clinical Chemistry Values

Sex: Female

3 mg/kg /dose Group 2	Day(s) Relative to Start Date	Reporting Biochemistry ALB (g/dL)	GLOB (g/dL)	A/G (ratio)	CA (mg/dL)	PHOS (mg/dL)	NA (mEq/L)
2501	-14	4.6	2.9	1.6	9.5	4.4	147
	31	4.3	3.6	1.2	9.1	5.7	147
2502	-14	4.5	2.6	1.7	9.9	5.6	148
	31	4.1	3.5	1.2	9.7	6.4	146
2503	-14	4.5	2.2	2.0	9.4	6.9	147
	31	4.3	3.1	1.4	9.3	8.1	146

Appendix 12

Individual Clinical Chemistry Values

Sex: Female

3 mg/kg /dose Group 2	Day(s) Relative to Start Date	Reporting Biochemistry K (mEq/L)	CL (mEq/L)	SAMQ	CRP3 (mg/dL)
2501	-14	4.2	111	N	0.096
	31	4.1	108	N	0.190
2502	-14	5.1	112	H+	0.006
	31	5.1	109	N	0.009
2503	-14	4.6	110	H+	0.080
	31	3.9	109	N	0.103

Appendix 12

Individual Clinical Chemistry Values

Sex: Female

10 mg/kg /dose Group 3	Day(s) Relative to Start Date	Reporting Biochemistry					
		AST (U/L)	ALT (U/L)	ALP (U/L)	GGT (U/L)	CK (U/L)	TBIL (mg/dL)
3501	-14	33	46	671	114	164	0.17
	31	44	49	570	123	282	0.20
3502	-14	37	52	635	103	124	0.22
	31	48	65	571	91	324	0.19
3503	-14	55	67	741	105	322	0.33
	31	44	68	759	130	187	0.23

Appendix 12

Individual Clinical Chemistry Values

Sex: Female

10 mg/kg /dose Group 3	Day(s) Relative to Start Date	Reporting Biochemistry					
		UREAN (mg/dL)	CREAT (mg/dL)	GLUC (mg/dL)	CHOL (mg/dL)	TRIG (mg/dL)	TPROT (g/dL)
3501	-14	21	0.7	89	149	44	8.4
	31	24	0.6	56	124	32	8.3
3502	-14	18	0.6	66	135	32	7.4
	31	21	0.6	63	123	22	7.3
3503	-14	19	0.7	52	117	55	8.0
	31	18	0.6	61	110	37	8.3

Appendix 12

Individual Clinical Chemistry Values

Sex: Female

10 mg/kg /dose Group 3	Day(s) Relative to Start Date	Reporting Biochemistry					
		ALB (g/dL)	GLOB (g/dL)	A/G (ratio)	CA (mg/dL)	PHOS (mg/dL)	NA (mEq/L)
3501	-14	5.0	3.4	1.5	10.8	4.8	148
	31	4.5	3.8	1.2	9.5	5.7	146
3502	-14	4.9	2.5	2.0	10.9	6.2	152
	31	4.3	3.0	1.4	10.3	6.7	150
3503	-14	5.2	2.8	1.9	10.3	5.6	153
	31	4.7	3.6	1.3	10.2	6.7	149

Appendix 12

Individual Clinical Chemistry Values

Sex: Female

10 mg/kg /dose Group 3	Day(s) Relative to Start Date	Reporting Biochemistry K (mEq/L)	CL (mEq/L)	SAMQ	CRP3 (mg/dL)
3501	-14	3.9	110	N	0.088
	31	4.4	108	N	0.218
3502	-14	4.9	111	N	0.028
	31	4.7	111	N	0.017
3503	-14	4.6	109	H+	0.018
	31	4.8	107	N	0.007

Appendix 12

Individual Clinical Chemistry Values

Sex: Female

100 mg/kg /dose Group 4	Day(s) Relative to Start Date	Reporting Biochemistry AST (U/L)	ALT (U/L)	ALP (U/L)	GGT (U/L)	CK (U/L)	TBIL (mg/dL)
4501	-14	42	56	676	70	339	0.40
	31	54	57	533	66	223	0.40
4502	-14	64	79	530	107	2488	0.20
	31	47	66	641	149	248	0.22
4503	-14	50	46	694	105	414	0.36
	31	61	63	661	121	259	0.40
4504	-14	143	69	808	90	13180	0.32
	31	76	89	651	92	570	0.15
	71	35	51	635	90	186	0.10
4505	-14	25	41	520	73	200	0.17
	31	135	105	468	68	2743	0.14
	71	25	40	465	67	178	0.13

Appendix 12

Individual Clinical Chemistry Values

Sex: Female

100 mg/kg /dose Group 4	Day(s) Relative to Start Date	Reporting Biochemistry					
		UREAN (mg/dL)	CREAT (mg/dL)	GLUC (mg/dL)	CHOL (mg/dL)	TRIG (mg/dL)	TPROT (g/dL)
4501	-14	20	0.5	55	122	43	7.0
	31	23	0.5	52	101	42	7.3
4502	-14	25	0.7	83	148	50	7.0
	31	22	0.8	124	147	46	7.8
4503	-14	19	0.5	65	99	49	8.3
	31	15	0.5	69	116	45	8.7
4504	-14	21	0.6	82	86	62	7.4
	31	22	0.6	97	84	52	8.2
	71	26	0.6	82	87	58	7.9
4505	-14	22	0.6	73	142	40	6.7
	31	27	0.6	113	120	37	7.1
	71	27	0.6	74	128	40	7.0

Appendix 12

Individual Clinical Chemistry Values

Sex: Female

100 mg/kg /dose Group 4	Day(s) Relative to Start Date	Reporting Biochemistry					
		ALB (g/dL)	GLOB (g/dL)	A/G (ratio)	CA (mg/dL)	PHOS (mg/dL)	NA (mEq/L)
4501	-14	4.6	2.4	1.9	9.4	5.6	147
	31	4.1	3.2	1.3	9.5	6.3	145
4502	-14	4.3	2.7	1.6	9.3	7.4	146
	31	4.3	3.5	1.2	10.3	9.8	152
4503	-14	5.2	3.1	1.7	10.7	5.6	150
	31	4.7	4.0	1.2	9.8	6.3	146
4504	-14	4.9	2.5	2.0	9.9	7.6	147
	31	4.5	3.7	1.2	10.4	5.8	150
	71	4.6	3.3	1.4	10.0	6.1	152
4505	-14	4.6	2.1	2.2	10.2	6.3	145
	31	4.0	3.1	1.3	7.1	5.5	144
	71	4.4	2.6	1.7	9.1	5.5	146

Appendix 12

Individual Clinical Chemistry Values

Sex: Female

100 mg/kg /dose Group 4	Day(s) Relative to Start Date	Reporting Biochemistry K (mEq/L)	CL (mEq/L)	SAMQ	CRP3 (mg/dL)
4501	-14	3.4	110	N	0.012
	31	4.3	109	N	0.010
4502	-14	5.6	110	H+++	0.153
	31	5.7	113	H+	0.717
4503	-14	4.4	110	N	1.287
	31	4.6	110	N	0.245
4504	-14	8.1	112	H+++	0.328
	31	5.1	110	H+	0.123
	71	4.2	105	N	0.068
4505	-14	4.2	111	N	0.030
	31	3.7	110	N	0.044
	71	3.8	107	N	0.055

Appendix 19

SUMMARY OF IMMUNOPHENOTYPING VALUES

ANALYSIS	GROUP:	MALES<br>0 MG/KG/DAY	3 MG/KG/DAY	10 MG/KG/DAY	100 MG/KG/DAY
CD3+ (%)					
DAY -14	MEAN	57.6	56.0	56.8	57.0
	% DIFFERENCE		-2.8	-1.4	-1.0
	S.D.	4.71	3.63	2.74	4.51
	N	5	3	3	5
DAY 31	MEAN	63.2	65.4	69.7	68.3
	% DIFFERENCE		3.5	10.3	8.1
	S.D.	4.44	3.89	4.41	3.64
	N	5	3	3	5
DAY 71	MEAN	58.9	NA	NA	62.8
	% DIFFERENCE				6.6
	S.D.	5.87			6.58
	N	2			2

thous/uL = THOUSANDS/MICROLITER

None significantly different from control group
NA = NOT APPLICABLE

Appendix 19

SUMMARY OF IMMUNOPHENOTYPING VALUES

ANALYSIS	GROUP:	0 MG/KG/DAY	3 MG/KG/DAY	10 MG/KG/DAY	100 MG/KG/DAY
			MALES		
CD3+CD4+ (%)					
DAY -14	MEAN	33.2	28.8	30.1	29.4
	% DIFFERENCE		-13.3	-9.3	-11.4
	S.D.	2.66	3.87	0.81	8.14
	N	5	3	3	5
DAY 31	MEAN	35.6	32.8	38.0	38.5
	% DIFFERENCE		-7.9	6.7	8.1
	S.D.	2.84	3.81	3.80	6.22
	N	5	3	3	5
DAY 71	MEAN	29.9	NA	NA	38.0
	% DIFFERENCE				27.1
	S.D.	0.42			7.07
	N	2			2

thous/uL = THOUSANDS/MICROLITER

None significantly different from control group
NA = NOT APPLICABLE

Appendix 19

SUMMARY OF IMMUNOPHENOTYPING VALUES

ANALYSIS	GROUP:	0 MG/KG/DAY	MALES<br>3 MG/KG/DAY	10 MG/KG/DAY	100 MG/KG/DAY
CD3+CD8+ (%)					
DAY -14	MEAN	22.1	23.8	24.5	24.1
	% DIFFERENCE		7.7	10.9	9.0
	S.D.	4.01	3.12	5.44	4.43
	N	5	3	3	5
DAY 31	MEAN	25.3	28.7	29.6	26.7
	% DIFFERENCE		13.4	17.0	5.5
	S.D.	3.85	4.56	2.55	2.78
	N	5	3	3	5
DAY 71	MEAN	26.8	NA	NA	21.6
	% DIFFERENCE				-19.4
	S.D.	2.69			0.42
	N	2			2

thous/uL = THOUSANDS/MICROLITER

None significantly different from control group
NA = NOT APPLICABLE

Appendix 19

SUMMARY OF IMMUNOPHENOTYPING VALUES

ANALYSIS	GROUP:	MALES 0 MG/KG/DAY	3 MG/KG/DAY	10 MG/KG/DAY	100 MG/KG/DAY
CD3-CD20+ (%)					
DAY -14	MEAN	15.4	13.0	11.8	10.5
	% DIFFERENCE		-15.6	-23.4	-31.8
	S.D.	4.80	0.79	4.10	2.38
	N	5	3	3	5
DAY 31	MEAN	13.1	11.7	10.5	10.1
	% DIFFERENCE		-10.7	-19.8	-22.9
	S.D.	3.67	1.40	2.26	1.93
	N	5	3	3	5
DAY 71	MEAN	12.1	NA	NA	12.2
	% DIFFERENCE				0.8
	S.D.	0.00			1.56
	N	2			2

thous/uL = THOUSANDS/MICROLITER

None significantly different from control group
NA = NOT APPLICABLE

Appendix 19

SUMMARY OF IMMUNOPHENOTYPING VALUES

ANALYSIS	GROUP:	MALES 0 MG/KG/DAY	3 MG/KG/DAY	10 MG/KG/DAY	100 MG/KG/DAY
CD3-CD16+ (%)					
DAY -14	MEAN	6.0	14.3*	5.1	7.6
	% DIFFERENCE		138.3	-15.0	26.7
	S.D.	1.43	2.84	1.97	5.83
	N	5	3	3	5
DAY 31	MEAN	1.6	2.2	1.3	2.2
	% DIFFERENCE		37.5	-18.8	37.5
	S.D.	1.43	1.47	0.12	1.18
	N	5	3	3	5
DAY 71	MEAN	2.3	NA	NA	1.8
	% DIFFERENCE				-21.7
	S.D.	2.05			0.42
	N	2			2

thous/uL = THOUSANDS/MICROLITER

* = Significantly different from the control group at 0.05 using Dunnett's test
NA = NOT APPLICABLE

Appendix 19

SUMMARY OF IMMUNOPHENOTYPING VALUES

ANALYSIS	GROUP:	MALES 0 MG/KG/DAY	3 MG/KG/DAY	10 MG/KG/DAY	100 MG/KG/DAY
CD14+ (%)					
DAY -14	MEAN	51.7	59.5	53.5	48.2
	% DIFFERENCE		15.1	3.5	-6.8
	S.D.	7.27	20.68	7.33	11.88
	N	5	3	3	5
DAY 31	MEAN	44.4	46.5	45.5	57.7
	% DIFFERENCE		4.7	2.5	30.0
	S.D.	18.68	10.46	15.31	9.18
	N	5	3	3	5
DAY 71	MEAN	61.6	NA	NA	65.5
	% DIFFERENCE				6.3
	S.D.	5.87			4.45
	N	2			2

thous/uL = THOUSANDS/MICROLITER

None significantly different from control group
NA = NOT APPLICABLE

Appendix 19

SUMMARY OF IMMUNOPHENOTYPING VALUES

ANALYSIS	GROUP:	MALES 0 MG/KG/DAY	3 MG/KG/DAY	10 MG/KG/DAY	100 MG/KG/DAY
CD3+ ABS (thous/uL)					
DAY -14	MEAN	2.66	2.31	3.17	3.20
	% DIFFERENCE		-13.2	19.2	20.3
	S.D.	0.782	0.130	0.093	0.738
	N	5	3	3	5
DAY 31	MEAN	3.26	3.65	3.88	3.67
	% DIFFERENCE		12.0	19.0	12.6
	S.D.	0.955	0.720	0.467	0.809
	N	5	3	3	5
DAY 71	MEAN	3.20	NA	NA	3.13
	% DIFFERENCE				-2.2
	S.D.	0.339			0.057
	N	2			2

thous/uL = THOUSANDS/MICROLITER

None significantly different from control group
NA = NOT APPLICABLE

Appendix 19

SUMMARY OF IMMUNOPHENOTYPING VALUES

ANALYSIS	GROUP:	0 MG/KG/DAY	MALES<br>3 MG/KG/DAY	10 MG/KG/DAY	100 MG/KG/DAY
CD3+CD4+ABS (thous/uL)					
DAY -14	MEAN	1.53	1.19	1.68	1.58
	% DIFFERENCE		-22.2	9.8	3.3
	S.D.	0.433	0.163	0.142	0.277
	N	5	3	3	5
DAY 31	MEAN	1.83	1.85	2.11	2.08
	% DIFFERENCE		1.1	15.3	13.7
	S.D.	0.499	0.485	0.162	0.574
	N	5	3	3	5
DAY 71	MEAN	1.63	NA	NA	1.90
	% DIFFERENCE				16.6
	S.D.	0.035			0.191
	N	2			2

thous/uL = THOUSANDS/MICROLITER

None significantly different from control group
NA = NOT APPLICABLE

Appendix 19

SUMMARY OF IMMUNOPHENOTYPING VALUES

ANALYSIS	GROUP:	0 MG/KG/DAY	3 MG/KG/DAY	10 MG/KG/DAY	100 MG/KG/DAY
			MALES		
CD3+CD8+ABS (thous/uL)					
DAY -14	MEAN	1.04	0.98	1.35	1.41
	% DIFFERENCE		-5.8	29.8	35.6
	S.D.	0.401	0.060	0.229	0.666
	N	5	3	3	5
DAY 31	MEAN	1.33	1.61	1.68	1.42
	% DIFFERENCE		21.1	26.3	6.8
	S.D.	0.497	0.389	0.415	0.264
	N	5	3	3	5
DAY 71	MEAN	1.46	NA	NA	1.09
	% DIFFERENCE				-25.3
	S.D.	0.156			0.120
	N	2			2

thous/uL = THOUSANDS/MICROLITER

None significantly different from control group
NA = NOT APPLICABLE

Appendix 19

SUMMARY OF IMMUNOPHENOTYPING VALUES

ANALYSIS	GROUP:	0 MG/KG/DAY	3 MG/KG/DAY	10 MG/KG/DAY	100 MG/KG/DAY
			MALES		
CD3-CD20+ ABS (thous/uL)					
DAY -14	MEAN	0.69	0.54	0.66	0.58
	% DIFFERENCE		-21.7	-4.3	-15.9
	S.D.	0.227	0.056	0.216	0.137
	N	5	3	3	5
DAY 31	MEAN	0.67	0.65	0.59	0.54
	% DIFFERENCE		-3.0	-11.9	-19.4
	S.D.	0.240	0.076	0.180	0.114
	N	5	3	3	5
DAY 71	MEAN	0.66	NA	NA	0.61
	% DIFFERENCE				-7.6
	S.D.	0.000			0.028
	N	2			2

thous/uL = THOUSANDS/MICROLITER

None significantly different from control group
NA = NOT APPLICABLE

Appendix 19

SUMMARY OF IMMUNOPHENOTYPING VALUES

ANALYSIS	GROUP:	MALES 0 MG/KG/DAY	3 MG/KG/DAY	10 MG/KG/DAY	100 MG/KG/DAY
CD3-CD16+ ABS (thous/uL)					
DAY -14	MEAN	0.26	0.59	0.28	0.40
	% DIFFERENCE		126.9	7.7	53.8
	S.D.	0.046	0.155	0.084	0.258
	N	5	3	3	5
DAY 31	MEAN	0.09	0.11	0.07	0.12
	% DIFFERENCE		22.2	-22.2	33.3
	S.D.	0.086	0.070	0.012	0.052
	N	5	3	3	5
DAY 71	MEAN	0.12	NA	NA	0.09
	% DIFFERENCE				-25.0
	S.D.	0.113			0.028
	N	2			2

thous/uL = THOUSANDS/MICROLITER

None significantly different from control group
NA = NOT APPLICABLE

Appendix 19

SUMMARY OF IMMUNOPHENOTYPING VALUES

ANALYSIS	GROUP:	MALES 0 MG/KG/DAY	3 MG/KG/DAY	10 MG/KG/DAY	100 MG/KG/DAY
CD14+ ABS (thous/uL)					
DAY -14	MEAN	0.17	0.31	0.25	0.23
	% DIFFERENCE		82.4	47.1	35.3
	S.D.	0.073	0.197	0.136	0.073
	N	5	3	3	5
DAY 31	MEAN	0.17	0.16	0.17	0.28
	% DIFFERENCE		-5.9	0.0	64.7
	S.D.	0.102	0.040	0.119	0.079
	N	5	3	3	5
DAY 71	MEAN	0.26	NA	NA	0.32
	% DIFFERENCE				23.1
	S.D.	0.042			0.106
	N	2			2

thous/uL = THOUSANDS/MICROLITER

None significantly different from control group
NA = NOT APPLICABLE

Appendix 19

SUMMARY OF IMMUNOPHENOTYPING VALUES

ANALYSIS	GROUP:	FEMALES 0 MG/KG/DAY	3 MG/KG/DAY	10 MG/KG/DAY	100 MG/KG/DAY
CD3+ (%)					
DAY -14	MEAN	54.9	49.6	41.2	49.2
	% DIFFERENCE		-9.7	-25.0	-10.4
	S.D.	10.08	9.26	2.75	10.86
	N	5	3	3	5
DAY 31	MEAN	59.0	58.8	52.8	63.0
	% DIFFERENCE		-0.3	-10.5	6.8
	S.D.	9.41	6.41	1.47	5.09
	N	5	3	3	5
DAY 71	MEAN	61.3	NA	NA	52.0
	% DIFFERENCE				-15.2
	S.D.	3.54			7.99
	N	2			2

thous/uL = THOUSANDS/MICROLITER

None significantly different from control group
NA = NOT APPLICABLE

Appendix 19

SUMMARY OF IMMUNOPHENOTYPING VALUES

ANALYSIS GROUP:		FEMALES 0 MG/KG/DAY	3 MG/KG/DAY	10 MG/KG/DAY	100 MG/KG/DAY
CD3+CD4+ (%)					
DAY -14	MEAN	28.1	29.2	22.1	26.1
	% DIFFERENCE		3.9	-21.4	-7.1
	S.D.	6.10	6.18	4.95	4.63
	N	5	3	3	5
DAY 31	MEAN	30.9	35.8	30.1	33.6
	% DIFFERENCE		15.9	-2.6	8.7
	S.D.	8.68	5.76	1.90	2.25
	N	5	3	3	5
DAY 71	MEAN	30.7	NA	NA	26.0
	% DIFFERENCE				-15.3
	S.D.	9.26			0.28
	N	2			2

thous/uL = THOUSANDS/MICROLITER

None significantly different from control group
NA = NOT APPLICABLE

Appendix 19

SUMMARY OF IMMUNOPHENOTYPING VALUES

ANALYSIS	GROUP:	FEMALES 0 MG/KG/DAY	3 MG/KG/DAY	10 MG/KG/DAY	100 MG/KG/DAY
CD3+CD8+ (%)					
DAY -14	MEAN	24.5	17.8	16.7	20.4
	% DIFFERENCE		-27.3	-31.8	-16.7
	S.D.	8.99	2.08	3.44	6.32
	N	5	3	3	5
DAY 31	MEAN	25.4	20.3	19.9	25.7
	% DIFFERENCE		-20.1	-21.7	1.2
	S.D.	6.69	1.21	0.93	4.46
	N	5	3	3	5
DAY 71	MEAN	28.5	NA	NA	22.9
	% DIFFERENCE				-19.6
	S.D.	2.19			6.29
	N	2			2

thous/uL = THOUSANDS/MICROLITER

None significantly different from control group
NA = NOT APPLICABLE

Appendix 19

SUMMARY OF IMMUNOPHENOTYPING VALUES

ANALYSIS	GROUP:	FEMALES 0 MG/KG/DAY	3 MG/KG/DAY	10 MG/KG/DAY	100 MG/KG/DAY
CD3-CD20+ (%)					
DAY -14	MEAN	14.3	16.5	13.2	13.5
	% DIFFERENCE		15.4	-7.7	-5.6
	S.D.	1.78	0.67	6.44	5.58
	N	5	3	3	5
DAY 31	MEAN	12.4	14.0	10.9	12.1
	% DIFFERENCE		12.9	-12.1	-2.4
	S.D.	2.80	2.59	5.91	3.00
	N	5	3	3	5
DAY 71	MEAN	11.7	NA	NA	12.8
	% DIFFERENCE				9.4
	S.D.	1.70			5.16
	N	2			2

thous/uL = THOUSANDS/MICROLITER

None significantly different from control group
NA = NOT APPLICABLE

Appendix 19

SUMMARY OF IMMUNOPHENOTYPING VALUES

ANALYSIS	GROUP:	FEMALES 0 MG/KG/DAY	3 MG/KG/DAY	10 MG/KG/DAY	100 MG/KG/DAY
CD3-CD16+ (%)					
DAY -14	MEAN	8.5	14.1	25.4**	6.1
	% DIFFERENCE		65.9	198.8	-28.2
	S.D.	6.59	10.64	3.10	4.59
	N	5	3	3	5
DAY 31	MEAN	3.7	2.4	4.3	1.3
	% DIFFERENCE		-35.1	16.2	-64.9
	S.D.	2.92	1.05	2.26	0.69
	N	5	3	3	5
DAY 71	MEAN	1.0	NA	NA	3.7
	% DIFFERENCE				270.0
	S.D.	0.49			1.98
	N	2			2

thous/uL = THOUSANDS/MICROLITER

** = Significantly different from the control group at 0.01 using Dunnett's test
NA = NOT APPLICABLE

Appendix 19

SUMMARY OF IMMUNOPHENOTYPING VALUES

ANALYSIS	GROUP:	FEMALES 0 MG/KG/DAY	3 MG/KG/DAY	10 MG/KG/DAY	100 MG/KG/DAY
CD14+ (%)					
DAY -14	MEAN	45.6	54.3	40.3	53.6
	% DIFFERENCE		19.1	-11.6	17.5
	S.D.	9.63	18.91	6.99	19.04
	N	5	3	3	5
DAY 31	MEAN	56.0	42.9	53.4	59.2
	% DIFFERENCE		-23.4	-4.6	5.7
	S.D.	20.98	14.46	17.99	4.52
	N	5	3	3	5
DAY 71	MEAN	53.5	NA	NA	78.7
	% DIFFERENCE				47.1
	S.D.	12.02			0.50
	N	2			2

thous/uL = THOUSANDS/MICROLITER

None significantly different from control group
NA = NOT APPLICABLE

Appendix 19

SUMMARY OF IMMUNOPHENOTYPING VALUES

ANALYSIS	GROUP:	FEMALES 0 MG/KG/DAY	3 MG/KG/DAY	10 MG/KG/DAY	100 MG/KG/DAY
CD3+ ABS (thous/uL)					
DAY -14	MEAN	2.20	2.00	1.61	1.93
	% DIFFERENCE		-9.1	-26.8	-12.3
	S.D.	0.610	0.854	0.280	0.394
	N	5	3	3	5
DAY 31	MEAN	2.85	2.61	2.98	3.57
	% DIFFERENCE		-8.4	4.6	25.3
	S.D.	0.747	0.913	0.839	0.931
	N	5	3	3	5
DAY 71	MEAN	3.50	NA	NA	2.17
	% DIFFERENCE				-38.0
	S.D.	0.304			0.057
	N	2			2

thous/uL = THOUSANDS/MICROLITER

None significantly different from control group
NA = NOT APPLICABLE

Appendix 19

SUMMARY OF IMMUNOPHENOTYPING VALUES

ANALYSIS	GROUP:	0 MG/KG/DAY	FEMALES<br>3 MG/KG/DAY	10 MG/KG/DAY	100 MG/KG/DAY
CD3+CD4+ABS (thous/uL)					
DAY -14	MEAN	1.12	1.18	0.86	1.02
	% DIFFERENCE		5.4	-23.2	-8.9
	S.D.	0.312	0.528	0.202	0.102
	N	5	3	3	5
DAY 31	MEAN	1.48	1.60	1.72	1.95
	% DIFFERENCE		8.1	16.2	31.8
	S.D.	0.489	0.620	0.552	0.666
	N	5	3	3	5
DAY 71	MEAN	1.75	NA	NA	1.10
	% DIFFERENCE				-37.1
	S.D.	0.580			0.120
	N	2			2

thous/uL = THOUSANDS/MICROLITER

None significantly different from control group
NA = NOT APPLICABLE

Appendix 19

SUMMARY OF IMMUNOPHENOTYPING VALUES

ANALYSIS	GROUP:	0 MG/KG/DAY	FEMALES<br>3 MG/KG/DAY	10 MG/KG/DAY	100 MG/KG/DAY
CD3+CD8+ABS (thous/uL)					
DAY -14	MEAN	0.99	0.70	0.66	0.81
	% DIFFERENCE		-29.3	-33.3	-18.2
	S.D.	0.444	0.225	0.204	0.283
	N	5	3	3	5
DAY 31	MEAN	1.22	0.89	1.11	1.43
	% DIFFERENCE		-27.0	-9.0	17.2
	S.D.	0.458	0.243	0.263	0.276
	N	5	3	3	5
DAY 71	MEAN	1.62	NA	NA	0.95
	% DIFFERENCE				-41.4
	S.D.	0.078			0.148
	N	2			2

thous/uL = THOUSANDS/MICROLITER

None significantly different from control group
NA = NOT APPLICABLE

Appendix 19

SUMMARY OF IMMUNOPHENOTYPING VALUES

ANALYSIS	GROUP:	FEMALES<br>0 MG/KG/DAY	3 MG/KG/DAY	10 MG/KG/DAY	100 MG/KG/DAY
CD3-CD20+ ABS (thous/uL)					
DAY -14	MEAN	0.57	0.65	0.50	0.55
	% DIFFERENCE		14.0	-12.3	-3.5
	S.D.	0.097	0.133	0.217	0.292
	N	5	3	3	5
DAY 31	MEAN	0.59	0.59	0.58	0.72
	% DIFFERENCE		0.0	-1.7	22.0
	S.D.	0.166	0.017	0.347	0.328
	N	5	3	3	5
DAY 71	MEAN	0.67	NA	NA	0.55
	% DIFFERENCE				-17.9
	S.D.	0.113			0.283
	N	2			2

thous/uL = THOUSANDS/MICROLITER

None significantly different from control group
NA = NOT APPLICABLE

Appendix 19

SUMMARY OF IMMUNOPHENOTYPING VALUES

			FEMALES		
ANALYSIS	GROUP:	0 MG/KG/DAY	3 MG/KG/DAY	10 MG/KG/DAY	100 MG/KG/DAY
CD3-CD16+ ABS (thous/uL)					
DAY -14	MEAN	0.31	0.51	0.99**	0.23
	% DIFFERENCE		64.5	219.4	-25.8
	S.D.	0.220	0.344	0.198	0.130
	N	5	3	3	5
DAY 31	MEAN	0.18	0.10	0.22	0.08
	% DIFFERENCE		-44.4	22.2	-55.6
	S.D.	0.134	0.026	0.050	0.069
	N	5	3	3	5
DAY 71	MEAN	0.06	NA	NA	0.16
	% DIFFERENCE				166.7
	S.D.	0.035			0.064
	N	2			2

thous/uL = THOUSANDS/MICROLITER

** = Significantly different from the control group at 0.01 using Dunnett's test
NA = NOT APPLICABLE

Appendix 19

SUMMARY OF IMMUNOPHENOTYPING VALUES

ANALYSIS	GROUP:	0 MG/KG/DAY	FEMALES<br>3 MG/KG/DAY	10 MG/KG/DAY	100 MG/KG/DAY
CD14+ ABS (thous/uL)					
DAY -14	MEAN	0.14	0.18	0.17	0.24
	% DIFFERENCE		28.6	21.4	71.4
	S.D.	0.056	0.101	0.060	0.167
	N	5	3	3	5
DAY 31	MEAN	0.25	0.15	0.29	0.29
	% DIFFERENCE		-40.0	16.0	16.0
	S.D.	0.169	0.080	0.125	0.156
	N	5	3	3	5
DAY 71	MEAN	0.18	NA	NA	0.42
	% DIFFERENCE				133.3
	S.D.	0.035			0.049
	N	2			2

thous/uL = THOUSANDS/MICROLITER

None significantly different from control group
NA = NOT APPLICABLE

PCPSv5.39
01/10/2023
R:01/10/2023

Appendix 19

INDIVIDUAL IMMUNOPHENOTYPING VALUES (DAY -14)

DAY -14

ANIMAL	CD3+	CD3+CD4+	CD3+CD8+	CD3-CD20+	CD3-CD16+	CD14+
	%	%	%	%	%	%
GROUP: 0 MG/KG/DAY MALES						
1001	57.3	33.9	19.9	15.5	8.3	46.4
1002	64.1	37.0	27.1	11.7	5.6	52.0
1003	50.8	30.3	16.7	23.0	4.8	50.0
1004	57.7	33.9	22.4	16.0	6.2	63.9
1005	58.1	31.1	24.4	10.9	4.9	46.1
MEAN	57.6	33.2	22.1	15.4	6.0	51.7
S.D.	4.71	2.66	4.01	4.80	1.43	7.27
N	5	5	5	5	5	5

Appendix 19

INDIVIDUAL IMMUNOPHENOTYPING VALUES (DAY -14)

DAY -14

ANIMAL	CD3+ ABS	CD3+CD4+ ABS	CD3+CD8+ ABS	CD3-CD20 + ABS	CD3-CD16 + ABS	CD14+ ABS
	thous/uL	thous/uL	thous/uL	thous/uL	thous/uL	thous/uL
GROUP: 0 MG/KG/DAY MALES						
1001	1.86	1.10	0.65	0.50	0.27	0.07
1002	3.90	2.25	1.65	0.71	0.34	0.21
1003	2.36	1.41	0.78	1.07	0.22	0.13
1004	2.30	1.35	0.89	0.64	0.25	0.26
1005	2.88	1.54	1.21	0.54	0.24	0.18
MEAN	2.66	1.53	1.04	0.69	0.26	0.17
S.D.	0.782	0.433	0.401	0.227	0.046	0.073
N	5	5	5	5	5	5

thous/uL = THOUSANDS/MICROLITER

Appendix 19

INDIVIDUAL IMMUNOPHENOTYPING VALUES (DAY -14)

DAY -14

ANIMAL	CD3+	CD3+CD4+	CD3+CD8+	CD3-CD20+	CD3-CD16+	CD14+
	%	%	%	%	%	%
GROUP: 3 MG/KG/DAY MALES						
2001	58.4	29.7	27.3	12.7	11.1	82.5
2002	57.7	32.2	22.6	12.4	15.1	42.5
2003	51.8	24.6	21.4	13.9	16.6	53.4
MEAN	56.0	28.8	23.8	13.0	14.3	59.5
S.D.	3.63	3.87	3.12	0.79	2.84	20.68
N	3	3	3	3	3	3

Appendix 19

INDIVIDUAL IMMUNOPHENOTYPING VALUES (DAY -14)

DAY -14

ANIMAL	CD3+ ABS	CD3+CD4+ ABS	CD3+CD8+ ABS	CD3-CD20 + ABS	CD3-CD16 + ABS	CD14+ ABS
	thous/uL	thous/uL	thous/uL	thous/uL	thous/uL	thous/uL
GROUP: 3 MG/KG/DAY MALES						
2001	2.23	1.13	1.04	0.49	0.42	0.54
2002	2.46	1.37	0.97	0.53	0.64	0.19
2003	2.24	1.06	0.92	0.60	0.72	0.21
MEAN	2.31	1.19	0.98	0.54	0.59	0.31
S.D.	0.130	0.163	0.060	0.056	0.155	0.197
N	3	3	3	3	3	3

thous/uL = THOUSANDS/MICROLITER

Appendix 19

INDIVIDUAL IMMUNOPHENOTYPING VALUES (DAY -14)

DAY -14

ANIMAL	CD3+	CD3+CD4+	CD3+CD8+	CD3-CD20+	CD3-CD16+	CD14+
	%	%	%	%	%	%
GROUP: 10 MG/KG/DAY MALES						
3001	59.8	29.6	28.6	13.3	7.4	55.8
3002	54.4	29.6	18.3	7.2	3.8	59.4
3003	56.3	31.0	26.5	15.0	4.2	45.3
MEAN	56.8	30.1	24.5	11.8	5.1	53.5
S.D.	2.74	0.81	5.44	4.10	1.97	7.33
N	3	3	3	3	3	3

Appendix 19

INDIVIDUAL IMMUNOPHENOTYPING VALUES (DAY -14)

DAY -14

ANIMAL	CD3+ ABS	CD3+CD4+ ABS	CD3+CD8+ ABS	CD3-CD20 + ABS	CD3-CD16 + ABS	CD14+ ABS
	thous/uL	thous/uL	thous/uL	thous/uL	thous/uL	thous/uL
GROUP: 10 MG/KG/DAY MALES						
3001	3.06	1.52	1.46	0.68	0.38	0.20
3002	3.23	1.76	1.09	0.43	0.23	0.40
3003	3.21	1.77	1.51	0.86	0.24	0.14
MEAN	3.17	1.68	1.35	0.66	0.28	0.25
S.D.	0.093	0.142	0.229	0.216	0.084	0.136
N	3	3	3	3	3	3

thous/uL = THOUSANDS/MICROLITER

Appendix 19

INDIVIDUAL IMMUNOPHENOTYPING VALUES (DAY -14)

DAY -14

ANIMAL	CD3+	CD3+CD4+	CD3+CD8+	CD3-CD20+	CD3-CD16+	CD14+
	%	%	%	%	%	%
GROUP: 100 MG/KG/DAY MALES						
4001	62.6	31.8	22.7	8.2	3.6	37.6
4002	52.3	16.0	31.8	9.0	5.6	38.4
4003	52.8	28.6	23.6	10.5	1.9	60.3
4004	60.2	37.3	20.7	14.4	10.4	43.0
4005	57.1	33.5	21.8	10.6	16.3	61.7
MEAN	57.0	29.4	24.1	10.5	7.6	48.2
S.D.	4.51	8.14	4.43	2.38	5.83	11.88
N	5	5	5	5	5	5

Appendix 19

INDIVIDUAL IMMUNOPHENOTYPING VALUES (DAY -14)

DAY -14

ANIMAL	CD3+ ABS	CD3+CD4+ ABS	CD3+CD8+ ABS	CD3-CD20 + ABS	CD3-CD16 + ABS	CD14+ ABS
	thous/uL	thous/uL	thous/uL	thous/uL	thous/uL	thous/uL
GROUP: 100 MG/KG/DAY MALES						
4001	3.62	1.84	1.31	0.47	0.21	0.19
4002	4.24	1.30	2.58	0.73	0.45	0.17
4003	2.46	1.33	1.10	0.49	0.09	0.33
4004	3.05	1.89	1.05	0.73	0.53	0.17
4005	2.61	1.53	1.00	0.48	0.74	0.28
MEAN	3.20	1.58	1.41	0.58	0.40	0.23
S.D.	0.738	0.277	0.666	0.137	0.258	0.073
N	5	5	5	5	5	5

thous/uL = THOUSANDS/MICROLITER

Appendix 19

INDIVIDUAL IMMUNOPHENOTYPING VALUES (DAY -14)

DAY -14

ANIMAL	CD3+	CD3+CD4+	CD3+CD8+	CD3-CD20+	CD3-CD16+	CD14+
	%	%	%	%	%	%
GROUP: 0 MG/KG/DAY FEMALES						
1501	42.7	20.6	18.3	14.1	17.3	35.2
1502	68.6	29.4	37.6	13.8	0.9	51.5
1503	48.3	31.9	14.7	14.0	10.4	47.1
1504	60.0	35.4	23.3	17.3	10.7	57.7
1505	55.1	23.3	28.5	12.5	3.0	36.7
MEAN	54.9	28.1	24.5	14.3	8.5	45.6
S.D.	10.08	6.10	8.99	1.78	6.59	9.63
N	5	5	5	5	5	5

Appendix 19

INDIVIDUAL IMMUNOPHENOTYPING VALUES (DAY -14)

DAY -14

ANIMAL	CD3+ ABS	CD3+CD4+ ABS	CD3+CD8+ ABS	CD3-CD20 + ABS	CD3-CD16 + ABS	CD14+ ABS
	thous/uL	thous/uL	thous/uL	thous/uL	thous/uL	thous/uL
GROUP: 0 MG/KG/DAY FEMALES						
1501	1.37	0.66	0.59	0.45	0.55	0.08
1502	3.00	1.28	1.64	0.60	0.04	0.22
1503	1.90	1.25	0.58	0.55	0.41	0.15
1504	2.45	1.45	0.95	0.71	0.44	0.17
1505	2.28	0.96	1.18	0.52	0.12	0.10
MEAN	2.20	1.12	0.99	0.57	0.31	0.14
S.D.	0.610	0.312	0.444	0.097	0.220	0.056
N	5	5	5	5	5	5

thous/uL = THOUSANDS/MICROLITER

Appendix 19

INDIVIDUAL IMMUNOPHENOTYPING VALUES (DAY -14)

DAY -14

ANIMAL	CD3+	CD3+CD4+	CD3+CD8+	CD3-CD20+	CD3-CD16+	CD14+
	%	%	%	%	%	%
GROUP: 3 MG/KG/DAY FEMALES						
2501	44.9	24.5	18.6	16.2	26.1	76.1
2502	43.7	26.9	15.4	17.3	10.2	43.5
2503	60.3	36.2	19.3	16.1	5.9	43.2
MEAN	49.6	29.2	17.8	16.5	14.1	54.3
S.D.	9.26	6.18	2.08	0.67	10.64	18.91
N	3	3	3	3	3	3

Appendix 19

INDIVIDUAL IMMUNOPHENOTYPING VALUES (DAY -14)

DAY -14

ANIMAL	CD3+ ABS	CD3+CD4+ ABS	CD3+CD8+ ABS	CD3-CD20 + ABS	CD3-CD16 + ABS	CD14+ ABS
	thous/uL	thous/uL	thous/uL	thous/uL	thous/uL	thous/uL
GROUP: 3 MG/KG/DAY FEMALES						
2501	1.57	0.86	0.65	0.57	0.91	0.30
2502	1.44	0.89	0.51	0.57	0.34	0.13
2503	2.98	1.79	0.95	0.80	0.29	0.12
MEAN	2.00	1.18	0.70	0.65	0.51	0.18
S.D.	0.854	0.528	0.225	0.133	0.344	0.101
N	3	3	3	3	3	3

thous/uL = THOUSANDS/MICROLITER

Appendix 19

INDIVIDUAL IMMUNOPHENOTYPING VALUES (DAY -14)

DAY -14

ANIMAL	CD3+	CD3+CD4+	CD3+CD8+	CD3-CD20+	CD3-CD16+	CD14+
	%	%	%	%	%	%
GROUP: 10 MG/KG/DAY FEMALES						
3501	41.3	17.1	20.7	14.4	23.9	45.0
3502	38.4	22.2	14.9	18.9	23.4	43.7
3503	43.9	27.0	14.6	6.2	29.0	32.3
MEAN	41.2	22.1	16.7	13.2	25.4	40.3
S.D.	2.75	4.95	3.44	6.44	3.10	6.99
N	3	3	3	3	3	3

Appendix 19

INDIVIDUAL IMMUNOPHENOTYPING VALUES (DAY -14)

DAY -14

ANIMAL	CD3+ ABS	CD3+CD4+ ABS	CD3+CD8+ ABS	CD3-CD20 + ABS	CD3-CD16 + ABS	CD14+ ABS
	thous/uL	thous/uL	thous/uL	thous/uL	thous/uL	thous/uL
GROUP: 10 MG/KG/DAY FEMALES						
3501	1.78	0.74	0.89	0.62	1.03	0.23
3502	1.29	0.74	0.50	0.63	0.78	0.16
3503	1.77	1.09	0.59	0.25	1.17	0.11
MEAN	1.61	0.86	0.66	0.50	0.99	0.17
S.D.	0.280	0.202	0.204	0.217	0.198	0.060
N	3	3	3	3	3	3

thous/uL = THOUSANDS/MICROLITER

Appendix 19

INDIVIDUAL IMMUNOPHENOTYPING VALUES (DAY -14)

DAY -14

ANIMAL	CD3+	CD3+CD4+	CD3+CD8+	CD3-CD20+	CD3-CD16+	CD14+
	%	%	%	%	%	%
GROUP: 100 MG/KG/DAY FEMALES						
4501	56.0	32.5	20.9	18.8	6.2	28.9
4502	35.3	21.8	13.0	9.8	4.3	75.1
4503	58.2	29.4	23.6	8.7	14.0	69.8
4504	39.7	22.5	15.7	20.3	3.7	50.9
4505	57.0	24.5	28.9	9.8	2.5	43.2
MEAN	49.2	26.1	20.4	13.5	6.1	53.6
S.D.	10.86	4.63	6.32	5.58	4.59	19.04
N	5	5	5	5	5	5

Appendix 19

INDIVIDUAL IMMUNOPHENOTYPING VALUES (DAY -14)

DAY -14

ANIMAL	CD3+ ABS	CD3+CD4+ ABS	CD3+CD8+ ABS	CD3-CD20 + ABS	CD3-CD16 + ABS	CD14+ ABS
	thous/uL	thous/uL	thous/uL	thous/uL	thous/uL	thous/uL
GROUP: 100 MG/KG/DAY FEMALES						
4501	1.79	1.04	0.67	0.60	0.20	0.04
4502	1.45	0.89	0.53	0.40	0.18	0.40
4503	1.87	0.95	0.76	0.28	0.45	0.41
4504	2.02	1.14	0.80	1.03	0.19	0.23
4505	2.53	1.09	1.28	0.44	0.11	0.11
MEAN	1.93	1.02	0.81	0.55	0.23	0.24
S.D.	0.394	0.102	0.283	0.292	0.130	0.167
N	5	5	5	5	5	5

thous/uL = THOUSANDS/MICROLITER

PCHEv4.14
01/10/2023
R:01/10/2023

Appendix 19

INDIVIDUAL IMMUNOPHENOTYPING VALUES (DAY 31)

ANIMAL	CD3+	CD3+CD4+	CD3+CD8+	CD3-CD20+	CD3-CD16+	CD14+
	%	%	%	%	%	%
GROUP: 0 MG/KG/DAY MALES						
1001	65.9	39.7	23.0	13.4	1.0	31.3
1002	65.9	36.1	29.1	10.3	1.8	29.2
1003	55.4	32.1	19.8	19.3	4.0	34.4
1004	64.2	36.2	26.9	11.3	0.7	54.3
1005	64.8	34.0	27.9	11.0	0.5	72.6
MEAN	63.2	35.6	25.3	13.1	1.6	44.4
S.D.	4.44	2.84	3.85	3.67	1.43	18.68
N	5	5	5	5	5	5

Appendix 19

INDIVIDUAL IMMUNOPHENOTYPING VALUES (DAY 31)

ANIMAL	CD3+ ABS	CD3+CD4+ ABS	CD3+CD8+ ABS	CD3-CD20 + ABS	CD3-CD16 + ABS	CD14+ ABS
	thous/uL	thous/uL	thous/uL	thous/uL	thous/uL	thous/uL
GROUP: 0 MG/KG/DAY MALES						
1001	2.14	1.29	0.75	0.44	0.03	0.06
1002	4.67	2.56	2.06	0.73	0.13	0.11
1003	2.98	1.73	1.07	1.04	0.22	0.11
1004	3.66	2.06	1.53	0.64	0.04	0.27
1005	2.85	1.50	1.23	0.48	0.02	0.28
MEAN	3.26	1.83	1.33	0.67	0.09	0.17
S.D.	0.955	0.499	0.497	0.240	0.086	0.102
N	5	5	5	5	5	5

thous/uL = THOUSANDS/MICROLITER

Appendix 19

INDIVIDUAL IMMUNOPHENOTYPING VALUES (DAY 31)

ANIMAL	CD3+	CD3+CD4+	CD3+CD8+	CD3-CD20+	CD3-CD16+	CD14+
	%	%	%	%	%	%
GROUP: 3 MG/KG/DAY MALES						
2001	68.7	34.5	33.4	13.1	2.7	43.1
2002	66.3	35.4	28.4	10.3	0.5	38.1
2003	61.1	28.4	24.3	11.7	3.3	58.2
MEAN	65.4	32.8	28.7	11.7	2.2	46.5
S.D.	3.89	3.81	4.56	1.40	1.47	10.46
N	3	3	3	3	3	3

Appendix 19

INDIVIDUAL IMMUNOPHENOTYPING VALUES (DAY 31)

ANIMAL	CD3+ ABS	CD3+CD4+ ABS	CD3+CD8+ ABS	CD3-CD20 + ABS	CD3-CD16 + ABS	CD14+ ABS
	thous/uL	thous/uL	thous/uL	thous/uL	thous/uL	thous/uL
GROUP: 3 MG/KG/DAY MALES						
2001	3.68	1.85	1.79	0.70	0.14	0.16
2002	4.36	2.33	1.87	0.68	0.03	0.12
2003	2.92	1.36	1.16	0.56	0.16	0.20
MEAN	3.65	1.85	1.61	0.65	0.11	0.16
S.D.	0.720	0.485	0.389	0.076	0.070	0.040
N	3	3	3	3	3	3

thous/uL = THOUSANDS/MICROLITER

Appendix 19

INDIVIDUAL IMMUNOPHENOTYPING VALUES (DAY 31)

ANIMAL	CD3+	CD3+CD4+	CD3+CD8+	CD3-CD20+	CD3-CD16+	CD14+
	%	%	%	%	%	%
GROUP: 10 MG/KG/DAY MALES						
3001	67.6	33.8	32.1	10.2	1.2	38.5
3002	74.8	41.2	27.0	8.4	1.2	63.1
3003	66.8	39.0	29.8	12.9	1.4	35.0
MEAN	69.7	38.0	29.6	10.5	1.3	45.5
S.D.	4.41	3.80	2.55	2.26	0.12	15.31
N	3	3	3	3	3	3

Appendix 19

INDIVIDUAL IMMUNOPHENOTYPING VALUES (DAY 31)

ANIMAL	CD3+ ABS	CD3+CD4+ ABS	CD3+CD8+ ABS	CD3-CD20 + ABS	CD3-CD16 + ABS	CD14+ ABS
	thous/uL	thous/uL	thous/uL	thous/uL	thous/uL	thous/uL
GROUP: 10 MG/KG/DAY MALES						
3001	4.40	2.20	2.09	0.66	0.08	0.13
3002	3.49	1.92	1.26	0.39	0.06	0.30
3003	3.76	2.20	1.68	0.73	0.08	0.07
MEAN	3.88	2.11	1.68	0.59	0.07	0.17
S.D.	0.467	0.162	0.415	0.180	0.012	0.119
N	3	3	3	3	3	3

thous/uL = THOUSANDS/MICROLITER

Appendix 19

INDIVIDUAL IMMUNOPHENOTYPING VALUES (DAY 31)

ANIMAL	CD3+	CD3+CD4+	CD3+CD8+	CD3-CD20+	CD3-CD16+	CD14+
	%	%	%	%	%	%
GROUP: 100 MG/KG/DAY MALES						
4001	71.4	40.4	26.6	8.4	1.1	60.9
4002	65.2	30.1	30.5	9.3	3.3	62.3
4003	63.6	34.7	27.3	9.5	3.5	62.1
4004	71.2	46.2	22.7	13.4	2.3	41.3
4005	70.1	41.1	26.4	9.8	1.0	61.9
MEAN	68.3	38.5	26.7	10.1	2.2	57.7
S.D.	3.64	6.22	2.78	1.93	1.18	9.18
N	5	5	5	5	5	5

Appendix 19

INDIVIDUAL IMMUNOPHENOTYPING VALUES (DAY 31)

ANIMAL	CD3+ ABS	CD3+CD4+ ABS	CD3+CD8+ ABS	CD3-CD20 + ABS	CD3-CD16 + ABS	CD14+ ABS
	thous/uL	thous/uL	thous/uL	thous/uL	thous/uL	thous/uL
GROUP: 100 MG/KG/DAY MALES						
4001	5.02	2.84	1.87	0.59	0.08	0.38
4002	2.98	1.38	1.39	0.43	0.15	0.29
4003	3.21	1.75	1.38	0.48	0.18	0.26
4004	3.77	2.44	1.20	0.71	0.12	0.16
4005	3.36	1.97	1.26	0.47	0.05	0.30
MEAN	3.67	2.08	1.42	0.54	0.12	0.28
S.D.	0.809	0.574	0.264	0.114	0.052	0.079
N	5	5	5	5	5	5

thous/uL = THOUSANDS/MICROLITER

Appendix 19

INDIVIDUAL IMMUNOPHENOTYPING VALUES (DAY 31)

ANIMAL	CD3+	CD3+CD4+	CD3+CD8+	CD3-CD20+	CD3-CD16+	CD14+
	%	%	%	%	%	%
GROUP: 0 MG/KG/DAY FEMALES						
1501	42.8	20.3	20.2	9.4	7.8	86.3
1502	64.7	33.6	29.4	12.4	0.6	42.8
1503	59.6	41.1	16.4	15.7	5.1	52.3
1504	66.3	36.0	28.8	14.7	1.3	66.1
1505	61.4	23.7	32.0	9.9	3.5	32.5
MEAN	59.0	30.9	25.4	12.4	3.7	56.0
S.D.	9.41	8.68	6.69	2.80	2.92	20.98
N	5	5	5	5	5	5

Appendix 19

INDIVIDUAL IMMUNOPHENOTYPING VALUES (DAY 31)

ANIMAL	CD3+ ABS	CD3+CD4+ ABS	CD3+CD8+ ABS	CD3-CD20 + ABS	CD3-CD16 + ABS	CD14+ ABS
	thous/uL	thous/uL	thous/uL	thous/uL	thous/uL	thous/uL
GROUP: 0 MG/KG/DAY FEMALES						
1501	1.78	0.84	0.84	0.39	0.32	0.54
1502	2.65	1.37	1.20	0.51	0.02	0.14
1503	3.20	2.21	0.88	0.84	0.27	0.25
1504	2.79	1.52	1.21	0.62	0.05	0.22
1505	3.81	1.47	1.98	0.61	0.22	0.12
MEAN	2.85	1.48	1.22	0.59	0.18	0.25
S.D.	0.747	0.489	0.458	0.166	0.134	0.169
N	5	5	5	5	5	5

thous/uL = THOUSANDS/MICROLITER

Appendix 19

INDIVIDUAL IMMUNOPHENOTYPING VALUES (DAY 31)

ANIMAL	CD3+	CD3+CD4+	CD3+CD8+	CD3-CD20+	CD3-CD16+	CD14+
	%	%	%	%	%	%
GROUP: 3 MG/KG/DAY FEMALES						
2501	54.1	30.3	21.0	14.8	2.4	57.0
2502	56.2	35.4	18.9	16.1	3.5	43.5
2503	66.1	41.8	21.0	11.1	1.4	28.1
MEAN	58.8	35.8	20.3	14.0	2.4	42.9
S.D.	6.41	5.76	1.21	2.59	1.05	14.46
N	3	3	3	3	3	3

Appendix 19

INDIVIDUAL IMMUNOPHENOTYPING VALUES (DAY 31)

ANIMAL	CD3+ ABS	CD3+CD4+ ABS	CD3+CD8+ ABS	CD3-CD20 + ABS	CD3-CD16 + ABS	CD14+ ABS
	thous/uL	thous/uL	thous/uL	thous/uL	thous/uL	thous/uL
GROUP: 3 MG/KG/DAY FEMALES						
2501	2.12	1.19	0.82	0.58	0.09	0.23
2502	2.04	1.29	0.69	0.58	0.13	0.14
2503	3.66	2.31	1.16	0.61	0.08	0.07
MEAN	2.61	1.60	0.89	0.59	0.10	0.15
S.D.	0.913	0.620	0.243	0.017	0.026	0.080
N	3	3	3	3	3	3

thous/uL = THOUSANDS/MICROLITER

Appendix 19

INDIVIDUAL IMMUNOPHENOTYPING VALUES (DAY 31)

ANIMAL	CD3+	CD3+CD4+	CD3+CD8+	CD3-CD20+	CD3-CD16+	CD14+
	%	%	%	%	%	%
GROUP: 10 MG/KG/DAY FEMALES						
3501	52.5	28.3	20.9	12.2	6.9	73.9
3502	51.5	30.0	19.1	16.0	2.8	46.3
3503	54.4	32.1	19.6	4.4	3.2	40.1
MEAN	52.8	30.1	19.9	10.9	4.3	53.4
S.D.	1.47	1.90	0.93	5.91	2.26	17.99
N	3	3	3	3	3	3

Appendix 19

INDIVIDUAL IMMUNOPHENOTYPING VALUES (DAY 31)

ANIMAL	CD3+ ABS	CD3+CD4+ ABS	CD3+CD8+ ABS	CD3-CD20 + ABS	CD3-CD16 + ABS	CD14+ ABS
	thous/uL	thous/uL	thous/uL	thous/uL	thous/uL	thous/uL
GROUP: 10 MG/KG/DAY FEMALES						
3501	2.08	1.12	0.83	0.48	0.27	0.42
3502	3.12	1.82	1.16	0.97	0.17	0.28
3503	3.74	2.21	1.35	0.30	0.22	0.17
MEAN	2.98	1.72	1.11	0.58	0.22	0.29
S.D.	0.839	0.552	0.263	0.347	0.050	0.125
N	3	3	3	3	3	3

thous/uL = THOUSANDS/MICROLITER

Appendix 19

INDIVIDUAL IMMUNOPHENOTYPING VALUES (DAY 31)

ANIMAL	CD3+	CD3+CD4+	CD3+CD8+	CD3-CD20+	CD3-CD16+	CD14+
	%	%	%	%	%	%
GROUP: 100 MG/KG/DAY FEMALES						
4501	63.5	34.3	24.7	15.3	1.7	54.6
4502	55.5	33.0	20.1	11.9	2.3	62.5
4503	68.0	36.5	27.7	8.5	0.9	64.4
4504	60.7	33.9	23.8	15.0	0.6	60.0
4505	67.1	30.3	32.0	10.0	1.0	54.5
MEAN	63.0	33.6	25.7	12.1	1.3	59.2
S.D.	5.09	2.25	4.46	3.00	0.69	4.52
N	5	5	5	5	5	5

Appendix 19

INDIVIDUAL IMMUNOPHENOTYPING VALUES (DAY 31)

ANIMAL	CD3+ ABS	CD3+CD4+ ABS	CD3+CD8+ ABS	CD3-CD20 + ABS	CD3-CD16 + ABS	CD14+ ABS
	thous/uL	thous/uL	thous/uL	thous/uL	thous/uL	thous/uL
GROUP: 100 MG/KG/DAY FEMALES						
4501	2.97	1.61	1.16	0.72	0.08	0.13
4502	4.88	2.90	1.77	1.05	0.20	0.52
4503	2.92	1.57	1.19	0.36	0.04	0.37
4504	4.24	2.37	1.66	1.05	0.04	0.24
4505	2.85	1.29	1.36	0.43	0.04	0.19
MEAN	3.57	1.95	1.43	0.72	0.08	0.29
S.D.	0.931	0.666	0.276	0.328	0.069	0.156
N	5	5	5	5	5	5

thous/uL = THOUSANDS/MICROLITER

PCHEv4.14
11/17/2022
R:11/17/2022

Appendix 19

INDIVIDUAL IMMUNOPHENOTYPING VALUES (DAY 71)

ANIMAL	CD3+	CD3+CD4+	CD3+CD8+	CD3-CD20+	CD3-CD16+	CD14+
	%	%	%	%	%	%
GROUP: 0 MG/KG/DAY MALES						
1004	54.7	29.6	24.9	12.1	3.7	57.4
1005	63.0	30.2	28.7	12.1	0.8	65.7
MEAN	58.9	29.9	26.8	12.1	2.3	61.6
S.D.	5.87	0.42	2.69	0.00	2.05	5.87
N	2	2	2	2	2	2

Appendix 19

INDIVIDUAL IMMUNOPHENOTYPING VALUES (DAY 71)

ANIMAL	CD3+ ABS	CD3+CD4+ ABS	CD3+CD8+ ABS	CD3-CD20 + ABS	CD3-CD16 + ABS	CD14+ ABS
	thous/uL	thous/uL	thous/uL	thous/uL	thous/uL	thous/uL
GROUP: 0 MG/KG/DAY MALES						
1004	2.96	1.60	1.35	0.66	0.20	0.29
1005	3.44	1.65	1.57	0.66	0.04	0.23
MEAN	3.20	1.63	1.46	0.66	0.12	0.26
S.D.	0.339	0.035	0.156	0.000	0.113	0.042
N	2	2	2	2	2	2

thous/uL = THOUSANDS/MICROLITER

Appendix 19

INDIVIDUAL IMMUNOPHENOTYPING VALUES (DAY 71)

ANIMAL	CD3+	CD3+CD4+	CD3+CD8+	CD3-CD20+	CD3-CD16+	CD14+
	%	%	%	%	%	%
GROUP: 100 MG/KG/DAY MALES						
4004	67.4	43.0	21.3	13.3	1.5	62.3
4005	58.1	33.0	21.9	11.1	2.1	68.6
MEAN	62.8	38.0	21.6	12.2	1.8	65.5
S.D.	6.58	7.07	0.42	1.56	0.42	4.45
N	2	2	2	2	2	2

Appendix 19

INDIVIDUAL IMMUNOPHENOTYPING VALUES (DAY 71)

ANIMAL	CD3+ ABS	CD3+CD4+ ABS	CD3+CD8+ ABS	CD3-CD20 + ABS	CD3-CD16 + ABS	CD14+ ABS
	thous/uL	thous/uL	thous/uL	thous/uL	thous/uL	thous/uL
GROUP: 100 MG/KG/DAY MALES						
4004	3.17	2.03	1.00	0.63	0.07	0.24
4005	3.09	1.76	1.17	0.59	0.11	0.39
MEAN	3.13	1.90	1.09	0.61	0.09	0.32
S.D.	0.057	0.191	0.120	0.028	0.028	0.106
N	2	2	2	2	2	2

thous/uL = THOUSANDS/MICROLITER

Appendix 19

INDIVIDUAL IMMUNOPHENOTYPING VALUES (DAY 71)

ANIMAL	CD3+	CD3+CD4+	CD3+CD8+	CD3-CD20+	CD3-CD16+	CD14+
	%	%	%	%	%	%
GROUP: 0 MG/KG/DAY FEMALES						
1504	63.8	37.2	26.9	12.9	1.3	62.0
1505	58.8	24.1	30.0	10.5	0.6	45.0
MEAN	61.3	30.7	28.5	11.7	1.0	53.5
S.D.	3.54	9.26	2.19	1.70	0.49	12.02
N	2	2	2	2	2	2

Appendix 19

INDIVIDUAL IMMUNOPHENOTYPING VALUES (DAY 71)

ANIMAL	CD3+ ABS	CD3+CD4+ ABS	CD3+CD8+ ABS	CD3-CD20 + ABS	CD3-CD16 + ABS	CD14+ ABS
	thous/uL	thous/uL	thous/uL	thous/uL	thous/uL	thous/uL
GROUP: 0 MG/KG/DAY FEMALES						
1504	3.71	2.16	1.56	0.75	0.08	0.20
1505	3.28	1.34	1.67	0.59	0.03	0.15
MEAN	3.50	1.75	1.62	0.67	0.06	0.18
S.D.	0.304	0.580	0.078	0.113	0.035	0.035
N	2	2	2	2	2	2

thous/uL = THOUSANDS/MICROLITER

Appendix 19

INDIVIDUAL IMMUNOPHENOTYPING VALUES (DAY 71)

ANIMAL	CD3+	CD3+CD4+	CD3+CD8+	CD3-CD20+	CD3-CD16+	CD14+
	%	%	%	%	%	%
GROUP: 100 MG/KG/DAY FEMALES						
4504	46.3	25.8	18.4	16.4	2.3	78.3
4505	57.6	26.2	27.3	9.1	5.1	79.0
MEAN	52.0	26.0	22.9	12.8	3.7	78.7
S.D.	7.99	0.28	6.29	5.16	1.98	0.50
N	2	2	2	2	2	2

Appendix 19

INDIVIDUAL IMMUNOPHENOTYPING VALUES (DAY 71)

ANIMAL	CD3+ ABS	CD3+CD4+ ABS	CD3+CD8+ ABS	CD3-CD20 + ABS	CD3-CD16 + ABS	CD14+ ABS
	thous/uL	thous/uL	thous/uL	thous/uL	thous/uL	thous/uL
GROUP: 100 MG/KG/DAY FEMALES						
4504	2.13	1.18	0.84	0.75	0.11	0.38
4505	2.21	1.01	1.05	0.35	0.20	0.45
MEAN	2.17	1.10	0.95	0.55	0.16	0.42
S.D.	0.057	0.120	0.148	0.283	0.064	0.049
N	2	2	2	2	2	2

thous/uL = THOUSANDS/MICROLITER

PCHEv4.14
11/17/2022
R:11/17/2022

Appendix 20

Summary of Cytokines

Male

Endpoint	Effects	Statistics	Value
IFN-γ (pg/mL)	INTERACTIONS	Treatment*Time P Value	0.1693(NS)
	MAIN EFFECTS	Time F-test P Value	0.0983(NS)
		Treatment F-test P Value	0.0917(NS)

(S,NS): Statistically significant (S) or not statistically significant (NS) per protocol
NT - Not Tested

Appendix 20

Summary of Cytokines

			Day(s) Relative to Animal Start Date				
Sex: Male			Overall	1 (2 Hr EOI)	1 (24 Hr EOI)	29 (2 Hr EOI)	29 (24 Hr EOI)
IFN-γ (pg/mL)	Group 1<br><br>0 mg/kg/dose	Mean<br>SD<br>N	181.0958*<br>55.07891*<br>20*	206.7502*<br>35.06757*<br>5*	170.7554*<br>36.55395*<br>5*	196.8774*<br>96.78705*<br>5*	150.0000*<br>0.00000*<br>5*
	Group 2<br><br>3 mg/kg/dose	Mean<br>SD<br>N	196.8198*<br>150.25153*<br>12*	150.0000*<br>0.00000*<br>3*	150.0000*<br>0.00000*<br>3*	337.2790*<br>291.04598*<br>3*	150.0000*<br>0.00000*<br>3*
	Group 3<br><br>10 mg/kg/dose	Mean<br>SD<br>N	156.5850*<br>13.74855*<br>12*	158.7220*<br>15.10695*<br>3*	150.0000*<br>0.00000*<br>3*	153.4437*<br>5.96461*<br>3*	164.1743*<br>24.55067*<br>3*
	Group 4<br><br>100 mg/kg/dose	Mean<br>SD<br>N	152.7453*<br>12.27713*<br>20*	150.0000*<br>0.00000*<br>5*	150.0000*<br>0.00000*<br>5*	160.9810*<br>24.55426*<br>5*	150.0000*<br>0.00000*<br>5*

N - Number of measures used to calculate mean
SD - Standard Deviation

*Calculation includes one or more individual value(s) out of linear range

Appendix 20

Summary of Cytokines

Male

Endpoint	Effects	Statistics	Value
IL-6 (pg/mL)	INTERACTIONS	Treatment*Time P Value	0.9489(NS)
	MAIN EFFECTS	Time F-test P Value	0.7920(NS)
		Treatment F-test P Value	0.2647(NS)

(S,NS): Statistically significant (S) or not statistically significant (NS) per protocol
NT - Not Tested

Appendix 20

Summary of Cytokines

			Day(s) Relative to Animal Start Date				
Sex: Male			Overall	1 (2 Hr EOI)	1 (24 Hr EOI)	29 (2 Hr EOI)	29 (24 Hr EOI)
IL-6 (pg/mL)	Group 1<br>0 mg/kg/dose	Mean<br>SD<br>N	155.6836*<br>25.23531*<br>20*	172.5788*<br>50.48773*<br>5*	150.0000*<br>0.00000*<br>5*	150.1556*<br>0.34793*<br>5*	150.0000*<br>0.00000*<br>5*
	Group 2<br>3 mg/kg/dose	Mean<br>SD<br>N	150.0000*<br>0.00000*<br>12*	150.0000*<br>0.00000*<br>3*	150.0000*<br>0.00000*<br>3*	150.0000*<br>0.00000*<br>3*	150.0000*<br>0.00000*<br>3*
	Group 3<br>10 mg/kg/dose	Mean<br>SD<br>N	150.0000*<br>0.00000*<br>12*	150.0000*<br>0.00000*<br>3*	150.0000*<br>0.00000*<br>3*	150.0000*<br>0.00000*<br>3*	150.0000*<br>0.00000*<br>3*
	Group 4<br>100 mg/kg/dose	Mean<br>SD<br>N	150.0000*<br>0.00000*<br>20*	150.0000*<br>0.00000*<br>5*	150.0000*<br>0.00000*<br>5*	150.0000*<br>0.00000*<br>5*	150.0000*<br>0.00000*<br>5*

N - Number of measures used to calculate mean
SD - Standard Deviation

*Calculation includes one or more individual value(s) out of linear range

Appendix 20

Summary of Cytokines

Male

Endpoint	Effects	Statistics	Value
TNF-α (pg/mL)	INTERACTIONS	Treatment*Time P Value	0.0831(NS)
	MAIN EFFECTS	Time F-test P Value	0.0851(NS)
		Treatment F-test P Value	0.2386(NS)

(S,NS): Statistically significant (S) or not statistically significant (NS) per protocol
NT - Not Tested

Appendix 20

Summary of Cytokines

			Day(s) Relative to Animal Start Date				
Sex: Male			Overall	1 (2 Hr EOI)	1 (24 Hr EOI)	29 (2 Hr EOI)	29 (24 Hr EOI)
TNF-α (pg/mL)	Group 1 0 mg/kg/dose	Mean	471.2625*	786.7708*	452.4172*	495.8618*	150.0000*
		SD	392.53605*	365.65536*	357.33536*	464.61275*	0.00000*
		N	20*	5*	5*	5*	5*
	Group 2 3 mg/kg/dose	Mean	413.9018*	150.0000*	156.1763*	1183.6887*	165.7420*
		SD	691.51083*	0.00000*	10.69772*	1201.59866*	27.26594*
		N	12*	3*	3*	3*	3*
	Group 3 10 mg/kg/dose	Mean	348.8053*	332.9970*	150.0000*	348.2937*	563.9303*
		SD	293.68049*	302.27627*	0.00000*	343.45471*	368.72002*
		N	12*	3*	3*	3*	3*
	Group 4 100 mg/kg/dose	Mean	232.1129*	247.7230*	153.3380*	375.3326*	152.0578*
		SD	229.08817*	218.51527*	7.46399*	399.68545*	4.60138*
		N	20*	5*	5*	5*	5*

N - Number of measures used to calculate mean
SD - Standard Deviation

*Calculation includes one or more individual value(s) out of linear range

Appendix 20

Summary of Cytokines

Female

Endpoint	Effects	Statistics	Value
IFN-γ (pg/mL)	INTERACTIONS	Treatment*Time P Value	0.4031(NS)
	MAIN EFFECTS	Time F-test P Value	0.4056(NS)
		Treatment F-test P Value	0.3766(NS)

(S,NS): Statistically significant (S) or not statistically significant (NS) per protocol
NT - Not Tested

Appendix 20

Summary of Cytokines

			Day(s) Relative to Animal Start Date				
Sex: Female			Overall	1 (2 Hr EOI)	1 (24 Hr EOI)	29 (2 Hr EOI)	29 (24 Hr EOI)
IFN-γ (pg/mL)	Group 1<br><br>0 mg/kg/dose	Mean<br>SD<br>N	172.3347*<br>53.84308*<br>20*	157.9006*<br>17.66628*<br>5*	191.9596*<br>93.82452*<br>5*	189.4786*<br>54.06193*<br>5*	150.0000*<br>0.00000*<br>5*
	Group 2<br><br>3 mg/kg/dose	Mean<br>SD<br>N	150.0000*<br>0.00000*<br>12*	150.0000*<br>0.00000*<br>3*	150.0000*<br>0.00000*<br>3*	150.0000*<br>0.00000*<br>3*	150.0000*<br>0.00000*<br>3*
	Group 3<br><br>10 mg/kg/dose	Mean<br>SD<br>N	196.0943*<br>68.97170*<br>12*	265.8410*<br>103.20549*<br>3*	183.8593*<br>58.64609*<br>3*	161.8873*<br>20.58947*<br>3*	172.7893*<br>39.47228*<br>3*
	Group 4<br><br>100 mg/kg/dose	Mean<br>SD<br>N	162.9248*<br>26.42998*<br>20*	152.7298*<br>6.10402*<br>5*	155.2008*<br>11.62934*<br>5*	177.7110*<br>36.96341*<br>5*	166.0576*<br>35.90589*<br>5*

N - Number of measures used to calculate mean
SD - Standard Deviation

*Calculation includes one or more individual value(s) out of linear range

Appendix 20

Summary of Cytokines

			Day(s) Relative to Animal Start Date				
Sex: Female			Overall	1 (2 Hr EOI)	1 (24 Hr EOI)	29 (2 Hr EOI)	29 (24 Hr EOI)
IL-6 (pg/mL)	Group 1<br>0 mg/kg/dose	Mean<br>SD<br>N	150.0000*<br>0.00000*<br>20*	150.0000*<br>0.00000*<br>5*	150.0000*<br>0.00000*<br>5*	150.0000*<br>0.00000*<br>5*	150.0000*<br>0.00000*<br>5*
	Group 2<br>3 mg/kg/dose	Mean<br>SD<br>N	150.0000*<br>0.00000*<br>12*	150.0000*<br>0.00000*<br>3*	150.0000*<br>0.00000*<br>3*	150.0000*<br>0.00000*<br>3*	150.0000*<br>0.00000*<br>3*
	Group 3<br>10 mg/kg/dose	Mean<br>SD<br>N	150.0000*<br>0.00000*<br>12*	150.0000*<br>0.00000*<br>3*	150.0000*<br>0.00000*<br>3*	150.0000*<br>0.00000*<br>3*	150.0000*<br>0.00000*<br>3*
	Group 4<br>100 mg/kg/dose	Mean<br>SD<br>N	150.0000*<br>0.00000*<br>20*	150.0000*<br>0.00000*<br>5*	150.0000*<br>0.00000*<br>5*	150.0000*<br>0.00000*<br>5*	150.0000*<br>0.00000*<br>5*

N - Number of measures used to calculate mean
SD - Standard Deviation

*Calculation includes one or more individual value(s) out of linear range
No statistical analysis performed due to sample size or lack of variability

Appendix 20

Summary of Cytokines

Female

Endpoint	Effects	Statistics	Value
TNF-α (pg/mL)	INTERACTIONS	Treatment*Time P Value	0.1154(NS)
	MAIN EFFECTS	Time F-test P Value	0.1211(NS)
		Treatment F-test P Value	0.8494(NS)

(S,NS): Statistically significant (S) or not statistically significant (NS) per protocol
NT - Not Tested

Appendix 20
Table 1

Summary of Cytokines

			Day(s) Relative to Animal Start Date				
Sex: Female			Overall	1 (2 Hr EOI)	1 (24 Hr EOI)	29 (2 Hr EOI)	29 (24 Hr EOI)
TNF-α (pg/mL)	Group 1<br><br>0 mg/kg/dose	Mean<br>SD<br>N	359.3187*<br>349.46287*<br>20*	286.7664*<br>301.49584*<br>5*	408.6288*<br>456.01986*<br>5*	591.8796*<br>386.32085*<br>5*	150.0000*<br>0.00000*<br>5*
	Group 2<br><br>3 mg/kg/dose	Mean<br>SD<br>N	206.8855*<br>81.51566*<br>12*	189.4107<br>32.20762<br>3	216.5243<br>56.73470<br>3	171.9933*<br>19.42450*<br>3*	249.6137*<br>163.55670*<br>3*
	Group 3<br><br>10 mg/kg/dose	Mean<br>SD<br>N	555.0986*<br>468.17329*<br>12*	875.3677*<br>629.14998*<br>3*	530.5197*<br>481.14696*<br>3*	434.5887*<br>460.82684*<br>3*	379.9183*<br>378.48385*<br>3*
	Group 4<br><br>100 mg/kg/dose	Mean<br>SD<br>N	411.0977*<br>350.78084*<br>20*	369.9552*<br>301.57959*<br>5*	282.4722*<br>285.29737*<br>5*	605.3380*<br>485.16033*<br>5*	386.6254*<br>325.64099*<br>5*

N - Number of measures used to calculate mean
SD - Standard Deviation

*Calculation includes one or more individual value(s) out of linear range

Appendix 20

Individual Cytokine Values Testing

Group	Animal	Sex	Occasion	IFN-γ pg/mL	IL-6 pg/mL	TNF-α pg/mL
1	1001	M	Day 1: 2 Hr EOI	222.535	<150.000	1019.138
			Day 1: 24 Hr EOI	<150.000	<150.000	317.130
			Day 29: 2 Hr EOI	<150.000	<150.000	188.886
			Day 29: 24 Hr EOI	<150.000	<150.000	<150.000
	1002	M	Day 1: 2 Hr EOI	226.494	<150.000	1010.147
			Day 1: 24 Hr EOI	<150.000	<150.000	<150.000
			Day 29: 2 Hr EOI	<150.000	<150.000	334.906
			Day 29: 24 Hr EOI	<150.000	<150.000	<150.000
	1003	M	Day 1: 2 Hr EOI	237.784	262.894	809.974
			Day 1: 24 Hr EOI	169.378	<150.000	688.969
			Day 29: 2 Hr EOI	164.749	<150.000	520.287
			Day 29: 24 Hr EOI	<150.000	<150.000	<150.000
	1004	M	Day 1: 2 Hr EOI	<150.000	<150.000	<150.000
			Day 1: 24 Hr EOI	234.399	<150.000	955.987
			Day 29: 2 Hr EOI	<150.000	<150.000	<150.000
			Day 29: 24 Hr EOI	<150.000	<150.000	<150.000
	1005	M	Day 1: 2 Hr EOI	196.938	<150.000	944.595
			Day 1: 24 Hr EOI	<150.000	<150.000	<150.000
			Day 29: 2 Hr EOI	369.638	150.778	1285.230
			Day 29: 24 Hr EOI	<150.000	<150.000	<150.000
2	2001	M	Day 1: 2 Hr EOI	<150.000	<150.000	<150.000
			Day 1: 24 Hr EOI	<150.000	<150.000	<150.000
			Day 29: 2 Hr EOI	672.585	<150.000	2502.066
			Day 29: 24 Hr EOI	<150.000	<150.000	197.226
	2002	M	Day 1: 2 Hr EOI	<150.000	<150.000	<150.000
			Day 1: 24 Hr EOI	<150.000	<150.000	168.529
			Day 29: 2 Hr EOI	<150.000	<150.000	<150.000
			Day 29: 24 Hr EOI	<150.000	<150.000	<150.000
	2003	M	Day 1: 2 Hr EOI	<150.000	<150.000	<150.000
			Day 1: 24 Hr EOI	<150.000	<150.000	<150.000
			Day 29: 2 Hr EOI	189.252	<150.000	899.000
			Day 29: 24 Hr EOI	<150.000	<150.000	<150.000

Appendix 20

Individual Cytokine Values Testing

Group	Animal	Sex	Occasion	IFN-γ pg/mL	IL-6 pg/mL	TNF-α pg/mL
3	3001	M	Day 1: 2 Hr EOI	176.166	<150.000	681.896
			Day 1: 24 Hr EOI	<150.000	<150.000	<150.000
			Day 29: 2 Hr EOI	160.331	<150.000	744.881
			Day 29: 24 Hr EOI	<150.000	<150.000	684.578
	3002	M	Day 1: 2 Hr EOI	<150.000	<150.000	<150.000
			Day 1: 24 Hr EOI	<150.000	<150.000	<150.000
			Day 29: 2 Hr EOI	<150.000	<150.000	<150.000
			Day 29: 24 Hr EOI	<150.000	<150.000	<150.000
	3003	M	Day 1: 2 Hr EOI	<150.000	<150.000	167.095
			Day 1: 24 Hr EOI	<150.000	<150.000	<150.000
			Day 29: 2 Hr EOI	<150.000	<150.000	<150.000
			Day 29: 24 Hr EOI	192.523	<150.000	857.213
4	4001	M	Day 1: 2 Hr EOI	<150.000	<150.000	<150.000
			Day 1: 24 Hr EOI	<150.000	<150.000	<150.000
			Day 29: 2 Hr EOI	<150.000	<150.000	<150.000
			Day 29: 24 Hr EOI	<150.000	<150.000	<150.000
	4002	M	Day 1: 2 Hr EOI	<150.000	<150.000	<150.000
			Day 1: 24 Hr EOI	<150.000	<150.000	<150.000
			Day 29: 2 Hr EOI	<150.000	<150.000	169.836
			Day 29: 24 Hr EOI	<150.000	<150.000	160.289
	4003	M	Day 1: 2 Hr EOI	<150.000	<150.000	<150.000
			Day 1: 24 Hr EOI	<150.000	<150.000	<150.000
			Day 29: 2 Hr EOI	<150.000	<150.000	329.287
			Day 29: 24 Hr EOI	<150.000	<150.000	<150.000
	4004	M	Day 1: 2 Hr EOI	<150.000	<150.000	<150.000
			Day 1: 24 Hr EOI	<150.000	<150.000	166.690
			Day 29: 2 Hr EOI	204.905	<150.000	1077.540
			Day 29: 24 Hr EOI	<150.000	<150.000	<150.000
	4005	M	Day 1: 2 Hr EOI	<150.000	<150.000	638.615
			Day 1: 24 Hr EOI	<150.000	<150.000	<150.000
			Day 29: 2 Hr EOI	<150.000	<150.000	<150.000
			Day 29: 24 Hr EOI	<150.000	<150.000	<150.000

Appendix 20

Individual Cytokine Values Testing

Group	Animal	Sex	Occasion	IFN-γ pg/mL	IL-6 pg/mL	TNF-α pg/mL
1	1501	F	Day 1: 2 Hr EOI	189.503	<150.000	826.065
			Day 1: 24 Hr EOI	359.798	<150.000	1221.436
			Day 29: 2 Hr EOI	249.582	<150.000	901.477
			Day 29: 24 Hr EOI	<150.000	<150.000	<150.000
	1502	F	Day 1: 2 Hr EOI	<150.000	<150.000	<150.000
			Day 1: 24 Hr EOI	<150.000	<150.000	220.742
			Day 29: 2 Hr EOI	<150.000	<150.000	429.925
			Day 29: 24 Hr EOI	<150.000	<150.000	<150.000
	1503	F	Day 1: 2 Hr EOI	<150.000	<150.000	<150.000
			Day 1: 24 Hr EOI	<150.000	<150.000	<150.000
			Day 29: 2 Hr EOI	<150.000	<150.000	395.232
			Day 29: 24 Hr EOI	<150.000	<150.000	<150.000
	1504	F	Day 1: 2 Hr EOI	<150.000	<150.000	<150.000
			Day 1: 24 Hr EOI	<150.000	<150.000	256.190
			Day 29: 2 Hr EOI	<150.000	<150.000	<150.000
			Day 29: 24 Hr EOI	<150.000	<150.000	<150.000
	1505	F	Day 1: 2 Hr EOI	<150.000	<150.000	157.767
			Day 1: 24 Hr EOI	<150.000	<150.000	194.776
			Day 29: 2 Hr EOI	247.811	<150.000	1082.764
			Day 29: 24 Hr EOI	<150.000	<150.000	<150.000
2	2501	F	Day 1: 2 Hr EOI	<150.000	<150.000	210.536
			Day 1: 24 Hr EOI	<150.000	<150.000	157.767
			Day 29: 2 Hr EOI	<150.000	<150.000	<150.000
			Day 29: 24 Hr EOI	<150.000	<150.000	<150.000
	2502	F	Day 1: 2 Hr EOI	<150.000	<150.000	152.341
			Day 1: 24 Hr EOI	<150.000	<150.000	220.813
			Day 29: 2 Hr EOI	<150.000	<150.000	179.178
			Day 29: 24 Hr EOI	<150.000	<150.000	160.465
	2503	F	Day 1: 2 Hr EOI	<150.000	<150.000	205.355
			Day 1: 24 Hr EOI	<150.000	<150.000	270.993
			Day 29: 2 Hr EOI	<150.000	<150.000	186.802
			Day 29: 24 Hr EOI	<150.000	<150.000	438.376

Appendix 20

Individual Cytokine Values Testing

Group	Animal	Sex	Occasion	IFN-γ pg/mL	IL-6 pg/mL	TNF-α pg/mL
3	3501	F	Day 1: 2 Hr EOI	<150.000	<150.000	<150.000
			Day 1: 24 Hr EOI	<150.000	<150.000	<150.000
			Day 29: 2 Hr EOI	<150.000	<150.000	<150.000
			Day 29: 24 Hr EOI	<150.000	<150.000	173.003
	3502	F	Day 1: 2 Hr EOI	299.533	<150.000	1203.252
			Day 1: 24 Hr EOI	<150.000	<150.000	370.200
			Day 29: 2 Hr EOI	185.662	<150.000	966.265
			Day 29: 24 Hr EOI	<150.000	<150.000	<150.000
	3503	F	Day 1: 2 Hr EOI	347.990	<150.000	1272.851
			Day 1: 24 Hr EOI	251.578	<150.000	1071.359
			Day 29: 2 Hr EOI	<150.000	<150.000	187.501
			Day 29: 24 Hr EOI	218.368	<150.000	816.752
4	4501	F	Day 1: 2 Hr EOI	<150.000	<150.000	<150.000
			Day 1: 24 Hr EOI	<150.000	<150.000	<150.000
			Day 29: 2 Hr EOI	<150.000	<150.000	<150.000
			Day 29: 24 Hr EOI	<150.000	<150.000	<150.000
	4502	F	Day 1: 2 Hr EOI	<150.000	<150.000	678.107
			Day 1: 24 Hr EOI	<150.000	<150.000	169.763
			Day 29: 2 Hr EOI	214.744	<150.000	1182.399
			Day 29: 24 Hr EOI	<150.000	<150.000	695.567
	4503	F	Day 1: 2 Hr EOI	<150.000	<150.000	<150.000
			Day 1: 24 Hr EOI	<150.000	<150.000	<150.000
			Day 29: 2 Hr EOI	221.466	<150.000	1077.605
			Day 29: 24 Hr EOI	<150.000	<150.000	<150.000
	4504	F	Day 1: 2 Hr EOI	163.649	<150.000	721.669
			Day 1: 24 Hr EOI	176.004	<150.000	792.598
			Day 29: 2 Hr EOI	152.345	<150.000	337.855
			Day 29: 24 Hr EOI	230.288	<150.000	787.560
	4505	F	Day 1: 2 Hr EOI	<150.000	<150.000	<150.000
			Day 1: 24 Hr EOI	<150.000	<150.000	<150.000
			Day 29: 2 Hr EOI	<150.000	<150.000	278.831
			Day 29: 24 Hr EOI	<150.000	<150.000	<150.000

Hr = hours; EOI = end of infusion.

Appendix 20

Back-calculated Values of Standards for Analyte IFN-γ Testing

Assay Date	Watson Assay ID	Theoretical Concentration (pg/mL)								
		Standard	Standard	Standard	Standard	Standard	Standard	Standard	Standard	Standard
		37.500	75.000	150.000	300.000	600.000	1200.000	2400.000	4800.000	6000.000
		Observed Concentration (pg/mL)								
16-Nov-2022	1	34.132	81.797	147.399	288.630	669.971	1214.708	2367.939	4883.661	5976.307
		40.285	74.824	146.289	305.064	561.441	1203.548	2407.350	4706.093	6048.297
16-Nov-2022	2	37.826	84.033	146.977	289.843	616.953	1228.970	2464.939	4857.483	6082.927
		36.778	69.305	155.374	308.160	588.069	1109.578	2577.206	4510.814	6046.060
16-Nov-2022	3	37.876	67.491	142.391	311.349	612.843	*623.373	2286.536	4546.458	5958.943
		38.935	78.054	153.981	307.140	627.585	1228.073	2268.350	5313.978	6115.513
16-Nov-2022	4	35.426	73.094	151.822	316.390	609.706	1281.591	2402.860	4783.904	6236.842
		39.465	78.227	152.866	278.688	597.250	1126.522	2367.536	4582.396	6183.524
17-Nov-2022	5	37.446	74.589	143.885	307.900	615.380	1196.105	2390.506	4907.706	5755.866
		37.446	76.685	150.531	304.901	605.010	1104.384	2557.264	4858.163	6056.138
17-Nov-2022	7	37.446	74.589	143.885	307.900	615.380	1196.105	2390.506	4907.706	5755.866
		37.446	76.685	150.531	304.901	605.010	1104.384	2557.264	4858.163	6056.138
Mean Concentration:		37.542	75.781	148.828	302.572	610.383	1181.270	2419.855	4809.710	6022.702
SD:		1.654	4.658	4.293	10.958	25.352	60.452	101.302	214.826	146.479
CV(%):		4	6	3	4	4	5	4	4	2
%RE		0	1	-1	1	2	-2	1	0	0
n		12	12	12	12	12	11	12	12	12

Note: Standard concentration 37.500 pg/mL is an accessory standard.

*Masked for CV% out of acceptance.

Appendix 20

Back-calculated Values of Standards for Analyte IL-6 Testing

Assay Date	Watson Assay ID	Theoretical Concentration (pg/mL)								
		Standard	Standard	Standard	Standard	Standard	Standard	Standard	Standard	Standard
		37.500	75.000	150.000	300.000	600.000	1200.000	2400.000	4800.000	6000.000
		Observed Concentration (pg/mL)								
16-Nov-2022	1	37.598	76.986	150.519	316.625	586.353	1176.455	2399.107	5203.581	5640.517
		37.598	73.019	141.814	320.363	587.200	1197.977	2392.424	4975.138	5812.543
16-Nov-2022	2	36.823	78.452	152.026	299.656	601.052	1237.636	2418.744	5093.576	5378.290
		37.761	74.328	146.056	302.302	582.004	1161.149	2480.756	5115.064	5662.114
16-Nov-2022	3	37.384	76.964	141.418	297.241	581.085	*594.962	2290.538	5324.399	5869.245
		37.520	74.237	155.765	309.971	623.149	1205.871	2385.882	5193.170	5459.084
16-Nov-2022	4	37.522	73.507	153.783	295.407	604.381	1306.418	2389.610	4899.523	5863.514
		37.522	76.115	149.041	293.647	631.018	1085.080	2418.962	4901.144	5934.645
17-Nov-2022	5	37.742	70.084	152.582	327.281	587.701	1159.681	2488.756	5336.024	5414.059
		38.208	75.930	149.794	302.778	604.879	1073.224	2626.518	5281.939	5672.266
Mean Concentration:		37.568	74.962	149.280	306.527	598.882	1178.166	2429.130	5132.356	5670.628
SD:		0.346	2.430	4.837	11.503	17.361	71.987	88.369	164.553	200.976
CV(%):		1	3	3	4	3	6	4	3	4
%RE		0	0	0	2	0	-2	1	7	-5
n		10	10	10	10	10	9	10	10	10

Note: Standard concentration 37.500 pg/mL is an accessory standard.

*Masked for CV% out of acceptance.

Appendix 20

Back-calculated Values of Standards for Analyte TNF-α Testing

Assay Date	Watson Assay ID	Theoretical Concentration (pg/mL)									
		Standard	Standard	Standard	Standard	Standard	Standard	Standard	Standard	Standard	Standard
		24.000	48.000	75.000	96.000	192.000	384.000	768.000	1536.000	3072.000	3840.000
		Observed Concentration (pg/mL)									
16-Nov-2022	1	19.711	55.653	72.551	102.504	210.034	407.296	768.397	1440.593	3149.619	4102.635
		19.711	61.340	69.767	94.443	200.112	388.834	649.183	1434.573	3085.051	4340.054
16-Nov-2022	2	32.880	56.679	84.264	120.569	216.245	410.639	742.638	1440.956	3009.783	4324.187
		19.900	32.880	62.347	94.862	202.425	389.939	671.008	1509.429	2993.432	4162.641
16-Nov-2022	3	26.807	49.914	83.548	95.283	210.097	398.896	*394.114	1332.768	3012.112	4481.146
		12.310	53.078	83.548	106.822	181.113	384.525	709.965	1346.931	3401.803	4324.651
16-Nov-2022	4	17.160	46.836	80.145	92.838	209.800	398.875	769.894	1470.090	3008.972	4314.500
		25.031	46.836	80.145	105.272	204.243	355.756	714.974	1432.886	2890.150	4436.964
17-Nov-2022	5	28.718	41.604	71.251	98.786	205.810	412.095	736.225	1415.008	3089.525	3999.548
		21.890	47.775	76.891	96.100	203.455	401.675	685.255	1585.367	3003.406	4168.701
17-Nov-2022	6	28.718	41.604	71.251	98.786	205.810	412.095	736.225	1415.008	3089.525	3999.548
		21.890	47.775	76.891	96.100	203.455	401.675	685.255	1585.367	3003.406	4168.701
Mean Concentration:		22.894	48.498	76.050	100.197	204.383	396.858	715.365	1450.748	3061.399	4235.273
SD:		5.749	7.696	6.745	7.789	8.549	15.873	39.484	78.679	125.884	158.730
CV(%):		25	16	9	8	4	4	6	5	4	4
%RE		-5	1	1	4	6	3	-7	-6	0	10
n		12	12	12	12	12	12	11	12	12	12

Note: Standard concentrations 24.000 and 48.000 pg/mL are accessory standards.

*Masked for CV% out of acceptance.

Appendix 20

Summary of Calibration Parameters for Analyte IFN-γ Testing

Assay Date	Watson Assay ID	Response at Lowest Working Concentration (A)	Response at Infinite Concentration (D)	Slope (B)	Mid-Range Concentration ED50 (C)	Asymmetry Factor (G)	Coefficient of Determination (R^2)	LLOQ (pg/mL)	ULOQ (pg/mL)
16-Nov-2022	1	17.470	58476.515	1.080	13948.096	0.290	0.997	75	6000
16-Nov-2022	2	17.432	55642.746	1.046	30866.122	0.689	0.997	75	6000
16-Nov-2022	3	7.404	301295.509	1.005	94773.873	0.307	0.996	75	6000
16-Nov-2022	4	7.037	214451.336	0.977	163820.153	0.639	0.998	75	6000
17-Nov-2022	5	10.649	37328.091	1.043	29408.290	0.934	0.999	75	6000
17-Nov-2022	7	10.649	37328.091	1.043	29408.290	0.934	0.999	75	6000

Appendix 20

Summary of Calibration Parameters for Analyte IL-6 Testing

Assay Date	Watson Assay ID	Response at Lowest Working Concentration (A)	Response at Infinite Concentration (D)	Slope (B)	Mid-Range Concentration ED50 (C)	Asymmetry Factor (G)	Coefficient of Determination (R^2)	LLOQ (pg/mL)	ULOQ (pg/mL)
16-Nov-2022	1	-16.498	28280.007	1.063	9668.232	4.083	0.999	75	6000
16-Nov-2022	2	9.209	27914.322	1.070	81894.012	45.700	0.999	75	6000
16-Nov-2022	3	26.124	38000.194	1.152	2364.950	0.761	0.999	75	6000
16-Nov-2022	4	-34.369	31362.548	1.044	7583.684	2.747	0.999	75	6000
17-Nov-2022	5	61.460	104208.699	1.262	782.380	0.101	0.997	75	6000

Appendix 20

Summary of Calibration Parameters for Analyte TNF-α Testing

Assay Date	Watson Assay ID	Response at Lowest Working Concentration (A)	Response at Infinite Concentration (D)	Slope (B)	Mid-Range Concentration ED50 (C)	Asymmetry Factor (G)	Coefficient of Determination (R^2)	LLOQ (pg/mL)	ULOQ (pg/mL)
16-Nov-2022	1	8.224	5184510.685	1.109	8561695.458	0.957	0.989	75	3840
16-Nov-2022	2	10.484	1573425.788	1.165	1786237.942	0.941	0.984	75	3840
16-Nov-2022	3	8.595	2424634.539	1.137	3610176.533	0.957	0.982	75	3840
16-Nov-2022	4	10.165	1392927.782	1.152	2060856.809	0.938	0.991	75	3840
17-Nov-2022	5	10.346	1168632.091	1.178	1269976.634	0.924	0.993	75	3840
17-Nov-2022	6	10.346	1168632.091	1.178	1269976.634	0.924	0.993	75	3840

Appendix 20

Summary Data for Quality Control Samples for Analyte IFN-γ Testing

Assay Date	Watson Assay ID	Theoretical Concentration (pg/mL)		
		QC1	QC2	QC3
		(200.000 pg/mL)	(1000.000 pg/mL)	(3200.000 pg/mL)
		Observed Concentration (pg/mL)		
16-Nov-2022	1	187.009	1027.630	3000.918
		203.339	1037.260	2952.360
		189.192	879.056	2930.815
		188.100	914.063	2989.038
16-Nov-2022	2	187.329	982.264	2838.688
		179.484	968.120	2954.711
		161.783	893.631	2818.084
		181.446	974.721	2827.893
16-Nov-2022	3	203.479	981.823	3330.484
		198.215	972.322	3110.425
		180.314	903.725	2867.233
		176.101	909.000	3019.215
16-Nov-2022	4	191.615	1097.805	3123.699
		203.175	1019.517	3268.903
		182.170	954.067	3047.486
		180.597	978.250	3110.989
17-Nov-2022	5	212.479	982.169	3160.255
		208.942	1077.148	3146.402
		204.898	989.584	3189.587
		188.188	975.743	3047.009
17-Nov-2022	7	212.479	982.169	3160.255
		208.942	1077.148	3146.402
		204.898	989.584	3189.587
		188.188	975.743	3047.009
	Mean	192.598	980.939	3053.227
	SD	13.281	56.660	138.642
	CV(%)	7	6	5
	%RE	-4	-2	-5
	n	24	24	24

Appendix 20

Summary Data for Quality Control Samples for Analyte IL-6 Testing

Assay Date	Watson Assay ID	Theoretical Concentration (pg/mL)		
		**QC1**	**QC2**	**QC3**
		(200.000 pg/mL)	(1000.000 pg/mL)	(3200.000 pg/mL)
		**Observed Concentration (pg/mL)**		
16-Nov-2022	1	209.037	1002.689	3146.895
		180.073	963.456	3183.834
		175.389	892.355	3026.812
		150.628	918.796	3241.070
16-Nov-2022	2	186.632	893.149	2992.244
		194.272	854.081	3214.135
		157.451	897.325	3118.212
		160.804	916.016	3276.216
16-Nov-2022	3	220.005	1050.322	3330.257
		205.762	1033.250	3633.290
		181.228	927.003	3207.247
		168.640	987.137	3516.989
16-Nov-2022	4	199.639	1106.362	3176.853
		215.034	1001.691	3321.668
		177.869	998.023	3224.867
		169.901	1039.440	3325.757
17-Nov-2022	5	195.601	873.972	3538.436
		209.874	954.589	3499.706
		190.342	945.170	3714.170
		178.423	952.160	3662.324
	Mean	186.330	960.349	3317.549
	SD	19.748	66.346	209.480
	CV(%)	11	7	6
	%RE	-7	-4	4
	n	20	20	20

Appendix 20

Summary Data for Quality Control Samples for Analyte TNF-α Testing

Assay Date	Watson Assay ID	Theoretical Concentration (pg/mL)		
		**QC1**	**QC2**	**QC3**
		(128.000 pg/mL)	(640.000 pg/mL)	(2048.000 pg/mL)
		**Observed Concentration (pg/mL)**		
16-Nov-2022	1	147.043	614.069	2015.211
		121.057	596.430	2100.130
		126.299	576.518	2065.436
		126.299	596.430	2065.436
16-Nov-2022	2	140.490	601.966	1928.133
		130.592	628.963	2012.539
		125.597	586.450	1926.535
		125.597	619.344	1870.455
16-Nov-2022	3	143.257	705.548	2044.977
		151.474	602.846	2062.153
		129.414	593.806	1877.885
		126.621	609.613	1956.872
16-Nov-2022	4	144.306	678.004	1960.017
		153.072	678.004	2066.234
		135.458	598.387	1918.477
		138.416	638.384	2054.474
17-Nov-2022	5	162.617	629.747	2113.187
		135.210	694.743	1842.248
		160.162	715.537	2114.744
		155.230	660.474	2060.140
17-Nov-2022	6	162.617	629.747	2113.187
		135.210	694.743	1842.248
		160.162	715.537	2114.744
		155.230	660.474	2060.140
	Mean	141.310	638.574	2007.733
	SD	13.574	44.293	91.391
	CV(%)	10	7	5
	%RE	10	0	-2
	n	24	24	24

Appendix 20

Summary of Cytokine Analyses Testing

Plate No.	Watson Run ID	Assay Date	Analyst	IFN-γ	IL-6	TNF-α	Comments
CYT01	1	16-Nov-2022	1				N/A
CYT02	2	16-Nov-2022	1				N/A
CYT03	3	16-Nov-2022	2				N/A
CYT04	4	16-Nov-2022	2				N/A
CYT05	5, 6, 7	17-Nov-2022	1				N/A

N/A = not applicable.
Blank = pass.
Shaded = specified analyte was not analyzed on this plate.
FAIL = all samples failed acceptance criteria for analyte specified on this plate.

Appendix 21

TABLES EXPLANATION PAGE

All Day(s) referenced throughout the outputs generated are Study Days beginning with Day 1, the first day of dosing

Abbreviations consistent throughout the Summary and Individual Tables

Note: All of the abbreviations listed on these pages may not be applicable to this report

Abbreviation	Description
mg/kg/dose	milligrams/kilograms/dose
mg/kg	milligrams/kilograms
ppm	parts per million
RC	result comment
% Diff	% Difference from Group 1
1F, 2F, 3F, 4F	Group 1 Female, Group 2 Female, Group 3 Female, Group 4 Female
1M, 2M, 3M, 4M	Group 1 Male, Group 2 Male, Group 3 Male, Group 4 Male
g	grams
kg	kilograms
mg	milligrams
N	Number of values included in analysis
M, F	Male, Female
<, >	Out of range
--	Not scheduled to be performed/dead

Group 1 - 0 mg/kg/dose
Group 2 - 3 mg/kg/dose
Group 3 - 10 mg/kg/dose
Group 4 - 100 mg/kg/dose

CAUSE OF DEATH

Abbreviation	Description	Abbreviation	Description
ACCD	Accidental death	TERM	Terminal euthanasia
FD	Found dead	TGL	Trackable gross lesion
M	Mass	UNSC	Unscheduled euthanasia
MSAC	Moribund sacrifice		

Appendix 21

MACROSCOPIC AND MICROSCOPIC FINDINGS

Abbreviation	Description	Abbreviation	Description
ACCD	Accidental death	MPF	Major pathological finding
C	Clinical observation	MSAC	Moribund sacrifice
E	Excluded	ONP	Organ not present
FD	Found dead	OPOP	Only one of the paired organs present
G	Gross pathology	OUM	Organ unidentifiable macroscopically
H	Histo pathology	TERM	Terminal euthanasia
LIBW	Lung infused before weighing	TGL	Trackable gross lesion
M	Mass	UNSC	Unscheduled euthanasia
mL	milliliters		

ORGAN WEIGHTS

Abbreviation	Description	Abbreviation	Description
CG	Coagulating gland	OUM	Organ unidentifiable macroscopically
Ep	Epididymis	PI	Macroscopic pathology - Included in mean
LIBW	Lung infused before weighing	Sem Ves	Seminal vesicle
Liver wt igb	Liver weight intact gallbladder	X	Excluded from calculation of the mean
MPE	Macroscopic pathology - exclude from mean	OP	Only one pair organ present - excluded from calculations
MPI	Macroscopic pathology - include in mean	OPMP	Only one of the paired organs present - macroscopic pathology
ONP	Organ not present	OPOP	Only one of the paired organs present
PARATHY	Parathyroid	TBR	Terminal Bodyweight Ratio
BR	Brain Ratio	WT	Weight
BW	Body Weight	GLD	Gland
%Diff G1	Percent Difference from Group 1	NT	Not taken
FC	Flag comment	%br	% Organ Weight/Brain Weight
%bw	% Organ Weight/Body Weight		

Appendix 21

Summary of Macroscopic Pathology: Terminal Euthanasia - Day 31

Removal Reason(s): TERMINAL EUTHANASIA<br>Summary: Incidence	Male				Female			
	0 mg/kg /dose Group 1	3 mg/kg /dose Group 2	10 mg/kg /dose Group 3	100 mg/kg /dose Group 4	0 mg/kg /dose Group 1	3 mg/kg /dose Group 2	10 mg/kg /dose Group 3	100 mg/kg /dose Group 4
Number of Animals:	3	3	3	3	3	3	3	3
**ARTERY, AORTA**								
Submitted	3	3	3	3	3	3	3	3
No Visible Lesions	3	3	3	3	3	3	3	3
**BONE MARROW, STERNUM**								
Submitted	3	3	3	3	3	3	3	3
No Visible Lesions	3	3	3	3	3	3	3	3
**BONE, FEMUR**								
Submitted	3	3	3	3	3	3	3	3
No Visible Lesions	3	3	3	3	3	3	3	3
**BONE, SKULL**								
Submitted	.	.	.	.	0	0	1	0
Abnormal appearance; deformity	.	.	.	.	.	.	1	.
**BONE, STERNUM**								
Submitted	3	3	3	3	3	3	3	3
No Visible Lesions	3	3	3	3	3	3	3	3
**BRAIN**								
Submitted	3	3	3	3	3	3	3	3
No Visible Lesions	3	3	3	3	3	3	2	3
Discoloration, dark; tan, cerebrum	0	0	0	0	0	0	1	0
**CERVIX**								
Submitted	.	.	.	.	3	3	3	3
No Visible Lesions	.	.	.	.	3	3	3	3
**EPIDIDYMIS**								
Submitted	3	3	3	3	.	.	.	.
No Visible Lesions	3	3	3	3	.	.	.	.
**ESOPHAGUS**								
Submitted	3	3	3	3	3	3	3	3
No Visible Lesions	3	3	3	3	3	3	3	3
**EYE**								
Submitted	3	3	3	3	3	3	3	3
No Visible Lesions	3	3	3	3	3	3	3	3
**GALLBLADDER**								
Submitted	3	3	3	3	3	3	3	3
No Visible Lesions	3	3	3	3	3	3	3	3

Appendix 21

Summary of Macroscopic Pathology: Terminal Euthanasia - Day 31

Removal Reason(s): TERMINAL EUTHANASIA	Male				Female			
Summary: Incidence	0 mg/kg /dose Group 1	3 mg/kg /dose Group 2	10 mg/kg /dose Group 3	100 mg/kg /dose Group 4	0 mg/kg /dose Group 1	3 mg/kg /dose Group 2	10 mg/kg /dose Group 3	100 mg/kg /dose Group 4
Number of Animals:	3	3	3	3	3	3	3	3
**GALT**								
Submitted	3	3	3	3	3	3	3	3
No Visible Lesions	3	3	3	3	3	3	3	3
**GLAND, ADRENAL**								
Submitted	3	3	3	3	3	3	3	3
No Visible Lesions	3	3	3	3	3	3	3	3
**GLAND, LACRIMAL**								
Submitted	3	3	3	3	3	3	3	3
No Visible Lesions	3	3	3	3	3	3	3	3
**GLAND, MAMMARY**								
Submitted	3	3	3	3	3	3	3	3
No Visible Lesions	3	3	3	3	3	3	3	3
**GLAND, PARATHYROID**								
Submitted	3	3	3	3	3	3	3	3
No Visible Lesions	3	3	3	3	3	3	3	3
**GLAND, PITUITARY**								
Submitted	3	3	3	3	3	3	3	3
No Visible Lesions	3	3	3	3	3	3	3	3
**GLAND, PROSTATE**								
Submitted	3	3	3	3	.	.	.	.
No Visible Lesions	3	3	3	3	.	.	.	.
**GLAND, SALIVARY, PAROTID**								
Submitted	3	3	3	3	3	3	3	3
No Visible Lesions	3	3	3	3	3	3	3	3
**GLAND, SALIVARY, SUBLINGUAL**								
Submitted	3	3	3	3	3	3	3	3
No Visible Lesions	3	3	3	3	3	3	3	3
**GLAND, SEMINAL VESICLE**								
Submitted	3	3	3	3	.	.	.	.
No Visible Lesions	3	3	3	3	.	.	.	.
**GLAND, THYROID**								
Submitted	3	3	3	3	3	3	3	3
No Visible Lesions	3	3	3	3	3	3	3	3

Appendix 21

Summary of Macroscopic Pathology: Terminal Euthanasia - Day 31

Removal Reason(s): TERMINAL EUTHANASIA<br>Summary: Incidence	Male				Female			
	0 mg/kg /dose Group 1	3 mg/kg /dose Group 2	10 mg/kg /dose Group 3	100 mg/kg /dose Group 4	0 mg/kg /dose Group 1	3 mg/kg /dose Group 2	10 mg/kg /dose Group 3	100 mg/kg /dose Group 4
Number of Animals:	3	3	3	3	3	3	3	3
**HEART**								
Submitted	3	3	3	3	3	3	3	3
No Visible Lesions	3	3	3	3	3	3	3	3
**JOINT, FEMOROTIBIAL**								
Submitted	3	3	3	3	3	3	3	3
No Visible Lesions	3	3	3	3	3	3	3	3
**KIDNEY**								
Submitted	3	3	3	3	3	3	3	3
No Visible Lesions	3	3	3	3	3	3	3	3
**LARGE INTESTINE, CECUM**								
Submitted	3	3	3	3	3	3	3	3
No Visible Lesions	3	3	3	3	3	3	3	3
**LARGE INTESTINE, COLON**								
Submitted	3	3	3	3	3	3	3	3
No Visible Lesions	3	3	3	3	3	3	3	3
**LARGE INTESTINE, RECTUM**								
Submitted	3	3	3	3	3	3	3	3
No Visible Lesions	3	3	3	3	3	3	3	3
**LIVER**								
Submitted	3	3	3	3	3	3	3	3
No Visible Lesions	3	3	3	3	3	3	3	3
**LUNG**								
Submitted	3	3	3	3	3	3	3	3
No Visible Lesions	3	3	3	3	2	3	3	3
Adhesion; thoracic wall	0	0	0	0	1	0	0	0
**LYMPH NODE, AXILLARY**								
Submitted	3	3	3	3	3	3	3	3
No Visible Lesions	3	3	3	3	3	3	3	3
**LYMPH NODE, MANDIBULAR**								
Submitted	3	3	3	3	3	3	3	3
No Visible Lesions	3	3	3	3	3	3	3	3
**LYMPH NODE, MESENTERIC**								
Submitted	3	3	3	3	3	3	3	3
No Visible Lesions	3	3	3	3	3	3	3	3

Appendix 21

Summary of Macroscopic Pathology: Terminal Euthanasia - Day 31

Removal Reason(s): TERMINAL EUTHANASIA Summary: Incidence	Male				Female			
	0 mg/kg /dose Group 1	3 mg/kg /dose Group 2	10 mg/kg /dose Group 3	100 mg/kg /dose Group 4	0 mg/kg /dose Group 1	3 mg/kg /dose Group 2	10 mg/kg /dose Group 3	100 mg/kg /dose Group 4
Number of Animals:	3	3	3	3	3	3	3	3
**MUSCLE, QUADRICEPS**								
Submitted	3	3	3	3	3	3	3	3
No Visible Lesions	3	3	3	3	3	3	3	3
**NERVE, OPTIC**								
Submitted	3	3	3	3	3	3	3	3
No Visible Lesions	3	3	3	3	3	3	3	3
**NERVE, SCIATIC**								
Submitted	3	3	3	3	3	3	3	3
No Visible Lesions	3	3	3	3	3	3	3	3
**NERVE, TIBIAL**								
Submitted	3	3	3	3	3	3	3	3
No Visible Lesions	3	3	3	3	3	3	3	3
**OVARY**								
Submitted	.	.	.	.	3	3	3	3
No Visible Lesions	.	.	.	.	1	3	3	2
Small	.	.	.	.	2	0	0	0
Cyst, clear; fluid	.	.	.	.	0	0	0	1
**OVIDUCT**								
Submitted	.	.	.	.	3	3	3	3
No Visible Lesions	.	.	.	.	3	2	3	3
Cyst, clear; fluid	.	.	.	.	0	1	0	0
**PANCREAS**								
Submitted	3	3	3	3	3	3	3	3
No Visible Lesions	3	3	3	3	3	3	3	3
**SITE, INFUSION, CEPHALIC, LEFT**								
Submitted	3	3	3	3	3	3	3	3
No Visible Lesions	3	3	3	3	3	3	3	3
**SITE, INFUSION, CEPHALIC, RIGHT**								
Submitted	3	3	3	3	3	3	3	3
No Visible Lesions	3	3	3	3	3	3	3	3
**SKIN**								
Submitted	3	3	3	3	3	3	3	3
No Visible Lesions	3	3	3	3	3	3	3	3

Appendix 21

Summary of Macroscopic Pathology: Terminal Euthanasia - Day 31

Removal Reason(s): TERMINAL EUTHANASIA<br>Summary: Incidence	Male				Female			
	0 mg/kg /dose Group 1	3 mg/kg /dose Group 2	10 mg/kg /dose Group 3	100 mg/kg /dose Group 4	0 mg/kg /dose Group 1	3 mg/kg /dose Group 2	10 mg/kg /dose Group 3	100 mg/kg /dose Group 4
Number of Animals:	3	3	3	3	3	3	3	3
**SMALL INTESTINE, DUODENUM**								
Submitted	3	3	3	3	3	3	3	3
No Visible Lesions	3	3	3	3	3	3	3	3
**SMALL INTESTINE, ILEUM**								
Submitted	3	3	3	3	3	3	3	3
No Visible Lesions	3	3	3	3	3	3	3	3
**SMALL INTESTINE, JEJUNUM**								
Submitted	3	3	3	3	3	3	3	3
No Visible Lesions	3	3	3	3	3	3	3	3
**SPINAL CORD, CERVICAL**								
Submitted	3	3	3	3	3	3	3	3
No Visible Lesions	3	3	3	3	3	3	3	3
**SPINAL CORD, LUMBAR**								
Submitted	3	3	3	3	3	3	3	3
No Visible Lesions	3	3	3	3	3	3	3	3
**SPINAL CORD, THORACIC**								
Submitted	3	3	3	3	3	3	3	3
No Visible Lesions	3	3	3	3	3	3	3	3
**SPLEEN**								
Submitted	3	3	3	3	3	3	3	3
No Visible Lesions	3	3	3	3	3	3	3	3
**STOMACH**								
Submitted	3	3	3	3	3	3	3	3
No Visible Lesions	3	3	3	2	3	3	3	3
Abnormal content; ingesta	0	0	0	1	0	0	0	0
**TESTIS**								
Submitted	3	3	3	3	.	.	.	.
No Visible Lesions	3	3	3	3	.	.	.	.
**THYMUS**								
Submitted	3	3	3	3	3	3	3	3
No Visible Lesions	3	3	3	3	3	3	3	3
**TONGUE**								
Submitted	3	3	3	3	3	3	3	3
No Visible Lesions	3	3	3	3	3	3	3	3

Appendix 21

Summary of Macroscopic Pathology: Terminal Euthanasia - Day 31

Removal Reason(s): TERMINAL EUTHANASIA	Male				Female			
Summary: Incidence	0 mg/kg /dose Group 1	3 mg/kg /dose Group 2	10 mg/kg /dose Group 3	100 mg/kg /dose Group 4	0 mg/kg /dose Group 1	3 mg/kg /dose Group 2	10 mg/kg /dose Group 3	100 mg/kg /dose Group 4
Number of Animals:	3	3	3	3	3	3	3	3
**TRACHEA**								
Submitted	3	3	3	3	3	3	3	3
No Visible Lesions	3	3	3	3	3	3	3	3
**URETER**								
Submitted	3	3	3	3	3	3	3	3
No Visible Lesions	3	3	3	3	3	3	3	3
**URINARY BLADDER**								
Submitted	3	3	3	3	3	3	3	3
No Visible Lesions	3	3	3	3	3	3	3	3
**UTERUS**								
Submitted	.	.	.	.	3	3	3	3
No Visible Lesions	.	.	.	.	2	3	3	3
Discoloration, dark; red	.	.	.	.	1	0	0	0
**VAGINA**								
Submitted	.	.	.	.	3	3	3	3
No Visible Lesions	.	.	.	.	3	3	3	3
**GLAND, SALIVARY, SUBMANDIBULAR**								
Submitted	3	3	3	3	3	3	3	3
No Visible Lesions	3	3	3	3	3	3	3	3

Appendix 21

Summary of Macroscopic Pathology: Recovery Euthanasia - Day 71

Removal Reason(s): RECOVERY EUTHANASIA	Male		Female	
Summary: Incidence	0 mg/kg /dose Group 1	100 mg/kg /dose Group 4	0 mg/kg /dose Group 1	100 mg/kg /dose Group 4
Number of Animals:	2	2	2	2
**ARTERY, AORTA**				
Submitted	2	2	2	2
No Visible Lesions	2	2	2	2
**BONE MARROW, STERNUM**				
Submitted	2	2	2	2
No Visible Lesions	2	2	2	2
**BONE, FEMUR**				
Submitted	2	2	2	2
No Visible Lesions	2	2	2	2
**BONE, STERNUM**				
Submitted	2	2	2	2
No Visible Lesions	2	2	2	2
**BRAIN**				
Submitted	2	2	2	2
No Visible Lesions	2	2	2	2
**CERVIX**				
Submitted	.	.	2	2
No Visible Lesions	.	.	2	2
**EPIDIDYMIS**				
Submitted	2	2	.	.
No Visible Lesions	2	2	.	.
**ESOPHAGUS**				
Submitted	2	2	2	2
No Visible Lesions	2	2	2	2
**EYE**				
Submitted	2	2	2	2
No Visible Lesions	2	2	2	2
**GALLBLADDER**				
Submitted	2	2	2	2
No Visible Lesions	2	2	2	2
**GALT**				
Submitted	2	2	2	2
No Visible Lesions	2	2	2	2
**GLAND, ADRENAL**				
Submitted	2	2	2	2

Appendix 21

Summary of Macroscopic Pathology: Recovery Euthanasia - Day 71

Removal Reason(s): RECOVERY EUTHANASIA	Male		Female	
Summary: Incidence	0 mg/kg /dose Group 1	100 mg/kg /dose Group 4	0 mg/kg /dose Group 1	100 mg/kg /dose Group 4
Number of Animals:	2	2	2	2
**GLAND, ADRENAL (Continued...)**				
No Visible Lesions	2	2	2	2
**GLAND, LACRIMAL**				
Submitted	2	2	2	2
No Visible Lesions	2	2	2	2
**GLAND, MAMMARY**				
Submitted	2	2	2	2
No Visible Lesions	2	2	2	2
**GLAND, PARATHYROID**				
Submitted	2	2	2	2
No Visible Lesions	2	2	2	2
**GLAND, PITUITARY**				
Submitted	2	2	2	2
No Visible Lesions	2	2	2	2
**GLAND, PROSTATE**				
Submitted	2	2	.	.
No Visible Lesions	2	2	.	.
**GLAND, SALIVARY, PAROTID**				
Submitted	2	2	2	2
No Visible Lesions	2	2	2	2
**GLAND, SALIVARY, SUBLINGUAL**				
Submitted	2	2	2	2
No Visible Lesions	2	2	2	2
**GLAND, SEMINAL VESICLE**				
Submitted	2	2	.	.
No Visible Lesions	2	2	.	.
**GLAND, THYROID**				
Submitted	2	2	2	2
No Visible Lesions	2	2	2	2
**HEART**				
Submitted	2	2	2	2
No Visible Lesions	2	2	2	2
**JOINT, FEMOROTIBIAL**				
Submitted	2	2	2	2
No Visible Lesions	2	2	2	2

Appendix 21

Summary of Macroscopic Pathology: Recovery Euthanasia - Day 71

Removal Reason(s): RECOVERY EUTHANASIA	Male		Female	
Summary: Incidence	0 mg/kg /dose Group 1	100 mg/kg /dose Group 4	0 mg/kg /dose Group 1	100 mg/kg /dose Group 4
Number of Animals:	2	2	2	2
**KIDNEY**				
Submitted	2	2	2	2
No Visible Lesions	2	2	2	2
**LARGE INTESTINE, CECUM**				
Submitted	2	2	2	2
No Visible Lesions	2	2	2	2
**LARGE INTESTINE, COLON**				
Submitted	2	2	2	2
No Visible Lesions	2	2	2	2
**LARGE INTESTINE, RECTUM**				
Submitted	2	2	2	2
No Visible Lesions	2	2	2	2
**LIVER**				
Submitted	2	2	2	2
No Visible Lesions	2	2	2	2
**LUNG**				
Submitted	2	2	2	2
No Visible Lesions	2	2	2	2
**LYMPH NODE, AXILLARY**				
Submitted	2	2	2	2
No Visible Lesions	2	2	2	2
**LYMPH NODE, MANDIBULAR**				
Submitted	2	2	2	2
No Visible Lesions	2	2	2	2
**LYMPH NODE, MESENTERIC**				
Submitted	2	2	2	2
No Visible Lesions	2	2	2	2
**MUSCLE, QUADRICEPS**				
Submitted	2	2	2	2
No Visible Lesions	2	2	2	2
**NERVE, OPTIC**				
Submitted	2	2	2	2
No Visible Lesions	2	2	2	2
**NERVE, SCIATIC**				
Submitted	2	2	2	2

Appendix 21

Summary of Macroscopic Pathology: Recovery Euthanasia - Day 71

Removal Reason(s): RECOVERY EUTHANASIA	Male		Female	
Summary: Incidence	0 mg/kg /dose Group 1	100 mg/kg /dose Group 4	0 mg/kg /dose Group 1	100 mg/kg /dose Group 4
Number of Animals:	2	2	2	2
**NERVE, SCIATIC (Continued...)**				
No Visible Lesions	2	2	2	2
**NERVE, TIBIAL**				
Submitted	2	2	2	2
No Visible Lesions	2	2	2	2
**OVARY**				
Submitted	.	.	2	2
No Visible Lesions	.	.	2	1
Enlargement	.	.	0	1
**OVIDUCT**				
Submitted	.	.	2	2
No Visible Lesions	.	.	2	2
**PANCREAS**				
Submitted	2	2	2	2
No Visible Lesions	2	2	2	2
**SITE, INFUSION, CEPHALIC, LEFT**				
Submitted	2	2	2	2
No Visible Lesions	2	2	2	2
**SITE, INFUSION, CEPHALIC, RIGHT**				
Submitted	2	2	2	2
No Visible Lesions	2	2	2	2
**SKIN**				
Submitted	2	2	2	2
No Visible Lesions	2	2	2	2
**SMALL INTESTINE, DUODENUM**				
Submitted	2	2	2	2
No Visible Lesions	2	2	2	2
**SMALL INTESTINE, ILEUM**				
Submitted	2	2	2	2
No Visible Lesions	2	2	2	2
**SMALL INTESTINE, JEJUNUM**				
Submitted	2	2	2	2
No Visible Lesions	2	2	2	2
**SPINAL CORD, CERVICAL**				
Submitted	2	2	2	2

Appendix 21

Summary of Macroscopic Pathology: Recovery Euthanasia - Day 71

Removal Reason(s): RECOVERY EUTHANASIA	Male		Female	
Summary: Incidence	0 mg/kg /dose Group 1	100 mg/kg /dose Group 4	0 mg/kg /dose Group 1	100 mg/kg /dose Group 4
Number of Animals:	2	2	2	2
**SPINAL CORD, CERVICAL (Continued...)**				
No Visible Lesions	2	2	2	2
**SPINAL CORD, LUMBAR**				
Submitted	2	2	2	2
No Visible Lesions	2	2	2	2
**SPINAL CORD, THORACIC**				
Submitted	2	2	2	2
No Visible Lesions	2	2	2	2
**SPLEEN**				
Submitted	2	2	2	2
No Visible Lesions	2	2	2	2
**STOMACH**				
Submitted	2	2	2	2
No Visible Lesions	2	2	2	2
**TESTIS**				
Submitted	2	2	.	.
No Visible Lesions	2	2	.	.
**THYMUS**				
Submitted	2	2	2	2
No Visible Lesions	2	2	1	2
Enlargement	0	0	1	0
**TONGUE**				
Submitted	2	2	2	2
No Visible Lesions	2	2	2	2
**TRACHEA**				
Submitted	2	2	2	2
No Visible Lesions	2	2	2	2
**URETER**				
Submitted	2	2	2	2
No Visible Lesions	2	2	2	2
**URINARY BLADDER**				
Submitted	2	2	2	2
No Visible Lesions	2	2	2	2
**UTERUS**				
Submitted	.	.	2	2

Appendix 21

Summary of Macroscopic Pathology: Recovery Euthanasia - Day 71

Removal Reason(s): RECOVERY EUTHANASIA	Male		Female	
Summary: Incidence	0 mg/kg /dose Group 1	100 mg/kg /dose Group 4	0 mg/kg /dose Group 1	100 mg/kg /dose Group 4
Number of Animals:	2	2	2	2
**UTERUS (Continued...)**				
No Visible Lesions	.	.	2	1
Discoloration, dark; red	.	.	0	1
**VAGINA**				
Submitted	.	.	2	2
No Visible Lesions	.	.	2	2
**GLAND, SALIVARY, SUBMANDIBULAR**				
Submitted	2	2	2	2
No Visible Lesions	2	2	2	2

Appendix 21

Summary of Organ Weights: Terminal Euthanasia - Day 31

Sex: Male<br>Day(s) Relative to Start Date		0 mg/kg /dose Group 1	3 mg/kg /dose Group 2	10 mg/kg /dose Group 3	100 mg/kg /dose Group 4
Terminal Body Weight (Kg) [G]	Mean	3.23	3.00	3.00	2.90
	SD	0.97	0.36	0.10	0.36
	N	3	3	3	3
	%Diff	-	-7.22	-7.22	-10.31
Brain Weight (g) [G]	Mean	66.4400	67.8133	70.0133	66.5200
	SD	1.3937	4.1728	3.5734	4.7970
	N	3	3	3	3
	%Diff	-	2.0670	5.3783	0.1204
Brain (%bw) [G]	Mean	2.17329	2.29229	2.33644	2.33298
	SD	0.60664	0.39802	0.16147	0.47893
	N	3	3	3	3
	%Diff	-	5.47551	7.50703	7.34796
Epididymis Weight (g) [G1]	Mean	1.1733	0.7400	0.8767	0.8167
	SD	1.0977	0.3143	0.1617	0.1850
	N	3	3	3	3
	%Diff	-	-36.9318	-25.2841	-30.3977
Epididymis (%br) [G1]	Mean	1.75063	1.09588	1.25229	1.23813
	SD	1.61192	0.46091	0.22741	0.33100
	N	3	3	3	3
	%Diff	-	-37.40079	-28.46607	-29.27511
Epididymis (%bw) [G1]	Mean	0.03230	0.02476	0.02936	0.02806
	SD	0.02118	0.01091	0.00639	0.00409
	N	3	3	3	3
	%Diff	-	-23.35387	-9.10345	-13.12892
Gland, Adrenal Weight (g) [G]	Mean	0.5633	0.6200	0.5067	0.6233
	SD	0.1358	0.0656	0.0513	0.1498
	N	3	3	3	3
	%Diff	-	10.0592	-10.0592	10.6509
Gland, Adrenal (%br) [G]	Mean	0.84602	0.91340	0.72535	0.94714
	SD	0.18989	0.06341	0.08955	0.27147
	N	3	3	3	3
	%Diff	-	7.96475	-14.26299	11.95322
Gland, Adrenal (%bw) [G]	Mean	0.01772	0.02099	0.01686	0.02134
	SD	0.00236	0.00417	0.00118	0.00292
	N	3	3	3	3
	%Diff	-	18.42940	-4.83127	20.42853

[G] - Anova & Dunnett
[G1] - Kruskal-Wallis & Dunn

Appendix 21

Summary of Organ Weights: Terminal Euthanasia - Day 31

Sex: Male<br>Day(s) Relative to Start Date		0 mg/kg /dose Group 1	3 mg/kg /dose Group 2	10 mg/kg /dose Group 3	100 mg/kg /dose Group 4
Gland, Pituitary Weight (g) [G]	Mean	0.0533	0.0333	0.0500	0.0433
	SD	0.0153	0.0208	0.0100	0.0058
	N	3	3	3	3
	%Diff	-	-37.5000	-6.2500	-18.7500
Gland, Pituitary (%br) [G]	Mean	0.08015	0.04995	0.07202	0.06554
	SD	0.02202	0.03145	0.01784	0.01150
	N	3	3	3	3
	%Diff	-	-37.68588	-10.15400	-18.23400
Gland, Pituitary (%bw) [G]	Mean	0.00165	0.00109	0.00166	0.00150
	SD	0.00002	0.00068	0.00031	0.00014
	N	3	3	3	3
	%Diff	-	-34.06933	0.63925	-9.38086
Prostate/Seminal Vesicle Wt (g) [G1]	Mean	4.0300	1.6967	1.6700	1.8067
	SD	4.9200	1.2158	0.8030	0.6591
	N	3	3	3	3
	%Diff	-	-57.8991	-58.5608	-55.1696
Prostate/Seminal Vesicle (%br) [G1]	Mean	5.99193	2.48661	2.38916	2.75718
	SD	7.25327	1.74059	1.16380	1.12348
	N	3	3	3	3
	%Diff	-	-58.50060	-60.12695	-53.98506
Prostate/Seminal Vesicle (%bw) [G1]	Mean	0.10530	0.05776	0.05626	0.06122
	SD	0.10473	0.04284	0.02889	0.01569
	N	3	3	3	3
	%Diff	-	-45.14673	-46.57014	-41.86006
Thyroid/Parathyroid Weight (g) [G]	Mean	0.2733	0.2233	0.2667	0.2333
	SD	0.0929	0.0577	0.0252	0.0379
	N	3	3	3	3
	%Diff	-	-18.2927	-2.4390	-14.6341
Thyroid/Parathyroid (%br) [G]	Mean	0.40998	0.33366	0.38275	0.35110
	SD	0.13223	0.10859	0.05491	0.05653
	N	3	3	3	3
	%Diff	-	-18.61616	-6.64303	-14.36231
Thyroid/Parathyroid (%bw) [G]	Mean	0.00847	0.00737	0.00889	0.00820
	SD	0.00133	0.00103	0.00077	0.00207
	N	3	3	3	3
	%Diff	-	-12.99095	4.89620	-3.21148

[G] - Anova & Dunnett
[G1] - Kruskal-Wallis & Dunn

Appendix 21

Summary of Organ Weights: Terminal Euthanasia - Day 31

Sex: Male<br>Day(s) Relative to Start Date		0 mg/kg /dose Group 1	3 mg/kg /dose Group 2	10 mg/kg /dose Group 3	100 mg/kg /dose Group 4
Heart Weight (g) [G]	Mean	14.1900	11.6600	13.7400	11.8667
	SD	5.9022	1.1112	2.0807	1.7116
	N	3	3	3	3
	%Diff	-	-17.8295	-3.1712	-16.3730
Heart (%br) [G1]	Mean	21.29737	17.29594	19.62819	18.01649
	SD	8.55045	2.60491	2.84642	3.70751
	N	3	3	3	3
	%Diff	-	-18.78839	-7.83751	-15.40509
Heart (%bw) [G1]	Mean	0.42953	0.38979	0.45715	0.40851
	SD	0.04776	0.02478	0.05980	0.01341
	N	3	3	3	3
	%Diff	-	-9.25056	6.43120	-4.89247
Kidney Weight (g) [G1]	Mean	15.6233	14.0300	15.2167	13.8133
	SD	3.8795	0.7308	0.6352	3.9992
	N	3	3	3	3
	%Diff	-	-10.1984	-2.6029	-11.5852
Kidney (%br) [G1]	Mean	23.50431	20.77648	21.80134	21.10376
	SD	5.69085	2.26825	1.98283	7.20304
	N	3	3	3	3
	%Diff	-	-11.60567	-7.24534	-10.21323
Kidney (%bw) [G1]	Mean	0.48798	0.47072	0.50751	0.46937
	SD	0.03862	0.04288	0.02455	0.08517
	N	3	3	3	3
	%Diff	-	-3.53609	4.00231	-3.81372
Liver/Gallbladder Weight (g) [G1]	Mean	65.6667	60.0767	60.8700	61.2400
	SD	18.2050	6.6446	2.6947	11.9200
	N	3	3	3	3
	%Diff	-	-8.5127	-7.3046	-6.7411
Liver/Gallbladder (%br) [G1]	Mean	98.73111	89.15400	86.98389	93.03694
	SD	26.42416	15.06436	2.61953	23.00112
	N	3	3	3	3
	%Diff	-	-9.70019	-11.89820	-5.76736
Liver/Gallbladder (%bw) [G1]	Mean	2.03889	2.00816	2.03218	2.10070
	SD	0.07884	0.16115	0.14999	0.17034
	N	3	3	3	3
	%Diff	-	-1.50737	-0.32914	3.03131

[G] - Kruskal-Wallis & Dunn
[G1] - Anova & Dunnett

Appendix 21

Summary of Organ Weights: Terminal Euthanasia - Day 31

Sex: Male<br>Day(s) Relative to Start Date		0 mg/kg /dose Group 1	3 mg/kg /dose Group 2	10 mg/kg /dose Group 3	100 mg/kg /dose Group 4
Spleen Weight (g) [G]	Mean	6.5767	6.3333	6.2267	6.5433
	SD	1.4647	0.3496	0.9577	0.9299
	N	3	3	3	3
	%Diff	-	-3.6999	-5.3218	-0.5068
Spleen (%br) [G]	Mean	9.88600	9.37015	8.91744	9.90805
	SD	2.08147	0.90395	1.50799	1.88099
	N	3	3	3	3
	%Diff	-	-5.21797	-9.79736	0.22301
Spleen (%bw) [G]	Mean	0.20659	0.21287	0.20831	0.22583
	SD	0.01670	0.02547	0.03851	0.01727
	N	3	3	3	3
	%Diff	-	3.04112	0.83199	9.31588
Testis Weight (g) [G1]	Mean	6.7433	1.2767	1.7267	1.3600
	SD	9.2044	0.3431	0.6117	0.3724
	N	3	3	3	3
	%Diff	-	-81.0677	-74.3945	-79.8319
Testis (%br) [G1]	Mean	10.01648	1.90715	2.48125	2.06755
	SD	13.58431	0.62025	0.91594	0.65714
	N	3	3	3	3
	%Diff	-	-80.95986	-75.22835	-79.35853
Testis (%bw) [G1]	Mean	0.16996	0.04211	0.05793	0.04648
	SD	0.20265	0.00729	0.02223	0.00798
	N	3	3	3	3
	%Diff	-	-75.22301	-65.91664	-72.65480
Thymus Weight (g) [G]	Mean	2.8067	2.3733	2.4900	3.3333
	SD	1.2208	0.5150	0.8229	1.1212
	N	3	3	3	3
	%Diff	-	-15.4394	-11.2827	18.7648
Thymus (%br) [G]	Mean	4.25147	3.53754	3.54157	5.10541
	SD	1.94509	0.96420	1.07319	1.97005
	N	3	3	3	3
	%Diff	-	-16.79264	-16.69774	20.08560
Thymus (%bw) [G]	Mean	0.09377	0.07861	0.08270	0.11285
	SD	0.04811	0.01013	0.02640	0.02718
	N	3	3	3	3
	%Diff	-	-16.16185	-11.80008	20.34567

[G] - Anova & Dunnett
[G1] - Kruskal-Wallis & Dunn

Appendix 21

Summary of Organ Weights: Terminal Euthanasia - Day 31

Sex: Female<br>Day(s) Relative to Start Date		0 mg/kg /dose Group 1	3 mg/kg /dose Group 2	10 mg/kg /dose Group 3	100 mg/kg /dose Group 4
Terminal Body Weight (Kg) [G]	Mean	2.97	2.77	2.73	2.90
	SD	0.35	0.15	0.15	0.53
	N	3	3	3	3
	%Diff	-	-6.74	-7.87	-2.25
Brain Weight (g) [G]	Mean	68.3833	58.7233	57.5133	60.3733
	SD	4.1531	2.4609	2.4913	7.8059
	N	3	3	3	3
	%Diff	-	-14.1262	-15.8957	-11.7134
Brain (%bw) [G]	Mean	2.31585	2.12816	2.10598	2.10932
	SD	0.13687	0.17097	0.07967	0.33514
	N	3	3	3	3
	%Diff	-	-8.10469	-9.06247	-8.91823
Gland, Adrenal Weight (g) [G]	Mean	0.6033	0.6167	0.6067	0.6400
	SD	0.1365	0.0551	0.1405	0.1229
	N	3	3	3	3
	%Diff	-	2.2099	0.5525	6.0773
Gland, Adrenal (%br) [G]	Mean	0.87754	1.05088	1.04950	1.06951
	SD	0.15142	0.09541	0.20547	0.21583
	N	3	3	3	3
	%Diff	-	19.75322	19.59657	21.87693
Gland, Adrenal (%bw) [G]	Mean	0.02022	0.02241	0.02216	0.02209
	SD	0.00257	0.00329	0.00490	0.00162
	N	3	3	3	3
	%Diff	-	10.84922	9.61872	9.25111
Gland, Pituitary Weight (g) [G]	Mean	0.0567	0.0567	0.0367	0.0467
	SD	0.0115	0.0115	0.0208	0.0058
	N	3	3	3	3
	%Diff	-	0.0000	-35.2941	-17.6471
Gland, Pituitary (%br) [G1]	Mean	0.08253	0.09656	0.06306	0.07735
	SD	0.01277	0.01959	0.03412	0.00214
	N	3	3	3	3
	%Diff	-	17.00060	-23.59431	-6.27457
Gland, Pituitary (%bw) [G]	Mean	0.00190	0.00207	0.00134	0.00163
	SD	0.00023	0.00054	0.00077	0.00021
	N	3	3	3	3
	%Diff	-	8.60109	-29.50504	-14.54279

[G] - Anova & Dunnett
[G1] - Kruskal-Wallis & Dunn

Appendix 21

Summary of Organ Weights: Terminal Euthanasia - Day 31

Sex: Female<br>Day(s) Relative to Start Date		0 mg/kg /dose Group 1	3 mg/kg /dose Group 2	10 mg/kg /dose Group 3	100 mg/kg /dose Group 4
Thyroid/Parathyroid Weight (g) [G]	Mean	0.3500	0.2067	0.2500	0.2867
	SD	0.1652	0.0153	0.0361	0.0231
	N	3	3	3	3
	%Diff	-	-40.9524	-28.5714	-18.0952
Thyroid/Parathyroid (%br) [G]	Mean	0.50592	0.35277	0.43577	0.48169
	SD	0.21255	0.03612	0.06950	0.08840
	N	3	3	3	3
	%Diff	-	-30.27034	-13.86578	-4.78790
Thyroid/Parathyroid (%bw) [G1]	Mean	0.01158	0.00750	0.00914	0.01018
	SD	0.00431	0.00087	0.00120	0.00242
	N	3	3	3	3
	%Diff	-	-35.28126	-21.05229	-12.11160
Heart Weight (g) [G1]	Mean	11.2233	9.5167	9.1900	9.9367
	SD	1.5528	1.4647	0.7264	1.8570
	N	3	3	3	3
	%Diff	-	-15.2064	-18.1170	-11.4642
Heart (%br) [G1]	Mean	16.36031	16.28245	15.97754	16.55361
	SD	1.28884	3.14147	1.00409	2.92502
	N	3	3	3	3
	%Diff	-	-0.47595	-2.33962	1.18151
Heart (%bw) [G]	Mean	0.37771	0.34300	0.33596	0.34264
	SD	0.00773	0.03688	0.00859	0.01123
	N	3	3	3	3
	%Diff	-	-9.18833	-11.05385	-9.28480
Kidney Weight (g) [G1]	Mean	13.6667	13.1533	12.0767	13.7633
	SD	1.4845	1.5927	1.1657	1.5643
	N	3	3	3	3
	%Diff	-	-3.7561	-11.6341	0.7073
Kidney (%br) [G1]	Mean	19.95025	22.50066	20.97139	22.87198
	SD	1.03326	3.65431	1.29123	1.67839
	N	3	3	3	3
	%Diff	-	12.78383	5.11842	14.64506
Kidney (%bw) [G1]	Mean	0.46123	0.47587	0.44122	0.47876
	SD	0.01420	0.05615	0.02073	0.04138
	N	3	3	3	3
	%Diff	-	3.17612	-4.33686	3.80278

[G] - Kruskal-Wallis & Dunn
[G1] - Anova & Dunnett

Appendix 21

Summary of Organ Weights: Terminal Euthanasia - Day 31

Sex: Female<br>Day(s) Relative to Start Date		0 mg/kg /dose Group 1	3 mg/kg /dose Group 2	10 mg/kg /dose Group 3	100 mg/kg /dose Group 4
Liver/Gallbladder Weight (g) [G]	Mean	67.6800	57.9267	54.2733	65.0067
	SD	11.0386	2.8223	2.6558	7.0583
	N	3	3	3	3
	%Diff	-	-14.4110	-19.8089	-3.9500
Liver/Gallbladder (%br) [G]	Mean	98.55762	98.89304	94.50615	108.29484
	SD	10.50049	9.00460	6.56843	11.26524
	N	3	3	3	3
	%Diff	-	0.34033	-4.11077	9.87972
Liver/Gallbladder (%bw) [G]	Mean	2.27313	2.09603	1.98768	2.26059
	SD	0.12363	0.10539	0.09893	0.15840
	N	3	3	3	3
	%Diff	-	-7.79102	-12.55746	-0.55157
Ovary Paired (g) [G]	Mean	0.2233	0.2567	0.2467	0.3100
	SD	0.0321	0.0907	0.0862	0.1562
	N	3	3	3	3
	%Diff	-	14.9254	10.4478	38.8060
Ovary Paired (%br) [G]	Mean	0.32929	0.43368	0.42614	0.50284
	SD	0.06818	0.13599	0.13715	0.20325
	N	3	3	3	3
	%Diff	-	31.69956	29.41132	52.70215
Ovary Paired (%bw) [G]	Mean	0.00769	0.00929	0.00893	0.01104
	SD	0.00206	0.00323	0.00262	0.00622
	N	3	3	3	3
	%Diff	-	20.85721	16.13363	43.61972
Spleen Weight (g) [G]	Mean	7.4000	5.7200	6.0200	4.9033
	SD	2.2687	0.6560	1.2946	0.6947
	N	3	3	3	3
	%Diff	-	-22.7027	-18.6486	-33.7387
Spleen (%br) [G]	Mean	10.72045	9.73221	10.52158	8.14670
	SD	2.67155	0.88956	2.56854	0.86965
	N	3	3	3	3
	%Diff	-	-9.21829	-1.85506	-24.00781
Spleen (%bw) [G]	Mean	0.24599	0.20676	0.22241	0.16990
	SD	0.04747	0.02088	0.05836	0.00851
	N	3	3	3	3
	%Diff	-	-15.94916	-9.58765	-30.93421

[G] - Anova & Dunnett

Appendix 21

Summary of Organ Weights: Terminal Euthanasia - Day 31

Sex: Female<br>Day(s) Relative to Start Date		0 mg/kg /dose Group 1	3 mg/kg /dose Group 2	10 mg/kg /dose Group 3	100 mg/kg /dose Group 4
Thymus Weight (g) [G]	Mean	4.2567	2.9533	2.8400	2.6100
	SD	1.4924	0.6910	0.1153	0.6582
	N	3	3	3	3
	%Diff	-	-30.6186	-33.2811	-38.6844
Thymus (%br) [G]	Mean	6.15071	5.06605	4.95018	4.34144
	SD	1.85083	1.36724	0.42357	1.00059
	N	3	3	3	3
	%Diff	-	-17.63476	-19.51858	-29.41566
Thymus (%bw) [G1]	Mean	0.14077	0.10694	0.10421	0.08936
	SD	0.03554	0.02519	0.00907	0.00685
	N	3	3	3	3
	%Diff	-	-24.03588	-25.97287	-36.52307
Uterus/Cervix (g) [G]	Mean	6.6767	4.6467	4.0033	5.6433
	SD	1.4684	1.0651	1.4049	0.7617
	N	3	3	3	3
	%Diff	-	-30.4044	-40.0399	-15.4768
Uterus/Cervix (%br) [G]	Mean	9.78479	7.87180	6.89906	9.35946
	SD	2.19726	1.51455	2.19664	0.59733
	N	3	3	3	3
	%Diff	-	-19.55068	-29.49205	-4.34686
Uterus/Cervix (%bw) [G]	Mean	0.22707	0.16907	0.14568	0.19853
	SD	0.05624	0.04321	0.04895	0.04201
	N	3	3	3	3
	%Diff	-	-25.54196	-35.84645	-12.56851

[G] - Anova & Dunnett
[G1] - Kruskal-Wallis & Dunn

Appendix 21

Summary of Organ Weights: Recovery Euthanasia - Day 71

Sex: Male<br>Day(s) Relative to Start Date		0 mg/kg /dose Group 1	100 mg/kg /dose Group 4
Terminal Body Weight (Kg)	Mean	3.35	3.00
	SD	0.35	0.85
	N	2	2
	%Diff	-	-10.45
Brain Weight (g)	Mean	66.8800	71.1700
	SD	6.2508	6.7034
	N	2	2
	%Diff	-	6.4145
Brain (%bw)	Mean	2.01750	2.43826
	SD	0.39952	0.46620
	N	2	2
	%Diff	-	20.85571
Epididymis Weight (g)	Mean	2.1600	1.8250
	SD	1.6546	1.8314
	N	2	2
	%Diff	-	-15.5093
Epididymis (%br)	Mean	3.35996	2.45398
	SD	2.78806	2.34215
	N	2	2
	%Diff	-	-26.96388
Epididymis (%bw)	Mean	0.06222	0.05438
	SD	0.04283	0.04567
	N	2	2
	%Diff	-	-12.60531
Gland, Adrenal Weight (g)	Mean	0.5300	0.4600
	SD	0.0849	0.1980
	N	2	2
	%Diff	-	-13.2075
Gland, Adrenal (%br)	Mean	0.80190	0.63606
	SD	0.20182	0.21828
	N	2	2
	%Diff	-	-20.68046
Gland, Adrenal (%bw)	Mean	0.01578	0.01500
	SD	0.00087	0.00236
	N	2	2
	%Diff	-	-4.91338

Appendix 21

Summary of Organ Weights: Recovery Euthanasia - Day 71

Sex: Male<br><br>Day(s) Relative to Start Date		0 mg/kg /dose Group 1	100 mg/kg /dose Group 4
Gland, Pituitary Weight (g)	Mean	0.0500	0.0400
	SD	0.0000	0.0283
	N	2	2
	%Diff	-	-20.0000
Gland, Pituitary (%br)	Mean	0.07509	0.05457
	SD	0.00702	0.03460
	N	2	2
	%Diff	-	-27.32075
Gland, Pituitary (%bw)	Mean	0.00150	0.00125
	SD	0.00016	0.00059
	N	2	2
	%Diff	-	-16.71642
Prostate/Seminal Vesicle Wt (g)	Mean	3.9350	3.7150
	SD	4.0376	3.4295
	N	2	2
	%Diff	-	-5.5909
Prostate/Seminal Vesicle (%br)	Mean	6.19284	5.01521
	SD	6.61585	4.34633
	N	2	2
	%Diff	-	-19.01602
Prostate/Seminal Vesicle (%bw)	Mean	0.11172	0.11215
	SD	0.10873	0.08259
	N	2	2
	%Diff	-	0.38297
Thyroid/Parathyroid Weight (g)	Mean	0.2750	0.3400
	SD	0.0212	0.0424
	N	2	2
	%Diff	-	23.6364
Thyroid/Parathyroid (%br)	Mean	0.41448	0.47704
	SD	0.07046	0.01468
	N	2	2
	%Diff	-	15.09402
Thyroid/Parathyroid (%bw)	Mean	0.00822	0.01160
	SD	0.00023	0.00187
	N	2	2
	%Diff	-	41.06267

Appendix 21

Summary of Organ Weights: Recovery Euthanasia - Day 71

Sex: Male<br>Day(s) Relative to Start Date		0 mg/kg /dose Group 1	100 mg/kg /dose Group 4
Heart Weight (g)	Mean	13.5900	11.8450
	SD	0.6505	3.2739
	N	2	2
	%Diff	-	-12.8403
Heart (%br)	Mean	20.45477	16.49980
	SD	2.88446	3.04603
	N	2	2
	%Diff	-	-19.33521
Heart (%bw)	Mean	0.40691	0.39521
	SD	0.02353	0.00265
	N	2	2
	%Diff	-	-2.87647
Kidney Weight (g)	Mean	16.0900	13.3850
	SD	3.2810	3.0476
	N	2	2
	%Diff	-	-16.8117
Kidney (%br)	Mean	24.39381	18.68831
	SD	7.18569	2.52197
	N	2	2
	%Diff	-	-23.38913
Kidney (%bw)	Mean	0.47779	0.44979
	SD	0.04751	0.02563
	N	2	2
	%Diff	-	-5.86021
Liver/Gallbladder Weight (g)	Mean	73.7300	62.4450
	SD	12.9401	19.4101
	N	2	2
	%Diff	-	-15.3058
Liver/Gallbladder (%br)	Mean	111.63398	86.84143
	SD	29.78184	19.09341
	N	2	2
	%Diff	-	-22.20878
Liver/Gallbladder (%bw)	Mean	2.19272	2.07292
	SD	0.15485	0.06069
	N	2	2
	%Diff	-	-5.46386

Appendix 21

Summary of Organ Weights: Recovery Euthanasia - Day 71

Sex: Male<br>Day(s) Relative to Start Date		0 mg/kg /dose Group 1	100 mg/kg /dose Group 4
Spleen Weight (g)	Mean	7.2200	6.1150
	SD	3.5638	0.2051
	N	2	2
	%Diff	-	-15.3047
Spleen (%br)	Mean	11.09292	8.61676
	SD	6.36546	0.52347
	N	2	2
	%Diff	-	-22.32204
Spleen (%bw)	Mean	0.21108	0.21132
	SD	0.08411	0.05293
	N	2	2
	%Diff	-	0.11143
Testis Weight (g)	Mean	15.2250	12.2650
	SD	19.0990	15.7473
	N	2	2
	%Diff	-	-19.4417
Testis (%br)	Mean	24.20489	16.26351
	SD	30.81932	20.59444
	N	2	2
	%Diff	-	-32.80900
Testis (%bw)	Mean	0.42677	0.34854
	SD	0.52508	0.42633
	N	2	2
	%Diff	-	-18.33027
Thymus Weight (g)	Mean	6.0850	4.6500
	SD	2.5244	1.3435
	N	2	2
	%Diff	-	-23.5826
Thymus (%br)	Mean	9.31546	6.47346
	SD	4.64513	1.27801
	N	2	2
	%Diff	-	-30.50837
Thymus (%bw)	Mean	0.17866	0.15486
	SD	0.05650	0.00098
	N	2	2
	%Diff	-	-13.32096

Appendix 21

Summary of Organ Weights: Recovery Euthanasia - Day 71

Sex: Female<br>Day(s) Relative to Start Date		0 mg/kg /dose Group 1	100 mg/kg /dose Group 4
Terminal Body Weight (Kg)	Mean	2.70	2.80
	SD	0.00	0.14
	N	2	2
	%Diff	-	3.70
Brain Weight (g)	Mean	65.3400	56.8900
	SD	1.1738	1.3294
	N	2	2
	%Diff	-	-12.9324
Brain (%bw)	Mean	2.42000	2.03318
	SD	0.04347	0.05521
	N	2	2
	%Diff	-	-15.98429
Gland, Adrenal Weight (g)	Mean	0.5500	0.5700
	SD	0.0000	0.0707
	N	2	2
	%Diff	-	3.6364
Gland, Adrenal (%br)	Mean	0.84189	1.00075
	SD	0.01512	0.10091
	N	2	2
	%Diff	-	18.87046
Gland, Adrenal (%bw)	Mean	0.02037	0.02032
	SD	0.00000	0.00150
	N	2	2
	%Diff	-	-0.25078
Gland, Pituitary Weight (g)	Mean	0.0450	0.0350
	SD	0.0212	0.0071
	N	2	2
	%Diff	-	-22.2222
Gland, Pituitary (%br)	Mean	0.06917	0.06168
	SD	0.03371	0.01387
	N	2	2
	%Diff	-	-10.82644
Gland, Pituitary (%bw)	Mean	0.00167	0.00126
	SD	0.00079	0.00032
	N	2	2
	%Diff	-	-24.52107

Appendix 21

Summary of Organ Weights: Recovery Euthanasia - Day 71

Sex: Female<br>Day(s) Relative to Start Date		0 mg/kg /dose Group 1	100 mg/kg /dose Group 4
Thyroid/Parathyroid Weight (g)	Mean	0.2350	0.2500
	SD	0.1202	0.0566
	N	2	2
	%Diff	-	6.3830
Thyroid/Parathyroid (%br)	Mean	0.36137	0.43840
	SD	0.19047	0.08919
	N	2	2
	%Diff	-	21.31747
Thyroid/Parathyroid (%bw)	Mean	0.00870	0.00889
	SD	0.00445	0.00157
	N	2	2
	%Diff	-	2.12766
Heart Weight (g)	Mean	8.8950	9.9750
	SD	0.6293	1.7748
	N	2	2
	%Diff	-	12.1417
Heart (%br)	Mean	13.62426	17.50217
	SD	1.20791	2.71079
	N	2	2
	%Diff	-	28.46327
Heart (%bw)	Mean	0.32944	0.35510
	SD	0.02331	0.04545
	N	2	2
	%Diff	-	7.78818
Kidney Weight (g)	Mean	13.4550	13.4300
	SD	0.8697	0.7212
	N	2	2
	%Diff	-	-0.1858
Kidney (%br)	Mean	20.60757	23.59859
	SD	1.70130	0.71636
	N	2	2
	%Diff	-	14.51420
Kidney (%bw)	Mean	0.49833	0.47960
	SD	0.03221	0.00154
	N	2	2
	%Diff	-	-3.75838

Appendix 21

Summary of Organ Weights: Recovery Euthanasia - Day 71

Sex: Female<br>Day(s) Relative to Start Date		0 mg/kg /dose Group 1	100 mg/kg /dose Group 4
Liver/Gallbladder Weight (g)	Mean	56.4050	61.9300
	SD	8.5772	7.1418
	N	2	2
	%Diff	-	9.7952
Liver/Gallbladder (%br)	Mean	86.45724	108.74222
	SD	14.68019	10.01266
	N	2	2
	%Diff	-	25.77573
Liver/Gallbladder (%bw)	Mean	2.08907	2.20816
	SD	0.31767	0.14353
	N	2	2
	%Diff	-	5.70046
Ovary Paired (g)	Mean	0.2700	0.3900
	SD	0.0849	0.2687
	N	2	2
	%Diff	-	44.4444
Ovary Paired (%br)	Mean	0.41446	0.68020
	SD	0.13731	0.45642
	N	2	2
	%Diff	-	64.11876
Ovary Paired (%bw)	Mean	0.01000	0.01370
	SD	0.00314	0.00890
	N	2	2
	%Diff	-	37.03704
Spleen Weight (g)	Mean	6.6250	5.7250
	SD	0.0071	0.3182
	N	2	2
	%Diff	-	-13.5849
Spleen (%br)	Mean	10.14081	10.05949
	SD	0.17135	0.32426
	N	2	2
	%Diff	-	-0.80190
Spleen (%bw)	Mean	0.24537	0.20444
	SD	0.00026	0.00104
	N	2	2
	%Diff	-	-16.68185

Appendix 21

Summary of Organ Weights: Recovery Euthanasia - Day 71

Sex: Female<br>Day(s) Relative to Start Date		0 mg/kg /dose Group 1	100 mg/kg /dose Group 4
Thymus Weight (g)	Mean	4.1550	2.0150
	SD	2.0011	1.2092
	N	2	2
	%Diff	-	-51.5042
Thymus (%br)	Mean	6.33256	3.51805
	SD	2.94885	2.04321
	N	2	2
	%Diff	-	-44.44502
Thymus (%bw)	Mean	0.15389	0.07096
	SD	0.07412	0.03960
	N	2	2
	%Diff	-	-53.88605
Uterus/Cervix (g)	Mean	6.3900	6.7550
	SD	3.3658	0.2475
	N	2	2
	%Diff	-	5.7121
Uterus/Cervix (%br)	Mean	9.82747	11.88212
	SD	5.32780	0.71268
	N	2	2
	%Diff	-	20.90720
Uterus/Cervix (%bw)	Mean	0.23667	0.24178
	SD	0.12466	0.02105
	N	2	2
	%Diff	-	2.16124

Appendix 21

Summary of Microscopic Pathology: Terminal Euthanasia - Day 31

Removal Reason(s): TERMINAL EUTHANASIA<br>Summary: Incidence	Male				Female			
	0 mg/kg /dose Group 1	3 mg/kg /dose Group 2	10 mg/kg /dose Group 3	100 mg/kg /dose Group 4	0 mg/kg /dose Group 1	3 mg/kg /dose Group 2	10 mg/kg /dose Group 3	100 mg/kg /dose Group 4
Number of Animals:	3	3	3	3	3	3	3	3
**ARTERY, AORTA**								
Examined	3	3	3	3	3	3	3	3
No Visible Lesions	3	3	3	3	3	3	3	3
**BONE MARROW, STERNUM**								
Examined	3	3	3	3	3	3	3	3
No Visible Lesions	3	3	3	3	3	3	3	3
**BONE, FEMUR**								
Examined	3	3	3	3	3	3	3	3
No Visible Lesions	3	3	3	3	3	3	3	3
**BONE, SKULL**								
Examined	.	.	.	.	0	0	1	0
Fracture; chronic	.	.	.	.	.	.	1	.
**BONE, STERNUM**								
Examined	3	3	3	3	3	3	3	3
No Visible Lesions	3	3	3	3	3	3	3	3
**BRAIN**								
Examined	3	3	3	3	3	3	3	3
No Visible Lesions	3	3	3	3	3	3	3	3
**CERVIX**								
Examined	.	.	.	.	3	3	3	3
No Visible Lesions	.	.	.	.	3	3	3	3
**EPIDIDYMIS**								
Examined	3	3	3	3	.	.	.	.
No Visible Lesions	3	3	2	3	.	.	.	.
Infiltration, mixed cell	0	0	1	0	.	.	.	.
.... mild	0	0	1	0	.	.	.	.
**ESOPHAGUS**								
Examined	3	3	3	3	3	3	3	3
No Visible Lesions	3	3	3	3	3	3	3	3
**EYE**								
Examined	3	3	3	3	3	3	3	3
No Visible Lesions	3	3	3	3	3	3	3	3
**GALLBLADDER**								
Examined	3	3	3	3	3	3	3	3
No Visible Lesions	3	3	3	3	3	3	3	3

Appendix 21

Summary of Microscopic Pathology: Terminal Euthanasia - Day 31

Removal Reason(s): TERMINAL EUTHANASIA Summary: Incidence	Male				Female			
	0 mg/kg /dose Group 1	3 mg/kg /dose Group 2	10 mg/kg /dose Group 3	100 mg/kg /dose Group 4	0 mg/kg /dose Group 1	3 mg/kg /dose Group 2	10 mg/kg /dose Group 3	100 mg/kg /dose Group 4
Number of Animals:	3	3	3	3	3	3	3	3
**GALT**								
Examined	3	3	3	3	3	3	3	3
No Visible Lesions	3	3	3	3	3	3	3	3
**GLAND, ADRENAL**								
Examined	3	3	3	3	3	3	3	3
No Visible Lesions	2	2	3	3	3	2	2	3
Vacuolation; cortical	1	0	0	0	0	0	0	0
.... minimal	1	0	0	0	0	0	0	0
Infiltration, mononuclear cell	0	1	0	0	0	0	0	0
.... minimal	0	1	0	0	0	0	0	0
Mineralization	0	0	0	0	0	0	1	0
.... minimal	0	0	0	0	0	0	1	0
Infiltration, lymphocytic	0	0	0	0	0	1	0	0
.... minimal	0	0	0	0	0	1	0	0
**GLAND, MAMMARY**								
Examined	3	3	3	2	3	3	3	3
No Visible Lesions	3	3	3	2	3	3	3	3
Not Examined: Not Present In Section.	0	0	0	1	.	.	.	.
**GLAND, PARATHYROID**								
Examined	1	3	2	2	3	2	3	3
No Visible Lesions	1	3	1	2	3	2	3	3
Not Examined: Not Present In Section.	2	0	1	1	0	1	0	0
Cyst	0	0	1	0	0	0	0	0
**GLAND, PITUITARY**								
Examined	3	3	3	3	3	3	3	3
No Visible Lesions	3	3	2	3	3	3	3	3
Infiltration, lymphocytic; pars intermedia	0	0	1	0	0	0	0	0
.... minimal	0	0	1	0	0	0	0	0
**GLAND, PROSTATE**								
Examined	3	3	3	3	.	.	.	.
No Visible Lesions	3	3	3	3	.	.	.	.

Appendix 21

Summary of Microscopic Pathology: Terminal Euthanasia - Day 31

Removal Reason(s): TERMINAL EUTHANASIA<br>Summary: Incidence	Male				Female			
	0 mg/kg /dose Group 1	3 mg/kg /dose Group 2	10 mg/kg /dose Group 3	100 mg/kg /dose Group 4	0 mg/kg /dose Group 1	3 mg/kg /dose Group 2	10 mg/kg /dose Group 3	100 mg/kg /dose Group 4
Number of Animals:	3	3	3	3	3	3	3	3
**GLAND, SEMINAL VESICLE**								
Examined	3	3	3	3	.	.	.	.
No Visible Lesions	3	3	3	3	.	.	.	.
**GLAND, THYROID**								
Examined	3	3	3	3	3	3	3	3
No Visible Lesions	2	2	2	3	3	1	2	1
Cyst	1	0	0	0	0	0	0	1
Infiltration, mononuclear cell	0	1	1	0	0	1	0	0
.... minimal	0	1	1	0	0	1	0	0
Ectopia	0	0	0	0	0	0	1	1
Infiltration, lymphocytic	0	0	0	0	0	1	0	0
.... mild	0	0	0	0	0	1	0	0
**HEART**								
Examined	3	3	3	3	3	3	3	3
No Visible Lesions	3	3	2	3	3	3	2	3
Infiltration, mononuclear cell	0	0	0	0	0	0	1	0
.... minimal	0	0	0	0	0	0	1	0
Cyst; squamous	0	0	1	0	0	0	0	0
**JOINT, FEMOROTIBIAL**								
Examined	3	3	3	3	3	3	3	3
No Visible Lesions	3	3	3	3	3	3	3	3
**KIDNEY**								
Examined	3	3	3	3	3	3	3	3
No Visible Lesions	3	1	2	1	1	2	3	1
Infiltration, mononuclear cell	0	2	1	2	2	1	0	2
.... minimal	0	2	1	2	2	1	0	2
**LARGE INTESTINE, CECUM**								
Examined	3	3	3	3	3	3	3	3
No Visible Lesions	3	3	3	3	3	3	3	3
**LARGE INTESTINE, COLON**								
Examined	3	3	3	3	3	3	3	3
No Visible Lesions	3	3	3	3	3	3	3	3
**LARGE INTESTINE, RECTUM**								
Examined	3	3	3	3	3	3	3	3

Appendix 21

Summary of Microscopic Pathology: Terminal Euthanasia - Day 31

Removal Reason(s): TERMINAL EUTHANASIA<br>Summary: Incidence	Male				Female			
	0 mg/kg /dose Group 1	3 mg/kg /dose Group 2	10 mg/kg /dose Group 3	100 mg/kg /dose Group 4	0 mg/kg /dose Group 1	3 mg/kg /dose Group 2	10 mg/kg /dose Group 3	100 mg/kg /dose Group 4
Number of Animals:	3	3	3	3	3	3	3	3
**LARGE INTESTINE, RECTUM (Continued...)**								
No Visible Lesions	3	3	3	3	3	3	3	3
**LIVER**								
Examined	3	3	3	3	3	3	3	3
No Visible Lesions	3	1	3	1	3	3	2	2
Infiltration, mononuclear cell	0	2	0	2	0	0	1	1
.... minimal	0	2	0	1	0	0	1	1
.... mild	0	0	0	1	0	0	0	0
**LUNG**								
Examined	3	3	3	3	3	3	3	3
No Visible Lesions	3	3	2	2	1	3	2	1
Fibrosis; pleural	0	0	0	0	1	0	0	0
.... mild	0	0	0	0	1	0	0	0
Infiltration, mononuclear cell	0	0	1	0	1	0	1	2
.... minimal	0	0	1	0	1	0	1	2
Alveolar macrophages, increased	0	0	0	1	0	0	0	0
.... mild	0	0	0	1	0	0	0	0
**LYMPH NODE, AXILLARY**								
Examined	3	3	3	3	3	3	3	3
No Visible Lesions	3	2	3	3	2	2	1	3
Pigment	0	1	0	0	1	1	2	0
.... minimal	0	0	0	0	1	0	1	0
.... mild	0	1	0	0	0	1	1	0
**LYMPH NODE, MANDIBULAR**								
Examined	3	3	3	3	3	3	3	3
No Visible Lesions	3	3	3	3	3	3	3	3
**LYMPH NODE, MESENTERIC**								
Examined	3	3	3	3	3	3	3	3
No Visible Lesions	3	3	3	3	3	3	3	3
**MUSCLE, QUADRICEPS**								
Examined	3	3	3	3	3	3	3	3
No Visible Lesions	3	3	3	3	2	3	3	3
Inflammation; chronic	0	0	0	0	1	0	0	0

Appendix 21

Summary of Microscopic Pathology: Terminal Euthanasia - Day 31

Removal Reason(s): TERMINAL EUTHANASIA<br>Summary: Incidence	Male				Female			
	0 mg/kg /dose Group 1	3 mg/kg /dose Group 2	10 mg/kg /dose Group 3	100 mg/kg /dose Group 4	0 mg/kg /dose Group 1	3 mg/kg /dose Group 2	10 mg/kg /dose Group 3	100 mg/kg /dose Group 4
Number of Animals:	3	3	3	3	3	3	3	3
**MUSCLE, QUADRICEPS (Continued...)**								
.... mild	0	0	0	0	1	0	0	0
**NERVE, OPTIC**								
Examined	3	3	3	3	3	3	3	3
No Visible Lesions	3	3	3	3	3	3	3	3
**NERVE, SCIATIC**								
Examined	3	3	3	3	3	3	3	3
No Visible Lesions	3	3	3	3	3	3	3	3
**OVARY**								
Examined	.	.	.	.	3	3	3	3
No Visible Lesions	.	.	.	.	1	2	3	2
Cyst	.	.	.	.	1	0	0	0
Cyst; luteal	.	.	.	.	0	0	0	1
Immaturity	.	.	.	.	1	0	0	0
Mineralization	.	.	.	.	0	1	0	0
.... minimal	.	.	.	.	0	1	0	0
Hypertrophy; corpus luteum	.	.	.	.	1	0	0	0
.... moderate	.	.	.	.	1	0	0	0
**OVIDUCT**								
Not Examined: Not Present In Section.	.	.	.	.	0	1	0	0
**PANCREAS**								
Examined	3	3	3	3	3	3	3	3
No Visible Lesions	2	3	3	3	3	3	3	3
Infiltration, mononuclear cell	1	0	0	0	0	0	0	0
.... mild	1	0	0	0	0	0	0	0
**SITE, INFUSION, CEPHALIC, LEFT**								
Examined	3	3	3	3	3	3	3	3
No Visible Lesions	3	2	0	2	3	3	3	3
Infiltration, mixed cell; subcutaneous tissue	0	1	3	1	0	0	0	0
.... minimal	0	1	2	0	0	0	0	0
.... mild	0	0	1	1	0	0	0	0
Hemorrhage; subcutaneous tissue	0	0	1	0	0	0	0	0

Appendix 21

Summary of Microscopic Pathology: Terminal Euthanasia - Day 31

Removal Reason(s): TERMINAL EUTHANASIA<br>Summary: Incidence	Male				Female			
	0 mg/kg /dose Group 1	3 mg/kg /dose Group 2	10 mg/kg /dose Group 3	100 mg/kg /dose Group 4	0 mg/kg /dose Group 1	3 mg/kg /dose Group 2	10 mg/kg /dose Group 3	100 mg/kg /dose Group 4
Number of Animals:	3	3	3	3	3	3	3	3
**SITE, INFUSION, CEPHALIC, LEFT (Continued...)**								
.... mild	0	0	1	0	0	0	0	0
**SITE, INFUSION, CEPHALIC, RIGHT**								
Examined	3	3	3	3	3	3	3	3
No Visible Lesions	3	2	3	3	3	2	3	3
Infiltration, mixed cell; subcutaneous tissue	0	1	0	0	0	1	0	0
.... minimal	0	1	0	0	0	1	0	0
**SKIN**								
Examined	3	3	3	3	3	3	3	3
No Visible Lesions	3	3	3	3	3	3	3	3
**SMALL INTESTINE, DUODENUM**								
Examined	3	3	3	3	3	3	3	3
No Visible Lesions	3	3	3	3	3	3	3	3
**SMALL INTESTINE, ILEUM**								
Examined	3	3	3	3	3	3	3	3
No Visible Lesions	3	3	3	3	3	3	3	3
**SMALL INTESTINE, JEJUNUM**								
Examined	3	3	3	3	3	3	3	3
No Visible Lesions	3	3	3	3	3	3	3	3
**SPINAL CORD**								
Examined	3	3	3	3	3	3	3	3
No Visible Lesions	3	3	3	3	3	3	3	3
**SPLEEN**								
Examined	3	3	3	3	3	3	3	3
No Visible Lesions	3	3	3	3	2	3	3	3
Inflammation, mixed cell	0	0	0	0	1	0	0	0
.... mild	0	0	0	0	1	0	0	0
**STOMACH**								
Examined	3	3	3	3	3	3	3	3
No Visible Lesions	2	3	3	3	3	3	3	3
Hyperplasia/hyperkeratosis	1	0	0	0	0	0	0	0
.... mild	1	0	0	0	0	0	0	0

Appendix 21

Summary of Microscopic Pathology: Terminal Euthanasia - Day 31

Removal Reason(s): TERMINAL EUTHANASIA<br>Summary: Incidence	Male				Female			
	0 mg/kg /dose Group 1	3 mg/kg /dose Group 2	10 mg/kg /dose Group 3	100 mg/kg /dose Group 4	0 mg/kg /dose Group 1	3 mg/kg /dose Group 2	10 mg/kg /dose Group 3	100 mg/kg /dose Group 4
Number of Animals:	3	3	3	3	3	3	3	3
**STOMACH (Continued...)**								
Metaplasia, squamous; fundus	1	0	0	0	0	0	0	0
.... moderate	1	0	0	0	0	0	0	0
Infiltration, lymphocytic; mucosal	1	0	0	0	0	0	0	0
.... moderate	1	0	0	0	0	0	0	0
**TESTIS**								
Examined	3	3	3	3	.	.	.	.
No Visible Lesions	3	3	3	3	.	.	.	.
**THYMUS**								
Examined	3	3	3	3	3	3	3	3
No Visible Lesions	2	3	2	3	3	3	3	3
Cellularity, decreased; cortical	1	0	1	0	0	0	0	0
.... minimal	0	0	1	0	0	0	0	0
.... mild	1	0	0	0	0	0	0	0
**TONGUE**								
Examined	3	3	3	3	3	3	3	3
No Visible Lesions	3	3	2	3	3	3	2	3
Infiltration, mononuclear cell	0	0	0	0	0	0	1	0
.... minimal	0	0	0	0	0	0	1	0
Infiltration, mixed cell; submucosal	0	0	1	0	0	0	0	0
.... mild	0	0	1	0	0	0	0	0
**TRACHEA**								
Examined	3	3	3	3	3	3	3	3
No Visible Lesions	3	3	3	3	3	3	3	3
**URINARY BLADDER**								
Examined	3	3	3	3	3	2	3	3
No Visible Lesions	3	3	3	3	2	2	3	3
Not Examined: Insufficient Tissue Available For Evaluation.	.	.	.	.	0	1	0	0
Infiltration, mononuclear cell; submucosal	0	0	0	0	1	0	0	0
.... minimal	0	0	0	0	1	0	0	0
**UTERUS**								
Examined	.	.	.	.	3	3	3	3
No Visible Lesions	.	.	.	.	3	3	3	3

Appendix 21

Summary of Microscopic Pathology: Terminal Euthanasia - Day 31

Removal Reason(s): TERMINAL EUTHANASIA<br>Summary: Incidence	Male				Female			
	0 mg/kg /dose Group 1	3 mg/kg /dose Group 2	10 mg/kg /dose Group 3	100 mg/kg /dose Group 4	0 mg/kg /dose Group 1	3 mg/kg /dose Group 2	10 mg/kg /dose Group 3	100 mg/kg /dose Group 4
Number of Animals:	3	3	3	3	3	3	3	3
**VAGINA**								
Examined	.	.	.	.	3	3	3	3
No Visible Lesions	.	.	.	.	3	3	3	3
**GLAND, SALIVARY, SUBMANDIBULAR**								
Examined	3	3	3	3	3	3	3	3
No Visible Lesions	3	3	3	3	3	2	3	3
Infiltration, lymphocytic	0	0	0	0	0	1	0	0
.... moderate	0	0	0	0	0	1	0	0

Appendix 21

Summary of Microscopic Pathology: Recovery Euthanasia - Day 71

Removal Reason(s): RECOVERY EUTHANASIA<br>Summary: Incidence	Male		Female	
	0 mg/kg /dose Group 1	100 mg/kg /dose Group 4	0 mg/kg /dose Group 1	100 mg/kg /dose Group 4
Number of Animals:	2	2	2	2
**ARTERY, AORTA**				
Examined	2	2	2	2
No Visible Lesions	2	2	2	2
**BONE MARROW, STERNUM**				
Examined	2	2	2	2
No Visible Lesions	2	2	2	2
**BONE, FEMUR**				
Examined	2	2	2	2
No Visible Lesions	2	2	2	2
**BONE, STERNUM**				
Examined	2	2	2	2
No Visible Lesions	2	2	2	2
**BRAIN**				
Examined	2	2	2	2
No Visible Lesions	2	2	2	2
**CERVIX**				
Examined	.	.	2	2
No Visible Lesions	.	.	2	1
Dilatation; lumen	.	.	0	1
.... moderate	.	.	0	1
**EPIDIDYMIS**				
Examined	2	2	.	.
No Visible Lesions	2	2	.	.
**ESOPHAGUS**				
Examined	2	2	2	2
No Visible Lesions	2	2	2	2
**EYE**				
Examined	2	2	2	2
No Visible Lesions	2	2	2	2
**GALLBLADDER**				
Examined	2	2	2	2
No Visible Lesions	2	2	2	2
**GALT**				
Examined	2	2	2	2
No Visible Lesions	2	2	2	2

Appendix 21

Summary of Microscopic Pathology: Recovery Euthanasia - Day 71

Removal Reason(s): RECOVERY EUTHANASIA	Male		Female	
Summary: Incidence	0 mg/kg /dose Group 1	100 mg/kg /dose Group 4	0 mg/kg /dose Group 1	100 mg/kg /dose Group 4
Number of Animals:	2	2	2	2
**GLAND, ADRENAL**				
Examined	2	2	2	2
No Visible Lesions	1	2	2	2
Mineralization	1	0	0	0
.... minimal	1	0	0	0
**GLAND, MAMMARY**				
Examined	2	2	2	2
No Visible Lesions	2	2	2	2
**GLAND, PARATHYROID**				
Examined	1	2	1	1
No Visible Lesions	1	2	1	1
Not Examined: Not Present In Section.	1	0	1	1
**GLAND, PITUITARY**				
Examined	2	2	2	2
No Visible Lesions	2	2	2	1
Cyst; pars distalis	0	0	0	1
**GLAND, PROSTATE**				
Examined	2	2	.	.
No Visible Lesions	2	2	.	.
**GLAND, SEMINAL VESICLE**				
Examined	2	2	.	.
No Visible Lesions	2	2	.	.
**GLAND, THYROID**				
Examined	2	2	2	2
No Visible Lesions	2	2	1	1
Cyst	0	0	1	0
Infiltration, mononuclear cell	0	0	0	1
.... minimal	0	0	0	1
**HEART**				
Examined	2	2	2	2
No Visible Lesions	2	2	2	2
**JOINT, FEMOROTIBIAL**				
Examined	2	2	2	2
No Visible Lesions	2	2	2	2

Appendix 21

Summary of Microscopic Pathology: Recovery Euthanasia - Day 71

Removal Reason(s): RECOVERY EUTHANASIA	Male		Female	
Summary: Incidence	0 mg/kg /dose Group 1	100 mg/kg /dose Group 4	0 mg/kg /dose Group 1	100 mg/kg /dose Group 4
Number of Animals:	2	2	2	2
**KIDNEY**				
Examined	2	2	2	2
No Visible Lesions	2	2	1	2
Infiltration, mononuclear cell	0	0	1	0
.... minimal	0	0	1	0
**LARGE INTESTINE, CECUM**				
Examined	2	2	2	2
No Visible Lesions	2	2	2	2
**LARGE INTESTINE, COLON**				
Examined	2	2	2	2
No Visible Lesions	2	2	2	2
**LARGE INTESTINE, RECTUM**				
Examined	2	2	2	2
No Visible Lesions	2	2	2	2
**LIVER**				
Examined	2	2	2	2
No Visible Lesions	1	1	2	2
Vacuolation; hepatocellular	1	0	0	0
.... mild	1	0	0	0
Infiltration, mononuclear cell	1	1	0	0
.... minimal	1	1	0	0
**LUNG**				
Examined	2	2	2	2
No Visible Lesions	2	2	2	2
**LYMPH NODE, AXILLARY**				
Examined	2	2	2	2
No Visible Lesions	2	2	2	2
**LYMPH NODE, MANDIBULAR**				
Examined	2	2	2	2
No Visible Lesions	2	2	2	2
**LYMPH NODE, MESENTERIC**				
Examined	2	2	2	2
No Visible Lesions	2	2	2	2
**MUSCLE, QUADRICEPS**				
Examined	2	2	2	2

Appendix 21

Summary of Microscopic Pathology: Recovery Euthanasia - Day 71

Removal Reason(s): RECOVERY EUTHANASIA	Male		Female	
Summary: Incidence	0 mg/kg /dose Group 1	100 mg/kg /dose Group 4	0 mg/kg /dose Group 1	100 mg/kg /dose Group 4
Number of Animals:	2	2	2	2
**MUSCLE, QUADRICEPS (Continued...)**				
No Visible Lesions	2	2	2	2
**NERVE, OPTIC**				
Examined	2	2	2	2
No Visible Lesions	2	2	2	2
**NERVE, SCIATIC**				
Examined	2	2	2	2
No Visible Lesions	2	2	2	2
**OVARY**				
Examined	.	.	2	2
No Visible Lesions	.	.	2	1
Hypertrophy; corpus luteum	.	.	0	1
.... moderate	.	.	0	1
**PANCREAS**				
Examined	2	2	2	2
No Visible Lesions	1	2	2	2
Infiltration, mononuclear cell	1	0	0	0
.... mild	1	0	0	0
**SITE, INFUSION, CEPHALIC, LEFT**				
Examined	2	2	2	2
No Visible Lesions	2	2	2	2
**SITE, INFUSION, CEPHALIC, RIGHT**				
Examined	2	2	2	2
No Visible Lesions	2	2	2	2
**SKIN**				
Examined	2	2	2	2
No Visible Lesions	2	2	2	2
**SMALL INTESTINE, DUODENUM**				
Examined	2	2	2	2
No Visible Lesions	2	2	2	2
**SMALL INTESTINE, ILEUM**				
Examined	2	2	2	2
No Visible Lesions	2	2	2	2
**SMALL INTESTINE, JEJUNUM**				
Examined	2	2	2	2

Appendix 21

Summary of Microscopic Pathology: Recovery Euthanasia - Day 71

Removal Reason(s): RECOVERY EUTHANASIA<br>Summary: Incidence	Male		Female	
	0 mg/kg /dose Group 1	100 mg/kg /dose Group 4	0 mg/kg /dose Group 1	100 mg/kg /dose Group 4
Number of Animals:	2	2	2	2
**SMALL INTESTINE, JEJUNUM (Continued...)**				
No Visible Lesions	2	2	2	2
**SPINAL CORD**				
Examined	2	2	2	2
No Visible Lesions	1	2	2	2
Mineralization	1	0	0	0
.... moderate	1	0	0	0
**SPLEEN**				
Examined	2	2	2	2
No Visible Lesions	2	2	2	2
**STOMACH**				
Examined	2	2	2	2
No Visible Lesions	2	2	1	2
Granuloma; submucosal	0	0	1	0
.... mild	0	0	1	0
**TESTIS**				
Examined	2	2	.	.
No Visible Lesions	2	2	.	.
**THYMUS**				
Examined	2	2	2	2
No Visible Lesions	2	2	2	2
**TONGUE**				
Examined	2	2	2	2
No Visible Lesions	2	2	2	2
**TRACHEA**				
Examined	2	2	2	2
No Visible Lesions	2	2	2	2
**URINARY BLADDER**				
Examined	1	2	2	2
No Visible Lesions	1	2	2	2
Not Examined: Not Present In Section.	1	0	.	.
**UTERUS**				
Examined	.	.	2	2
No Visible Lesions	.	.	2	2

Appendix 21

Summary of Microscopic Pathology: Recovery Euthanasia - Day 71

Removal Reason(s): RECOVERY EUTHANASIA	Male		Female	
Summary: Incidence	0 mg/kg /dose Group 1	100 mg/kg /dose Group 4	0 mg/kg /dose Group 1	100 mg/kg /dose Group 4
Number of Animals:	2	2	2	2
**VAGINA**				
Examined	.	.	2	2
No Visible Lesions	.	.	2	2
**GLAND, SALIVARY, SUBMANDIBULAR**				
Examined	2	2	2	2
No Visible Lesions	2	1	2	1
Infiltration, mononuclear cell	0	1	0	1
.... minimal	0	0	0	1
.... mild	0	1	0	0

Appendix 21

TABLES EXPLANATION PAGE

All Day(s) referenced throughout the outputs generated are Study Days beginning with Day 1, the first day of dosing

Abbreviations consistent throughout the Summary and Individual Tables

Note: All of the abbreviations listed on these pages may not be applicable to this report

Abbreviation	Description
mg/kg/dose	milligrams/kilograms/dose
mg/kg	milligrams/kilograms
ppm	parts per million
RC	result comment
% Diff	% Difference from Group 1
1F, 2F, 3F, 4F	Group 1 Female, Group 2 Female, Group 3 Female, Group 4 Female
1M, 2M, 3M, 4M	Group 1 Male, Group 2 Male, Group 3 Male, Group 4 Male
g	grams
kg	kilograms
mg	milligrams
N	Number of values included in analysis
M, F	Male, Female
<, >	Out of range
--	Not scheduled to be performed/dead

Group 1 - 0 mg/kg/dose
Group 2 - 3 mg/kg/dose
Group 3 - 10 mg/kg/dose
Group 4 - 100 mg/kg/dose

CAUSE OF DEATH

Abbreviation	Description	Abbreviation	Description
ACCD	Accidental death	TERM	Terminal euthanasia
FD	Found dead	TGL	Trackable gross lesion
M	Mass	UNSC	Unscheduled euthanasia
MSAC	Moribund sacrifice		

Appendix 21

MACROSCOPIC AND MICROSCOPIC FINDINGS

Abbreviation	Description	Abbreviation	Description
ACCD	Accidental death	MPF	Major pathological finding
C	Clinical observation	MSAC	Moribund sacrifice
E	Excluded	ONP	Organ not present
FD	Found dead	OPOP	Only one of the paired organs present
G	Gross pathology	OUM	Organ unidentifiable macroscopically
H	Histo pathology	TERM	Terminal euthanasia
LIBW	Lung infused before weighing	TGL	Trackable gross lesion
M	Mass	UNSC	Unscheduled euthanasia
mL	milliliters		

ORGAN WEIGHTS

Abbreviation	Description	Abbreviation	Description
CG	Coagulating gland	OUM	Organ unidentifiable macroscopically
Ep	Epididymis	PI	Macroscopic pathology - Included in mean
LIBW	Lung infused before weighing	Sem Ves	Seminal vesicle
Liver wt igb	Liver weight intact gallbladder	X	Excluded from calculation of the mean
MPE	Macroscopic pathology - exclude from mean	OP	Only one pair organ present - excluded from calculations
MPI	Macroscopic pathology - include in mean	OPMP	Only one of the paired organs present - macroscopic pathology
ONP	Organ not present	OPOP	Only one of the paired organs present
PARATHY	Parathyroid	TBR	Terminal Bodyweight Ratio
BR	Brain Ratio	WT	Weight
BW	Body Weight	GLD	Gland
%Diff G1	Percent Difference from Group 1	NT	Not taken
FC	Flag comment	%br	% Organ Weight/Brain Weight
%bw	% Organ Weight/Body Weight		

Appendix 21

Individual Organ Weights: Terminal Euthanasia - Day 31

Sex: Male   Day(s) Relative to Start Date

0 mg/kg /dose Group 1	Organ Weights (BW kg)					
	Terminal Body Wt (Kg)	Brain (g)	Brain (%bw)	Epididymis (g)	Epididymis (%br)	Epididymis (%bw)
	-	-	-	-	-	-
1001	2.4	66.840	2.7850	0.500	0.7481	0.0208
1002	4.3	67.590	1.5719	2.440	3.6100	0.0567
1003	3.0	64.890	2.1630	0.580	0.8938	0.0193
Mean	3.23	66.4400	2.17329	1.1733	1.75063	0.03230
SD	0.97	1.3937	0.60664	1.0977	1.61192	0.02118
N	3	3	3	3	3	3

Appendix 21

Individual Organ Weights: Terminal Euthanasia - Day 31

Sex: Male Day(s) Relative to Start Date

0 mg/kg /dose Group 1	Organ Weights (BW kg)					
	Gland Adrenal (g)	Gland Adrenal (%br)	Gland Adrenal (%bw)	Gland Pituitary (g)	Gland Pituitary (%br)	Gland Pituitary (%bw)
	-	-	-	-	-	-
1001	0.490	0.7331	0.0204	0.040	0.0598	0.0017
1002	0.720	1.0652	0.0167	0.070	0.1036	0.0016
1003	0.480	0.7397	0.0160	0.050	0.0771	0.0017
Mean	0.5633	0.84602	0.01772	0.0533	0.08015	0.00165
SD	0.1358	0.18989	0.00236	0.0153	0.02202	0.00002
N	3	3	3	3	3	3

Appendix 21

Individual Organ Weights: Terminal Euthanasia - Day 31

Sex: Male   Day(s) Relative to Start Date

0 mg/kg /dose Group 1	Organ Weights (BW kg)					
	Prostate/ Seminal Ves (g)	Prostate Seminal Ves (%br)	Prostate Seminal Ves (%bw)	Thyroid/ Parathyroid (g)	Thyroid/ Parathyroid (%br)	Thyroid/ Parathyroid (%bw)
	-	-	-	-	-	-
1001	1.290	1.9300	0.0538	0.230	0.3441	0.0096
1002	9.710	14.3660	0.2258	0.380	0.5622	0.0088
1003	1.090	1.6798	0.0363	0.210	0.3236	0.0070
Mean	4.0300	5.99193	0.10530	0.2733	0.40998	0.00847
SD	4.9200	7.25327	0.10473	0.0929	0.13223	0.00133
N	3	3	3	3	3	3

Appendix 21

Individual Organ Weights: Terminal Euthanasia - Day 31

Sex: Male Day(s) Relative to Start Date

0 mg/kg /dose Group 1	Organ Weights (BW kg)					
	Heart (g)	Heart (%br)	Heart (%bw)	Kidney (g)	Kidney (%br)	Kidney (%bw)
	-	-	-	-	-	-
1001	9.540	14.2729	0.3975	11.640	17.4147	0.4850
1002	20.830	30.8182	0.4844	19.390	28.6877	0.4509
1003	12.200	18.8010	0.4067	15.840	24.4105	0.5280
Mean	14.1900	21.29737	0.42953	15.6233	23.50431	0.48798
SD	5.9022	8.55045	0.04776	3.8795	5.69085	0.03862
N	3	3	3	3	3	3

Appendix 21

Individual Organ Weights: Terminal Euthanasia - Day 31

Sex: Male Day(s) Relative to Start Date

0 mg/kg /dose Group 1	Organ Weights (BW kg)					
	Liver/ Gallbladder (g)	Liver/ Gallbladder (%br)	Liver/ Gallbladder (%bw)	Spleen (g)	Spleen (%br)	Spleen (%bw)
	-	-	-	-	-	-
1001	48.490	72.5464	2.0204	5.380	8.0491	0.2242
1002	84.750	125.3884	1.9709	8.210	12.1468	0.1909
1003	63.760	98.2586	2.1253	6.140	9.4622	0.2047
Mean	65.6667	98.73111	2.03889	6.5767	9.88600	0.20659
SD	18.2050	26.42416	0.07884	1.4647	2.08147	0.01670
N	3	3	3	3	3	3

Appendix 21

Individual Organ Weights: Terminal Euthanasia - Day 31

Sex: Male   Day(s) Relative to Start Date

0 mg/kg /dose Group 1	Organ Weights (BW kg)					
	Testis (g)	Testis (%br)	Testis (%bw)	Thymus (g)	Thymus (%br)	Thymus (%bw)
	-	-	-	-	-	-
1001	1.270	1.9001	0.0529	2.350	3.5159	0.0979
1002	17.370	25.6991	0.4040	1.880	2.7815	0.0437
1003	1.590	2.4503	0.0530	4.190	6.4571	0.1397
Mean	6.7433	10.01648	0.16996	2.8067	4.25147	0.09377
SD	9.2044	13.58431	0.20265	1.2208	1.94509	0.04811
N	3	3	3	3	3	3

Appendix 21

Individual Organ Weights: Terminal Euthanasia - Day 31

Sex: Male Day(s) Relative to Start Date

3 mg/kg /dose Group 2	Organ Weights (BW kg)					
	Terminal Body Wt (Kg)	Brain (g)	Brain (%bw)	Epididymis (g)	Epididymis (%br)	Epididymis (%bw)
	-	-	-	-	-	-
2001	3.4	63.190	1.8585	0.680	1.0761	0.0200
2002	2.7	71.300	2.6407	0.460	0.6452	0.0170
2003	2.9	68.950	2.3776	1.080	1.5664	0.0372
Mean	3.00	67.8133	2.29229	0.7400	1.09588	0.02476
SD	0.36	4.1728	0.39802	0.3143	0.46091	0.01091
N	3	3	3	3	3	3
%Diff	-7.22	2.0670	5.47551	-36.9318	-37.40079	-23.35387

Appendix 21

Individual Organ Weights: Terminal Euthanasia - Day 31

Sex: Male Day(s) Relative to Start Date

3 mg/kg /dose Group 2	Organ Weights (BW kg)					
	Gland Adrenal (g)	Gland Adrenal (%br)	Gland Adrenal (%bw)	Gland Pituitary (g)	Gland Pituitary (%br)	Gland Pituitary (%bw)
	-	-	-	-	-	-
2001	0.550	0.8704	0.0162	0.040	0.0633	0.0012
2002	0.630	0.8836	0.0233	0.010	0.0140	0.0004
2003	0.680	0.9862	0.0234	0.050	0.0725	0.0017
Mean	0.6200	0.91340	0.02099	0.0333	0.04995	0.00109
SD	0.0656	0.06341	0.00417	0.0208	0.03145	0.00068
N	3	3	3	3	3	3
%Diff	10.0592	7.96475	18.42940	-37.5000	-37.68588	-34.06933

Appendix 21

Individual Organ Weights: Terminal Euthanasia - Day 31

Sex: Male   Day(s) Relative to Start Date

3 mg/kg /dose Group 2	Organ Weights (BW kg)					
	Prostate/ Seminal Ves (g)	Prostate Seminal Ves (%br)	Prostate Seminal Ves (%bw)	Thyroid/ Parathyroid (g)	Thyroid/ Parathyroid (%br)	Thyroid/ Parathyroid (%bw)
	-	-	-	-	-	-
2001	0.960	1.5192	0.0282	0.290	0.4589	0.0085
2002	1.030	1.4446	0.0381	0.190	0.2665	0.0070
2003	3.100	4.4960	0.1069	0.190	0.2756	0.0066
Mean	1.6967	2.48661	0.05776	0.2233	0.33366	0.00737
SD	1.2158	1.74059	0.04284	0.0577	0.10859	0.00103
N	3	3	3	3	3	3
%Diff	-57.8991	-58.50060	-45.14673	-18.2927	-18.61616	-12.99095

Appendix 21

Individual Organ Weights: Terminal Euthanasia - Day 31

Sex: Male Day(s) Relative to Start Date

3 mg/kg /dose Group 2	Organ Weights (BW kg)					
	Heart (g)	Heart (%br)	Heart (%bw)	Kidney (g)	Kidney (%br)	Kidney (%bw)
	-	-	-	-	-	-
2001	12.500	19.7816	0.3676	14.780	23.3898	0.4347
2002	10.400	14.5863	0.3852	13.990	19.6213	0.5181
2003	12.080	17.5199	0.4166	13.320	19.3183	0.4593
Mean	11.6600	17.29594	0.38979	14.0300	20.77648	0.47072
SD	1.1112	2.60491	0.02478	0.7308	2.26825	0.04288
N	3	3	3	3	3	3
%Diff	-17.8295	-18.78839	-9.25056	-10.1984	-11.60567	-3.53609

Appendix 21

Individual Organ Weights: Terminal Euthanasia - Day 31

Sex: Male Day(s) Relative to Start Date

3 mg/kg /dose Group 2	Organ Weights (BW kg)					
	Liver/ Gallbladder (g)	Liver/ Gallbladder (%br)	Liver/ Gallbladder (%bw)	Spleen (g)	Spleen (%br)	Spleen (%bw)
	-	-	-	-	-	-
2001	67.220	106.3776	1.9771	6.550	10.3656	0.1926
2002	58.930	82.6508	2.1826	6.520	9.1445	0.2415
2003	54.080	78.4336	1.8648	5.930	8.6004	0.2045
Mean	60.0767	89.15400	2.00816	6.3333	9.37015	0.21287
SD	6.6446	15.06436	0.16115	0.3496	0.90395	0.02547
N	3	3	3	3	3	3
%Diff	-8.5127	-9.70019	-1.50737	-3.6999	-5.21797	3.04112

Appendix 21

Individual Organ Weights: Terminal Euthanasia - Day 31

Sex: Male Day(s) Relative to Start Date

3 mg/kg /dose Group 2	Organ Weights (BW kg)					
	Testis (g)	Testis (%br)	Testis (%bw)	Thymus (g)	Thymus (%br)	Thymus (%bw)
	-	-	-	-	-	-
2001	1.590	2.5162	0.0468	2.820	4.4627	0.0829
2002	0.910	1.2763	0.0337	1.810	2.5386	0.0670
2003	1.330	1.9289	0.0459	2.490	3.6113	0.0859
Mean	1.2767	1.90715	0.04211	2.3733	3.53754	0.07861
SD	0.3431	0.62025	0.00729	0.5150	0.96420	0.01013
N	3	3	3	3	3	3
%Diff	-81.0677	-80.95986	-75.22301	-15.4394	-16.79264	-16.16185

Appendix 21

Individual Organ Weights: Terminal Euthanasia - Day 31

Sex: Male Day(s) Relative to Start Date

10 mg/kg /dose Group 3	Organ Weights (BW kg)					
	Terminal Body Wt (Kg)	Brain (g)	Brain (%bw)	Epididymis (g)	Epididymis (%br)	Epididymis (%bw)
	-	-	-	-	-	-
3001	3.0	73.850	2.4617	0.850	1.1510	0.0283
3002	2.9	69.410	2.3934	1.050	1.5128	0.0362
3003	3.1	66.780	2.1542	0.730	1.0931	0.0235
Mean	3.00	70.0133	2.33644	0.8767	1.25229	0.02936
SD	0.10	3.5734	0.16147	0.1617	0.22741	0.00639
N	3	3	3	3	3	3
%Diff	-7.22	5.3783	7.50703	-25.2841	-28.46607	-9.10345

Appendix 21

Individual Organ Weights: Terminal Euthanasia - Day 31

Sex: Male Day(s) Relative to Start Date

10 mg/kg /dose Group 3	Organ Weights (BW kg)					
	Gland Adrenal (g)	Gland Adrenal (%br)	Gland Adrenal (%bw)	Gland Pituitary (g)	Gland Pituitary (%br)	Gland Pituitary (%bw)
	-	-	-	-	-	-
3001	0.520	0.7041	0.0173	0.040	0.0542	0.0013
3002	0.450	0.6483	0.0155	0.050	0.0720	0.0017
3003	0.550	0.8236	0.0177	0.060	0.0898	0.0019
Mean	0.5067	0.72535	0.01686	0.0500	0.07202	0.00166
SD	0.0513	0.08955	0.00118	0.0100	0.01784	0.00031
N	3	3	3	3	3	3
%Diff	-10.0592	-14.26299	-4.83127	-6.2500	-10.15400	0.63925

Appendix 21

Individual Organ Weights: Terminal Euthanasia - Day 31

Sex: Male Day(s) Relative to Start Date

10 mg/kg /dose Group 3	Organ Weights (BW kg)					
	Prostate/ Seminal Ves (g)	Prostate Seminal Ves (%br)	Prostate Seminal Ves (%bw)	Thyroid/ Parathyroid (g)	Thyroid/ Parathyroid (%br)	Thyroid/ Parathyroid (%bw)
	-	-	-	-	-	-
3001	1.310	1.7739	0.0437	0.240	0.3250	0.0080
3002	2.590	3.7315	0.0893	0.270	0.3890	0.0093
3003	1.110	1.6622	0.0358	0.290	0.4343	0.0094
Mean	1.6700	2.38916	0.05626	0.2667	0.38275	0.00889
SD	0.8030	1.16380	0.02889	0.0252	0.05491	0.00077
N	3	3	3	3	3	3
%Diff	-58.5608	-60.12695	-46.57014	-2.4390	-6.64303	4.89620

Appendix 21

Individual Organ Weights: Terminal Euthanasia - Day 31

Sex: Male Day(s) Relative to Start Date

10 mg/kg /dose Group 3	Organ Weights (BW kg)					
	Heart (g)	Heart (%br)	Heart (%bw)	Kidney (g)	Kidney (%br)	Kidney (%bw)
	-	-	-	-	-	-
3001	15.310	20.7312	0.5103	14.500	19.6344	0.4833
3002	11.380	16.3953	0.3924	15.440	22.2446	0.5324
3003	14.530	21.7580	0.4687	15.710	23.5250	0.5068
Mean	13.7400	19.62819	0.45715	15.2167	21.80134	0.50751
SD	2.0807	2.84642	0.05980	0.6352	1.98283	0.02455
N	3	3	3	3	3	3
%Diff	-3.1712	-7.83751	6.43120	-2.6029	-7.24534	4.00231

Appendix 21

Individual Organ Weights: Terminal Euthanasia - Day 31

Sex: Male Day(s) Relative to Start Date

10 mg/kg /dose Group 3	Organ Weights (BW kg)					
	Liver/ Gallbladder (g)	Liver/ Gallbladder (%br)	Liver/ Gallbladder (%bw)	Spleen (g)	Spleen (%br)	Spleen (%bw)
	-	-	-	-	-	-
3001	62.510	84.6445	2.0837	5.610	7.5965	0.1870
3002	62.340	89.8141	2.1497	7.330	10.5604	0.2528
3003	57.760	86.4930	1.8632	5.740	8.5954	0.1852
Mean	60.8700	86.98389	2.03218	6.2267	8.91744	0.20831
SD	2.6947	2.61953	0.14999	0.9577	1.50799	0.03851
N	3	3	3	3	3	3
%Diff	-7.3046	-11.89820	-0.32914	-5.3218	-9.79736	0.83199

Appendix 21

Individual Organ Weights: Terminal Euthanasia - Day 31

Sex: Male Day(s) Relative to Start Date

10 mg/kg /dose Group 3	Organ Weights (BW kg)					
	Testis (g)	Testis (%br)	Testis (%bw)	Thymus (g)	Thymus (%br)	Thymus (%bw)
	-	-	-	-	-	-
3001	1.230	1.6655	0.0410	3.270	4.4279	0.1090
3002	2.410	3.4721	0.0831	1.630	2.3484	0.0562
3003	1.540	2.3061	0.0497	2.570	3.8485	0.0829
Mean	1.7267	2.48125	0.05793	2.4900	3.54157	0.08270
SD	0.6117	0.91594	0.02223	0.8229	1.07319	0.02640
N	3	3	3	3	3	3
%Diff	-74.3945	-75.22835	-65.91664	-11.2827	-16.69774	-11.80008

Appendix 21

Individual Organ Weights: Terminal Euthanasia - Day 31

Sex: Male Day(s) Relative to Start Date

100 mg/kg /dose Group 4	Organ Weights (BW kg)					
	Terminal Body Wt (Kg)	Brain (g)	Brain (%bw)	Epididymis (g)	Epididymis (%br)	Epididymis (%bw)
	-	-	-	-	-	-
4001	3.0	63.480	2.1160	0.720	1.1342	0.0240
4002	3.2	64.030	2.0009	1.030	1.6086	0.0322
4003	2.5	72.050	2.8820	0.700	0.9715	0.0280
Mean	2.90	66.5200	2.33298	0.8167	1.23813	0.02806
SD	0.36	4.7970	0.47893	0.1850	0.33100	0.00409
N	3	3	3	3	3	3
%Diff	-10.31	0.1204	7.34796	-30.3977	-29.27511	-13.12892

Appendix 21

Individual Organ Weights: Terminal Euthanasia - Day 31

Sex: Male   Day(s) Relative to Start Date

100 mg/kg /dose Group 4	Organ Weights (BW kg)					
	Gland Adrenal (g)	Gland Adrenal (%br)	Gland Adrenal (%bw)	Gland Pituitary (g)	Gland Pituitary (%br)	Gland Pituitary (%bw)
	-	-	-	-	-	-
4001	0.580	0.9137	0.0193	0.040	0.0630	0.0013
4002	0.790	1.2338	0.0247	0.050	0.0781	0.0016
4003	0.500	0.6940	0.0200	0.040	0.0555	0.0016
Mean	0.6233	0.94714	0.02134	0.0433	0.06554	0.00150
SD	0.1498	0.27147	0.00292	0.0058	0.01150	0.00014
N	3	3	3	3	3	3
%Diff	10.6509	11.95322	20.42853	-18.7500	-18.23400	-9.38086

Appendix 21

Individual Organ Weights: Terminal Euthanasia - Day 31

Sex: Male Day(s) Relative to Start Date

100 mg/kg /dose Group 4	Organ Weights (BW kg)					
	Prostate/ Seminal Ves (g)	Prostate Seminal Ves (%br)	Prostate Seminal Ves (%bw)	Thyroid/ Parathyroid (g)	Thyroid/ Parathyroid (%br)	Thyroid/ Parathyroid (%bw)
	-	-	-	-	-	-
4001	1.650	2.5992	0.0550	0.260	0.4096	0.0087
4002	2.530	3.9513	0.0791	0.190	0.2967	0.0059
4003	1.240	1.7210	0.0496	0.250	0.3470	0.0100
Mean	1.8067	2.75718	0.06122	0.2333	0.35110	0.00820
SD	0.6591	1.12348	0.01569	0.0379	0.05653	0.00207
N	3	3	3	3	3	3
%Diff	-55.1696	-53.98506	-41.86006	-14.6341	-14.36231	-3.21148

Appendix 21

Individual Organ Weights: Terminal Euthanasia - Day 31

Sex: Male Day(s) Relative to Start Date

100 mg/kg /dose Group 4	Organ Weights (BW kg)					
	Heart (g)	Heart (%br)	Heart (%bw)	Kidney (g)	Kidney (%br)	Kidney (%bw)
	-	-	-	-	-	-
4001	12.680	19.9748	0.4227	15.270	24.0548	0.5090
4002	13.020	20.3342	0.4069	16.880	26.3626	0.5275
4003	9.900	13.7405	0.3960	9.290	12.8938	0.3716
Mean	11.8667	18.01649	0.40851	13.8133	21.10376	0.46937
SD	1.7116	3.70751	0.01341	3.9992	7.20304	0.08517
N	3	3	3	3	3	3
%Diff	-16.3730	-15.40509	-4.89247	-11.5852	-10.21323	-3.81372

Appendix 21

Individual Organ Weights: Terminal Euthanasia - Day 31

Sex: Male Day(s) Relative to Start Date

100 mg/kg /dose Group 4	Organ Weights (BW kg)					
	Liver/ Gallbladder (g)	Liver/ Gallbladder (%br)	Liver/ Gallbladder (%bw)	Spleen (g)	Spleen (%br)	Spleen (%bw)
	-	-	-	-	-	-
4001	60.550	95.3844	2.0183	6.180	9.7353	0.2060
4002	73.490	114.7743	2.2966	7.600	11.8694	0.2375
4003	49.680	68.9521	1.9872	5.850	8.1194	0.2340
Mean	61.2400	93.03694	2.10070	6.5433	9.90805	0.22583
SD	11.9200	23.00112	0.17034	0.9299	1.88099	0.01727
N	3	3	3	3	3	3
%Diff	-6.7411	-5.76736	3.03131	-0.5068	0.22301	9.31588

Appendix 21

Individual Organ Weights: Terminal Euthanasia - Day 31

Sex: Male Day(s) Relative to Start Date

100 mg/kg /dose Group 4	Organ Weights (BW kg)					
	Testis (g)	Testis (%br)	Testis (%bw)	Thymus (g)	Thymus (%br)	Thymus (%bw)
	-	-	-	-	-	-
4001	1.230	1.9376	0.0410	3.930	6.1909	0.1310
4002	1.780	2.7799	0.0556	4.030	6.2939	0.1259
4003	1.070	1.4851	0.0428	2.040	2.8314	0.0816
Mean	1.3600	2.06755	0.04648	3.3333	5.10541	0.11285
SD	0.3724	0.65714	0.00798	1.1212	1.97005	0.02718
N	3	3	3	3	3	3
%Diff	-79.8319	-79.35853	-72.65480	18.7648	20.08560	20.34567

Appendix 21

Individual Organ Weights: Terminal Euthanasia - Day 31

Sex: Female Day(s) Relative to Start Date

0 mg/kg /dose Group 1	Organ Weights (BW kg)					
	Terminal Body Wt (Kg)	Brain (g)	Brain (%bw)	Gland Adrenal (g)	Gland Adrenal (%br)	Gland Adrenal (%bw)
	-	-	-	-	-	-
1501	3.0	68.940	2.2980	0.540	0.7833	0.0180
1502	3.3	72.230	2.1888	0.760	1.0522	0.0230
1503	2.6	63.980	2.4608	0.510	0.7971	0.0196
Mean	2.97	68.3833	2.31585	0.6033	0.87754	0.02022
SD	0.35	4.1531	0.13687	0.1365	0.15142	0.00257
N	3	3	3	3	3	3

Appendix 21

Individual Organ Weights: Terminal Euthanasia - Day 31

Sex: Female Day(s) Relative to Start Date

0 mg/kg /dose Group 1	Organ Weights (BW kg)					
	Gland Pituitary (g)	Gland Pituitary (%br)	Gland Pituitary (%bw)	Thyroid/ Parathyroid (g)	Thyroid/ Parathyroid (%br)	Thyroid/ Parathyroid (%bw)
	-	-	-	-	-	-
1501	0.050	0.0725	0.0017	0.240	0.3481	0.0080
1502	0.070	0.0969	0.0021	0.540	0.7476	0.0164
1503	0.050	0.0781	0.0019	0.270	0.4220	0.0104
Mean	0.0567	0.08253	0.00190	0.3500	0.50592	0.01158
SD	0.0115	0.01277	0.00023	0.1652	0.21255	0.00431
N	3	3	3	3	3	3

Appendix 21

Individual Organ Weights: Terminal Euthanasia - Day 31

Sex: Female Day(s) Relative to Start Date

0 mg/kg /dose Group 1	Organ Weights (BW kg)					
	Heart (g)	Heart (%br)	Heart (%bw)	Kidney (g)	Kidney (%br)	Kidney (%bw)
	-	-	-	-	-	-
1501	11.330	16.4346	0.3777	13.390	19.4227	0.4463
1502	12.720	17.6104	0.3855	15.270	21.1408	0.4627
1503	9.620	15.0359	0.3700	12.340	19.2873	0.4746
Mean	11.2233	16.36031	0.37771	13.6667	19.95025	0.46123
SD	1.5528	1.28884	0.00773	1.4845	1.03326	0.01420
N	3	3	3	3	3	3

Appendix 21

Individual Organ Weights: Terminal Euthanasia - Day 31

Sex: Female   Day(s) Relative to Start Date

0 mg/kg /dose Group 1	Organ Weights (BW kg)					
	Liver/ Gallbladder (g)	Liver/ Gallbladder (%br)	Liver/ Gallbladder (%bw)	Ovary Paired (g)	Ovary Paired (%br)	Ovary Paired (%bw)
	-	-	-	-	-	-
1501	70.790	102.6835	2.3597	0.210	0.3046	0.0070
1502	76.830	106.3685	2.3282	0.200	0.2769	0.0061
1503	55.420	86.6208	2.1315	0.260	0.4064	0.0100
Mean	67.6800	98.55762	2.27313	0.2233	0.32929	0.00769
SD	11.0386	10.50049	0.12363	0.0321	0.06818	0.00206
N	3	3	3	3	3	3

Appendix 21

Individual Organ Weights: Terminal Euthanasia - Day 31

Sex: Female   Day(s) Relative to Start Date

0 mg/kg /dose Group 1	Organ Weights (BW kg)					
	Spleen (g)	Spleen (%br)	Spleen (%bw)	Thymus (g)	Thymus (%br)	Thymus (%bw)
	-	-	-	-	-	-
1501	6.860	9.9507	0.2287	4.640	6.7305	0.1547
1502	9.890	13.6924	0.2997	5.520	7.6423	0.1673
1503	5.450	8.5183	0.2096	2.610	4.0794	0.1004
Mean	7.4000	10.72045	0.24599	4.2567	6.15071	0.14077
SD	2.2687	2.67155	0.04747	1.4924	1.85083	0.03554
N	3	3	3	3	3	3

Appendix 21

Individual Organ Weights: Terminal Euthanasia - Day 31

Sex: Female Day(s) Relative to Start Date

0 mg/kg /dose Group 1	Organ Weights (BW kg)		
	Uterus/ Cervix (g)	Uterus/ Cervix (%br)	Uterus/ Cervix (%bw)
	-	-	-
1501	5.010	7.2672	0.1670
1502	7.780	10.7711	0.2358
1503	7.240	11.3160	0.2785
Mean	6.6767	9.78479	0.22707
SD	1.4684	2.19726	0.05624
N	3	3	3

Appendix 21

Individual Organ Weights: Terminal Euthanasia - Day 31

Sex: Female   Day(s) Relative to Start Date

3 mg/kg /dose Group 2	Organ Weights (BW kg)					
	Terminal Body Wt (Kg)	Brain (g)	Brain (%bw)	Gland Adrenal (g)	Gland Adrenal (%br)	Gland Adrenal (%bw)
	-	-	-	-	-	-
2501	2.8	61.080	2.1814	0.590	0.9659	0.0211
2502	2.6	58.920	2.2662	0.680	1.1541	0.0262
2503	2.9	56.170	1.9369	0.580	1.0326	0.0200
Mean	2.77	58.7233	2.12816	0.6167	1.05088	0.02241
SD	0.15	2.4609	0.17097	0.0551	0.09541	0.00329
N	3	3	3	3	3	3
%Diff	-6.74	-14.1262	-8.10469	2.2099	19.75322	10.84922

Appendix 21

Individual Organ Weights: Terminal Euthanasia - Day 31

Sex: Female Day(s) Relative to Start Date

3 mg/kg /dose Group 2	Organ Weights (BW kg)					
	Gland Pituitary (g)	Gland Pituitary (%br)	Gland Pituitary (%bw)	Thyroid/ Parathyroid (g)	Thyroid/ Parathyroid (%br)	Thyroid/ Parathyroid (%bw)
	-	-	-	-	-	-
2501	0.050	0.0819	0.0018	0.190	0.3111	0.0068
2502	0.070	0.1188	0.0027	0.220	0.3734	0.0085
2503	0.050	0.0890	0.0017	0.210	0.3739	0.0072
Mean	0.0567	0.09656	0.00207	0.2067	0.35277	0.00750
SD	0.0115	0.01959	0.00054	0.0153	0.03612	0.00087
N	3	3	3	3	3	3
%Diff	0.0000	17.00060	8.60109	-40.9524	-30.27034	-35.28126

Appendix 21

Individual Organ Weights: Terminal Euthanasia - Day 31

Sex: Female   Day(s) Relative to Start Date

3 mg/kg /dose Group 2	Organ Weights (BW kg)					
	Heart (g)	Heart (%br)	Heart (%bw)	Kidney (g)	Kidney (%br)	Kidney (%bw)
	-	-	-	-	-	-
2501	8.950	14.6529	0.3196	11.510	18.8441	0.4111
2502	8.420	14.2906	0.3238	13.260	22.5051	0.5100
2503	11.180	19.9039	0.3855	14.690	26.1528	0.5066
Mean	9.5167	16.28245	0.34300	13.1533	22.50066	0.47587
SD	1.4647	3.14147	0.03688	1.5927	3.65431	0.05615
N	3	3	3	3	3	3
%Diff	-15.2064	-0.47595	-9.18833	-3.7561	12.78383	3.17612

Appendix 21

Individual Organ Weights: Terminal Euthanasia - Day 31

Sex: Female Day(s) Relative to Start Date

3 mg/kg /dose Group 2	Organ Weights (BW kg)					
	Liver/ Gallbladder (g)	Liver/ Gallbladder (%br)	Liver/ Gallbladder (%bw)	Ovary Paired (g)	Ovary Paired (%br)	Ovary Paired (%bw)
	-	-	-	-	-	-
2501	55.610	91.0445	1.9861	0.360	0.5894	0.0129
2502	57.100	96.9111	2.1962	0.220	0.3734	0.0085
2503	61.070	108.7235	2.1059	0.190	0.3383	0.0066
Mean	57.9267	98.89304	2.09603	0.2567	0.43368	0.00929
SD	2.8223	9.00460	0.10539	0.0907	0.13599	0.00323
N	3	3	3	3	3	3
%Diff	-14.4110	0.34033	-7.79102	14.9254	31.69956	20.85721

Appendix 21

Individual Organ Weights: Terminal Euthanasia - Day 31

Sex: Female Day(s) Relative to Start Date

3 mg/kg /dose Group 2	Organ Weights (BW kg)					
	Spleen (g)	Spleen (%br)	Spleen (%bw)	Thymus (g)	Thymus (%br)	Thymus (%bw)
	-	-	-	-	-	-
2501	6.450	10.5599	0.2304	2.180	3.5691	0.0779
2502	5.180	8.7916	0.1992	3.170	5.3802	0.1219
2503	5.530	9.8451	0.1907	3.510	6.2489	0.1210
Mean	5.7200	9.73221	0.20676	2.9533	5.06605	0.10694
SD	0.6560	0.88956	0.02088	0.6910	1.36724	0.02519
N	3	3	3	3	3	3
%Diff	-22.7027	-9.21829	-15.94916	-30.6186	-17.63476	-24.03588

Appendix 21

Individual Organ Weights: Terminal Euthanasia - Day 31

Sex: Female   Day(s) Relative to Start Date

3 mg/kg /dose Group 2	Organ Weights (BW kg)		
	Uterus/ Cervix (g)	Uterus/ Cervix (%br)	Uterus/ Cervix (%bw)
	-	-	-
2501	5.520	9.0373	0.1971
2502	4.960	8.4182	0.1908
2503	3.460	6.1599	0.1193
Mean	4.6467	7.87180	0.16907
SD	1.0651	1.51455	0.04321
N	3	3	3
%Diff	-30.4044	-19.55068	-25.54196

Appendix 21

Individual Organ Weights: Terminal Euthanasia - Day 31

Sex: Female   Day(s) Relative to Start Date

10 mg/kg /dose Group 3	Organ Weights (BW kg)					
	Terminal Body Wt (Kg)	Brain (g)	Brain (%bw)	Gland Adrenal (g)	Gland Adrenal (%br)	Gland Adrenal (%bw)
	-	-	-	-	-	-
3501	2.6	54.640	2.1015	0.460	0.8419	0.0177
3502	2.9	58.830	2.0286	0.620	1.0539	0.0214
3503	2.7	59.070	2.1878	0.740	1.2528	0.0274
Mean	2.73	57.5133	2.10598	0.6067	1.04950	0.02216
SD	0.15	2.4913	0.07967	0.1405	0.20547	0.00490
N	3	3	3	3	3	3
%Diff	-7.87	-15.8957	-9.06247	0.5525	19.59657	9.61872

Appendix 21

Individual Organ Weights: Terminal Euthanasia - Day 31

Sex: Female Day(s) Relative to Start Date

10 mg/kg /dose Group 3	Organ Weights (BW kg)					
	Gland Pituitary (g)	Gland Pituitary (%br)	Gland Pituitary (%bw)	Thyroid/ Parathyroid (g)	Thyroid/ Parathyroid (%br)	Thyroid/ Parathyroid (%bw)
	-	-	-	-	-	-
3501	0.020	0.0366	0.0008	0.260	0.4758	0.0100
3502	0.030	0.0510	0.0010	0.280	0.4759	0.0097
3503	0.060	0.1016	0.0022	0.210	0.3555	0.0078
Mean	0.0367	0.06306	0.00134	0.2500	0.43577	0.00914
SD	0.0208	0.03412	0.00077	0.0361	0.06950	0.00120
N	3	3	3	3	3	3
%Diff	-35.2941	-23.59431	-29.50504	-28.5714	-13.86578	-21.05229

Appendix 21

Individual Organ Weights: Terminal Euthanasia - Day 31

Sex: Female Day(s) Relative to Start Date

10 mg/kg /dose Group 3	Organ Weights (BW kg)					
	Heart (g)	Heart (%br)	Heart (%bw)	Kidney (g)	Kidney (%br)	Kidney (%bw)
	-	-	-	-	-	-
3501	8.670	15.8675	0.3335	10.850	19.8572	0.4173
3502	10.020	17.0321	0.3455	13.170	22.3865	0.4541
3503	8.880	15.0330	0.3289	12.210	20.6704	0.4522
Mean	9.1900	15.97754	0.33596	12.0767	20.97139	0.44122
SD	0.7264	1.00409	0.00859	1.1657	1.29123	0.02073
N	3	3	3	3	3	3
%Diff	-18.1170	-2.33962	-11.05385	-11.6341	5.11842	-4.33686

Appendix 21

Individual Organ Weights: Terminal Euthanasia - Day 31

Sex: Female Day(s) Relative to Start Date

10 mg/kg /dose Group 3	Organ Weights (BW kg)					
	Liver/ Gallbladder (g)	Liver/ Gallbladder (%br)	Liver/ Gallbladder (%bw)	Ovary Paired (g)	Ovary Paired (%br)	Ovary Paired (%bw)
	-	-	-	-	-	-
3501	54.550	99.8353	2.0981	0.170	0.3111	0.0065
3502	56.780	96.5154	1.9579	0.340	0.5779	0.0117
3503	51.490	87.1678	1.9070	0.230	0.3894	0.0085
Mean	54.2733	94.50615	1.98768	0.2467	0.42614	0.00893
SD	2.6558	6.56843	0.09893	0.0862	0.13715	0.00262
N	3	3	3	3	3	3
%Diff	-19.8089	-4.11077	-12.55746	10.4478	29.41132	16.13363

Appendix 21

Individual Organ Weights: Terminal Euthanasia - Day 31

Sex: Female   Day(s) Relative to Start Date

10 mg/kg /dose Group 3	Organ Weights (BW kg)					
	Spleen (g)	Spleen (%br)	Spleen (%bw)	Thymus (g)	Thymus (%br)	Thymus (%bw)
	-	-	-	-	-	-
3501	6.990	12.7928	0.2688	2.970	5.4356	0.1142
3502	4.550	7.7341	0.1569	2.800	4.7595	0.0966
3503	6.520	11.0378	0.2415	2.750	4.6555	0.1019
Mean	6.0200	10.52158	0.22241	2.8400	4.95018	0.10421
SD	1.2946	2.56854	0.05836	0.1153	0.42357	0.00907
N	3	3	3	3	3	3
%Diff	-18.6486	-1.85506	-9.58765	-33.2811	-19.51858	-25.97287

Appendix 21

Individual Organ Weights: Terminal Euthanasia - Day 31

Sex: Female   Day(s) Relative to Start Date

10 mg/kg /dose Group 3	Organ Weights (BW kg)		
	Uterus/ Cervix (g)	Uterus/ Cervix (%br)	Uterus/ Cervix (%bw)
	-	-	-
3501	2.440	4.4656	0.0938
3502	4.410	7.4962	0.1521
3503	5.160	8.7354	0.1911
Mean	4.0033	6.89906	0.14568
SD	1.4049	2.19664	0.04895
N	3	3	3
%Diff	-40.0399	-29.49205	-35.84645

Appendix 21

Individual Organ Weights: Terminal Euthanasia - Day 31

Sex: Female   Day(s) Relative to Start Date

100 mg/kg /dose Group 4	Organ Weights (BW kg)					
	Terminal Body Wt (Kg)	Brain (g)	Brain (%bw)	Gland Adrenal (g)	Gland Adrenal (%br)	Gland Adrenal (%bw)
	-	-	-	-	-	-
4501	2.5	51.600	2.0640	0.590	1.1434	0.0236
4502	3.5	62.970	1.7991	0.780	1.2387	0.0223
4503	2.7	66.550	2.4648	0.550	0.8264	0.0204
Mean	2.90	60.3733	2.10932	0.6400	1.06951	0.02209
SD	0.53	7.8059	0.33514	0.1229	0.21583	0.00162
N	3	3	3	3	3	3
%Diff	-2.25	-11.7134	-8.91823	6.0773	21.87693	9.25111

Appendix 21

Individual Organ Weights: Terminal Euthanasia - Day 31

Sex: Female   Day(s) Relative to Start Date

100 mg/kg /dose Group 4	Organ Weights (BW kg)					
	Gland Pituitary (g)	Gland Pituitary (%br)	Gland Pituitary (%bw)	Thyroid/ Parathyroid (g)	Thyroid/ Parathyroid (%br)	Thyroid/ Parathyroid (%bw)
	-	-	-	-	-	-
4501	0.040	0.0775	0.0016	0.300	0.5814	0.0120
4502	0.050	0.0794	0.0014	0.260	0.4129	0.0074
4503	0.050	0.0751	0.0019	0.300	0.4508	0.0111
Mean	0.0467	0.07735	0.00163	0.2867	0.48169	0.01018
SD	0.0058	0.00214	0.00021	0.0231	0.08840	0.00242
N	3	3	3	3	3	3
%Diff	-17.6471	-6.27457	-14.54279	-18.0952	-4.78790	-12.11160

Appendix 21

Individual Organ Weights: Terminal Euthanasia - Day 31

Sex: Female Day(s) Relative to Start Date

100 mg/kg /dose Group 4	Organ Weights (BW kg)					
	Heart (g)	Heart (%br)	Heart (%bw)	Kidney (g)	Kidney (%br)	Kidney (%bw)
	-	-	-	-	-	-
4501	8.810	17.0736	0.3524	12.110	23.4690	0.4844
4502	12.080	19.1837	0.3451	15.220	24.1702	0.4349
4503	8.920	13.4035	0.3304	13.960	20.9767	0.5170
Mean	9.9367	16.55361	0.34264	13.7633	22.87198	0.47876
SD	1.8570	2.92502	0.01123	1.5643	1.67839	0.04138
N	3	3	3	3	3	3
%Diff	-11.4642	1.18151	-9.28480	0.7073	14.64506	3.80278

Appendix 21

Individual Organ Weights: Terminal Euthanasia - Day 31

Sex: Female Day(s) Relative to Start Date

100 mg/kg /dose Group 4	Organ Weights (BW kg)					
	Liver/ Gallbladder (g)	Liver/ Gallbladder (%br)	Liver/ Gallbladder (%bw)	Ovary Paired (g)	Ovary Paired (%br)	Ovary Paired (%bw)
	-	-	-	-	-	-
4501	58.870	114.0891	2.3548	0.210	0.4070	0.0084
4502	72.720	115.4836	2.0777	0.230	0.3653	0.0066
4503	63.430	95.3118	2.3493	0.490	0.7363	0.0181
Mean	65.0067	108.29484	2.26059	0.3100	0.50284	0.01104
SD	7.0583	11.26524	0.15840	0.1562	0.20325	0.00622
N	3	3	3	3	3	3
%Diff	-3.9500	9.87972	-0.55157	38.8060	52.70215	43.61972

Appendix 21

Individual Organ Weights: Terminal Euthanasia - Day 31

Sex: Female Day(s) Relative to Start Date

100 mg/kg /dose Group 4	Organ Weights (BW kg)					
	Spleen (g)	Spleen (%br)	Spleen (%bw)	Thymus (g)	Thymus (%br)	Thymus (%bw)
	-	-	-	-	-	-
4501	4.260	8.2558	0.1704	2.230	4.3217	0.0892
4502	5.640	8.9566	0.1611	3.370	5.3518	0.0963
4503	4.810	7.2276	0.1781	2.230	3.3509	0.0826
Mean	4.9033	8.14670	0.16990	2.6100	4.34144	0.08936
SD	0.6947	0.86965	0.00851	0.6582	1.00059	0.00685
N	3	3	3	3	3	3
%Diff	-33.7387	-24.00781	-30.93421	-38.6844	-29.41566	-36.52307

Appendix 21

Individual Organ Weights: Terminal Euthanasia - Day 31

Sex: Female   Day(s) Relative to Start Date

100 mg/kg /dose Group 4	Organ Weights (BW kg)		
	Uterus/ Cervix (g)	Uterus/ Cervix (%br)	Uterus/ Cervix (%bw)
	-	-	-
4501	4.990	9.6705	0.1996
4502	5.460	8.6708	0.1560
4503	6.480	9.7370	0.2400
Mean	5.6433	9.35946	0.19853
SD	0.7617	0.59733	0.04201
N	3	3	3
%Diff	-15.4768	-4.34686	-12.56851

Appendix 21

Individual Organ Weights: Recovery Euthanasia - Day 71

Sex: Male Day(s) Relative to Start Date

0 mg/kg /dose Group 1	Organ Weights (BW kg)					
	Terminal Body Wt (Kg)	Brain (g)	Brain (%bw)	Epididymis (g)	Epididymis (%br)	Epididymis (%bw)
	-	-	-	-	-	-
1004	3.1	71.300	2.3000	0.990	1.3885	0.0319
1005	3.6	62.460	1.7350	3.330	5.3314	0.0925
Mean	3.35	66.8800	2.01750	2.1600	3.35996	0.06222
SD	0.35	6.2508	0.39952	1.6546	2.78806	0.04283
N	2	2	2	2	2	2

Appendix 21

Individual Organ Weights: Recovery Euthanasia - Day 71

Sex: Male   Day(s) Relative to Start Date

0 mg/kg /dose Group 1	Organ Weights (BW kg)					
	Gland Adrenal (g)	Gland Adrenal (%br)	Gland Adrenal (%bw)	Gland Pituitary (g)	Gland Pituitary (%br)	Gland Pituitary (%bw)
	-	-	-	-	-	-
1004	0.470	0.6592	0.0152	0.050	0.0701	0.0016
1005	0.590	0.9446	0.0164	0.050	0.0801	0.0014
Mean	0.5300	0.80190	0.01578	0.0500	0.07509	0.00150
SD	0.0849	0.20182	0.00087	0.0000	0.00702	0.00016
N	2	2	2	2	2	2

Appendix 21

Individual Organ Weights: Recovery Euthanasia - Day 71

Sex: Male Day(s) Relative to Start Date

0 mg/kg /dose Group 1	Organ Weights (BW kg)					
	Prostate/ Seminal Ves (g)	Prostate Seminal Ves (%br)	Prostate Seminal Ves (%bw)	Thyroid/ Parathyroid (g)	Thyroid/ Parathyroid (%br)	Thyroid/ Parathyroid (%bw)
	-	-	-	-	-	-
1004	1.080	1.5147	0.0348	0.260	0.3647	0.0084
1005	6.790	10.8710	0.1886	0.290	0.4643	0.0081
Mean	3.9350	6.19284	0.11172	0.2750	0.41448	0.00822
SD	4.0376	6.61585	0.10873	0.0212	0.07046	0.00023
N	2	2	2	2	2	2

Appendix 21

Individual Organ Weights: Recovery Euthanasia - Day 71

Sex: Male Day(s) Relative to Start Date

0 mg/kg /dose Group 1	Organ Weights (BW kg)					
	Heart (g)	Heart (%br)	Heart (%bw)	Kidney (g)	Kidney (%br)	Kidney (%bw)
	-	-	-	-	-	-
1004	13.130	18.4151	0.4235	13.770	19.3128	0.4442
1005	14.050	22.4944	0.3903	18.410	29.4749	0.5114
Mean	13.5900	20.45477	0.40691	16.0900	24.39381	0.47779
SD	0.6505	2.88446	0.02353	3.2810	7.18569	0.04751
N	2	2	2	2	2	2

Appendix 21

Individual Organ Weights: Recovery Euthanasia - Day 71

Sex: Male   Day(s) Relative to Start Date

0 mg/kg /dose Group 1	Organ Weights (BW kg)					
	Liver/ Gallbladder (g)	Liver/ Gallbladder (%br)	Liver/ Gallbladder (%bw)	Spleen (g)	Spleen (%br)	Spleen (%bw)
	-	-	-	-	-	-
1004	64.580	90.5750	2.0832	4.700	6.5919	0.1516
1005	82.880	132.6929	2.3022	9.740	15.5940	0.2706
Mean	73.7300	111.63398	2.19272	7.2200	11.09292	0.21108
SD	12.9401	29.78184	0.15485	3.5638	6.36546	0.08411
N	2	2	2	2	2	2

Appendix 21

Individual Organ Weights: Recovery Euthanasia - Day 71

Sex: Male Day(s) Relative to Start Date

0 mg/kg /dose Group 1	Organ Weights (BW kg)					
	Testis (g)	Testis (%br)	Testis (%bw)	Thymus (g)	Thymus (%br)	Thymus (%bw)
	-	-	-	-	-	-
1004	1.720	2.4123	0.0555	4.300	6.0309	0.1387
1005	28.730	45.9974	0.7981	7.870	12.6001	0.2186
Mean	15.2250	24.20489	0.42677	6.0850	9.31546	0.17866
SD	19.0990	30.81932	0.52508	2.5244	4.64513	0.05650
N	2	2	2	2	2	2

Appendix 21

Individual Organ Weights: Recovery Euthanasia - Day 71

Sex: Male Day(s) Relative to Start Date

100 mg/kg /dose Group 4	Organ Weights (BW kg)					
	Terminal Body Wt (Kg)	Brain (g)	Brain (%bw)	Epididymis (g)	Epididymis (%br)	Epididymis (%bw)
	-	-	-	-	-	-
4004	2.4	66.430	2.7679	0.530	0.7978	0.0221
4005	3.6	75.910	2.1086	3.120	4.1101	0.0867
Mean	3.00	71.1700	2.43826	1.8250	2.45398	0.05438
SD	0.85	6.7034	0.46620	1.8314	2.34215	0.04567
N	2	2	2	2	2	2
%Diff	-10.45	6.4145	20.85571	-15.5093	-26.96388	-12.60531

Appendix 21

Individual Organ Weights: Recovery Euthanasia - Day 71

Sex: Male Day(s) Relative to Start Date

100 mg/kg /dose Group 4	Organ Weights (BW kg)					
	Gland Adrenal (g)	Gland Adrenal (%br)	Gland Adrenal (%bw)	Gland Pituitary (g)	Gland Pituitary (%br)	Gland Pituitary (%bw)
	-	-	-	-	-	-
4004	0.320	0.4817	0.0133	0.020	0.0301	0.0008
4005	0.600	0.7904	0.0167	0.060	0.0790	0.0017
Mean	0.4600	0.63606	0.01500	0.0400	0.05457	0.00125
SD	0.1980	0.21828	0.00236	0.0283	0.03460	0.00059
N	2	2	2	2	2	2
%Diff	-13.2075	-20.68046	-4.91338	-20.0000	-27.32075	-16.71642

Appendix 21

Individual Organ Weights: Recovery Euthanasia - Day 71

Sex: Male   Day(s) Relative to Start Date

100 mg/kg /dose Group 4	Organ Weights (BW kg)					
	Prostate/ Seminal Ves (g)	Prostate Seminal Ves (%br)	Prostate Seminal Ves (%bw)	Thyroid/ Parathyroid (g)	Thyroid/ Parathyroid (%br)	Thyroid/ Parathyroid (%bw)
	-	-	-	-	-	-
4004	1.290	1.9419	0.0538	0.310	0.4667	0.0129
4005	6.140	8.0885	0.1706	0.370	0.4874	0.0103
Mean	3.7150	5.01521	0.11215	0.3400	0.47704	0.01160
SD	3.4295	4.34633	0.08259	0.0424	0.01468	0.00187
N	2	2	2	2	2	2
%Diff	-5.5909	-19.01602	0.38297	23.6364	15.09402	41.06267

Appendix 21

Individual Organ Weights: Recovery Euthanasia - Day 71

Sex: Male Day(s) Relative to Start Date

100 mg/kg /dose Group 4	Organ Weights (BW kg)					
	Heart (g)	Heart (%br)	Heart (%bw)	Kidney (g)	Kidney (%br)	Kidney (%bw)
	-	-	-	-	-	-
4004	9.530	14.3459	0.3971	11.230	16.9050	0.4679
4005	14.160	18.6537	0.3933	15.540	20.4716	0.4317
Mean	11.8450	16.49980	0.39521	13.3850	18.68831	0.44979
SD	3.2739	3.04603	0.00265	3.0476	2.52197	0.02563
N	2	2	2	2	2	2
%Diff	-12.8403	-19.33521	-2.87647	-16.8117	-23.38913	-5.86021

Appendix 21

Individual Organ Weights: Recovery Euthanasia - Day 71

Sex: Male Day(s) Relative to Start Date

100 mg/kg /dose Group 4	Organ Weights (BW kg)					
	Liver/ Gallbladder (g)	Liver/ Gallbladder (%br)	Liver/ Gallbladder (%bw)	Spleen (g)	Spleen (%br)	Spleen (%bw)
	-	-	-	-	-	-
4004	48.720	73.3404	2.0300	5.970	8.9869	0.2488
4005	76.170	100.3425	2.1158	6.260	8.2466	0.1739
Mean	62.4450	86.84143	2.07292	6.1150	8.61676	0.21132
SD	19.4101	19.09341	0.06069	0.2051	0.52347	0.05293
N	2	2	2	2	2	2
%Diff	-15.3058	-22.20878	-5.46386	-15.3047	-22.32204	0.11143

Appendix 21

Individual Organ Weights: Recovery Euthanasia - Day 71

Sex: Male   Day(s) Relative to Start Date

100 mg/kg /dose Group 4	Organ Weights (BW kg)					
	Testis (g)	Testis (%br)	Testis (%bw)	Thymus (g)	Thymus (%br)	Thymus (%bw)
	-	-	-	-	-	-
4004	1.130	1.7010	0.0471	3.700	5.5698	0.1542
4005	23.400	30.8260	0.6500	5.600	7.3772	0.1556
Mean	12.2650	16.26351	0.34854	4.6500	6.47346	0.15486
SD	15.7473	20.59444	0.42633	1.3435	1.27801	0.00098
N	2	2	2	2	2	2
%Diff	-19.4417	-32.80900	-18.33027	-23.5826	-30.50837	-13.32096

Appendix 21

Individual Organ Weights: Recovery Euthanasia - Day 71

Sex: Female Day(s) Relative to Start Date

0 mg/kg /dose Group 1	Organ Weights (BW kg)					
	Terminal Body Wt (Kg)	Brain (g)	Brain (%bw)	Gland Adrenal (g)	Gland Adrenal (%br)	Gland Adrenal (%bw)
	-	-	-	-	-	-
1504	2.7	64.510	2.3893	0.550	0.8526	0.0204
1505	2.7	66.170	2.4507	0.550	0.8312	0.0204
Mean	2.70	65.3400	2.42000	0.5500	0.84189	0.02037
SD	0.00	1.1738	0.04347	0.0000	0.01512	0.00000
N	2	2	2	2	2	2

Appendix 21

Individual Organ Weights: Recovery Euthanasia - Day 71

Sex: Female   Day(s) Relative to Start Date

0 mg/kg /dose Group 1	Organ Weights (BW kg)					
	Gland Pituitary (g)	Gland Pituitary (%br)	Gland Pituitary (%bw)	Thyroid/ Parathyroid (g)	Thyroid/ Parathyroid (%br)	Thyroid/ Parathyroid (%bw)
	-	-	-	-	-	-
1504	0.060	0.0930	0.0022	0.320	0.4960	0.0119
1505	0.030	0.0453	0.0011	0.150	0.2267	0.0056
Mean	0.0450	0.06917	0.00167	0.2350	0.36137	0.00870
SD	0.0212	0.03371	0.00079	0.1202	0.19047	0.00445
N	2	2	2	2	2	2

Appendix 21

Individual Organ Weights: Recovery Euthanasia - Day 71

Sex: Female   Day(s) Relative to Start Date

0 mg/kg /dose Group 1	Organ Weights (BW kg)					
	Heart (g)	Heart (%br)	Heart (%bw)	Kidney (g)	Kidney (%br)	Kidney (%bw)
	-	-	-	-	-	-
1504	9.340	14.4784	0.3459	14.070	21.8106	0.5211
1505	8.450	12.7701	0.3130	12.840	19.4046	0.4756
Mean	8.8950	13.62426	0.32944	13.4550	20.60757	0.49833
SD	0.6293	1.20791	0.02331	0.8697	1.70130	0.03221
N	2	2	2	2	2	2

Appendix 21

Individual Organ Weights: Recovery Euthanasia - Day 71

Sex: Female   Day(s) Relative to Start Date

0 mg/kg /dose Group 1	Organ Weights (BW kg)					
	Liver/ Gallbladder (g)	Liver/ Gallbladder (%br)	Liver/ Gallbladder (%bw)	Ovary Paired (g)	Ovary Paired (%br)	Ovary Paired (%bw)
	-	-	-	-	-	-
1504	62.470	96.8377	2.3137	0.330	0.5115	0.0122
1505	50.340	76.0768	1.8644	0.210	0.3174	0.0078
Mean	56.4050	86.45724	2.08907	0.2700	0.41446	0.01000
SD	8.5772	14.68019	0.31767	0.0849	0.13731	0.00314
N	2	2	2	2	2	2

Appendix 21

Individual Organ Weights: Recovery Euthanasia - Day 71

Sex: Female Day(s) Relative to Start Date

0 mg/kg /dose Group 1	Organ Weights (BW kg)					
	Spleen (g)	Spleen (%br)	Spleen (%bw)	Thymus (g)	Thymus (%br)	Thymus (%bw)
	-	-	-	-	-	-
1504	6.620	10.2620	0.2452	2.740	4.2474	0.1015
1505	6.630	10.0196	0.2456	5.570	8.4177	0.2063
Mean	6.6250	10.14081	0.24537	4.1550	6.33256	0.15389
SD	0.0071	0.17135	0.00026	2.0011	2.94885	0.07412
N	2	2	2	2	2	2

Appendix 21

Individual Organ Weights: Recovery Euthanasia - Day 71

Sex: Female   Day(s) Relative to Start Date

0 mg/kg /dose Group 1	Organ Weights (BW kg)		
	Uterus/ Cervix (g)	Uterus/ Cervix (%br)	Uterus/ Cervix (%bw)
	-	-	-
1504	8.770	13.5948	0.3248
1505	4.010	6.0601	0.1485
Mean	6.3900	9.82747	0.23667
SD	3.3658	5.32780	0.12466
N	2	2	2

Appendix 21

Individual Organ Weights: Recovery Euthanasia - Day 71

Sex: Female   Day(s) Relative to Start Date

100 mg/kg /dose Group 4	Organ Weights (BW kg)					
	Terminal Body Wt (Kg)	Brain (g)	Brain (%bw)	Gland Adrenal (g)	Gland Adrenal (%br)	Gland Adrenal (%bw)
	-	-	-	-	-	-
4504	2.7	55.950	2.0722	0.520	0.9294	0.0193
4505	2.9	57.830	1.9941	0.620	1.0721	0.0214
Mean	2.80	56.8900	2.03318	0.5700	1.00075	0.02032
SD	0.14	1.3294	0.05521	0.0707	0.10091	0.00150
N	2	2	2	2	2	2
%Diff	3.70	-12.9324	-15.98429	3.6364	18.87046	-0.25078

Appendix 21

Individual Organ Weights: Recovery Euthanasia - Day 71

Sex: Female   Day(s) Relative to Start Date

100 mg/kg /dose Group 4	Organ Weights (BW kg)					
	Gland Pituitary (g)	Gland Pituitary (%br)	Gland Pituitary (%bw)	Thyroid/ Parathyroid (g)	Thyroid/ Parathyroid (%br)	Thyroid/ Parathyroid (%bw)
	-	-	-	-	-	-
4504	0.040	0.0715	0.0015	0.210	0.3753	0.0078
4505	0.030	0.0519	0.0010	0.290	0.5015	0.0100
Mean	0.0350	0.06168	0.00126	0.2500	0.43840	0.00889
SD	0.0071	0.01387	0.00032	0.0566	0.08919	0.00157
N	2	2	2	2	2	2
%Diff	-22.2222	-10.82644	-24.52107	6.3830	21.31747	2.12766

Appendix 21

Individual Organ Weights: Recovery Euthanasia - Day 71

Sex: Female   Day(s) Relative to Start Date

100 mg/kg /dose Group 4	Organ Weights (BW kg)					
	Heart (g)	Heart (%br)	Heart (%bw)	Kidney (g)	Kidney (%br)	Kidney (%bw)
	-	-	-	-	-	-
4504	8.720	15.5853	0.3230	12.920	23.0920	0.4785
4505	11.230	19.4190	0.3872	13.940	24.1051	0.4807
Mean	9.9750	17.50217	0.35510	13.4300	23.59859	0.47960
SD	1.7748	2.71079	0.04545	0.7212	0.71636	0.00154
N	2	2	2	2	2	2
%Diff	12.1417	28.46327	7.78818	-0.1858	14.51420	-3.75838

Appendix 21

Individual Organ Weights: Recovery Euthanasia - Day 71

Sex: Female Day(s) Relative to Start Date

100 mg/kg /dose Group 4	Organ Weights (BW kg)					
	Liver/ Gallbladder (g)	Liver/ Gallbladder (%br)	Liver/ Gallbladder (%bw)	Ovary Paired (g)	Ovary Paired (%br)	Ovary Paired (%bw)
	-	-	-	-	-	-
4504	56.880	101.6622	2.1067	0.200	0.3575	0.0074
4505	66.980	115.8222	2.3097	0.580	1.0029	0.0200
Mean	61.9300	108.74222	2.20816	0.3900	0.68020	0.01370
SD	7.1418	10.01266	0.14353	0.2687	0.45642	0.00890
N	2	2	2	2	2	2
%Diff	9.7952	25.77573	5.70046	44.4444	64.11876	37.03704

Appendix 21

Individual Organ Weights: Recovery Euthanasia - Day 71

Sex: Female Day(s) Relative to Start Date

100 mg/kg /dose Group 4	Organ Weights (BW kg)					
	Spleen (g)	Spleen (%br)	Spleen (%bw)	Thymus (g)	Thymus (%br)	Thymus (%bw)
	-	-	-	-	-	-
4504	5.500	9.8302	0.2037	1.160	2.0733	0.0430
4505	5.950	10.2888	0.2052	2.870	4.9628	0.0990
Mean	5.7250	10.05949	0.20444	2.0150	3.51805	0.07096
SD	0.3182	0.32426	0.00104	1.2092	2.04321	0.03960
N	2	2	2	2	2	2
%Diff	-13.5849	-0.80190	-16.68185	-51.5042	-44.44502	-53.88605

Appendix 21

Individual Organ Weights: Recovery Euthanasia - Day 71

Sex: Female   Day(s) Relative to Start Date

100 mg/kg /dose Group 4	Organ Weights (BW kg)		
	Uterus/ Cervix (g)	Uterus/ Cervix (%br)	Uterus/ Cervix (%bw)
	-	-	-
4504	6.930	12.3861	0.2567
4505	6.580	11.3782	0.2269
Mean	6.7550	11.88212	0.24178
SD	0.2475	0.71268	0.02105
N	2	2	2
%Diff	5.7121	20.90720	2.16124

Appendix 21

Individual Macroscopic and Microscopic Pathology

Animal:	1001	Group:	1	Sex:	Male
Species:	Monkey	Strain:	Cyno Mauritius Island		
		Dose:	0 mg/kg /dose		
		Removal Reason:	Terminal Euthanasia		
		Study Day (Week) of Death:	31 (5)		
				Gross Status:	Complete
				Histo Status:	Complete

Gross Pathology Animal Details:

Animal Comment: Gross examination was performed

Gross Pathology Observations [Correlation]:

GLAND, PITUITARY : (Comment) Damaged At Necropsy.

Any remaining protocol required tissues, which have been examined, have no visible lesions

Gross Pathology - The following Tissues were Not Examined:

None

Histopathology Animal Details:

No animal details found

Histopathology Observations [Correlation]:

EYE : (Note) Maculae Unavailable For Evaluation
PANCREAS : Infiltration, mononuclear cell; mild
STOMACH : Hyperplasia/hyperkeratosis; mild
STOMACH : Metaplasia, squamous; focal, moderate, fundus
STOMACH : Infiltration, lymphocytic; mucosal, moderate

Histopathology - The following Tissues were Within Normal Limits:

ARTERY, AORTA; BONE MARROW, STERNUM; BONE, FEMUR; BONE, STERNUM; BRAIN; EPIDIDYMIS; ESOPHAGUS; EYE; GALLBLADDER; GALT; GLAND, ADRENAL; GLAND, MAMMARY; GLAND, PITUITARY; GLAND, PROSTATE; GLAND, SEMINAL VESICLE; GLAND, THYROID; HEART; JOINT, FEMOROTIBIAL; KIDNEY; LARGE INTESTINE, CECUM; LARGE INTESTINE, COLON; LARGE INTESTINE, RECTUM; LIVER; LUNG; LYMPH NODE, AXILLARY; LYMPH NODE, MANDIBULAR; LYMPH NODE, MESENTERIC; MUSCLE, QUADRICEPS; NERVE, OPTIC; NERVE, SCIATIC; SITE, INFUSION, CEPHALIC, LEFT; SITE, INFUSION, CEPHALIC, RIGHT; SKIN; SMALL INTESTINE, DUODENUM; SMALL INTESTINE, ILEUM; SMALL INTESTINE, JEJUNUM; SPINAL CORD; SPLEEN; TESTIS; THYMUS; TONGUE; TRACHEA; URINARY BLADDER; GLAND, SALIVARY, SUBMANDIBULAR

Histopathology - The following Tissues were Not Examined:

GLAND, PARATHYROID - Not Present In Section.

Appendix 21

Individual Macroscopic and Microscopic Pathology

Animal:	1002	Group:	1	Sex:	Male
Species:	Monkey	Strain:	Cyno Mauritius Island		
		Dose:	0 mg/kg /dose		
		Removal Reason:	Terminal Euthanasia		
		Study Day (Week) of Death:	31 (5)		
				Gross Status:	Complete
				Histo Status:	Complete

Gross Pathology Animal Details:

Animal Comment: Gross examination was performed

Gross Pathology Observations [Correlation]:

No observations found

Any remaining protocol required tissues, which have been examined, have no visible lesions

Gross Pathology - The following Tissues were Not Examined:

None

Histopathology Animal Details:

No animal details found

Histopathology Observations [Correlation]:

EYE : (Note) Maculae Unavailable For Evaluation
GLAND, PARATHYROID : (Note) One Of A Pair Available For Evaluation.
THYMUS : Cellularity, decreased; cortical, mild

Histopathology - The following Tissues were Within Normal Limits:

ARTERY, AORTA; BONE MARROW, STERNUM; BONE, FEMUR; BONE, STERNUM; BRAIN; EPIDIDYMIS; ESOPHAGUS; EYE; GALLBLADDER; GALT; GLAND, ADRENAL; GLAND, MAMMARY; GLAND, PARATHYROID; GLAND, PITUITARY; GLAND, PROSTATE; GLAND, SEMINAL VESICLE; GLAND, THYROID; HEART; JOINT, FEMOROTIBIAL; KIDNEY; LARGE INTESTINE, CECUM; LARGE INTESTINE, COLON; LARGE INTESTINE, RECTUM; LIVER; LUNG; LYMPH NODE, AXILLARY; LYMPH NODE, MANDIBULAR; LYMPH NODE, MESENTERIC; MUSCLE, QUADRICEPS; NERVE, OPTIC; NERVE, SCIATIC; PANCREAS; SITE, INFUSION, CEPHALIC, LEFT; SITE, INFUSION, CEPHALIC, RIGHT; SKIN; SMALL INTESTINE, DUODENUM; SMALL INTESTINE, ILEUM; SMALL INTESTINE, JEJUNUM; SPINAL CORD; SPLEEN; STOMACH; TESTIS; TONGUE; TRACHEA; URINARY BLADDER; GLAND, SALIVARY, SUBMANDIBULAR

Histopathology - The following Tissues were Not Examined:

None

Appendix 21

Individual Macroscopic and Microscopic Pathology

Animal:	1003	Group:	1	Sex:	Male
Species:	Monkey	Strain:	Cyno Mauritius Island		
		Dose:	0 mg/kg /dose		
		Removal Reason:	Terminal Euthanasia		
		Study Day (Week) of Death:	31 (5)		
				Gross Status:	Complete
				Histo Status:	Complete

Gross Pathology Animal Details:

Animal Comment: Gross examination was performed

Gross Pathology Observations [Correlation]:

No observations found

Any remaining protocol required tissues, which have been examined, have no visible lesions

Gross Pathology - The following Tissues were Not Examined:

None

Histopathology Animal Details:

No animal details found

Histopathology Observations [Correlation]:

EYE : (Note) Maculae Unavailable For Evaluation
GLAND, ADRENAL : Vacuolation; cortical, minimal
GLAND, THYROID : Cyst

Histopathology - The following Tissues were Within Normal Limits:

ARTERY, AORTA; BONE MARROW, STERNUM; BONE, FEMUR; BONE, STERNUM; BRAIN; EPIDIDYMIS; ESOPHAGUS; EYE; GALLBLADDER; GALT; GLAND, MAMMARY; GLAND, PITUITARY; GLAND, PROSTATE; GLAND, SEMINAL VESICLE; HEART; JOINT, FEMOROTIBIAL; KIDNEY; LARGE INTESTINE, CECUM; LARGE INTESTINE, COLON; LARGE INTESTINE, RECTUM; LIVER; LUNG; LYMPH NODE, AXILLARY; LYMPH NODE, MANDIBULAR; LYMPH NODE, MESENTERIC; MUSCLE, QUADRICEPS; NERVE, OPTIC; NERVE, SCIATIC; PANCREAS; SITE, INFUSION, CEPHALIC, LEFT; SITE, INFUSION, CEPHALIC, RIGHT; SKIN; SMALL INTESTINE, DUODENUM; SMALL INTESTINE, ILEUM; SMALL INTESTINE, JEJUNUM; SPINAL CORD; SPLEEN; STOMACH; TESTIS; THYMUS; TONGUE; TRACHEA; URINARY BLADDER; GLAND, SALIVARY, SUBMANDIBULAR

Histopathology - The following Tissues were Not Examined:

GLAND, PARATHYROID - Not Present In Section.

Appendix 21

Individual Macroscopic and Microscopic Pathology

Animal:	1004	Group:	1	Sex:	Male
Species:	Monkey	Strain:	Cyno Mauritius Island		
		Dose:	0 mg/kg /dose		
		Removal Reason:	Recovery Euthanasia		
		Study Day (Week) of Death:	71 (11)		
				Gross Status:	Complete
				Histo Status:	Complete

Gross Pathology Animal Details:

Animal Comment: Gross examination was performed

Gross Pathology Observations [Correlation]:

No observations found

Any remaining protocol required tissues, which have been examined, have no visible lesions

Gross Pathology - The following Tissues were Not Examined:

None

Histopathology Animal Details:

No animal details found

Histopathology Observations [Correlation]:

EYE : (Note) Maculae Unavailable For Evaluation
GLAND, PARATHYROID : (Note) One Of A Pair Available For Evaluation.
PANCREAS : Infiltration, mononuclear cell; mild : (Comment) On Slide #24 with duodenum

Histopathology - The following Tissues were Within Normal Limits:

ARTERY, AORTA; BONE MARROW, STERNUM; BONE, FEMUR; BONE, STERNUM; BRAIN; EPIDIDYMIS; ESOPHAGUS; EYE; GALLBLADDER; GALT; GLAND, ADRENAL; GLAND, MAMMARY; GLAND, PARATHYROID; GLAND, PITUITARY; GLAND, PROSTATE; GLAND, SEMINAL VESICLE; GLAND, THYROID; HEART; JOINT, FEMOROTIBIAL; KIDNEY; LARGE INTESTINE, CECUM; LARGE INTESTINE, COLON; LARGE INTESTINE, RECTUM; LIVER; LUNG; LYMPH NODE, AXILLARY; LYMPH NODE, MANDIBULAR; LYMPH NODE, MESENTERIC; MUSCLE, QUADRICEPS; NERVE, OPTIC; NERVE, SCIATIC; SITE, INFUSION, CEPHALIC, LEFT; SITE, INFUSION, CEPHALIC, RIGHT; SKIN; SMALL INTESTINE, DUODENUM; SMALL INTESTINE, ILEUM; SMALL INTESTINE, JEJUNUM; SPINAL CORD; SPLEEN; STOMACH; TESTIS; THYMUS; TONGUE; TRACHEA; URINARY BLADDER; GLAND, SALIVARY, SUBMANDIBULAR

Histopathology - The following Tissues were Not Examined:

None

Appendix 21

Individual Macroscopic and Microscopic Pathology

Animal:	1005	Group:	1	Sex:	Male
Species:	Monkey	Strain:	Cyno Mauritius Island		
		Dose:	0 mg/kg /dose		
		Removal Reason:	Recovery Euthanasia		
		Study Day (Week) of Death:	71 (11)		
				Gross Status:	Complete
				Histo Status:	Complete

Gross Pathology Animal Details:

Animal Comment: Gross examination was performed

Gross Pathology Observations [Correlation]:

No observations found

Any remaining protocol required tissues, which have been examined, have no visible lesions

Gross Pathology - The following Tissues were Not Examined:

None

Histopathology Animal Details:

No animal details found

Histopathology Observations [Correlation]:

EYE : (Note) Maculae Unavailable For Evaluation
GLAND, ADRENAL : Mineralization; minimal
LIVER : Vacuolation; hepatocellular, mild
LIVER : Infiltration, mononuclear cell; minimal
SPINAL CORD : Mineralization; moderate

Histopathology - The following Tissues were Within Normal Limits:

ARTERY, AORTA; BONE MARROW, STERNUM; BONE, FEMUR; BONE, STERNUM; BRAIN; EPIDIDYMIS; ESOPHAGUS; EYE; GALLBLADDER; GALT; GLAND, MAMMARY; GLAND, PITUITARY; GLAND, PROSTATE; GLAND, SEMINAL VESICLE; GLAND, THYROID; HEART; JOINT, FEMOROTIBIAL; KIDNEY; LARGE INTESTINE, CECUM; LARGE INTESTINE, COLON; LARGE INTESTINE, RECTUM; LUNG; LYMPH NODE, AXILLARY; LYMPH NODE, MANDIBULAR; LYMPH NODE, MESENTERIC; MUSCLE, QUADRICEPS; NERVE, OPTIC; NERVE, SCIATIC; PANCREAS; SITE, INFUSION, CEPHALIC, LEFT; SITE, INFUSION, CEPHALIC, RIGHT; SKIN; SMALL INTESTINE, DUODENUM; SMALL INTESTINE, ILEUM; SMALL INTESTINE, JEJUNUM; SPLEEN; STOMACH; TESTIS; THYMUS; TONGUE; TRACHEA; GLAND, SALIVARY, SUBMANDIBULAR

Histopathology - The following Tissues were Not Examined:

GLAND, PARATHYROID - Not Present In Section.
URINARY BLADDER - Not Present In Section.

Appendix 21

Individual Macroscopic and Microscopic Pathology

Animal:	1501	Group:	1	Sex:	Female
Species:	Monkey	Strain:	Cyno Mauritius Island		
		Dose:	0 mg/kg /dose		
		Removal Reason:	Terminal Euthanasia		
		Study Day (Week) of Death:	31 (5)		
				Gross Status:	Complete
				Histo Status:	Complete

Gross Pathology Animal Details:

Animal Comment: Gross examination was performed

Gross Pathology Observations [Correlation]:

LUNG : Adhesion; left, unilateral, thoracic wall (TGL)
OVARY : Small; bilateral (TGL)

Any remaining protocol required tissues, which have been examined, have no visible lesions

Gross Pathology - The following Tissues were Not Examined:

None

Histopathology Animal Details:

No animal details found

Histopathology Observations [Correlation]:

KIDNEY : Infiltration, mononuclear cell; minimal
LUNG : Fibrosis; pleural, mild [LUNG : Adhesion; left, unilateral, thoracic wall (G)]
OVARY : Cyst
OVARY : Immaturity [OVARY : Small; bilateral (G)]
URINARY BLADDER : Infiltration, mononuclear cell; submucosal, focal, minimal

Histopathology - The following Tissues were Within Normal Limits:

ARTERY, AORTA; BONE MARROW, STERNUM; BONE, FEMUR; BONE, STERNUM; BRAIN; CERVIX; ESOPHAGUS; EYE; GALLBLADDER; GALT; GLAND, ADRENAL; GLAND, MAMMARY; GLAND, PARATHYROID; GLAND, PITUITARY; GLAND, THYROID; HEART; JOINT, FEMOROTIBIAL; LARGE INTESTINE, CECUM; LARGE INTESTINE, COLON; LARGE INTESTINE, RECTUM; LIVER; LYMPH NODE, AXILLARY; LYMPH NODE, MANDIBULAR; LYMPH NODE, MESENTERIC; MUSCLE, QUADRICEPS; NERVE, OPTIC; NERVE, SCIATIC; PANCREAS; SITE, INFUSION, CEPHALIC, LEFT; SITE, INFUSION, CEPHALIC, RIGHT; SKIN; SMALL INTESTINE, DUODENUM; SMALL INTESTINE, ILEUM; SMALL INTESTINE, JEJUNUM; SPINAL CORD; SPLEEN; STOMACH; THYMUS; TONGUE; TRACHEA; UTERUS; VAGINA; GLAND, SALIVARY, SUBMANDIBULAR

Histopathology - The following Tissues were Not Examined:

None

Appendix 21

Individual Macroscopic and Microscopic Pathology

Animal:	1502	Group:	1	Sex:	Female
Species:	Monkey	Strain:	Cyno Mauritius Island		
		Dose:	0 mg/kg /dose		
		Removal Reason:	Terminal Euthanasia		
		Study Day (Week) of Death:	31 (5)		
				Gross Status:	Complete
				Histo Status:	Complete

Gross Pathology Animal Details:

Animal Comment: Gross examination was performed

Gross Pathology Observations [Correlation]:

SPLEEN : (Comment) Accessory

Any remaining protocol required tissues, which have been examined, have no visible lesions

Gross Pathology - The following Tissues were Not Examined:

None

Histopathology Animal Details:

No animal details found

Histopathology Observations [Correlation]:

GLAND, PARATHYROID : (Note) One Of A Pair Available For Evaluation.
LYMPH NODE, AXILLARY : Pigment; minimal : (Comment) Black
SPLEEN : Inflammation, mixed cell; mild

Histopathology - The following Tissues were Within Normal Limits:

ARTERY, AORTA; BONE MARROW, STERNUM; BONE, FEMUR; BONE, STERNUM; BRAIN; CERVIX; ESOPHAGUS; EYE; GALLBLADDER; GALT; GLAND, ADRENAL; GLAND, MAMMARY; GLAND, PARATHYROID; GLAND, PITUITARY; GLAND, THYROID; HEART; JOINT, FEMOROTIBIAL; KIDNEY; LARGE INTESTINE, CECUM; LARGE INTESTINE, COLON; LARGE INTESTINE, RECTUM; LIVER; LUNG; LYMPH NODE, MANDIBULAR; LYMPH NODE, MESENTERIC; MUSCLE, QUADRICEPS; NERVE, OPTIC; NERVE, SCIATIC; OVARY; PANCREAS; SITE, INFUSION, CEPHALIC, LEFT; SITE, INFUSION, CEPHALIC, RIGHT; SKIN; SMALL INTESTINE, DUODENUM; SMALL INTESTINE, ILEUM; SMALL INTESTINE, JEJUNUM; SPINAL CORD; STOMACH; THYMUS; TONGUE; TRACHEA; URINARY BLADDER; UTERUS; VAGINA; GLAND, SALIVARY, SUBMANDIBULAR

Histopathology - The following Tissues were Not Examined:

None

Appendix 21

Individual Macroscopic and Microscopic Pathology

Animal:	1503	Group:	1	Sex:	Female
Species:	Monkey	Strain:	Cyno Mauritius Island		
		Dose:	0 mg/kg /dose		
		Removal Reason:	Terminal Euthanasia		
		Study Day (Week) of Death:	31 (5)		
				Gross Status:	Complete
				Histo Status:	Complete

Gross Pathology Animal Details:

Animal Comment: Gross examination was performed

Gross Pathology Observations [Correlation]:

OVARY : Small; left (TGL)
UTERUS : Discoloration, dark; red (TGL)

Any remaining protocol required tissues, which have been examined, have no visible lesions

Gross Pathology - The following Tissues were Not Examined:

None

Histopathology Animal Details:

No animal details found

Histopathology Observations [Correlation]:

GLAND, PARATHYROID : (Note) One Of A Pair Available For Evaluation.
GLAND, PITUITARY : (Note) Only Pars Intermedia Available For Evaluation.
KIDNEY : Infiltration, mononuclear cell; minimal
LUNG : Infiltration, mononuclear cell; minimal
MUSCLE, QUADRICEPS : Inflammation; chronic, mild
OVARY : Hypertrophy; moderate, corpus luteum [OVARY : Small; left (G)]
NO CORRELATE : No correlating lesion [UTERUS : Discoloration, dark; red (G)]

Histopathology - The following Tissues were Within Normal Limits:

ARTERY, AORTA; BONE MARROW, STERNUM; BONE, FEMUR; BONE, STERNUM; BRAIN; CERVIX; ESOPHAGUS; EYE; GALLBLADDER; GALT; GLAND, ADRENAL; GLAND, MAMMARY; GLAND, PARATHYROID; GLAND, PITUITARY; GLAND, THYROID; HEART; JOINT, FEMOROTIBIAL; LARGE INTESTINE, CECUM; LARGE INTESTINE, COLON; LARGE INTESTINE, RECTUM; LIVER; LYMPH NODE, AXILLARY; LYMPH NODE, MANDIBULAR; LYMPH NODE, MESENTERIC; NERVE, OPTIC; NERVE, SCIATIC; PANCREAS; SITE, INFUSION, CEPHALIC, LEFT; SITE, INFUSION, CEPHALIC, RIGHT; SKIN; SMALL INTESTINE, DUODENUM; SMALL INTESTINE, ILEUM; SMALL INTESTINE, JEJUNUM; SPINAL CORD; SPLEEN; STOMACH; THYMUS; TONGUE; TRACHEA; URINARY BLADDER; UTERUS; VAGINA; GLAND, SALIVARY, SUBMANDIBULAR

Histopathology - The following Tissues were Not Examined:

None

Appendix 21

Individual Macroscopic and Microscopic Pathology

Animal:	1504	Group:	1	Sex:	Female
Species:	Monkey	Strain:	Cyno Mauritius Island		
		Dose:	0 mg/kg /dose		
		Removal Reason:	Recovery Euthanasia		
		Study Day (Week) of Death:	71 (11)		
				Gross Status:	Complete
				Histo Status:	Complete

Gross Pathology Animal Details:

Animal Comment: Gross examination was performed

Gross Pathology Observations [Correlation]:

No observations found

Any remaining protocol required tissues, which have been examined, have no visible lesions

Gross Pathology - The following Tissues were Not Examined:

None

Histopathology Animal Details:

No animal details found

Histopathology Observations [Correlation]:

KIDNEY : Infiltration, mononuclear cell; minimal
STOMACH : Granuloma; submucosal, focal, mild

Histopathology - The following Tissues were Within Normal Limits:

ARTERY, AORTA; BONE MARROW, STERNUM; BONE, FEMUR; BONE, STERNUM; BRAIN; CERVIX; ESOPHAGUS; EYE; GALLBLADDER; GALT; GLAND, ADRENAL; GLAND, MAMMARY; GLAND, PITUITARY; GLAND, THYROID; HEART; JOINT, FEMOROTIBIAL; LARGE INTESTINE, CECUM; LARGE INTESTINE, COLON; LARGE INTESTINE, RECTUM; LIVER; LUNG; LYMPH NODE, AXILLARY; LYMPH NODE, MANDIBULAR; LYMPH NODE, MESENTERIC; MUSCLE, QUADRICEPS; NERVE, OPTIC; NERVE, SCIATIC; OVARY; PANCREAS; SITE, INFUSION, CEPHALIC, LEFT; SITE, INFUSION, CEPHALIC, RIGHT; SKIN; SMALL INTESTINE, DUODENUM; SMALL INTESTINE, ILEUM; SMALL INTESTINE, JEJUNUM; SPINAL CORD; SPLEEN; THYMUS; TONGUE; TRACHEA; URINARY BLADDER; UTERUS; VAGINA; GLAND, SALIVARY, SUBMANDIBULAR

Histopathology - The following Tissues were Not Examined:

GLAND, PARATHYROID - Not Present In Section.

Appendix 21

Individual Macroscopic and Microscopic Pathology

Animal:	1505	Group:	1	Sex:	Female
Species:	Monkey	Strain:	Cyno Mauritius Island		
		Dose:	0 mg/kg /dose		
		Removal Reason:	Recovery Euthanasia		
		Study Day (Week) of Death:	71 (11)		
				Gross Status:	Complete
				Histo Status:	Complete

Gross Pathology Animal Details:

Animal Comment: Gross examination was performed

Gross Pathology Observations [Correlation]:

THYMUS : Enlargement (TGL)

Any remaining protocol required tissues, which have been examined, have no visible lesions

Gross Pathology - The following Tissues were Not Examined:

None

Histopathology Animal Details:

No animal details found

Histopathology Observations [Correlation]:

GLAND, PARATHYROID : (Note) One Of A Pair Available For Evaluation.
GLAND, PITUITARY : (Note) Only Pars Distalis Available For Evaluation.
GLAND, THYROID : Cyst
NO CORRELATE : No correlating lesion [THYMUS : Enlargement (G)]

Histopathology - The following Tissues were Within Normal Limits:

ARTERY, AORTA; BONE MARROW, STERNUM; BONE, FEMUR; BONE, STERNUM; BRAIN; CERVIX; ESOPHAGUS; EYE; GALLBLADDER; GALT; GLAND, ADRENAL; GLAND, MAMMARY; GLAND, PARATHYROID; GLAND, PITUITARY; HEART; JOINT, FEMOROTIBIAL; KIDNEY; LARGE INTESTINE, CECUM; LARGE INTESTINE, COLON; LARGE INTESTINE, RECTUM; LIVER; LUNG; LYMPH NODE, AXILLARY; LYMPH NODE, MANDIBULAR; LYMPH NODE, MESENTERIC; MUSCLE, QUADRICEPS; NERVE, OPTIC; NERVE, SCIATIC; OVARY; PANCREAS; SITE, INFUSION, CEPHALIC, LEFT; SITE, INFUSION, CEPHALIC, RIGHT; SKIN; SMALL INTESTINE, DUODENUM; SMALL INTESTINE, ILEUM; SMALL INTESTINE, JEJUNUM; SPINAL CORD; SPLEEN; STOMACH; THYMUS; TONGUE; TRACHEA; URINARY BLADDER; UTERUS; VAGINA; GLAND, SALIVARY, SUBMANDIBULAR

Histopathology - The following Tissues were Not Examined:

None

Appendix 21

Individual Macroscopic and Microscopic Pathology

Animal:	2001	Group:	2	Sex:	Male
Species:	Monkey	Strain:	Cyno Mauritius Island		
		Dose:	3 mg/kg /dose		
		Removal Reason:	Terminal Euthanasia		
		Study Day (Week) of Death:	31 (5)		
				Gross Status:	Complete
				Histo Status:	Complete

Gross Pathology Animal Details:

Animal Comment: Gross examination was performed

Gross Pathology Observations [Correlation]:

No observations found

Any remaining protocol required tissues, which have been examined, have no visible lesions

Gross Pathology - The following Tissues were Not Examined:

None

Histopathology Animal Details:

No animal details found

Histopathology Observations [Correlation]:

GLAND, ADRENAL : Infiltration, mononuclear cell; minimal
KIDNEY : Infiltration, mononuclear cell; minimal
LIVER : Infiltration, mononuclear cell; minimal
SITE, INFUSION, CEPHALIC, LEFT : Infiltration, mixed cell; minimal, subcutaneous tissue

Histopathology - The following Tissues were Within Normal Limits:

ARTERY, AORTA; BONE MARROW, STERNUM; BONE, FEMUR; BONE, STERNUM; BRAIN; EPIDIDYMIS; ESOPHAGUS; EYE; GALLBLADDER; GALT; GLAND, MAMMARY; GLAND, PARATHYROID; GLAND, PITUITARY; GLAND, PROSTATE; GLAND, SEMINAL VESICLE; GLAND, THYROID; HEART; JOINT, FEMOROTIBIAL; LARGE INTESTINE, CECUM; LARGE INTESTINE, COLON; LARGE INTESTINE, RECTUM; LUNG; LYMPH NODE, AXILLARY; LYMPH NODE, MANDIBULAR; LYMPH NODE, MESENTERIC; MUSCLE, QUADRICEPS; NERVE, OPTIC; NERVE, SCIATIC; PANCREAS; SITE, INFUSION, CEPHALIC, RIGHT; SKIN; SMALL INTESTINE, DUODENUM; SMALL INTESTINE, ILEUM; SMALL INTESTINE, JEJUNUM; SPINAL CORD; SPLEEN; STOMACH; TESTIS; THYMUS; TONGUE; TRACHEA; URINARY BLADDER; GLAND, SALIVARY, SUBMANDIBULAR

Histopathology - The following Tissues were Not Examined:

None

Appendix 21

Individual Macroscopic and Microscopic Pathology

Animal:	2002	Group:	2	Sex:	Male
Species:	Monkey	Strain:	Cyno Mauritius Island		
		Dose:	3 mg/kg /dose		
		Removal Reason:	Terminal Euthanasia		
		Study Day (Week) of Death:	31 (5)		
				Gross Status:	Complete
				Histo Status:	Complete

Gross Pathology Animal Details:

Animal Comment: Gross examination was performed

Gross Pathology Observations [Correlation]:

GLAND, PITUITARY : (Comment) Damaged At Necropsy.

Any remaining protocol required tissues, which have been examined, have no visible lesions

Gross Pathology - The following Tissues were Not Examined:

None

Histopathology Animal Details:

No animal details found

Histopathology Observations [Correlation]:

GLAND, PARATHYROID : (Note) One Of A Pair Available For Evaluation.
GLAND, PITUITARY : (Note) Only Pars Distalis Available For Evaluation.

Histopathology - The following Tissues were Within Normal Limits:

ARTERY, AORTA; BONE MARROW, STERNUM; BONE, FEMUR; BONE, STERNUM; BRAIN; EPIDIDYMIS; ESOPHAGUS; EYE; GALLBLADDER; GALT; GLAND, ADRENAL; GLAND, MAMMARY; GLAND, PARATHYROID; GLAND, PITUITARY; GLAND, PROSTATE; GLAND, SEMINAL VESICLE; GLAND, THYROID; HEART; JOINT, FEMOROTIBIAL; KIDNEY; LARGE INTESTINE, CECUM; LARGE INTESTINE, COLON; LARGE INTESTINE, RECTUM; LIVER; LUNG; LYMPH NODE, AXILLARY; LYMPH NODE, MANDIBULAR; LYMPH NODE, MESENTERIC; MUSCLE, QUADRICEPS; NERVE, OPTIC; NERVE, SCIATIC; PANCREAS; SITE, INFUSION, CEPHALIC, LEFT; SITE, INFUSION, CEPHALIC, RIGHT; SKIN; SMALL INTESTINE, DUODENUM; SMALL INTESTINE, ILEUM; SMALL INTESTINE, JEJUNUM; SPINAL CORD; SPLEEN; STOMACH; TESTIS; THYMUS; TONGUE; TRACHEA; URINARY BLADDER; GLAND, SALIVARY, SUBMANDIBULAR

Histopathology - The following Tissues were Not Examined:

None

Appendix 21

Individual Macroscopic and Microscopic Pathology

Animal:	2003	Group:	2	Sex:	Male
Species:	Monkey	Strain:	Cyno Mauritius Island		
		Dose:	3 mg/kg /dose		
		Removal Reason:	Terminal Euthanasia		
		Study Day (Week) of Death:	31 (5)		
				Gross Status:	Complete
				Histo Status:	Complete

Gross Pathology Animal Details:

Animal Comment: Gross examination was performed

Gross Pathology Observations [Correlation]:

No observations found

Any remaining protocol required tissues, which have been examined, have no visible lesions

Gross Pathology - The following Tissues were Not Examined:

None

Histopathology Animal Details:

No animal details found

Histopathology Observations [Correlation]:

GLAND, THYROID : Infiltration, mononuclear cell; minimal
KIDNEY : Infiltration, mononuclear cell; minimal
LIVER : Infiltration, mononuclear cell; minimal
LYMPH NODE, AXILLARY : Pigment; mild : (Comment) Black
SITE, INFUSION, CEPHALIC, RIGHT : Infiltration, mixed cell; minimal, subcutaneous tissue

Histopathology - The following Tissues were Within Normal Limits:

ARTERY, AORTA; BONE MARROW, STERNUM; BONE, FEMUR; BONE, STERNUM; BRAIN; EPIDIDYMIS; ESOPHAGUS; EYE; GALLBLADDER; GALT; GLAND, ADRENAL; GLAND, MAMMARY; GLAND, PARATHYROID; GLAND, PITUITARY; GLAND, PROSTATE; GLAND, SEMINAL VESICLE; HEART; JOINT, FEMOROTIBIAL; LARGE INTESTINE, CECUM; LARGE INTESTINE, COLON; LARGE INTESTINE, RECTUM; LUNG; LYMPH NODE, MANDIBULAR; LYMPH NODE, MESENTERIC; MUSCLE, QUADRICEPS; NERVE, OPTIC; NERVE, SCIATIC; PANCREAS; SITE, INFUSION, CEPHALIC, LEFT; SKIN; SMALL INTESTINE, DUODENUM; SMALL INTESTINE, ILEUM; SMALL INTESTINE, JEJUNUM; SPINAL CORD; SPLEEN; STOMACH; TESTIS; THYMUS; TONGUE; TRACHEA; URINARY BLADDER; GLAND, SALIVARY, SUBMANDIBULAR

Histopathology - The following Tissues were Not Examined:

None

Appendix 21

Individual Macroscopic and Microscopic Pathology

Animal:	2501	Group:	2	Sex:	Female
Species:	Monkey	Strain:	Cyno Mauritius Island		
		Dose:	3 mg/kg /dose		
		Removal Reason:	Terminal Euthanasia		
		Study Day (Week) of Death:	31 (5)		
				Gross Status:	Complete
				Histo Status:	Complete

Gross Pathology Animal Details:

Animal Comment: Gross examination was performed

Gross Pathology Observations [Correlation]:

OVIDUCT : Cyst, clear; fluid, right, focal : (Comment) 6 x 3 x 2 mm (TGL)

Any remaining protocol required tissues, which have been examined, have no visible lesions

Gross Pathology - The following Tissues were Not Examined:

None

Histopathology Animal Details:

No animal details found

Histopathology Observations [Correlation]:

GLAND, ADRENAL : Infiltration, lymphocytic; minimal
GLAND, THYROID : Infiltration, lymphocytic; focal, mild
SITE, INFUSION, CEPHALIC, RIGHT : Infiltration, mixed cell; minimal, subcutaneous tissue
NO CORRELATE : No correlating lesion [OVIDUCT : Cyst, clear; fluid, right, focal : (Comment) 6 x 3 x 2 mm (G)]

Histopathology - The following Tissues were Within Normal Limits:

ARTERY, AORTA; BONE MARROW, STERNUM; BONE, FEMUR; BONE, STERNUM; BRAIN; CERVIX; ESOPHAGUS; EYE; GALLBLADDER; GALT; GLAND, MAMMARY; GLAND, PITUITARY; HEART; JOINT, FEMOROTIBIAL; KIDNEY; LARGE INTESTINE, CECUM; LARGE INTESTINE, COLON; LARGE INTESTINE, RECTUM; LIVER; LUNG; LYMPH NODE, AXILLARY; LYMPH NODE, MANDIBULAR; LYMPH NODE, MESENTERIC; MUSCLE, QUADRICEPS; NERVE, OPTIC; NERVE, SCIATIC; OVARY; PANCREAS; SITE, INFUSION, CEPHALIC, LEFT; SKIN; SMALL INTESTINE, DUODENUM; SMALL INTESTINE, ILEUM; SMALL INTESTINE, JEJUNUM; SPINAL CORD; SPLEEN; STOMACH; THYMUS; TONGUE; TRACHEA; URINARY BLADDER; UTERUS; VAGINA; GLAND, SALIVARY, SUBMANDIBULAR

Histopathology - The following Tissues were Not Examined:

GLAND, PARATHYROID - Not Present In Section.
OVIDUCT - Not Present In Section.

Appendix 21

Individual Macroscopic and Microscopic Pathology

Animal:	2502	Group:	2	Sex:	Female
Species:	Monkey	Strain:	Cyno Mauritius Island		
		Dose:	3 mg/kg /dose		
		Removal Reason:	Terminal Euthanasia		
		Study Day (Week) of Death:	31 (5)		
				Gross Status:	Complete
				Histo Status:	Complete

Gross Pathology Animal Details:

Animal Comment: Gross examination was performed

Gross Pathology Observations [Correlation]:

No observations found

Any remaining protocol required tissues, which have been examined, have no visible lesions

Gross Pathology - The following Tissues were Not Examined:

None

Histopathology Animal Details:

No animal details found

Histopathology Observations [Correlation]:

EYE : (Note) Maculae Unavailable For Evaluation
GLAND, PARATHYROID : (Note) One Of A Pair Available For Evaluation.
GLAND, THYROID : Infiltration, mononuclear cell; minimal
KIDNEY : Infiltration, mononuclear cell; minimal
OVARY : Mineralization; minimal
GLAND, SALIVARY, SUBMANDIBULAR : Infiltration, lymphocytic; focal, moderate

Histopathology - The following Tissues were Within Normal Limits:

ARTERY, AORTA; BONE MARROW, STERNUM; BONE, FEMUR; BONE, STERNUM; BRAIN; CERVIX; ESOPHAGUS; EYE; GALLBLADDER; GALT; GLAND, ADRENAL; GLAND, MAMMARY; GLAND, PARATHYROID; GLAND, PITUITARY; HEART; JOINT, FEMOROTIBIAL; LARGE INTESTINE, CECUM; LARGE INTESTINE, COLON; LARGE INTESTINE, RECTUM; LIVER; LUNG; LYMPH NODE, AXILLARY; LYMPH NODE, MANDIBULAR; LYMPH NODE, MESENTERIC; MUSCLE, QUADRICEPS; NERVE, OPTIC; NERVE, SCIATIC; PANCREAS; SITE, INFUSION, CEPHALIC, LEFT; SITE, INFUSION, CEPHALIC, RIGHT; SKIN; SMALL INTESTINE, DUODENUM; SMALL INTESTINE, ILEUM; SMALL INTESTINE, JEJUNUM; SPINAL CORD; SPLEEN; STOMACH; THYMUS; TONGUE; TRACHEA; URINARY BLADDER; UTERUS; VAGINA

Histopathology - The following Tissues were Not Examined:

None

Appendix 21

Individual Macroscopic and Microscopic Pathology

Animal: 2503	Group: 2		Sex: Female
Species: Monkey	Strain: Cyno Mauritius Island		
	Dose: 3 mg/kg /dose		
	Removal Reason: Terminal Euthanasia		
	Study Day (Week) of Death: 31 (5)		
		Gross Status: Complete	
		Histo Status: Complete	

Gross Pathology Animal Details:

Animal Comment: Gross examination was performed

Gross Pathology Observations [Correlation]:

No observations found

Any remaining protocol required tissues, which have been examined, have no visible lesions

Gross Pathology - The following Tissues were Not Examined:

None

Histopathology Animal Details:

No animal details found

Histopathology Observations [Correlation]:

GLAND, PARATHYROID : (Note) One Of A Pair Available For Evaluation.
LYMPH NODE, AXILLARY : Pigment; mild : (Comment) Black

Histopathology - The following Tissues were Within Normal Limits:

ARTERY, AORTA; BONE MARROW, STERNUM; BONE, FEMUR; BONE, STERNUM; BRAIN; CERVIX; ESOPHAGUS; EYE; GALLBLADDER; GALT; GLAND, ADRENAL; GLAND, MAMMARY; GLAND, PARATHYROID; GLAND, PITUITARY; GLAND, THYROID; HEART; JOINT, FEMOROTIBIAL; KIDNEY; LARGE INTESTINE, CECUM; LARGE INTESTINE, COLON; LARGE INTESTINE, RECTUM; LIVER; LUNG; LYMPH NODE, MANDIBULAR; LYMPH NODE, MESENTERIC; MUSCLE, QUADRICEPS; NERVE, OPTIC; NERVE, SCIATIC; OVARY; PANCREAS; SITE, INFUSION, CEPHALIC, LEFT; SITE, INFUSION, CEPHALIC, RIGHT; SKIN; SMALL INTESTINE, DUODENUM; SMALL INTESTINE, ILEUM; SMALL INTESTINE, JEJUNUM; SPINAL CORD; SPLEEN; STOMACH; THYMUS; TONGUE; TRACHEA; UTERUS; VAGINA; GLAND, SALIVARY, SUBMANDIBULAR

Histopathology - The following Tissues were Not Examined:

URINARY BLADDER - Insufficient Tissue Available For Evaluation.

Appendix 21

Individual Macroscopic and Microscopic Pathology

Animal: 3001	Group: 3		Sex: Male
Species: Monkey	Strain: Cyno Mauritius Island		
	Dose: 10 mg/kg /dose		
	Removal Reason: Terminal Euthanasia		
	Study Day (Week) of Death: 31 (5)		
		Gross Status:	Complete
		Histo Status:	Complete

Gross Pathology Animal Details:

Animal Comment: Gross examination was performed

Gross Pathology Observations [Correlation]:

No observations found

Any remaining protocol required tissues, which have been examined, have no visible lesions

Gross Pathology - The following Tissues were Not Examined:

None

Histopathology Animal Details:

No animal details found

Histopathology Observations [Correlation]:

GLAND, PITUITARY : Infiltration, lymphocytic; focal, minimal, pars intermedia
GLAND, THYROID : Infiltration, mononuclear cell; minimal
KIDNEY : Infiltration, mononuclear cell; minimal
SITE, INFUSION, CEPHALIC, LEFT : Infiltration, mixed cell; mild, subcutaneous tissue
SITE, INFUSION, CEPHALIC, LEFT : Hemorrhage; mild, subcutaneous tissue

Histopathology - The following Tissues were Within Normal Limits:

ARTERY, AORTA; BONE MARROW, STERNUM; BONE, FEMUR; BONE, STERNUM; BRAIN; EPIDIDYMIS; ESOPHAGUS; EYE; GALLBLADDER; GALT; GLAND, ADRENAL; GLAND, MAMMARY; GLAND, PROSTATE; GLAND, SEMINAL VESICLE; HEART; JOINT, FEMOROTIBIAL; LARGE INTESTINE, CECUM; LARGE INTESTINE, COLON; LARGE INTESTINE, RECTUM; LIVER; LUNG; LYMPH NODE, AXILLARY; LYMPH NODE, MANDIBULAR; LYMPH NODE, MESENTERIC; MUSCLE, QUADRICEPS; NERVE, OPTIC; NERVE, SCIATIC; PANCREAS; SITE, INFUSION, CEPHALIC, RIGHT; SKIN; SMALL INTESTINE, DUODENUM; SMALL INTESTINE, ILEUM; SMALL INTESTINE, JEJUNUM; SPINAL CORD; SPLEEN; STOMACH; TESTIS; THYMUS; TONGUE; TRACHEA; URINARY BLADDER; GLAND, SALIVARY, SUBMANDIBULAR

Histopathology - The following Tissues were Not Examined:

GLAND, PARATHYROID - Not Present In Section.

Appendix 21

Individual Macroscopic and Microscopic Pathology

Animal:	3002	Group:	3	Sex:	Male
Species:	Monkey	Strain:	Cyno Mauritius Island		
		Dose:	10 mg/kg /dose		
		Removal Reason:	Terminal Euthanasia		
		Study Day (Week) of Death:	31 (5)		
				Gross Status:	Complete
				Histo Status:	Complete

Gross Pathology Animal Details:

Animal Comment: Gross examination was performed

Gross Pathology Observations [Correlation]:

No observations found

Any remaining protocol required tissues, which have been examined, have no visible lesions

Gross Pathology - The following Tissues were Not Examined:

None

Histopathology Animal Details:

No animal details found

Histopathology Observations [Correlation]:

EYE : (Note) Maculae Unavailable For Evaluation
GLAND, PARATHYROID : (Note) One Of A Pair Available For Evaluation.
HEART : Cyst; squamous, multiple
LUNG : Infiltration, mononuclear cell; focal, minimal
SITE, INFUSION, CEPHALIC, LEFT : Infiltration, mixed cell; minimal, subcutaneous tissue

Histopathology - The following Tissues were Within Normal Limits:

ARTERY, AORTA; BONE MARROW, STERNUM; BONE, FEMUR; BONE, STERNUM; BRAIN; EPIDIDYMIS; ESOPHAGUS; EYE; GALLBLADDER; GALT; GLAND, ADRENAL; GLAND, MAMMARY; GLAND, PARATHYROID; GLAND, PITUITARY; GLAND, PROSTATE; GLAND, SEMINAL VESICLE; GLAND, THYROID; JOINT, FEMOROTIBIAL; KIDNEY; LARGE INTESTINE, CECUM; LARGE INTESTINE, COLON; LARGE INTESTINE, RECTUM; LIVER; LYMPH NODE, AXILLARY; LYMPH NODE, MANDIBULAR; LYMPH NODE, MESENTERIC; MUSCLE, QUADRICEPS; NERVE, OPTIC; NERVE, SCIATIC; PANCREAS; SITE, INFUSION, CEPHALIC, RIGHT; SKIN; SMALL INTESTINE, DUODENUM; SMALL INTESTINE, ILEUM; SMALL INTESTINE, JEJUNUM; SPINAL CORD; SPLEEN; STOMACH; TESTIS; THYMUS; TONGUE; TRACHEA; URINARY BLADDER; GLAND, SALIVARY, SUBMANDIBULAR

Histopathology - The following Tissues were Not Examined:

None

Appendix 21

Individual Macroscopic and Microscopic Pathology

Animal:	3003	Group:	3	Sex:	Male
Species:	Monkey	Strain:	Cyno Mauritius Island		
		Dose:	10 mg/kg /dose		
		Removal Reason:	Terminal Euthanasia		
		Study Day (Week) of Death:	31 (5)		
				Gross Status:	Complete
				Histo Status:	Complete

Gross Pathology Animal Details:

Animal Comment: Gross examination was performed

Gross Pathology Observations [Correlation]:

No observations found

Any remaining protocol required tissues, which have been examined, have no visible lesions

Gross Pathology - The following Tissues were Not Examined:

None

Histopathology Animal Details:

No animal details found

Histopathology Observations [Correlation]:

EPIDIDYMIS : Infiltration, mixed cell; focal, mild : (Comment) With edema
EYE : (Note) Maculae Unavailable For Evaluation
GLAND, PARATHYROID : Cyst
SITE, INFUSION, CEPHALIC, LEFT : Infiltration, mixed cell; minimal, subcutaneous tissue
THYMUS : Cellularity, decreased; cortical, minimal
TONGUE : Infiltration, mixed cell; submucosal, mild

Histopathology - The following Tissues were Within Normal Limits:

ARTERY, AORTA; BONE MARROW, STERNUM; BONE, FEMUR; BONE, STERNUM; BRAIN; ESOPHAGUS; EYE; GALLBLADDER; GALT; GLAND, ADRENAL; GLAND, MAMMARY; GLAND, PITUITARY; GLAND, PROSTATE; GLAND, SEMINAL VESICLE; GLAND, THYROID; HEART; JOINT, FEMOROTIBIAL; KIDNEY; LARGE INTESTINE, CECUM; LARGE INTESTINE, COLON; LARGE INTESTINE, RECTUM; LIVER; LUNG; LYMPH NODE, AXILLARY; LYMPH NODE, MANDIBULAR; LYMPH NODE, MESENTERIC; MUSCLE, QUADRICEPS; NERVE, OPTIC; NERVE, SCIATIC; PANCREAS; SITE, INFUSION, CEPHALIC, RIGHT; SKIN; SMALL INTESTINE, DUODENUM; SMALL INTESTINE, ILEUM; SMALL INTESTINE, JEJUNUM; SPINAL CORD; SPLEEN; STOMACH; TESTIS; TRACHEA; URINARY BLADDER; GLAND, SALIVARY, SUBMANDIBULAR

Histopathology - The following Tissues were Not Examined:

None

Appendix 21

Individual Macroscopic and Microscopic Pathology

Animal:	3501	Group:	3	Sex:	Female
Species:	Monkey	Strain:	Cyno Mauritius Island		
		Dose:	10 mg/kg /dose		
		Removal Reason:	Terminal Euthanasia		
		Study Day (Week) of Death:	31 (5)		
				Gross Status:	Complete
				Histo Status:	Complete

Gross Pathology Animal Details:

Animal Comment: Gross examination was performed

Gross Pathology Observations [Correlation]:

BONE, SKULL : Abnormal appearance; deformity (TGL)
BRAIN : Discoloration, dark; tan, left, cerebrum (TGL)

Any remaining protocol required tissues, which have been examined, have no visible lesions

Gross Pathology - The following Tissues were Not Examined:

None

Histopathology Animal Details:

No animal details found

Histopathology Observations [Correlation]:

BONE, SKULL : Fracture; chronic : (Comment) Nonunion [BONE, SKULL : Abnormal appearance; deformity (G)]
GLAND, ADRENAL : Mineralization; minimal
HEART : Infiltration, mononuclear cell; minimal
LIVER : Infiltration, mononuclear cell; minimal
LUNG : Infiltration, mononuclear cell; minimal
LYMPH NODE, AXILLARY : Pigment; minimal
NO CORRELATE : No correlating lesion [BRAIN : Discoloration, dark; tan, left, cerebrum (G)]

Histopathology - The following Tissues were Within Normal Limits:

ARTERY, AORTA; BONE MARROW, STERNUM; BONE, FEMUR; BONE, STERNUM; BRAIN; CERVIX; ESOPHAGUS; EYE; GALLBLADDER; GALT; GLAND, MAMMARY; GLAND, PARATHYROID; GLAND, PITUITARY; GLAND, THYROID; JOINT, FEMOROTIBIAL; KIDNEY; LARGE INTESTINE, CECUM; LARGE INTESTINE, COLON; LARGE INTESTINE, RECTUM; LYMPH NODE, MANDIBULAR; LYMPH NODE, MESENTERIC; MUSCLE, QUADRICEPS; NERVE, OPTIC; NERVE, SCIATIC; OVARY; PANCREAS; SITE, INFUSION, CEPHALIC, LEFT; SITE, INFUSION, CEPHALIC, RIGHT; SKIN; SMALL INTESTINE, DUODENUM; SMALL INTESTINE, ILEUM; SMALL INTESTINE, JEJUNUM; SPINAL CORD; SPLEEN; STOMACH; THYMUS; TONGUE; TRACHEA; URINARY BLADDER; UTERUS; VAGINA; GLAND, SALIVARY, SUBMANDIBULAR

Histopathology - The following Tissues were Not Examined:

None

Appendix 21

Individual Macroscopic and Microscopic Pathology

Animal:	3502	Group:	3	Sex:	Female
Species:	Monkey	Strain:	Cyno Mauritius Island		
		Dose:	10 mg/kg /dose		
		Removal Reason:	Terminal Euthanasia		
		Study Day (Week) of Death:	31 (5)		
				Gross Status:	Complete
				Histo Status:	Complete

Gross Pathology Animal Details:

Animal Comment: Gross examination was performed

Gross Pathology Observations [Correlation]:

No observations found

Any remaining protocol required tissues, which have been examined, have no visible lesions

Gross Pathology - The following Tissues were Not Examined:

None

Histopathology Animal Details:

No animal details found

Histopathology Observations [Correlation]:

GLAND, THYROID : Ectopia : (Comment) Thymus
TONGUE : Infiltration, mononuclear cell; minimal

Histopathology - The following Tissues were Within Normal Limits:

ARTERY, AORTA; BONE MARROW, STERNUM; BONE, FEMUR; BONE, STERNUM; BRAIN; CERVIX; ESOPHAGUS; EYE; GALLBLADDER; GALT; GLAND, ADRENAL; GLAND, MAMMARY; GLAND, PARATHYROID; GLAND, PITUITARY; HEART; JOINT, FEMOROTIBIAL; KIDNEY; LARGE INTESTINE, CECUM; LARGE INTESTINE, COLON; LARGE INTESTINE, RECTUM; LIVER; LUNG; LYMPH NODE, AXILLARY; LYMPH NODE, MANDIBULAR; LYMPH NODE, MESENTERIC; MUSCLE, QUADRICEPS; NERVE, OPTIC; NERVE, SCIATIC; OVARY; PANCREAS; SITE, INFUSION, CEPHALIC, LEFT; SITE, INFUSION, CEPHALIC, RIGHT; SKIN; SMALL INTESTINE, DUODENUM; SMALL INTESTINE, ILEUM; SMALL INTESTINE, JEJUNUM; SPINAL CORD; SPLEEN; STOMACH; THYMUS; TRACHEA; URINARY BLADDER; UTERUS; VAGINA; GLAND, SALIVARY, SUBMANDIBULAR

Histopathology - The following Tissues were Not Examined:

None

Appendix 21

Individual Macroscopic and Microscopic Pathology

Animal: 3503	Group: 3		Sex: Female
Species: Monkey	Strain: Cyno Mauritius Island		
	Dose: 10 mg/kg /dose		
	Removal Reason: Terminal Euthanasia		
	Study Day (Week) of Death: 31 (5)		
		Gross Status:	Complete
		Histo Status:	Complete

Gross Pathology Animal Details:

Animal Comment: Gross examination was performed

Gross Pathology Observations [Correlation]:

No observations found

Any remaining protocol required tissues, which have been examined, have no visible lesions

Gross Pathology - The following Tissues were Not Examined:

None

Histopathology Animal Details:

No animal details found

Histopathology Observations [Correlation]:

EYE : (Note) Maculae Unavailable For Evaluation
LYMPH NODE, AXILLARY : Pigment; mild : (Comment) Black

Histopathology - The following Tissues were Within Normal Limits:

ARTERY, AORTA; BONE MARROW, STERNUM; BONE, FEMUR; BONE, STERNUM; BRAIN; CERVIX; ESOPHAGUS; EYE; GALLBLADDER; GALT; GLAND, ADRENAL; GLAND, MAMMARY; GLAND, PARATHYROID; GLAND, PITUITARY; GLAND, THYROID; HEART; JOINT, FEMOROTIBIAL; KIDNEY; LARGE INTESTINE, CECUM; LARGE INTESTINE, COLON; LARGE INTESTINE, RECTUM; LIVER; LUNG; LYMPH NODE, MANDIBULAR; LYMPH NODE, MESENTERIC; MUSCLE, QUADRICEPS; NERVE, OPTIC; NERVE, SCIATIC; OVARY; PANCREAS; SITE, INFUSION, CEPHALIC, LEFT; SITE, INFUSION, CEPHALIC, RIGHT; SKIN; SMALL INTESTINE, DUODENUM; SMALL INTESTINE, ILEUM; SMALL INTESTINE, JEJUNUM; SPINAL CORD; SPLEEN; STOMACH; THYMUS; TONGUE; TRACHEA; URINARY BLADDER; UTERUS; VAGINA; GLAND, SALIVARY, SUBMANDIBULAR

Histopathology - The following Tissues were Not Examined:

None

Appendix 21

Individual Macroscopic and Microscopic Pathology

Animal:	4001	Group:	4	Sex:	Male
Species:	Monkey	Strain:	Cyno Mauritius Island		
		Dose:	100 mg/kg /dose		
		Removal Reason:	Terminal Euthanasia		
		Study Day (Week) of Death:	31 (5)		
				Gross Status:	Complete
				Histo Status:	Complete

Gross Pathology Animal Details:

Animal Comment: Gross examination was performed

Gross Pathology Observations [Correlation]:

No observations found

Any remaining protocol required tissues, which have been examined, have no visible lesions

Gross Pathology - The following Tissues were Not Examined:

None

Histopathology Animal Details:

No animal details found

Histopathology Observations [Correlation]:

EYE : (Note) Maculae Unavailable For Evaluation
GLAND, PARATHYROID : (Note) One Of A Pair Available For Evaluation.
KIDNEY : Infiltration, mononuclear cell; minimal
LIVER : Infiltration, mononuclear cell; mild
SITE, INFUSION, CEPHALIC, LEFT : Infiltration, mixed cell; mild, subcutaneous tissue

Histopathology - The following Tissues were Within Normal Limits:

ARTERY, AORTA; BONE MARROW, STERNUM; BONE, FEMUR; BONE, STERNUM; BRAIN; EPIDIDYMIS; ESOPHAGUS; EYE; GALLBLADDER; GALT; GLAND, ADRENAL; GLAND, MAMMARY; GLAND, PARATHYROID; GLAND, PITUITARY; GLAND, PROSTATE; GLAND, SEMINAL VESICLE; GLAND, THYROID; HEART; JOINT, FEMOROTIBIAL; LARGE INTESTINE, CECUM; LARGE INTESTINE, COLON; LARGE INTESTINE, RECTUM; LUNG; LYMPH NODE, AXILLARY; LYMPH NODE, MANDIBULAR; LYMPH NODE, MESENTERIC; MUSCLE, QUADRICEPS; NERVE, OPTIC; NERVE, SCIATIC; PANCREAS; SITE, INFUSION, CEPHALIC, RIGHT; SKIN; SMALL INTESTINE, DUODENUM; SMALL INTESTINE, ILEUM; SMALL INTESTINE, JEJUNUM; SPINAL CORD; SPLEEN; STOMACH; TESTIS; THYMUS; TONGUE; TRACHEA; URINARY BLADDER; GLAND, SALIVARY, SUBMANDIBULAR

Histopathology - The following Tissues were Not Examined:

None

Appendix 21

Individual Macroscopic and Microscopic Pathology

Animal:	4002	Group:	4	Sex:	Male
Species:	Monkey	Strain:	Cyno Mauritius Island		
		Dose:	100 mg/kg /dose		
		Removal Reason:	Terminal Euthanasia		
		Study Day (Week) of Death:	31 (5)		
				Gross Status:	Complete
				Histo Status:	Complete

Gross Pathology Animal Details:

Animal Comment: Gross examination was performed

Gross Pathology Observations [Correlation]:

No observations found

Any remaining protocol required tissues, which have been examined, have no visible lesions

Gross Pathology - The following Tissues were Not Examined:

None

Histopathology Animal Details:

No animal details found

Histopathology Observations [Correlation]:

No observations found

Histopathology - The following Tissues were Within Normal Limits:

ARTERY, AORTA; BONE MARROW, STERNUM; BONE, FEMUR; BONE, STERNUM; BRAIN; EPIDIDYMIS; ESOPHAGUS; EYE; GALLBLADDER; GALT; GLAND, ADRENAL; GLAND, PARATHYROID; GLAND, PITUITARY; GLAND, PROSTATE; GLAND, SEMINAL VESICLE; GLAND, THYROID; HEART; JOINT, FEMOROTIBIAL; KIDNEY; LARGE INTESTINE, CECUM; LARGE INTESTINE, COLON; LARGE INTESTINE, RECTUM; LIVER; LUNG; LYMPH NODE, AXILLARY; LYMPH NODE, MANDIBULAR; LYMPH NODE, MESENTERIC; MUSCLE, QUADRICEPS; NERVE, OPTIC; NERVE, SCIATIC; PANCREAS; SITE, INFUSION, CEPHALIC, LEFT; SITE, INFUSION, CEPHALIC, RIGHT; SKIN; SMALL INTESTINE, DUODENUM; SMALL INTESTINE, ILEUM; SMALL INTESTINE, JEJUNUM; SPINAL CORD; SPLEEN; STOMACH; TESTIS; THYMUS; TONGUE; TRACHEA; URINARY BLADDER; GLAND, SALIVARY, SUBMANDIBULAR

Histopathology - The following Tissues were Not Examined:

GLAND, MAMMARY - Not Present In Section.

Appendix 21

Individual Macroscopic and Microscopic Pathology

Animal: 4003	Group: 4		Sex: Male
Species: Monkey	Strain: Cyno Mauritius Island		
	Dose: 100 mg/kg /dose		
	Removal Reason: Terminal Euthanasia		
	Study Day (Week) of Death: 31 (5)		
		Gross Status:	Complete
		Histo Status:	Complete

Gross Pathology Animal Details:

Animal Comment: Gross examination was performed

Gross Pathology Observations [Correlation]:

STOMACH : Abnormal content; ingesta

Any remaining protocol required tissues, which have been examined, have no visible lesions

Gross Pathology - The following Tissues were Not Examined:

None

Histopathology Animal Details:

No animal details found

Histopathology Observations [Correlation]:

KIDNEY : Infiltration, mononuclear cell; minimal
LIVER : Infiltration, mononuclear cell; minimal
LUNG : Alveolar macrophages, increased; focal, mild

Histopathology - The following Tissues were Within Normal Limits:

ARTERY, AORTA; BONE MARROW, STERNUM; BONE, FEMUR; BONE, STERNUM; BRAIN; EPIDIDYMIS; ESOPHAGUS; EYE; GALLBLADDER; GALT; GLAND, ADRENAL; GLAND, MAMMARY; GLAND, PITUITARY; GLAND, PROSTATE; GLAND, SEMINAL VESICLE; GLAND, THYROID; HEART; JOINT, FEMOROTIBIAL; LARGE INTESTINE, CECUM; LARGE INTESTINE, COLON; LARGE INTESTINE, RECTUM; LYMPH NODE, AXILLARY; LYMPH NODE, MANDIBULAR; LYMPH NODE, MESENTERIC; MUSCLE, QUADRICEPS; NERVE, OPTIC; NERVE, SCIATIC; PANCREAS; SITE, INFUSION, CEPHALIC, LEFT; SITE, INFUSION, CEPHALIC, RIGHT; SKIN; SMALL INTESTINE, DUODENUM; SMALL INTESTINE, ILEUM; SMALL INTESTINE, JEJUNUM; SPINAL CORD; SPLEEN; STOMACH; TESTIS; THYMUS; TONGUE; TRACHEA; URINARY BLADDER; GLAND, SALIVARY, SUBMANDIBULAR

Histopathology - The following Tissues were Not Examined:

GLAND, PARATHYROID - Not Present In Section.

Appendix 21

Individual Macroscopic and Microscopic Pathology

Animal:	4004	Group:	4	Sex:	Male
Species:	Monkey	Strain:	Cyno Mauritius Island		
		Dose:	100 mg/kg /dose		
		Removal Reason:	Recovery Euthanasia		
		Study Day (Week) of Death:	71 (11)		
				Gross Status:	Complete
				Histo Status:	Complete

Gross Pathology Animal Details:

Animal Comment: Gross examination was performed

Gross Pathology Observations [Correlation]:

SPLEEN : (Comment) accessory

Any remaining protocol required tissues, which have been examined, have no visible lesions

Gross Pathology - The following Tissues were Not Examined:

None

Histopathology Animal Details:

No animal details found

Histopathology Observations [Correlation]:

GLAND, PARATHYROID : (Note) One Of A Pair Available For Evaluation.
GLAND, PITUITARY : (Note) Only Pars Distalis Available For Evaluation.
LIVER : Infiltration, mononuclear cell; minimal
GLAND, SALIVARY, SUBMANDIBULAR : Infiltration, mononuclear cell; mild

Histopathology - The following Tissues were Within Normal Limits:

ARTERY, AORTA; BONE MARROW, STERNUM; BONE, FEMUR; BONE, STERNUM; BRAIN; EPIDIDYMIS; ESOPHAGUS; EYE; GALLBLADDER; GALT; GLAND, ADRENAL; GLAND, MAMMARY; GLAND, PARATHYROID; GLAND, PITUITARY; GLAND, PROSTATE; GLAND, SEMINAL VESICLE; GLAND, THYROID; HEART; JOINT, FEMOROTIBIAL; KIDNEY; LARGE INTESTINE, CECUM; LARGE INTESTINE, COLON; LARGE INTESTINE, RECTUM; LUNG; LYMPH NODE, AXILLARY; LYMPH NODE, MANDIBULAR; LYMPH NODE, MESENTERIC; MUSCLE, QUADRICEPS; NERVE, OPTIC; NERVE, SCIATIC; PANCREAS; SITE, INFUSION, CEPHALIC, LEFT; SITE, INFUSION, CEPHALIC, RIGHT; SKIN; SMALL INTESTINE, DUODENUM; SMALL INTESTINE, ILEUM; SMALL INTESTINE, JEJUNUM; SPINAL CORD; SPLEEN; STOMACH; TESTIS; THYMUS; TONGUE; TRACHEA; URINARY BLADDER

Histopathology - The following Tissues were Not Examined:

None

Appendix 21

Individual Macroscopic and Microscopic Pathology

Animal:	4005	Group:	4	Sex:	Male
Species:	Monkey	Strain:	Cyno Mauritius Island		
		Dose:	100 mg/kg /dose		
		Removal Reason:	Recovery Euthanasia		
		Study Day (Week) of Death:	71 (11)		
				Gross Status:	Complete
				Histo Status:	Complete

Gross Pathology Animal Details:

Animal Comment: Gross examination was performed

Gross Pathology Observations [Correlation]:

No observations found

Any remaining protocol required tissues, which have been examined, have no visible lesions

Gross Pathology - The following Tissues were Not Examined:

None

Histopathology Animal Details:

No animal details found

Histopathology Observations [Correlation]:

EYE : (Note) Maculae Unavailable For Evaluation

Histopathology - The following Tissues were Within Normal Limits:

ARTERY, AORTA; BONE MARROW, STERNUM; BONE, FEMUR; BONE, STERNUM; BRAIN; EPIDIDYMIS; ESOPHAGUS; EYE; GALLBLADDER; GALT; GLAND, ADRENAL; GLAND, MAMMARY; GLAND, PARATHYROID; GLAND, PITUITARY; GLAND, PROSTATE; GLAND, SEMINAL VESICLE; GLAND, THYROID; HEART; JOINT, FEMOROTIBIAL; KIDNEY; LARGE INTESTINE, CECUM; LARGE INTESTINE, COLON; LARGE INTESTINE, RECTUM; LIVER; LUNG; LYMPH NODE, AXILLARY; LYMPH NODE, MANDIBULAR; LYMPH NODE, MESENTERIC; MUSCLE, QUADRICEPS; NERVE, OPTIC; NERVE, SCIATIC; PANCREAS; SITE, INFUSION, CEPHALIC, LEFT; SITE, INFUSION, CEPHALIC, RIGHT; SKIN; SMALL INTESTINE, DUODENUM; SMALL INTESTINE, ILEUM; SMALL INTESTINE, JEJUNUM; SPINAL CORD; SPLEEN; STOMACH; TESTIS; THYMUS; TONGUE; TRACHEA; URINARY BLADDER; GLAND, SALIVARY, SUBMANDIBULAR

Histopathology - The following Tissues were Not Examined:

None

Appendix 21

Individual Macroscopic and Microscopic Pathology

Animal:	4501	Group:	4	Sex:	Female
Species:	Monkey	Strain:	Cyno Mauritius Island		
		Dose:	100 mg/kg /dose		
		Removal Reason:	Terminal Euthanasia		
		Study Day (Week) of Death:	31 (5)		
				Gross Status:	Complete
				Histo Status:	Complete

Gross Pathology Animal Details:

Animal Comment: Gross examination was performed

Gross Pathology Observations [Correlation]:

No observations found

Any remaining protocol required tissues, which have been examined, have no visible lesions

Gross Pathology - The following Tissues were Not Examined:

None

Histopathology Animal Details:

No animal details found

Histopathology Observations [Correlation]:

EYE : (Note) Maculae Unavailable For Evaluation
GLAND, PARATHYROID : (Note) One Of A Pair Available For Evaluation.
KIDNEY : Infiltration, mononuclear cell; minimal
LIVER : Infiltration, mononuclear cell; minimal
LUNG : Infiltration, mononuclear cell; minimal

Histopathology - The following Tissues were Within Normal Limits:

ARTERY, AORTA; BONE MARROW, STERNUM; BONE, FEMUR; BONE, STERNUM; BRAIN; CERVIX; ESOPHAGUS; EYE; GALLBLADDER; GALT; GLAND, ADRENAL; GLAND, MAMMARY; GLAND, PARATHYROID; GLAND, PITUITARY; GLAND, THYROID; HEART; JOINT, FEMOROTIBIAL; LARGE INTESTINE, CECUM; LARGE INTESTINE, COLON; LARGE INTESTINE, RECTUM; LYMPH NODE, AXILLARY; LYMPH NODE, MANDIBULAR; LYMPH NODE, MESENTERIC; MUSCLE, QUADRICEPS; NERVE, OPTIC; NERVE, SCIATIC; OVARY; PANCREAS; SITE, INFUSION, CEPHALIC, LEFT; SITE, INFUSION, CEPHALIC, RIGHT; SKIN; SMALL INTESTINE, DUODENUM; SMALL INTESTINE, ILEUM; SMALL INTESTINE, JEJUNUM; SPINAL CORD; SPLEEN; STOMACH; THYMUS; TONGUE; TRACHEA; URINARY BLADDER; UTERUS; VAGINA; GLAND, SALIVARY, SUBMANDIBULAR

Histopathology - The following Tissues were Not Examined:

None

Appendix 21

Individual Macroscopic and Microscopic Pathology

Animal:	4502	Group:	4	Sex:	Female
Species:	Monkey	Strain:	Cyno Mauritius Island		
		Dose:	100 mg/kg /dose		
		Removal Reason:	Terminal Euthanasia		
		Study Day (Week) of Death:	31 (5)		
				Gross Status:	Complete
				Histo Status:	Complete

Gross Pathology Animal Details:

Animal Comment: Gross examination was performed

Gross Pathology Observations [Correlation]:

No observations found

Any remaining protocol required tissues, which have been examined, have no visible lesions

Gross Pathology - The following Tissues were Not Examined:

None

Histopathology Animal Details:

No animal details found

Histopathology Observations [Correlation]:

EYE : (Note) Maculae Unavailable For Evaluation
GLAND, THYROID : Ectopia : (Comment) Thymus

Histopathology - The following Tissues were Within Normal Limits:

ARTERY, AORTA; BONE MARROW, STERNUM; BONE, FEMUR; BONE, STERNUM; BRAIN; CERVIX; ESOPHAGUS; EYE; GALLBLADDER; GALT; GLAND, ADRENAL; GLAND, MAMMARY; GLAND, PARATHYROID; GLAND, PITUITARY; HEART; JOINT, FEMOROTIBIAL; KIDNEY; LARGE INTESTINE, CECUM; LARGE INTESTINE, COLON; LARGE INTESTINE, RECTUM; LIVER; LUNG; LYMPH NODE, AXILLARY; LYMPH NODE, MANDIBULAR; LYMPH NODE, MESENTERIC; MUSCLE, QUADRICEPS; NERVE, OPTIC; NERVE, SCIATIC; OVARY; PANCREAS; SITE, INFUSION, CEPHALIC, LEFT; SITE, INFUSION, CEPHALIC, RIGHT; SKIN; SMALL INTESTINE, DUODENUM; SMALL INTESTINE, ILEUM; SMALL INTESTINE, JEJUNUM; SPINAL CORD; SPLEEN; STOMACH; THYMUS; TONGUE; TRACHEA; URINARY BLADDER; UTERUS; VAGINA; GLAND, SALIVARY, SUBMANDIBULAR

Histopathology - The following Tissues were Not Examined:

None

Appendix 21

Individual Macroscopic and Microscopic Pathology

Animal:	4503	Group:	4	Sex:	Female
Species:	Monkey	Strain:	Cyno Mauritius Island		
		Dose:	100 mg/kg /dose		
		Removal Reason:	Terminal Euthanasia		
		Study Day (Week) of Death:	31 (5)		
				Gross Status:	Complete
				Histo Status:	Complete

Gross Pathology Animal Details:

Animal Comment: Gross examination was performed

Gross Pathology Observations [Correlation]:

OVARY : Cyst, clear; fluid, left, focal : (Comment) 11 x 9 x 5 mm (TGL)

Any remaining protocol required tissues, which have been examined, have no visible lesions

Gross Pathology - The following Tissues were Not Examined:

None

Histopathology Animal Details:

No animal details found

Histopathology Observations [Correlation]:

GLAND, PARATHYROID : (Note) One Of A Pair Available For Evaluation.
GLAND, THYROID : Cyst
KIDNEY : Infiltration, mononuclear cell; minimal
LUNG : Infiltration, mononuclear cell; minimal
OVARY : Cyst; luteal [OVARY : Cyst, clear; fluid, left, focal : (Comment) 11 x 9 x 5 mm (G)]

Histopathology - The following Tissues were Within Normal Limits:

ARTERY, AORTA; BONE MARROW, STERNUM; BONE, FEMUR; BONE, STERNUM; BRAIN; CERVIX; ESOPHAGUS; EYE; GALLBLADDER; GALT; GLAND, ADRENAL; GLAND, MAMMARY; GLAND, PARATHYROID; GLAND, PITUITARY; HEART; JOINT, FEMOROTIBIAL; LARGE INTESTINE, CECUM; LARGE INTESTINE, COLON; LARGE INTESTINE, RECTUM; LIVER; LYMPH NODE, AXILLARY; LYMPH NODE, MANDIBULAR; LYMPH NODE, MESENTERIC; MUSCLE, QUADRICEPS; NERVE, OPTIC; NERVE, SCIATIC; PANCREAS; SITE, INFUSION, CEPHALIC, LEFT; SITE, INFUSION, CEPHALIC, RIGHT; SKIN; SMALL INTESTINE, DUODENUM; SMALL INTESTINE, ILEUM; SMALL INTESTINE, JEJUNUM; SPINAL CORD; SPLEEN; STOMACH; THYMUS; TONGUE; TRACHEA; URINARY BLADDER; UTERUS; VAGINA; GLAND, SALIVARY, SUBMANDIBULAR

Histopathology - The following Tissues were Not Examined:

None

Appendix 21

Individual Macroscopic and Microscopic Pathology

Animal:	4504	Group:	4	Sex:	Female
Species:	Monkey	Strain:	Cyno Mauritius Island		
		Dose:	100 mg/kg /dose		
		Removal Reason:	Recovery Euthanasia		
		Study Day (Week) of Death:	71 (11)		
				Gross Status:	Complete
				Histo Status:	Complete

Gross Pathology Animal Details:

Animal Comment: Gross examination was performed

Gross Pathology Observations [Correlation]:

No observations found

Any remaining protocol required tissues, which have been examined, have no visible lesions

Gross Pathology - The following Tissues were Not Examined:

None

Histopathology Animal Details:

No animal details found

Histopathology Observations [Correlation]:

GLAND, PITUITARY : Cyst; pars distalis

Histopathology - The following Tissues were Within Normal Limits:

ARTERY, AORTA; BONE MARROW, STERNUM; BONE, FEMUR; BONE, STERNUM; BRAIN; CERVIX; ESOPHAGUS; EYE; GALLBLADDER; GALT; GLAND, ADRENAL; GLAND, MAMMARY; GLAND, PARATHYROID; GLAND, THYROID; HEART; JOINT, FEMOROTIBIAL; KIDNEY; LARGE INTESTINE, CECUM; LARGE INTESTINE, COLON; LARGE INTESTINE, RECTUM; LIVER; LUNG; LYMPH NODE, AXILLARY; LYMPH NODE, MANDIBULAR; LYMPH NODE, MESENTERIC; MUSCLE, QUADRICEPS; NERVE, OPTIC; NERVE, SCIATIC; OVARY; PANCREAS; SITE, INFUSION, CEPHALIC, LEFT; SITE, INFUSION, CEPHALIC, RIGHT; SKIN; SMALL INTESTINE, DUODENUM; SMALL INTESTINE, ILEUM; SMALL INTESTINE, JEJUNUM; SPINAL CORD; SPLEEN; STOMACH; THYMUS; TONGUE; TRACHEA; URINARY BLADDER; UTERUS; VAGINA; GLAND, SALIVARY, SUBMANDIBULAR

Histopathology - The following Tissues were Not Examined:

None

Appendix 21

Individual Macroscopic and Microscopic Pathology

Animal:	4505	Group:	4	Sex:	Female
Species:	Monkey	Strain:	Cyno Mauritius Island		
		Dose:	100 mg/kg /dose		
		Removal Reason:	Recovery Euthanasia		
		Study Day (Week) of Death:	71 (11)		
				Gross Status:	Complete
				Histo Status:	Complete

Gross Pathology Animal Details:

Animal Comment: Gross examination was performed

Gross Pathology Observations [Correlation]:

OVARY : Enlargement; bilateral (TGL)
UTERUS : Discoloration, dark; red (TGL)

Any remaining protocol required tissues, which have been examined, have no visible lesions

Gross Pathology - The following Tissues were Not Examined:

None

Histopathology Animal Details:

No animal details found

Histopathology Observations [Correlation]:

CERVIX : Dilatation; moderate, lumen
GLAND, THYROID : Infiltration, mononuclear cell; minimal
OVARY : Hypertrophy; bilateral, moderate, corpus luteum [OVARY : Enlargement; bilateral (G)]
NO CORRELATE : No correlating lesion [UTERUS : Discoloration, dark; red (G)]
GLAND, SALIVARY, SUBMANDIBULAR : Infiltration, mononuclear cell; minimal

Histopathology - The following Tissues were Within Normal Limits:

ARTERY, AORTA; BONE MARROW, STERNUM; BONE, FEMUR; BONE, STERNUM; BRAIN; ESOPHAGUS; EYE; GALLBLADDER; GALT; GLAND, ADRENAL; GLAND, MAMMARY; GLAND, PITUITARY; HEART; JOINT, FEMOROTIBIAL; KIDNEY; LARGE INTESTINE, CECUM; LARGE INTESTINE, COLON; LARGE INTESTINE, RECTUM; LIVER; LUNG; LYMPH NODE, AXILLARY; LYMPH NODE, MANDIBULAR; LYMPH NODE, MESENTERIC; MUSCLE, QUADRICEPS; NERVE, OPTIC; NERVE, SCIATIC; PANCREAS; SITE, INFUSION, CEPHALIC, LEFT; SITE, INFUSION, CEPHALIC, RIGHT; SKIN; SMALL INTESTINE, DUODENUM; SMALL INTESTINE, ILEUM; SMALL INTESTINE, JEJUNUM; SPINAL CORD; SPLEEN; STOMACH; THYMUS; TONGUE; TRACHEA; URINARY BLADDER; UTERUS; VAGINA

Histopathology - The following Tissues were Not Examined:

GLAND, PARATHYROID - Not Present In Section.
