# Supplementary material for: VISTA checkpoint inhibition by pH-selective antibody SNS-101 with optimized safety and pharmacokinetic profiles enhances PD-1 response
Source: Nat Commun. 2024 Apr 4;15:2917. doi: 10.1038/s41467-024-47256-x (PMC10995192; doi:10.1038/s41467-024-47256-x)
Supplement: Supplementary file 5 — Reporting Summary [file 41467_2024_47256_MOESM5_ESM.pdf]

nature portfolio

Corresponding author(s): Dr. Edward van der Horst

Last updated by author(s): Mar 1, 2024

Reporting Summary

Nature Portfolio wishes to improve the reproducibility of the work that we publish. This form provides structure for consistency and transparency in reporting. For further information on Nature Portfolio policies, see our Editorial Policies and the Editorial Policy Checklist.

Statistics

For all statistical analyses, confirm that the following items are present in the figure legend, table legend, main text, or Methods section.

n/a	Confirmed	
☐	☒	The exact sample size (*n*) for each experimental group/condition, given as a discrete number and unit of measurement
☐	☒	A statement on whether measurements were taken from distinct samples or whether the same sample was measured repeatedly
☐	☒	The statistical test(s) used AND whether they are one- or two-sided<br>*Only common tests should be described solely by name; describe more complex techniques in the Methods section.*
☐	☒	A description of all covariates tested
☐	☒	A description of any assumptions or corrections, such as tests of normality and adjustment for multiple comparisons
☐	☒	A full description of the statistical parameters including central tendency (e.g. means) or other basic estimates (e.g. regression coefficient) AND variation (e.g. standard deviation) or associated estimates of uncertainty (e.g. confidence intervals)
☐	☒	For null hypothesis testing, the test statistic (e.g. *F*, *t*, *r*) with confidence intervals, effect sizes, degrees of freedom and *P* value noted<br>*Give P values as exact values whenever suitable.*
☒	☐	For Bayesian analysis, information on the choice of priors and Markov chain Monte Carlo settings
☒	☐	For hierarchical and complex designs, identification of the appropriate level for tests and full reporting of outcomes
☒	☐	Estimates of effect sizes (e.g. Cohen's *d*, Pearson's *r*), indicating how they were calculated

Our web collection on statistics for biologists contains articles on many of the points above.

Software and code

Policy information about availability of computer code

Data collection	Bruker Sierra Analyser 3.4.3; Molecular Devices SoftMax® Pro Software v. 6.5.1; Bio-Plex Manager (TM) v. 6.2.0.175 (Bio-Rad); Miltenyi Biotec MACSQuantify v. 2.11.1907.19925
Data analysis	Data was analyzed in Microsoft Excel (multiple versions updated regularly through MS 365 Subscription), GraphPad Prism (versions 8-10), and R (version 4.2.2) with R packages “pheatmap,” “corrplot,” “randomForest,” and “caret.” R scripts used for analyses are available at https://github.com/SenseiBio/SenseiBio.

For manuscripts utilizing custom algorithms or software that are central to the research but not yet described in published literature, software must be made available to editors and reviewers. We strongly encourage code deposition in a community repository (e.g. GitHub). See the Nature Portfolio guidelines for submitting code & software for further information.

Data

Policy information about availability of data

All manuscripts must include a data availability statement. This statement should provide the following information, where applicable:

- Accession codes, unique identifiers, or web links for publicly available datasets
- A description of any restrictions on data availability
- For clinical datasets or third party data, please ensure that the statement adheres to our policy

No large-scale data sets were generated during this study. Protein structure data has been deposited at RCSB (www.rcsb.org) with the PDB identifier 8TBQ. Source

data are provided with this paper. Additional Sensei Biotherapeutics materials and data that support the findings of this study may be made available to qualified academic, noncommercial researchers through a materials transfer agreement. Contact the corresponding offer and info@senseibio.com for more information.

Research involving human participants, their data, or biological material

Policy information about studies with human participants or human data. See also policy information about sex, gender (identity/presentation), and sexual orientation and race, ethnicity and racism.

Reporting on sex and gender	For ID.Flow Cytokine Release assays, blood was donated from healthy female and male volunteers 18 years and older; four female and two males donors were included in the study.
Reporting on race, ethnicity, or other socially relevant groupings	Human blood from anonymous donors was used for experiments; race, ethnicity or other socially relevant groupings information were not collected.
Population characteristics	Human blood from anonymous donors was used for experiments, and no population information was collected.
Recruitment	Healthy volunteers 18 years and older. Exclusion criteria included intake of NSAID or any kind of corticosteroids (within 7 days from blood donation) or ongoing acute infection or sickness within the last 7 days (for example: common cold).
Ethics oversight	Ethics approval to Immuneed AB by Regionala etikprövningsnämnden i Uppsala (now national Ethics Approval Authority) Reference number 2015/325 Approved 2015-11-04

Note that full information on the approval of the study protocol must also be provided in the manuscript.

Field-specific reporting

Please select the one below that is the best fit for your research. If you are not sure, read the appropriate sections before making your selection.

☒ Life sciences ☐ Behavioural & social sciences ☐ Ecological, evolutionary & environmental sciences

For a reference copy of the document with all sections, see nature.com/documents/nr-reporting-summary-flat.pdf

Life sciences study design

All studies must disclose on these points even when the disclosure is negative.

Sample size	No statistical method was used to predetermine sample size. Sample size in the mouse studies were based on preliminary experimentation and we designed our experiments to achieve statistical power by using n ≥ 8 mice per group/condition, while responsibly using animals.
Data exclusions	Only data from assay development and technically failed experiments were excluded.
Replication	The number of replicates for each study is indicated within the manuscript, with all technically successful replicate studies reproducing the indicated results. The cynomolgus monkey pharmacokinetics and ex vivo CRS experiments were conducted once due to the significant ethical, logistical, and financial considerations associated with using non-human primates in research. These studies were designed with rigorous methodological planning to ensure robustness and reliability of the data from a single iteration. Additionally, the ethical use of animals in research mandates minimizing the number of animals used. Our experimental designs, therefore, adhered to the principles of the 3Rs (Replacement, Reduction, and Refinement) to ensure that we obtained comprehensive and reliable data while respecting ethical guidelines. For experiments other than those involving cynomolgus monkeys and ex vivo CRS, we ensured replication and independent performance as indicated, to validate our findings and adhere to scientific standards for reproducibility.
Randomization	For in vivo studies, animals were randomized at the start of experiment or treatment regimen.
Blinding	Some aspects of our study were conducted externally, under conditions where partial blinding was implemented to minimize bias. For the internal components of our research, blinding was not performed due to the nature of the experimental procedures and the objective metrics used for data collection and analysis. The methodologies employed in our study, including automated data acquisition and quantitative analyses, are designed to be objective and minimize the potential for experimenter bias. Furthermore, the endpoints measured are largely quantitative and not subject to subjective interpretation, which significantly reduces the risk of bias in ways that blinding would not necessarily mitigate.

Reporting for specific materials, systems and methods

We require information from authors about some types of materials, experimental systems and methods used in many studies. Here, indicate whether each material, system or method listed is relevant to your study. If you are not sure if a list item applies to your research, read the appropriate section before selecting a response.

Materials & experimental systems

n/a	Involved in the study	
☐	☒	Antibodies
☐	☒	Eukaryotic cell lines
☒	☐	Palaeontology and archaeology
☐	☒	Animals and other organisms
☒	☐	Clinical data
☒	☐	Dual use research of concern
☒	☐	Plants

Methods

n/a	Involved in the study	
☒	☐	ChIP-seq
☐	☒	Flow cytometry
☒	☐	MRI-based neuroimaging

Antibodies

Antibodies used

[Antibody, Clone, Supplier, Catalog #]
Anti-SNS-101 anti-idiotypic antibody, AbD51857ad, produced by Bio-Rad
Anti-SNS-101 anti-idiotypic antibody, AbD51841rao, produced by Bio-Rad
Ultra-LEAF Purified Human IgG1 Isotype Control, BioLegend, 403501
anti-human IgG1-Fc, AbD27686, Bio-Rad, HCA285
mouse anti-human IgG Fc, JDC-10, Abcam, ab99757
peroxidase-conjugated mouse anti-human IgG F(ab)2 fragment-specific polyclonal reagent, Jackson Laboratories, 209-035-097
rat α-mPD-1, RMP1-14, Bio X Cell, BP0146
rat IgG2a, 2A3 isotype control, Bio X Cell, BP0089
mouse IgG2a, C1.18.4 isotype control, Bio X Cell, BP0085
rat IgG2a, 1-1, Leinco, I-1177
human IgG1 isotype control Ab, Bio X Cell, BP0297
anti-mouse PD-1, RMP1-14, Leinco P372
anti-mouse CTLA-4, 9H10, Bio X Cell, BE0131
anti-human CD3, OKT-3, Bio X Cell, BE0001-2
anti-mouse CD45 FITC, 30-F11, Thermo Fisher Scientific, 11-0451-82
anti-mouse CD4 Brilliant Violet™ 421, RM4-5, Thermo Fisher Scientific, 404-0042-82
anti-mouse CD8 PE, 53-6.7, Thermo Fisher Scientific, 12-0081-82
anti-human CD45 VioGreen, REA747, Miltenyi Biotec, 130-110-638
anti-human CD3 PE-Vio 770, REA613, Miltenyi Biotec, 130-113-140
anti-human CD16 Vio Bright B515, REA423, Miltenyi Biotec, 130-119-616
anti-human CD14 PE, TÜK4, Miltenyi Biotec, 130-113-147
anti-human CD56 FITC, REA196, Miltenyi Biotec, 130-114-740
anti-human PSGL-1 PE, KPL-1, BioLegend, 328805
anti-human Syndecan-2 PE, 305515, R&D Systems, FAB2965P
anti-human LRIG-1 PE, 789211, R&D Systems, FAB7498P
anti-human VSIG-3 AF647, 973408, R&D Systems, FAB92292R
anti-human VSIG-8 PE, 961823, R&D Systems, FAB9418P
anti-mouse CD45 PerCP-Vio700, REA737, Miltenyi Biotec, 130-110-801
anti-human CD45 APC/Fire750, HI30, BioLegend, 304062
anti-human CD3 Vio Bright R720, REA613, Miltenyi Biotec, 130-127-377
anti-human CD4 VioBlue, REA623, Miltenyi Biotec, 130-114-725
anti-human CD8 PE Vio 615, REA734, Miltenyi Biotec, 130-110-823
anti-human CD56 PE Vio 770, REA196, Miltenyi Biotec, 130-113-313
anti-human CD45RA Brilliant Violet 711, HI100, BioLegend, 304138
anti-human CD197 (CCR7) APC, G043H7, BioLegend, 353214
anti-human CD127 (IL-7Ra) Brilliant Violet 605, A019D5, BioLegend, 351334
anti-human CD25 PE, REA570, Miltenyi Biotec, 130-113-286
anti-human CD19 Brilliant Violet 711, HIB19, BioLegend, 302246
anti-human CD11c PE/Cyanine7, Bu15, BioLegend, 337216
anti-human HLA-DR APC, LN3, BioLegend, 327022
anti-Human CD14 FITC, M5E2 (RUO), BD Biosciences, 555397
anti-human CD16 eFluor450, eBIOCB16, Thermo Fisher Scientific, 48-0168-42
anti-human CD123 Brilliant Violet 605, 6H6, BioLegend 306026
anti-human CD86 PE, REA968, Miltenyi Biotec, 130-116-160

Validation

The anti-SNS-101 anti-idiotypic reagents AbD51857ad and AbD51841rao were validated to bind specifically to SNS-101 in a sandwich ELISA format by the manufacturer (Bio-Rad), and further validation was performed by Charles River who implemented the MSD assay for measuring SNS-101 in clinical serum samples. Validation of commercial antibodies used in this study was done by the manufacturer, and details of these validation efforts are described on the manufacturer's website as indicated below.

Ultra-LEAF Purified Human IgG1 Isotype Control, BioLegend, 403501
https://www.biolegend.com/de-at/products/ultra-leaf-purified-human-igg1-isotype-control-recombinant-antibody-14241
anti-human IgG1-Fc, AbD27686, Bio-Rad, HCA285
https://www.bio-rad-antibodies.com/monoclonal/human-igg1-antibody-abd27686-hca285.html?f=purified
mouse anti-human IgG Fc, JDC-10, Abcam, ab99757
https://www.abcam.com/products/secondary-antibodies/mouse-monoclonal-jdc-10-human-igg-fc-ab99757.html
Peroxidase-conjugated mouse anti-human IgG F(ab)2 fragment-specific polyclonal reagent, Jackson Laboratories, 209-035-097

https://www.jacksonimmuno.com/catalog/products/209-035-097
rat α-mPD-1 RMP1-14, Bio X Cell, BP0146
https://bioxcell.com/invivoplus-anti-mouse-pd-1-cd279-bp0146
rat IgG2a, 2A3 isotype control, Bio X Cell, BP0089
https://bioxcell.com/invivoplus-rat-igg2a-isotype-control-anti-trinitrophenol-bp0089
mouse IgG2a, C1.18.4 isotype control, Bio X Cell, BP0085
https://bioxcell.com/invivoplus-mouse-igg2a-isotype-control-unknown-specificity
rat IgG2a, 1-1, Leinco, I-1177
https://www.leinco.com/p/rat-igg2a-isotype-control-purified-functional-grade-gold/
human IgG1 isotype control Ab, Bio X Cell, BP0297
https://bioxcell.com/invivoplus-human-igg1-isotype-control-bp0297
anti-mouse PD-1, RMP1-14, Leinco, P372
https://www.leinco.com/p/anti-mouse-pd-1-cd279-purified-functional-grade-platinum/
anti-mouse CTLA-4, 9H10, Bio X Cell, BE0131
https://bioxcell.com/invivomab-anti-mouse-ctla-4-cd152-be0131
anti-human CD3, OKT-3, Bio X Cell, BE0001-2
https://bioxcell.com/invivomab-anti-human-cd3-be0001-2
anti-mouse CD45 FITC, 30-F11, Thermo Fisher Scientific, 11-0451-82
https://www.thermofisher.com/antibody/product/CD45-Antibody-clone-30-F11-Monoclonal/11-0451-82
anti-mouse CD4 Brilliant Violet™ 421, RM4-5, Thermo Fisher Scientific, 404-0042-82
https://www.thermofisher.com/antibody/product/CD4-Antibody-clone-RM4-5-Monoclonal/404-0042-82
anti-mouse CD8 PE, 53-6.7, Thermo Fisher Scientific, 12-0081-82
https://www.thermofisher.com/antibody/product/CD8a-Antibody-clone-53-6-7-Monoclonal/12-0081-82
anti-human CD45 VioGreen, REA747, Miltenyi Biotec, 130-110-638
https://www.miltenyibiotec.com/US-en/products/cd45-antibody-anti-human-reafinity-rea747.html#conjugate=viogreen:size=100-tests-in-200-ul
anti-human CD3 PE-Vio 770, REA613, Miltenyi Biotec, 130-113-140
https://www.miltenyibiotec.com/US-en/products/cd3-antibody-anti-human-reafinity-rea613.html#conjugate=pe-vio-770:size=100-tests-in-200-ul
anti-human CD16 Vio Bright, B515, REA423, Miltenyi Biotec, 130-119-616
https://www.miltenyibiotec.com/US-en/products/cd16-antibody-anti-human-reafinity-rea423.html#conjugate=vio-bright-b515:size=100-tests-in-200-ul
anti-human CD14 PE, TÜK4, Miltenyi Biotec, 130-113-147
https://www.miltenyibiotec.com/US-en/products/cd14-antibody-anti-human-tuk4.html#conjugate=pe:size=100-tests-in-200-ul
anti-human CD56 FITC, REA196, Miltenyi Biotec, 130-114-740
https://www.miltenyibiotec.com/US-en/products/cd56-antibody-anti-human-reafinity-rea196.html#conjugate=fitc:size=30-tests-in-60-ul
anti-human PSGL-1 PE, KPL-1, BioLegend 328805
https://www.biolegend.com/ja-jp/products/pe-anti-human-cd162-antibody-4510
anti-human Syndecan-2 PE, 305515, R&D Systems, FAB2965P
https://www.rndsystems.com/products/human-syndecan-2-cd362-pe-conjugated-antibody-305515_fab2965p
anti-human LRIG-1 PE, 789211, R&D Systems, FAB7498P
https://www.rndsystems.com/products/human-lrig1-pe-conjugated-antibody-789211_fab7498p
anti-human VSIG-3 AF647, 973408, R&D Systems, FAB92292R
https://www.rndsystems.com/products/human-vsig3-alexa-fluor-647-conjugated-antibody-973408_fab92292r
anti-human VSIG-8 PE, 961823, R&D Systems, FAB9418P
https://www.rndsystems.com/products/human-vsig8-pe-conjugated-antibody-961823_fab9418p
anti-mouse CD45 PerCP-Vio700, REA737, Miltenyi Biotec, 130-110-801
https://www.miltenyibiotec.com/US-en/products/cd45-antibody-anti-mouse-reafinity-rea737.html#conjugate=percp-vio-700:size=30-ug-in-200-ul
anti-human CD45 APC/Fire750, HI30, BioLegend, 304062
https://www.biolegend.com/en-ie/products/apc-fire-750-anti-human-cd45-antibody-13565?GroupID=BLG5926
anti-human CD3 Vio Bright R720, REA613, Miltenyi Biotec, 130-127-377
https://www.miltenyibiotec.com/US-en/products/cd3-antibody-anti-human-reafinity-rea613.html#conjugate=vio-bright-r720:size=100-tests-in-200-ul
anti-human CD4 VioBlue, REA623, Miltenyi Biotec, 130-114-725
https://www.miltenyibiotec.com/US-en/products/cd4-antibody-anti-human-reafinity-rea623.html#conjugate=vioblue:size=30-tests-in-60-ul
anti-human CD8 PE-Vio 615, REA734, Miltenyi Biotec, 130-110-823
https://www.miltenyibiotec.com/US-en/products/cd8-antibody-anti-human-reafinity-rea734.html#conjugate=pe-vio-670:size=100-tests-in-200-ul
anti-human CD56 PE-Vio 770, REA196, Miltenyi Biotec, 130-113-313
https://www.miltenyibiotec.com/US-en/products/cd56-antibody-anti-human-reafinity-rea196.html#conjugate=pe-vio-670:size=100-tests-in-200-ul
anti-human CD45RA Brilliant Violet 711, HI100, BioLegend, 304138
https://www.biolegend.com/en-ie/products/brilliant-violet-711-anti-human-cd45ra-antibody-7937
anti-human CD197 (CCR7) APC, G043H7, BioLegend, 353214
https://www.biolegend.com/en-ie/products/apc-anti-human-cd197-ccr7-antibody-7536
anti-human CD127 (IL-7Ra) Brilliant Violet 605, A019D5, BioLegend, 351334
https://www.biolegend.com/en-ie/products/brilliant-violet-605-anti-human-cd127-il-7ralpha-antibody-8495
anti-human CD25 PE, REA570, Miltenyi Biotec, 130-113-286
https://www.miltenyibiotec.com/US-en/products/cd25-antibody-anti-human-reafinity-rea570.html#conjugate=vio-r667:size=600-ul
anti-human CD19 Brilliant Violet 711, HIB19, BioLegend, 302246
https://www.biolegend.com/en-ie/products/brilliant-violet-711-anti-human-cd19-antibody-8519
anti-human CD11c PE/Cyanine7, Bu15, BioLegend, 337216
https://www.biolegend.com/en-ie/products/pe-cyanine7-anti-human-cd11c-antibody-6129

anti-human HLA-DR APC, LN3, BioLegend, 327022
https://www.biolegend.com/en-ie/products/apc-anti-human-hla-dr-antibody-16020
anti-human CD14 FITC, M5E2 (RUO), BD Biosciences, 555397
https://www.bdbiosciences.com/en-eu/products/reagents/flow-cytometry-reagents/research-reagents/single-color-antibodies-ruo/fitc-mouse-anti-human-cd14.555397
anti-human CD16 eFluor450, eBIOCB16, Thermo Fisher Scientific, 48-0168-42
https://www.thermofisher.com/antibody/product/CD16-Antibody-clone-eBioCB16-CB16-Monoclonal/48-0168-42
anti-human CD123 Brilliant Violet 605, 6H6, BioLegend 306026
https://www.biolegend.com/en-ie/products/brilliant-violet-605-anti-human-cd123-antibody-8545
anti-human CD86 PE, REA968, Miltenyi Biotec, 130-116-160
https://www.miltenyibiotec.com/US-en/products/cd86-antibody-anti-human-reafinity-rea968.html#conjugate=pe:size=100-tests-in-200-ul

Eukaryotic cell lines

Policy information about cell lines and Sex and Gender in Research

Cell line source(s)	MC38: Kerafast ENH204-FP<br>MB49: Millipore SCC148<br>EG.7-OVA ATCC CRL-2113<br>ExpiCHO-S™ was obtained from Thermo Fisher Scientific<br>MCA/1959 Sarcoma: Schreiber lab, Washington University, St. Louis
Authentication	No cell line authentication was performed beyond information from suppliers.
Mycoplasma contamination	All cell lines were certified as Mycoplasma negative by the suppliers. Random testing was conducted internally; all tests found to be negative.
Commonly misidentified lines<br>(See ICLAC register)	No commonly misidentified cell lines were used.

Animals and other research organisms

Policy information about studies involving animals; ARRIVE guidelines recommended for reporting animal research, and Sex and Gender in Research

Laboratory animals	Male and female C57BL/6N-Vsirtm1(huVSIR-ICP3; Male and female WT C57BL/6. Mice were 6-12 weeks of age. Cynomolgus macaques were males and females (2/sex) 6-24 months of age.
Wild animals	The study did not involve wild animals.
Reporting on sex	Single sex cohorts were used to match origin sex of mouse cancer cell lines.
Field-collected samples	The study did not involve field-collected samples.
Ethics oversight	All in vivo experiments were conducted in accordance with relevant ethical regulations for animal testing and research, in specific-pathogen free facilities at genOway, Murigenics, Charles River Labs, and Washington University in St. Louis. The protocol for non-human primate studies received approval from the Institutional Animal Care and Use Committee (IACUC) at Charles River Labs. Mouse experiments were authorized by the IACUC of Murigenics and the Animal Studies Committee at Washington University School of Medicine in St. Louis.

Note that full information on the approval of the study protocol must also be provided in the manuscript.

Flow Cytometry

Plots

Confirm that:

☒ The axis labels state the marker and fluorochrome used (e.g. CD4-FITC).

☒ The axis scales are clearly visible. Include numbers along axes only for bottom left plot of group (a 'group' is an analysis of identical markers).

☒ All plots are contour plots with outliers or pseudocolor plots.

☒ A numerical value for number of cells or percentage (with statistics) is provided.

Methodology

Sample preparation	Samples were prepared as listed in the methods
Instrument	Miltenyi MACSQuant Analyzer 10 (Miltenyi Biotec)

Software	All flow cytometry data were acquired with MACSQuantify v. 2.11.1907.19925 (Miltenyi Biotec) and analyzed with FlowJo software (BD Biosciences)
Cell population abundance	*Describe the abundance of the relevant cell populations within post-sort fractions, providing details on the purity of the samples and how it was determined.*
Gating strategy	*Describe the gating strategy used for all relevant experiments, specifying the preliminary FSC/SSC gates of the starting cell population, indicating where boundaries between "positive" and "negative" staining cell populations are defined.*

☒ Tick this box to confirm that a figure exemplifying the gating strategy is provided in the Supplementary Information.
